# Thiocyanate Degradation by a Highly Enriched Culture of the Neutrophilic Halophile *Thiohalobacter sp*. Strain FOKN1 from Activated Sludge and Genomic Insights into Thiocyanate Metabolism

**DOI:** 10.1264/jsme2.ME19068

**Published:** 2019-12-27

**Authors:** Mamoru Oshiki, Toshikazu Fukushima, Shuichi Kawano, Yasuhiro Kasahara, Junichi Nakagawa

**Affiliations:** 1 Department of Civil Engineering, National Institute of Technology, Nagaoka College Japan; 2 Advanced Technology Research Laboratories, Research & Development, Nippon Steel Corporation Japan; 3 Department of Computer and Network Engineering Graduate School of Informatics and Engineering, The University of Electro-Communications Japan; 4 Institute of Low-Temperature Science, Hokkaido University Sapporo Japan

**Keywords:** *Thiohalobacter sp*. strain FOKN1, thiocyanate degradation, neutrophilic halophile, whole genome sequence and proteome, thiocyanate dehydrogenase

## Abstract

Thiocyanate (SCN^−^) is harmful to a wide range of organisms, and its removal is essential for environmental protection. A neutrophilic halophile capable of thiocyanate degradation, *Thiohalobacter sp*. strain FOKN1, was highly enriched (relative abundance; 98.4%) from activated sludge collected from a bioreactor receiving thiocyanate-rich wastewater. The enrichment culture degraded 3.38 mM thiocyanate within 140 h, with maximum activity at pH 8.8, 37°C, and 0.18 M sodium chloride. Thiocyanate degradation was inhibited by 30 mg L^−1^ phenol, but not by thiosulfate. Microbial thiocyanate degradation is catalyzed by thiocyanate dehydrogenase, while limited information is currently available on the molecular mechanisms underlying thiocyanate degradation by the thiocyanate dehydrogenase of neutrophilic halophiles. Therefore, (meta)genomic and proteomic analyses of enrichment cultures were performed to elucidate the whole genome sequence and proteome of *Thiohalobacter sp*. strain FOKN1. The 3.23-Mb circular *Thiohalobacter sp*. strain FOKN1 genome was elucidated using a PacBio RSII sequencer, and the expression of 914 proteins was identified by tandem mass spectrometry. The *Thiohalobacter sp*. strain FOKN1 genome had a gene encoding thiocyanate dehydrogenase, which was abundant in the proteome, suggesting that thiocyanate is degraded by thiocyanate dehydrogenase to sulfur and cyanate. The sulfur formed may be oxidized to sulfate by the sequential oxidation reactions of dissimilatory sulfite reductase, adenosine-5′-phosphosulfate reductase, and dissimilatory ATP sulfurylase. Although the *Thiohalobacter sp*. strain FOKN1 genome carried a gene encoding cyanate lyase, its protein expression was not detectable. The present study advances the understanding of the molecular mechanisms underlying thiocyanate degradation by the thiocyanate dehydrogenase of neutrophilic halophiles.

Thiocyanate (SCN^−^) is a major component of wastewater produced during coal gasification in industries such as steel manufacturing (25, 59) and is also a constituent of wastewater produced during gold mining (18). Since SCN^−^ is harmful to a wide range of organisms, including aquatic life forms (7), the degradation of SCN^−^-contaminated wastewater is necessary for the protection of aquatic communities in receiving aquatic environments, including river and sea areas. Biological treatments, such as activated sludge processes (54), are commonly employed, and several SCN^−^-degrading bacteria have been identified, *e.g*., the freshwater bacterium *Thiobacillus thioparus* (24) (For a review, see 18). Apart from freshwater species, few bacteria are known to degrade SCN^−^ under halophilic conditions, and these halophiles are attractive candidates for the biological treatment of coke-oven wastewater discharged from steel-manufacturing plants. Coke-oven wastewater contains high concentrations of SCN^−^ (3–9 mM), phenols (330–1,040 mg L^−1^), and NH_4_^+^ (24–364 mM) (59), and this wastewater is often diluted prior to biological treatments in order to decrease the concentration and toxicity of contaminants (30, 46, 55). Seawater is a commonly used diluent due to its availability and low cost. A dilution ratio of 1:1–5 (v/v) results in neutrophilic (pH 8.1–8.2) and halophilic conditions (30). To date, three halophiles that are capable of SCN^−^ degradation have been described, including the neutrophilic halophiles, *Thiohalobacter thiocyanaticus* (52) and *Thiohalophilus thiocyanatoxidans* (51), and the alkaliphilic halophile, *Thioalkalivibrio* (50). It is important to note that these halophiles were isolated from hypersaline and soda lakes (50–52) in which SCN^−^ is not available, at least not as a dominant sulfur-containing compound. In contrast, bioreactor receiving coke-oven wastewater provides a unique neutrophilic and halophilic environment in which SCN^−^ is abundantly available. The isolation of new candidate neutrophilic halophiles capable of SCN^−^ degradation may be anticipated from this SCN^−^-rich, halophilic environment. After the isolation of a new candidate, optimal cultivation conditions (including pH, temperature, and salinity ranges) need to be investigated when the isolate is used for SCN^−^ removal from wastewater in the activated sludge process. Furthermore, isolates obtained in the form of planktonic cultures are difficult to directly inoculate into the activated sludge process because the biomass with poor settleability is easily washed out as effluent (57). In this case, planktonic cells may be immobilized in gel media (*e.g*., polyvinyl alcohol, sodium alginate, and polyethylene glycol media) (1); however, the gel immobilization of SCN^−^-degrading bacteria and the performance of SCN^−^ degradation have not yet been investigated.

All known SCN^−^-degrading halophiles chemoautotrophically degrade SCN^−^ to NH_4_^+^ and sulfate, and two distinct metabolic pathways are involved in SCN^−^ degradation; *i.e*., 1) thiocyanate hydrolase (SCNase) and 2) thiocyanate dehydrogenase (TcDH) pathways. *Thioalkalivibrio thiocyanodenitrificans* ARhD1 and *Thiohalophilus thiocyanoxidans* HRhD2 degrade SCN^−^ with SCNase to carbonyl sulfide (COS) and NH_4_^+^ (20, 25). COS is hydrolyzed to hydrogen sulfide by carbonyl sulfide hydrolase (37), and the hydrogen sulfide generated enters sulfur oxidation pathways. On the other hand, *Thioalkalivibrio paradoxus* ARh1, *Thioalkalivibrio thiocyanoxidans* ARh2, and *Thiohalobacter thiocyanaticus* degrade SCN^−^ with TcDH to sulfur (S_0_) and CNO^−^ (Tsallagov, S.I *et al*. 2015. Abstracts for the EMBO Workshop on Microbial Sulfur Metabolism. p. 104, Helsingør), and the S_0_ and CNO^−^ formed are sequentially converted to sulfate and NH_4_^+^ (6, 61). Therefore, a crucial difference between the SCNase and TcDH pathways is the first reaction in the sequence (52). The TcDH pathway has been examined using the alkaliphilic halophile *Thioalkalivibrio* (hereafter designated as the alkaliphilic TcDH pathway), whereas recent genomic studies suggested that neutrophilic halophiles (*e.g*., *Thiohalobacter thiocyanaticus* Hrh1, GenBank assembly accession GCA_003932505.1) also employ the TcDH pathway (*i.e*., the neutrophilic TcDH pathway) (61). However, the molecular mechanisms underlying the neutrophilic TcDH pathway remain largely unexplored. As described below, we obtained a highly enriched culture of a neutrophilic halophile capable of SCN^−^ degradation, and elucidated its whole genome sequence and proteome. The gene encoding TcDH was located in the genome and abundant in the proteome; this result prompted us to investigate the molecular mechanisms involved in the neutrophilic TcDH pathway.

Therefore, the present study aimed 1) to obtain a neutrophilic halophile capable of SCN^−^ degradation from a SCN^−^-rich halophilic environment, 2) to examine optimal cultivation conditions and the performance of SCN^−^ degradation by the neutrophilic halophile and also the gel-immobilized biomass, and 3) to investigate the molecular mechanisms underlying the neutrophilic TcDH pathway. A neutrophilic halophile capable of SCN^−^ degradation was enriched from a bioreactor receiving synthetic coke-oven wastewater diluted with seawater. The enrichment of individual bacterial species during subculturing was examined by the amplicon sequencing of partial 16S rRNA gene sequences. A highly enriched culture of *Thiohalobacter sp*. strain FOKN1 was obtained by serial dilution, in which *Thiohalobacter sp*. strain FOKN1 16S rRNA gene reads accounted for 98.4% of all reads. SCN^−^ degradation by and the optimal cultivation conditions of *Thiohalobacter sp*. strain FOKN1 were examined, including 1) SCN^−^ degradation-dependent growth, 2) optimal pH, temperature, and salinity ranges for SCN^−^ degradation, and 3) the inhibition of SCN^−^ degradation by phenol and thiosulfate, common contaminants in coke-oven wastewater. We also examined the performance of SCN^−^ degradation after gel immobilization into polyvinyl alcohol (PVA)-alginate gel beads because *Thiohalobacter sp*. strain FOKN1 proliferated in the form of planktonic cells. To clarify the molecular mechanisms underlying the neutrophilic TcDH pathway, (meta)genomic and proteomic analyses of the enrichment culture were performed in order to elucidate the whole genome sequence and proteome of *Thiohalobacter sp*. strain FOKN1, and these analyses were performed using a PacBio RSII sequencer and by tandem mass spectrometry, respectively. The molecular mechanisms underlying the neutrophilic TcDH pathway were investigated based on the protein expression of core genes involved in SCN^−^ degradation and sulfur oxidation. We describe the molecular mechanisms involved in the neutrophilic TcDH pathway, and the results obtained will advance our understanding of molecular mechanisms contributing to microbial SCN^−^ degradation.

## Materials and Methods

### Enrichment and cultivation of Thiohalobacter sp. *strain* FOKN1

A SCN^−^-degrading culture was obtained by inoculating the biomass into an inorganic medium containing SCN^−^ (L^−1^: 3.44 mmol sodium thiocyanate, 30.8 mmol ammonium chloride, 7.74 mmol sodium hydrogen carbonate, 0.206 mmol disodium phosphate, 0.001% [w/v] yeast extract, and 60% [v/v] seawater, pH 7.0–7.5). The conductivity of the prepared inorganic medium was 3.46 S m^−1^, as assessed using the electrical conductivity meter CM-21P (DKK-TOA, Tokyo, Japan), corresponding to 0.36 M sodium chloride. The biomass was collected from a lab-scale moving bed biofilm reactor (volume; 3.5 L) under neutrophilic (pH 7.5) and halophilic (salinity; 0.36 M sodium chloride) conditions for the degradation of phenol, SCN^−^, and thiosulfate from synthetic coke-oven wastewater. The reactor was operated at 30°C with SCN^−^ (3.95 mM), NH_4_^+^ (30.8 mM), phenol (1.36 mM), and thiosulfate (2.25 mM) at loading rates of 3.5, 4.7, 7.0, and 10.5 L d^−1^, respectively. Cubic-shaped polyurethane sponges (10×10×10 mm) and activated sludge from real coke-oven wastewater were used as carrier and seeding sludges, respectively. After 56 d of continuous operation, the reactor degraded SCN^−^ at a rate of 0.7 kg SCN^−^ m^−3^ d^−1^, and activated sludge squeezed from the sponge media was used as the inoculum. After the inoculation of the biomass (1% [v/v]), the culture was aerobically incubated in a 500-mL Erlenmeyer flask containing 200 mL of inorganic medium. The incubation was performed in the dark at 30°C with shaking at 80 rpm. After the depletion of SCN^−^, 2% of the culture was transferred into fresh medium, and the incubation was repeated. After repeating this subculture eight times, a highly enriched culture of *Thiohalobacter sp*. strain FOKN1 was obtained by serial dilution. The enrichment of *Thiohalobacter sp*. strain FOKN1 was examined by light microscopy and microbial community structure analyses using the direct sequencing and amplicon sequencing of the 16S rRNA gene. The enrichment culture obtained was used for activity tests, genome and proteome analyses, and gel immobilization.

*Thiohalobacter sp*. strain FOKN1 was cultivated in 2.5-L glass bottles containing 2 L of inorganic medium. Oxygen was supplied by aeration at a flow rate of 0.5 L min^−1^. The concentration of SCN^−^ in the culture was monitored over time, and bacterial cells, corresponding to the late-exponential growth phase, were harvested by centrifugation (18,000×*g*, 10 min) when SCN^−^ was exhausted. The cell pellet was used for DNA and protein extraction and immobilization in PVA-alginate gel beads (each 180 mg-wet).

### Activity tests

The enrichment culture of *Thiohalobacter sp*. strain FOKN1 was suspended in inorganic medium at a concentration of 10^6^ cells mL^−1^. The cell suspension (2.5 mL) was dispensed into 15-mL glass serum vials (Nichiden-Rika glass, Tokyo, Japan) and incubated at 30°C with shaking at 80 rpm. Thiosulfate (Na_2_S_2_O_3_·5H_2_O) was added at a final concentration of 200 mg L^−1^ instead of SCN^−^ when thiosulfate degradation was examined. The influence of phenol and thiosulfate on SCN^−^ degradation was examined by supplementing medium with phenol or Na_2_S_2_O_3_·5H_2_O at final concentrations of 0 to 100 mg L^−1^ and 0 to 250 mg L^−1^, respectively. pH, temperature, and salinity varied across ranges of pH 6 to 11, 4 to 65°C, and 0 to 0.6 M sodium chloride, respectively. pH in the culture was adjusted by adding buffer at a final concentration of 25 mM. The buffers used were 2-morpholinoethanesulfonic acid, monohydrate (MES) for pH 6, 2-(4-[2-hydroxyethyl]-1-piperazinyl) ethanesulfonic acid (HEPES) for pH 7 to 8.8, N-cyclohexyl-2-aminoethanesulfonic acid (CHES) for pH 9, and N-cyclohexyl-3-aminopropanesulfonic acid (CAPS) for pH 10 to 11. Salinity was adjusted by changing the proportion of seawater in the medium. In the chemical analysis, a portion of the culture was collected aseptically, filtered through a 0.45-μm pore PDVF filter (Dikma Technologies, Foothill Ranch, CA, USA), and immediately subjected to analyses in order to minimize the influence of the air oxidation of sulfur compounds.

### Immobilization in PVA-alginate gel beads and SCN^−^ degradation

The immobilization of *Thiohalobacter sp*. strain FOKN1 cells in PVA-alginate gel beads was accomplished as previously described by Ali *et al*. (1). Briefly, cell pellets (180 mg-wet) were resuspended in 25 mL of inorganic medium, which was mixed with an equal volume of PVA-alginate (FUJIFILM Wako Pure Chemical, Osaka, Japan) solution (6 and 2% [w/v], respectively). The gel solution was dropped using a disposable 50-mL plastic syringe (Terumo, Tokyo, Japan) into a 4% (w/v) CaCl_2_ solution to form gel beads (*ca*. 3 to 5 mm in diameter). After an overnight incubation at 20°C, gel beads were washed with fresh inorganic medium. The gel beads obtained (volume; 20 mL) were inoculated into a 500-mL Erlenmeyer flask containing 100 mL of inorganic media, and incubated at 30°C in the dark with shaking at 80 rpm. The liquid culture was aseptically collected and filtered with a 0.45-μm pore PDVF filter to assess SCN^−^ concentrations.

### Chemical analysis

SCN^−^ concentrations were assessed colorimetrically using iron(III) nitrate, as previously described (53). Samples were mixed with a nitric acid and iron(III) nitrate solution, and absorbance at 460 nm was measured using a 96-well microplate reader (Sunrise Rainbow, Tecan Japan, Kawasaki, Japan).

NH_4_^+^ concentrations were evaluated colorimetrically using the indophenol method as described elsewhere. Samples were mixed with phenol and hypochlorous acid solutions, and absorbance was measured at 635 nm.

Cyanate concentrations were assessed as NH_4_^+^ concentrations after acidification using 6M HCl and boiling as previously described (49). The NH_4_^+^ concentrations of samples with and without acidification and boiling were measured, and differences in NH_4_^+^ concentrations were defined as cyanate concentrations.

When the concentrations of sulfur compounds (*i.e*., sulfate, sulfite, sulfide, thiosulfate, and zero-valent sulfur) were assessed, the dilution of liquid samples and preparation of reagents and standards were conducted using anoxic distilled water to prevent the oxidation of sulfur compounds. Distilled water was dispensed into sealable 100-mL glass vials and purged with argon gas for 30 min to prepare anoxic distilled water.

Sulfate concentrations were measured using turbidity after the addition of barium chloride as previously described (4). One hundred microliters of a liquid sample was mixed with 40 μL of buffer solution (30 g L^−1^ MgCl_2_·6H_2_O, 5 g L^−1^ CH_3_COONa·3H_2_O, 1 g L^−1^ KNO_3_, and 20 mL L^−1^ acetic acid) and 20 μL of barium chloride solution (15 g L^−1^) in a 96-well plate (Becton Dickinson, Franklin Lakes, NJ, USA), and turbidity was measured at 420 nm.

Sulfite and thiosulfate concentrations were assessed colorimetrically using 2,2′-dithiobis (5-nitropyridine) (DNTP) as previously described (38). A liquid sample (250 μL) was mixed with 10 mM DNTP (12.5 μL) and 161 mM sodium acetate solution (12.5 μL). The sample was loaded onto a spin column packed with Sep-Pak C_18_ medium (bed volume; 500 μL). The column was preequilibrated with methanol and then distilled water. The sample was filtrated using the preequilibrated column by centrifugation at 22×*g* for 1 min. The filtrate was loaded onto a second spin column packed with Sep-Pak C_18_ medium. The second column was preequilibrated with methanol, distilled water, and 10 mM tetrabutylammonium hydrogen sulfate (TBAHS) solution. Thiosulfate (*i.e*., thiosulfate-DNTP complex) was bound in the first column, and was eluted with 20% methanol solution. Sulfite (sulfite-DNTP complex) was bound in the second column, washed with 20% methanol-7.5 mM TBAHS solution, and eluted with 40% methanol-7.5 mM TBAHS solution. The absorbance of eluents was evaluated at 400 nm. Standard curves for the quantification of sulfite and thiosulfate concentrations were produced using serial dilutions of pure Na_2_SO_3_ and Na_2_S_2_O_3_·5H_2_O solutions, respectively.

Sulfide concentrations were assessed colorimetrically using methylene blue as previously described (60). A liquid sample was mixed with 3.7 mM N, N-dimethyl-1,4-phenylenediamine, 0.02 M FeCl_3_, and 3 M (NH_4_)_2_HPO_4_, and absorbance at 670 nm was measured.

Zero-valent sulfur concentrations were measured using high-performance liquid chromatography (HPLC) as previously described by Zopfi *et al*. (61). Briefly, zero-valent sulfur compounds in a liquid sample were fixed with ZnCl_2_ as elemental sulfur. Elemental sulfur was dissolved in HPLC-grade methanol and analyzed as S_6_ by HPLC using a LC-10AD HPLC pump (Shimadzu, Kyoto, Japan), UV-VIS detector SPD-10A (Shimadzu), and CAPCELL PAK C_18_ ODS column (4.8×250 mm) (OSAKA SODA, Osaka, Japan). The standard curve for quantification was prepared using sulfur powder (FUJIFILM Wako Pure Chemical).

### Microscopy

Epifluorescence and light microscopy employed an Olympus BX60 equipped with CCD DP71 (Olympus, Tokyo, Japan). Regarding total cell counts, cells in cultures were stained using SYBR Green I solution (Life Technologies, Carlsbad, CA, USA) for 10 min, and filtered using a 0.2-μm-pore polycarbonate membrane (Merck, Burlington, MA, USA). Under the microscope, at least ten randomly selected fields containing more than 50 cells per field were used for quantification; the numbers of cells were counted manually.

### Sequencing of the 16S rRNA gene

DNA was extracted from cell pellets of the enrichment culture using the DNeasy PowerSoil Kit (Qiagen Japan, Tokyo, Japan) according to the manufacturer’s protocols. DNA concentrations were assessed using Qubit dsDNA BR assay kits and a Qubit 3.0 fluorospectrometer (Thermo Fisher Scientific, Waltham, MA, USA). Extracted DNA was processed as follows for direct and amplicon sequencing of the 16S rRNA gene. In direct sequencing, the PCR amplification of a partial 16S rRNA gene sequence and Sanger sequencing were performed as previously described (39) with slight modifications. PCR amplification used the oligonucleotide primers 515F (5′-GTGCCAGCMGCCGCGGTA-3′)–806R (5′-GGACTACHVGGGTWTCTAAT-3′) (8) for a prokaryotic 16S rRNA gene. The PCR mixture had a volume of 100 μL and contained 4–100 ng of DNA, oligonucleotide primers (0.3 μM each), dNTPs (200 μM), 1× PCR buffer, and PrimeSTAR GXL Taq (0.025 U μL^−1^) (Takara Bio, Kusatsu, Japan). Cycling conditions were as follows: 40 cycles at 98°C for 10 s, followed by 50°C for 15 s, then 72°C for 45 s; and finally 72°C for 5 min. PCR products were purified using FastGene Gel/PCR Extraction Kits (Nippon Genetics, Tokyo, Japan). The direct sequencing of the purified PCR amplicon was performed using the 515F and 806R primers as sequencing primers. The nucleotide sequences identified were assembled manually and subjected to a blastn search using the nr database as a reference (National Center for Biotechnology Information, NCBI).

Amplicon sequencing of the 16S rRNA gene was performed as previously described (19). PCR amplification used the above-described oligonucleotide primers 515F and 806R containing Illumina tag sequences at the 5′ ends. The amplicon was purified and used in the preparation of a library by means of MiSeq Reagent Kits v2 nano (Illumina, San Diego, CA, USA) for sequencing on Illumina MiSeq (Illumina). Amplicon library concentrations were measured using BioAnalyzer DNA 1000 (Agilent Technologies, Santa Clara, CA, USA). The quality of the sequencing analysis was verified by examining the PhiX library prepared from the PhiX spike-in control (Illumina). Sequence reads with a low quality score (Phred quality score ≤30) were eliminated using the fastx trimmer tool, and paired-end sequence reads were then assembled in the paired-end assembler for the Illumina sequence software package (PANDAseq) (33). Sequence reads with ≥97% similarity were grouped into an operational taxonomic unit (OTU) by the UCLUST algorithm (14). The phylogenetic affiliations of the OTUs were identified using a blastn search against reference sequences in the Greengenes database version 13_5 (13) and in the nr database provided by the NCBI.

### Genome sequencing, annotation, and comparative genome analysis

Genomic DNA was extracted using Qiagen DNeasy Mini Kits (Qiagen) and subjected to sequencing on a PacBio RSII sequencer and SMART cell 8PAC V3, with DNA Polymerase Binding Kit P6 (Pacific Biosciences, Menlo Park, CA, USA). Sequence reads were assembled using HGAP3 software (9), and gene prediction and annotation were performed via the MiGap pipeline (56) as previously described (40). The MetaGeneAnnotator (36) and Glimmer version 2.10 (12), tRNAScan-SE version 1.23 (31), and blastn (3) software applications were used to predict gene-coding sequences (CDSs), tRNA, and rRNA, respectively. The functional annotation of CDSs was performed by a blastp search against clusters of the orthologous groups of the protein (COG) database and by using the KEGG Automatic Annotation Server (KAAS). The threshold *e*-value of the blastp search for the annotation of COG was 10^−6^, and the protein sequence file available at the NCBI (ftp://ftp.ncbi.nih.gov/pub/COG/COG/myva) was used as a reference database. KAAS (35) performed a blast search using the gene dataset for prokaryotes as a reference, and a bi-directional best hit method was used to assign orthologs. The completeness of the genome sequence was calculated by the gene set assessment of Benchmarking Universal Single-Copy Orthologs (BUSCO) software version 3 using the gammaproteobacteria_odh9 dataset as the lineage dataset (48). BUSCO software examines the presence/absence of universal single-copy orthologs conserved among gammaproteobacterial genomes. Average nucleotide identity (ANI) values (45) were calculated using the OrthoANI and USEARCH tools (14, 29). Signal peptide cleavage sites and transmembrane helices were predicted using the Signal 4.0 server for Gram-negative bacteria with default D-cut-off values (43) and TMHMM Server 2.0 (27), respectively. The alignment of nucleic acid and protein sequences was performed by ClustalW 1.83 (58). Gap opening and extension penalties in a pairwise alignment were set to 10 and 0.1, respectively.

The phylogeny of *Thiohalobacter sp*. strain FOKN1 was examined using the full-length 16S rRNA gene sequence (1,504 bp) from the genome as a query and the nr database (NCBI) as a reference. A phylogenetic tree was constructed by aligning 16S rRNA gene sequences using ClustalW 1.83 (positions 100–1,386 in the *Escherichia coli* 16S rRNA gene), and by calculating the tree in MEGA 7.0.26 using maximum likelihood (Jones-Taylor-Thornton model with 250 bootstrap iterations), neighbor joining (Poisson model with 500 bootstrap iterations), and maximum parsimony methods (search algorithm: close neighbor interchange on random trees with 250 bootstrap iterations) (28) by means of the 16S rRNA gene sequence of *Aquifex pyrophilus* (accession number M83548) as an outgroup.

### Proteomic analysis

The proteome of *Thiohalobacter sp*. strain FOKN1 cells was examined by one-dimensional sodium dodecyl sulfate polyacrylamide gel electrophoresis (SDS-PAGE) and nanoscale liquid chromatography with tandem mass spectrometry (nanoLC-MS/MS). Bacterial cells in the enrichment culture were harvested for the proteomic analysis when the SCN^−^ concentration (initial concentration: 3.44 mM) decreased below 0.1 mM (corresponding to the late-exponential growth phase). The harvested cell pellet was subjected to protein extraction, separation, and identification by one-dimensional SDS-PAGE and nanoLC-MS/MS as previously described (2). Briefly, cell pellets of the FOKN1 culture (38 mg-wet) were suspended in 250 μL of extraction buffer A containing 3-([3-cholamidopropyl] dimethylammonio)-1-propanesulfonate (CHAPS) (4% [w/v]), Triton X-100 (2% [w/v]), urea (7 M), thiourea (2 M), and dithiothreitol (60 mM), and were then disrupted by sonication with Sonifier 250 (output strength 30, 1 min, and 3 cycles) (Branson Ultrasonics, Emerson Japan, Kanagawa, Japan). After a 1-h incubation on ice, the extract was centrifuged at 20,630×*g* for 4 min, and the supernatant was recovered. The remaining pellet was resuspended in 250 μL of extraction buffer B containing SDS (4% [w/v]), glycerol (20% [v/v]), Tris-HCl (0.125 M, pH 8.4), and dithiothreitol (20 mM) to extract proteins from the remaining biomass. After boiling for 15 min and centrifugation (20,630×*g* at 20°C for 1 h), the supernatant was recovered as soluble protein fraction B. Proteins in soluble protein fractions A and B were separated on an SDS-containing polyacrylamide gel (Mini-PROTEAN TGX 4–15% gel) (Bio-Rad, Hercules, CA, USA), and stained with the CBB protein safe stain (Takara Bio). Gel lanes (6 cm in length) were cut into 24–26 gel slices, which were destained with acetonitrile and subjected to a nanoLC-MS/MS analysis after in-gel tryptic digestion (23). Briefly, nanoLC-MS/MS analyses were performed using an LTQ ion-trap MS (Thermo Fisher Scientific, Yokohama, Japan) equipped with the multidimensional HPLC paradigm MS2 HPLC (AMR, Tokyo, Japan), a nano-spray electrospray ionization device (Michrom Bioresources, Auburn, CA, USA), and L-column2 ODS column packed with C_18_ modified silica particles (Chemicals Evaluation & Research, Tokyo, Japan). Peptide spectra were obtained in a mass range of *m/z* 450–1800, and MS/MS spectra were acquired in the data-dependent scan mode. After a full spectrum scan, 1 MS/MS spectrum of the single most intense peaks was also collected. Dynamic exclusion features were set as follows: a repeat count of 1 within 30 s, an exclusion duration of 180 s, and an exclusion list size of 50. The MS/MS data obtained were analyzed using MASCOT search software, version 2.3.01 (42). The amino acid sequences of 3,026 CDSs, located in the FOKN1 genome, were used as the reference database, and search parameters were as follows: tryptic digest with a maximum of two missed cleavage sites; fixed modifications, carbamidomethyl cysteine; variable modifications, methionine oxidation; peptide masses, monoisotopic, positive charge (+1, +2, +3) of the peptide; and mass tolerance of 1.2 Da for the precursor ion and 0.8 Da for product ions. Proteins with two or more peptides were identified in the present study. An automatic decoy search was performed against a randomized database with a default significance threshold (*P*<0.05) to examine false-positive identifications.

The abundance of identified proteins among all proteins was examined by a semiquantitative method (21, 47) using the exponentially modified protein abundance index (emPAI) and protein content index (PCI). emPAI values were provided by MASCOT software, which compares the number of observed unique parent ions per protein with the number of observable peptides per protein. The PCI value represents the molar percentages of particular proteins among all detected proteins, as calculated by Shinoda *et al*. (47):

PCI(%)=emPAI÷Σ(emPAI)×100

where ∑(*emPAI*) is the summation of the emPAI values for all of the identified proteins.

### Nucleotide sequence accession numbers

The *Thiohalobacter sp*. strain FOKN1 genome was deposited in the DDBJ nucleic acid sequence database under the accession number AP018052. Sequence data obtained from the amplicon sequencing of the 16S rRNA gene were deposited in the DDBJ nucleotide sequence database under accession number DRA007056.

## Results

### Enrichment of Thiohalobacter sp. *strain* FOKN1 from activated sludge

The biomass collected from a lab-scale moving bed biofilm reactor receiving synthetic coke-oven wastewater was inoculated into inorganic medium containing 3.44 mM SCN^−^, and SCN^−^-degrading microorganisms were enriched by repeating the inoculation of the culture into fresh medium. To monitor the enrichment of target bacterial species during the cultivation, amplicon sequencing of a partial 16S rRNA gene sequence was performed. As shown in Fig. S1, bacteria affiliated with the uncultured family *Chromatiales* and the family *Idiomarinaceae* (both belonging to the class *Gammaproteobacteria*) were enriched during subculturing. The uncultured family *Chromatiales* includes the genus *Thiohalobacter*, and the sequence reads of the *Thiohalobacter* 16S rRNA gene accounted for 1.4 and 26.3% of total reads obtained from the inoculum and 8^th^ subculture, respectively. Since colony formation was not observed on any tested agar media (inorganic medium used for the above enrichment, 1/10-fold Luria-Bertani broth, R2A, and the DSM1058e medium previously used for the cultivation of the SCN^−^-degrading neutrophilic halophile *Thiohalobacter thiocyanaticus*) (52), a serial dilution (10^−3^ to 10^−9^-fold dilution) was performed to isolate cells in the 8^th^ subculture. The culture exhibiting SCN^−^ degradation activity and containing morphologically uniform cells (*ca*. 1 μm, rod-shaped cells) was obtained after a 10^−6^-fold dilution, and the serial dilution was repeated twice. In the enrichment culture ultimately obtained, the sequence reads of the *Thiohalobacter* 16S rRNA gene accounted for 98.4% of total reads (Fig. S1). PCR amplification of the prokaryotic 16S rRNA gene and subsequent direct sequencing of the PCR amplicon were conducted using total genomic DNA extracted from the enrichment culture. Single nucleotide polymorphisms were not found in the 16S rRNA sequence. The outcomes of the above amplicon and direct sequencing indicated that a SCN^−^-degrading neutrophilic halophile affiliated with the genus *Thiohalobacter* was highly enriched in the present study, and was herein designated as *Thiohalobacter sp*. strain FOKN1.

### SCN^−^ degradation and optimal cultivation conditions of Thiohalobacter sp. *strain* FOKN1

**Fig. 1:**
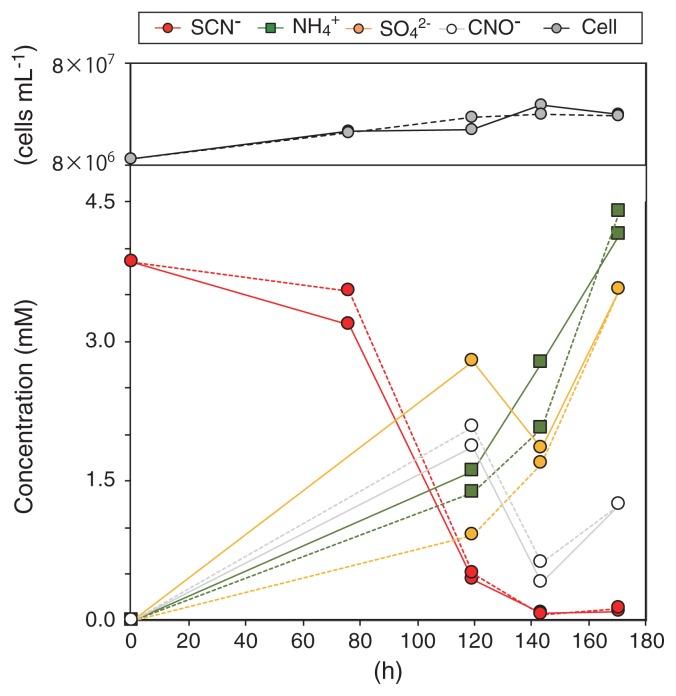
Thiocyanate (SCN^−^) degradation by a highly enriched culture of *Thiohalobacter sp*. strain FOKN1. The enrichment culture was aerobically cultivated for 170 h in the presence of SCN^−^. Incubations were performed in duplicate, and concentrations measured from each replicate are shown with solid and dotted lines, respectively.

**Fig. 2:**
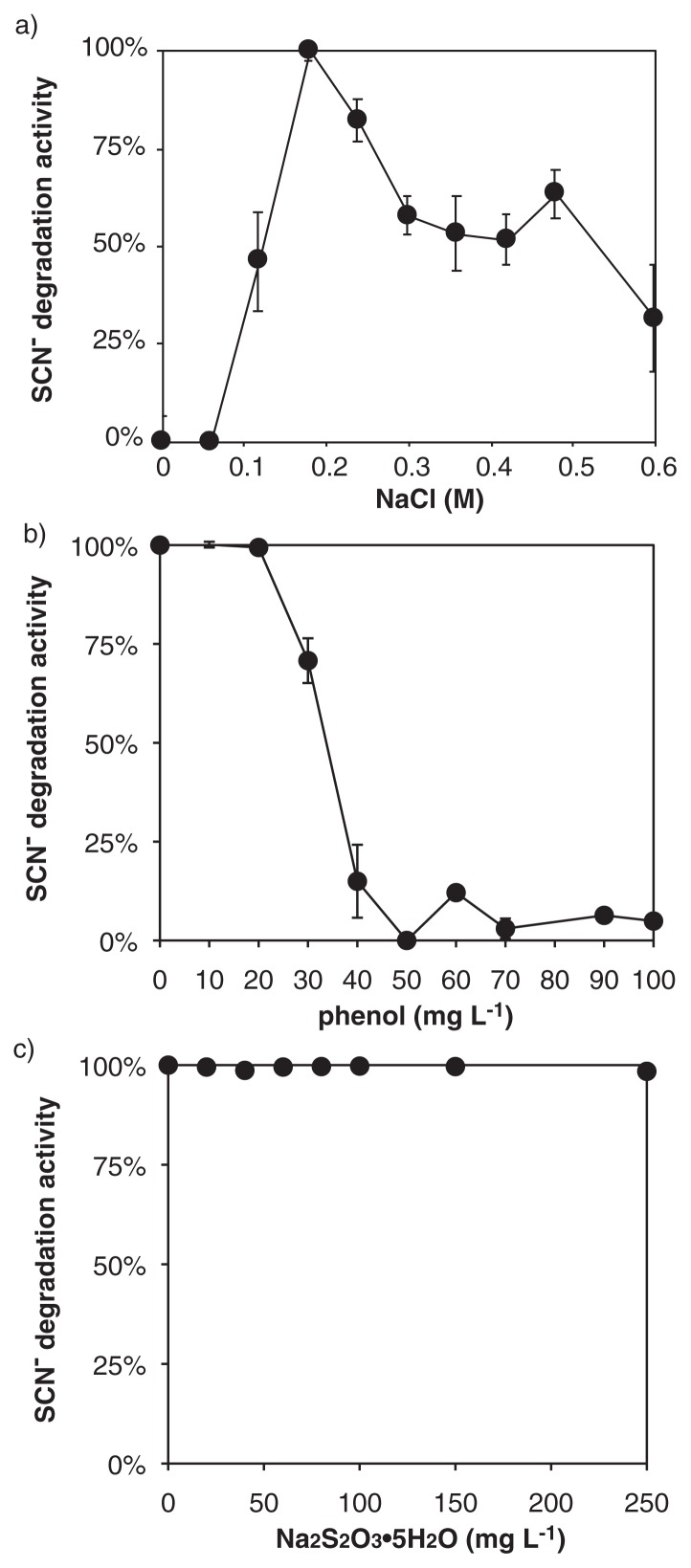
Influence of salinity (a), phenol (b), and thiosulfate (c) on thiocyanate (SCN^−^) degradation. a) Salinity was adjusted by changing the proportion of seawater in the medium. SCN^−^ degradation at 0.18 M sodium chloride was 23 μM h^−1^, and relative activities are shown in the graph. b and c) Phenol or thiosulfate was supplemented into inorganic medium containing SCN^−^. SCN^−^ degradation without phenol or thiosulfate was 43 μM h^−1^, and relative activities are shown in the graph. Error bars represent the range of standard deviations obtained from triplicate incubations.

The enrichment culture obtained degraded 3.38 mM SCN^−^ within 140 h (corresponding to a degradation rate of 24 μM h^−1^), and quantitatively produced NH_4_^+^ and SO_4_^2−^ (Fig. 1). CNO^−^ accumulated after 120 h of incubation, whereas sulfite, sulfide, zero-valent sulfur, and thiosulfate did not. The growth of bacterial cells occurred concurrently with SCN^−^ degradation, and the growth rate calculated from the increase in cell numbers during 143 h of incubation was 0.19 d^−1^ (corresponding to 0.0078 h^−1^). SCN^−^ degradation occurred at a pH between 6 to 11, at temperatures between 4 to 55°C, and at salinity between 0.12 to 0.6 M sodium chloride. When salinity was lower than 0.12 M sodium chloride, the activity of SCN^−^ degradation disappeared (Fig. 2A), indicating that this bacterium is a mild halophile (32). Maximum activity was observed at pH 8.8, 37°C, and 0.18 M sodium chloride. SCN^−^ degradation was inhibited by the addition of phenol into the culture at the final concentration of >30 mg L^−1^, but not by that of thiosulfate (up to 250 mg L^−1^) (Fig. 2B and C). When the enrichment culture was incubated with the addition of 1.75 mM thiosulfate instead of SCN^−^, thiosulfate was degraded within 61 h. The enrichment culture degraded SCN^−^ with the reduction of NO_2_^−^ to N_2_ and N_2_O when the culture was incubated anoxically (*i.e*., the addition of 1 mM NO_2_^−^), while NO_2_^−^ reduction did not support the growth of *Thiohalobacter sp*. strain FOKN1 (See Supplementary text for details).

**Fig. 3:**
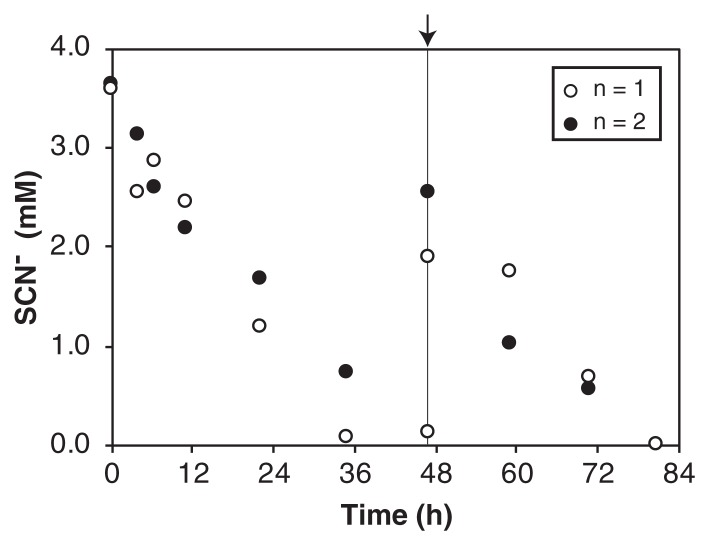
Thiocyanate (SCN^−^) degradation using the gel-immobilized *Thiohalobacter sp*. strain FOKN1 biomass. The biomass of *Thiohalobacter sp*. strain FOKN1 was immobilized on polyvinyl alcohol (PVA)-alginate gel (6 and 2%, w/v, respectively) beads with diameters of 3 to 5 mm. Gel beads were aerobically incubated in duplicate vials, and SCN^−^ concentrations in cultures were measured. After 47 h of incubation, a stock solution of SCN^−^ was added as a spike.

SCN^−^ degradation was examined after PVA-gel immobilization because *Thiohalobacter sp*. strain FOKN1 cells proliferated in the form of a planktonic culture and it was not possible to settle cells by gravitational sedimentation. The gel beads had excellent settleability and were separated by gravitational sedimentation within 1 min. Gel beads degraded SCN^−^ at a rate of 43 μM h^−1^ (Fig. 3), which was 1.8-fold higher than that observed in the FOKN1 culture (Fig. 1).

### Overview of the Thiohalobacter sp. *strain* FOKN1 genome and proteome

**Fig. 4:**
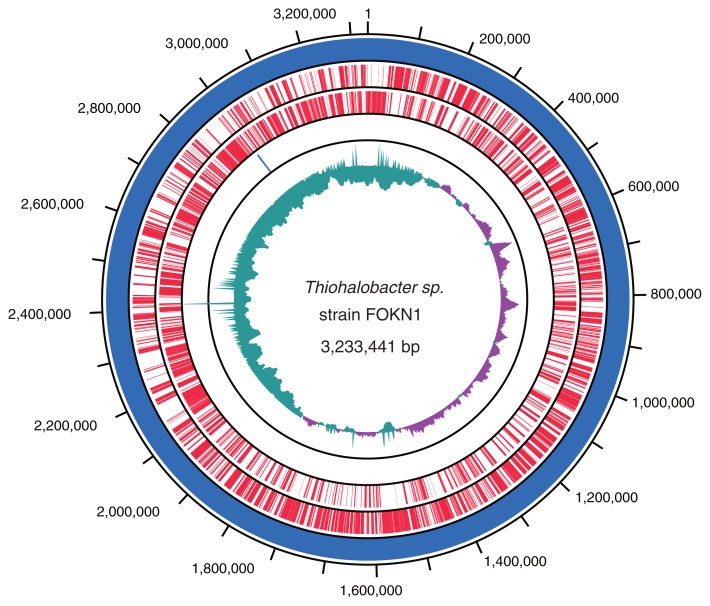
A circular map of the *Thiohalobacter sp*. strain FOKN1 genome. The outermost circle and second circle represent forward and reverse gene-coding regions (CDSs), respectively, highlighted in red. The third outermost circle represents the *rrn* operon (blue). The innermost circle represents the G+C skew; the blue and red colors correspond to regions at which the skew is less or higher than 0.05, respectively.

A 3.23-Mb circular genome of *Thiohalobacter sp*. strain FOKN1 was assembled using sequence reads obtained from the PacBio RSII sequencer as previously reported (41). The *Thiohalobacter sp*. strain FOKN1 genome had 3,026 genes, 1 *rrn* operon, and 45 tRNA genes, and the functional annotation of genes located in the genome was performed by a blastp search using the COG database and KAAS (Fig. 4 and Table S1). Among the 3,026 genes identified, 1,214 were conserved among the *Thiohalobacter sp*. strain FOKN1 and alkaliphilic halophile (*i.e*., *Thioalkalivibrio*) genomes (Fig. S2). In addition to *Thiohalobacter sp*. strain FOKN1, another *Thiohalobacter* genome was recently elucidated from *Thiohalobacter thiocyanaticus* Hrh1 (GenBank assembly accession; GCA_003932505.1), which is a 3.30-Mb genome composed of two contigs. The completeness of genome sequences was examined using BUSCO software (48), and the completeness of the FOKN1 and Hrh1 genomes was calculated to be 96.0 and 89.1%, respectively, based on the presence of universal single-copy orthologs (Table S2). ANI values were calculated using known SCN^−^-degrading bacterial genomes and shown in Table S3.

Protein expression was examined using one-dimensional SDS-PAGE and nanoLC-MS/MS analyses. *Thiohalobacter sp*. strain FOKN1 cells were harvested at the late-exponential growth phase, and total proteins were subjected to SDS-PAGE and nanoLC-MS/MS analyses. A total of 914 proteins were identified (false discovery rate at the identity threshold: 0.0788) from a set of peptide fragments obtained from the nanoLC-MS/MS analysis (Table S4). The abundance of the identified proteins among total proteins was examined by a semiquantitative method with emPAI and PCI (47, See also Materials and Methods), and the abundance of identified proteins classified into the COG functional categories was shown in Fig. S3.

### Phylogeny and environmental distribution of Thiohalobacter sp. *strain* FOKN1

The phylogeny and environmental distribution of *Thiohalobacter sp*. strain FOKN1 were investigated using the full-length 16S rRNA gene sequence located in the assembled genome. A blastn search against the nr database (NCBI) revealed that the 16S rRNA gene sequence of *Thiohalobacter sp*. strain FOKN1 showed 99.1% similarity with that of the SCN^−^-degrading neutrophilic halophile *Thiohalobacter thiocyanaticus* HRh1 (52) (Fig. S4). This similarity was close to the threshold above which a bacterium may be assigned to the same bacterial species (*i.e*., 99% similarity) (10). On the other hand, the ANI value between the FOKN1 and HRh1 genomes was 86.1% (Table S3), which was lower than the threshold for species demarcation (95–96%) (45): therefore, the ANI value suggests that *Thiohalobacter sp*. strain FOKN1 is affiliated into a different species from *Thiohalobacter thiocyanaticus*. Further verification studies that include the analyses of membrane lipids and quinone species is required to conclude a taxonomic classification of *Thiohalobacter sp*. strain FOKN1. The blastn search with nr database revealed that the sequence of the *Thiohalobacter* 16S rRNA gene has not yet been obtained from environmental samples (accessed on 28^th^ June 2018).

### Molecular mechanisms underlying the neutrophilic TcDH pathway

**Fig. 5:**
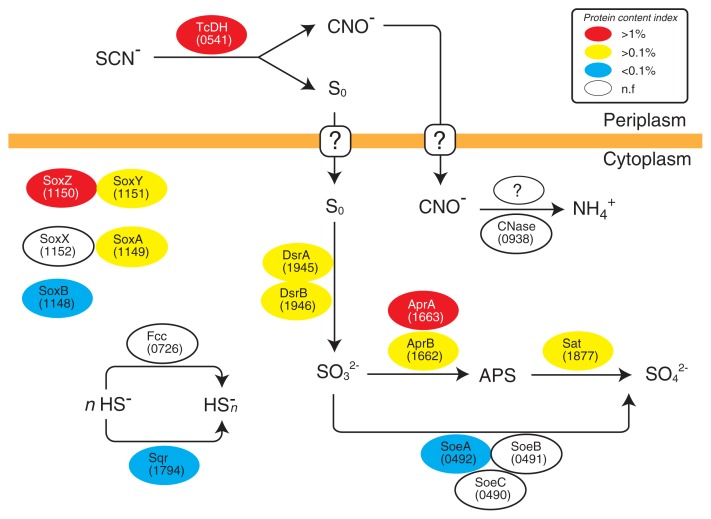
A proposed metabolic pathway responsible for thiocyanate (SCN^−^) degradation by *Thiohalobacter sp*. strain FOKN1. TcDH; thiocyanate dehydrogenase, CNase; cyanate lyase, DsrAB; dissimilatory sulfite reductase, Apa; adenosine-5′-phosphosulfate (APS) reductase, Sat; sulfate adenylyltransferase, Sox; sulfur-oxidizing protein, Fcc; flavocytochrome *c* sulfide dehydrogenase, Sqr; sulfide:quinone reductase, and Soe; sulfite oxidoreductase. Locus tag numbers are shown in parentheses. The abundance of proteins detected in the proteome is presented as the protein content index. n.f; not found in the proteome.

A set of core genes potentially involved in SCN^−^ degradation by *Thiohalobacter sp*. strain FOKN1 is summarized in Table 1. Based on genomic information and protein expression in *Thiohalobacter sp*. strain FOKN1, the metabolic pathway of SCN^−^ degradation and subsequent sulfur oxidation was examined (Fig. 5). The orthologue of the gene encoding TcDH (the FOKN1_0541 gene) was located in the *Thiohalobacter sp*. strain FOKN1 genome, and the FOKN1_0541 protein was abundant in the proteome (Fig. S3). In contrast, the gene encoding SCNase was not found in the genome, suggesting that *Thiohalobacter sp*. strain FOKN1 oxidizes SCN^−^ using TcDH, similar to *Thioalkalivibrio thiocyanoxidans* ARh2 (6). Limited information is currently available on the reaction substrate and product of SCN^−^ degradation by TcDH; therefore, TcDH purified from cell-free extracts of the FOKN1 culture were used to examine the reaction. However, isolated TcDH did not oxidize SCN^−^ (See Supplementary text). SCN^−^ degradation has not been observed, even from cell-free extracts, as previously described for *Thiohalobacter thiocyanaticus* HRh1 (52). *Thiohalobacter sp*. strain FOKN1 TcDH may degrade SCN^−^ to S_0_ and CNO^−^ in a similar manner to *Thioalkalivibrio paradoxus* ARh1 TcDH (Tsallagov, S.I *et al*. 2015. Abstracts for the EMBO Workshop on Microbial Sulfur Metabolism. p. 104, Helsingør), while the biochemistry of *Thiohalobacter* TcDH needs to be examined in more detail in order to demonstrate the above pathway. During SCN^−^ degradation, the sulfur atom of SCN^−^ is oxidized, and *Thioalkalivibrio thiocyanoxidans* TcDH transfers electrons to the flavocytochrome *c* protein (Fcc) (5). However, *Thiohalobacter sp*. strain FOKN1 Fcc (the FOKN1_726 protein) was not detected in the proteome, suggesting that an as-yet-unknown protein is involved in electron transfer in *Thiohalobacter sp*. strain FOKN1 cells.

*Thiohalobacter sp*. strain FOKN1 TcDH has the signal peptide sequence at the N-terminal region (Met_1_-Ala_31_), suggesting that TcDH is localized in the periplasmic space, and that SCN^−^ degradation occurs in the periplasmic space. However, several proteins potentially involved in S_0_ and CNO^−^ oxidation do not have signal peptide sequences (Table 1), and, thus, are localized in the cytoplasm, including DsrAB (dissimilatory sulfite reductase), AprAB (adenosine-5′-phosphosulfate reductase), Sat (dissimilatory ATP sulfurylase), and CNase (cyanate lyase). Transport across the cytoplasmic membrane is required for the further oxidation of S_0_ and CNO^−^. In the sulfur-oxidizing bacterium, *Allochromatium vinosum*, S_0_ is transported into the cytoplasm using a persulfide carrier molecule (*e.g*., glutathione amide persulfide) and unidentified membrane transporter (16). DsrAB, AprAB, and Sat were abundantly detected (Table 1), suggesting that S_0_ is oxidized to sulfite by Dsr (44), and then to sulfate via APS by Apr and Sat (34). The *Thiohalobacter sp*. strain FOKN1 genome also has the gene encoding SoeABC, which is involved in sulfite oxidation to sulfate (11). This enzyme was not found in the proteome, which supports the conclusion that oxidation via APS is important for the conversion of sulfite to sulfate. The gene encoding CNase (the FOKN1_0938 gene) is found in the *Thiohalobacter sp*. strain FOKN1 genome, whereas CNase was not found in the proteome.

## Discussion

A highly enriched culture in which the SCN^−^-degrading neutrophilic halophile, *Thiohalobacter sp*. strain FOKN1, accounted for 98.4%, as examined by 16S rRNA gene amplicon sequencing, was obtained from a bioreactor operated for the treatment of synthetic coke-oven wastewater. Although *Thiohalobacter sp*. strain FOKN1 was not purified by a traditional serial dilution method, the whole genome sequence and proteome of this bacterium were successfully elucidated by (meta)genomic and proteomic analyses. The enrichment of *Thiohalobacter sp*. strain FOKN1 from the SCN^−^-rich and moderately halophilic bioreactor was unexpected because a close relative, *Thiohalobacter thiocyanaticus* HRh1, was previously obtained from hypersaline lakes in Russia (52), environments in which SCN^−^ is not available as a dominant sulfur compound. *Thiohalobacter thiocyanaticus* HRh1 oxidizes reduced sulfur compounds, such as thiosulfate, and this metabolic versatility may enable this bacterium to survive in sulfur-rich halophilic environments. Since the sequence of the *Thiohalobacter* 16S rRNA gene has not yet been detected from other natural and man-made ecosystems, this bacterium has a well-defined, but as-yet-uncharacterized environmental niche.

Although the alkaliphilic TcDH pathway has been investigated using alkaliphilic halophile *Thioalkalivibrio* (5, 6), limited information is currently available on the molecular mechanisms involved in the neutrophilic TcDH pathway. Very recently, Tsallagov *et al*. (61) provided a genomic insight into the TcDH pathway of *Thiohalobacter thiocyanaticus* HRh1, and showed overexpression of TcDH in HRh1 cells during SCN^−^ degradation. The genome sequence and proteome of *Thiohalobacter sp*. strain FOKN1 provide an excellent opportunity to advance our understanding of the molecular mechanisms involved in the neutrophilic TcDH pathway. As shown in Fig. 1, the enrichment culture of *Thiohalobacter sp*. strain FOKN1 accumulated CNO^−^ during SCN^−^ degradation, suggesting that *Thiohalobacter sp*. strain FOKN1 degrades SCN^−^ using TcDH, which is supported by the results of our genomic and proteomic analyses (Fig. S3). The proteome of *Thiohalobacter sp*. strain FOKN1 suggests the involvement of several proteins during SCN^−^ degradation; TcDH, Dsr, Apr, and Sat (Table 1). The involvement of TcDH, Dsr, Apr, and Sat in the SCN^−^ degradation of the alkaliphilic halophile *Thioalkalivibrio* has been deduced along with the transcription of these genes (5, 6). and of a neutrophilic halophile *Thiohalobacter thiocyanaticus* HRh1 (61) has been deduced because those genes were located in the *Thioalkalivibrio* and *Thiohalobacter thiocyanaticus* HRh1 genomes. Thus, SCN^−^ degradation mediated by TcDH proceeds in the cells of neutrophilic and alkaliphilic halophiles in a similar manner. TcDH oxidizes SCN^−^ to CNO^−^ (Tsallagov, S.I *et al*. 2015. Abstracts for the EMBO Workshop on Microbial Sulfur Metabolism. p. 104, Helsingør), while controversy still surrounds the enzymes responsible for subsequent CNO^−^ oxidation. CNase (EC 4.2.1.104) is the only known enzyme for bacterial CNO^−^ oxidation (26); however, the gene encoding CNase is not common in the bacterial genomes of neutrophilic and alkaliphilic halophiles capable of SCN^−^ degradation. The *Thiohalobacter sp*. strain FOKN1 genome possesses the gene encoding CNase, whereas the FOKN1_0938 protein was not found in the proteome. The involvement of CNase in CNO^−^ metabolism by *Thiohalobacter sp*. strain FOKN1 remains unclear.

SoxYZA proteins were abundantly detected in the proteome of *Thiohalobacter sp*. strain FOKN1 (Table 1). Sox proteins are involved in thiosulfate oxidation (17), in which thiosulfate is initially oxidized by SoxXA and the sulfur atom is covalently bound to SoxYZ. SoxB then hydrolytically releases sulfur from SoxYZ (17), and the S_0_ released is oxidized. *Thiohalobacter sp*. strain FOKN1 Sox proteins may be functional because this bacterium is capable of thiosulfate oxidation as described above. The high abundance of SoxYZA in the proteome of FOKN1 cells suggests that the formation of thiosulfate during SCN^−^ degradation occurs; however, the accumulation of thiosulfate as a free intermediate was not detected (Fig. 1). Therefore, the mechanisms by which SoxYZA contributes to SCN^−^ degradation warrant further study.

The optimal cultivation conditions of *Thiohalobacter sp*. strain FOKN1 (*e.g*., pH and temperature) need to be investigated when we consider SCN^−^ removal from wastewater using the culture. Notably, phenol is a common contaminant of coke-oven wastewater, whereas its influence on SCN^−^ degradation by halophiles has not yet been investigated. As shown in Fig. 2B, phenol inhibited the SCN^−^ degradation of *Thiohalobacter sp*. strain FOKN1, and phenol concentrations need to be monitored and maintained at less than 30 mg L^−1^ during wastewater treatment. In addition to the influence of the inhibitory substrate, the settleability of the biomass in the culture is also critical when we consider the inoculation of the culture into the activated sludge process. PVA-alginate gel beads with high settleability and SCN^−^-degradation activity were successfully prepared in the present study, and this is the first example of SCN^−^-degradation using the gel-immobilized biomass. The gel immobilization technique extends the applicability of the SCN^−^-degrading culture for the treatment of SCN^−^-contaminated wastewater.

In summary, a highly enriched culture of *Thiohalobacter sp*. strain FOKN1 was obtained, and the physiology and molecular mechanisms of the neutrophilic TcDH pathway were investigated in detail. Further studies are needed to obtain a more complete understanding of SCN^−^ degradation in bioreactors. Activated sludge in the bioreactor contained a complex microbial community (Fig. S1), in which SCN^−^ degradation by microorganisms, other than *Thiohalobacter sp*. strain FOKN1, and interactions among microorganisms in the community (15) are highly expected. Kantor *et al*. (22) performed metagenomic and metaproteomic analyses of the biomass collected from a continuous-flow bioreactor fed with SCN^−^, and identified *Thiobacillus* sp. and *Afipia* bacterium as SCN^−^-degrading bacteria. These meta-omics analyses provide insights into the metabolic potential of microorganisms without the need for cultivation and isolation. Future studies using these molecular tools will promote a deeper understanding of SCN^−^ degradation in the bioreactor used in the present study.

## SUPPLEMENTARY MATERIAL







## Figures and Tables

**Table 1:** Summary of core genes potentially involved in thiocyanate (SCN) oxidation metabolism. Presence/absence of the signal peptide sequence (SP) and transmembrane helix (TMH) predicted using the SignalP and TMHMM servers were shown as “Y” or “N”, respectively. PCI; protein content index. n.f; not found in the proteome.

Product	locus_tag	SP	TMH	PCI (%)
TcDH, thiocyanate dehydrogenase	FOKN1_0541	Y	N	2.455%
CNase, cyanate lyase	FOKN1_0938	N	N	n.f
DsrA, dissimilatory sulfite reductase subunit A	FOKN1_1945	N	N	0.343%
DsrB, dissimilatory sulfite reductase subunit B	FOKN1_1946	N	N	0.380%
DsrE, dissimilatory sulfite reductase sulfur relay complex	FOKN1_1947	N	N	0.846%
DsrF, dissimilatory sulfite reductase; sulfur relay complex	FOKN1_1948	N	N	0.064%
DsrH, dissimilatory sulfite reductase; sulfur relay complex	FOKN1_1949	N	N	0.066%
DscC, dissimilatory sulfite reductase sulfur carrier protein	FOKN1_1950	N	N	0.153%
	FOKN1_1941	N	N	0.033%
DsrM, dissimilatory sulfite reductase, membrane protein	FOKN1_1951	N	Y	0.050%
DsrK, dissimilatory sulfite reductase, iron-sulfur protein	FOKN1_1952	N	N	0.066%
DsrL, dissimilatory sulfite reductase, intracellular sulfur oxidation protein	FOKN1_1953	N	N	0.096%
DsrJ, dissimilatory sulfite reductase, triheme cytochrome c	FOKN1_1954	Y	N	n.f
DsrO, dissimilatory sulfite reductase, iron-sulfur protein	FOKN1_1955	N	Y	0.087%
DsrP, dissimilatory sulfite reductase,*b*-type cytochrome transmembrane protein	FOKN1_1956	N	Y	n.f
DsrN, dissimilatory sulfite reductase	FOKN1_1958	N	N	n.f
DsrR, dissimilatory sulfite reductase, Fe-S cluster	FOKN1_1959	N	N	n.f
AprA, APS reductase	FOKN1_1663	N	N	n.f
AprB, APS reductase	FOKN1_1662	N	N	0.578%
SAT, sulfate adenylyltransferase	FOKN1_1877	N	N	0.273%
SoeA, sulfite oxidoreductase	FOKN1_0492	N	N	0.029%
SoeB, sulfite oxidoreductase	FOKN1_0491	N	N	n.f
SoeC, sulfite oxidoreductase	FOKN1_0490	N	Y	n.f
Fcc, flavocytochrome*c* sulfide dehydrogenase	FOKN1_726	N	N	n.f
SQR, sulfide:quinone oxidoreductase	FOKN1_1794	N	N	0.087%
SirA/TusR, Rhodanase	FOKN1_1154	N	N	n.f
PSR, sulfur/persulfide dioxygenase	FOKN1_1013	N	N	n.f
SoxY, Sulfur oxidizing protein	FOKN1_1151	N	N	0.839%
SoxZ, Sulfur oxidizing protein	FOKN1_1150	N	N	7.121%
SoxA, Sulfur oxidizing protein	FOKN1_1149	N	N	0.339%
SoxX, Sulfur oxidizing protein	FOKN1_1152	N	N	n.f
SoxB, Sulfur oxidizing protein	FOKN1_1148	N	N	0.077%
SOR, sulfur oxygenase/reductase	n.f			
PSR, polysulfide reductase	n.f			

## References

[1] Ali M., Oshiki M., Rathnayake L., Ishii S., Satoh H., Okabe S. (2015). Rapid and successful start-up of anammox process by immobilizing the minimal quantity of biomass in PVA-SA gel beads. Water Res.

[2] Ali M., Oshiki M., Awata T. (2015). Physiological characterization of anaerobic ammonium oxidizing bacterium “*Candidatus* Jettenia caeni”. Environ Microbiol.

[3] Altschul S.F., Madden T.L., Schaffer A.A., Zhang J., Zhang Z., Miller W., Lipman D.J. (1997). Gapped BLAST and PSI-BLAST: a new generation of protein database search programs. Nucleic Acids Res.

[4] Rice E.W., Baird R.B., Eaton A.D., Clesceri L.S., American Public Health Association, American Water Works Association, and Water Environment Federation (2012). 4500-SO_4_^2−^ turbidimetric method. Standard Methods for the Examination of Water and Wastewater.

[5] Berben T., Balkema C., Sorokin D.Y., Muyzer G. (2017). Analysis of the genes involved in thiocyanate oxidation during growth in continuous culture of the haloalkaliphilic sulfur-oxidizing bacterium *Thioalkalivibrio thiocyanoxidans* ARh 2^T^ using transcriptomics. mSystems.

[6] Berben T., Overmars L., Sorokin D.Y., Muyzer G. (2017). Comparative genome analysis of three thiocyanate oxidizing *Thioalkalivibrio* species isolated from soda lakes. Front Microbiol.

[7] Bhunia F., Saha N.C., Kaviraj A. (2000). Toxicity of thiocyanate to fish, plankton, worm, and aquatic ecosystem. Bull Environ Contam Toxicol.

[8] Caporaso J.G., Lauber C.L., Walters W.A. (2012). Ultra-high-throughput microbial community analysis on the Illumina HiSeq and MiSeq platforms. ISME J.

[9] Chin C.S., Alexander D.H., Marks P. (2013). Nonhybrid, finished microbial genome assemblies from long-read SMRT sequencing data. Nat Methods.

[10] Clarridge J.E. (2004). Impact of 16S rRNA gene sequence analysis for identification of bacteria on clinical microbiology and infectious diseases. Clin Microbiol Rev.

[11] Dahl C., Franz B., Hensen D., Kesselheim A., Zigann R. (2013). Sulfite oxidation in the purple sulfur bacterium *Allochromatium vinosum*: identification of SoeABC as a major player and relevance of SoxYZ in the process. Microbiology.

[12] Delcher A.L., Harmon D., Kashif S., White O., Salzberg S.L. (1999). Improved microbial gene identification with GLIMMER. Nucleic Acids Res.

[13] DeSantis T.Z., Hugenholtz P., Larsen N. (2006). Greengenes, a chimera-checked 16S rRNA gene database and workbench compatible with ARB. Appl Environ Microbiol.

[14] Edgar R.C. (2010). Search and clustering orders of magnitude faster than BLAST. Bioinformatics.

[15] Elias S., Banin E. (2012). Multi-species biofilms: living with friendly neighbors. FEMS Microbiol Rev.

[16] Frigaard N.U., Dahl C. (2009). Sulfur metabolism in phototrophic sulfur bacteria. Adv Microb Physiol.

[17] Ghosh W., Dam B. (2009). Biochemistry and molecular biology of lithotrophic sulfur oxidation by taxonomically and ecologically diverse bacteria and archaea. FEMS Microbiol Rev.

[18] Gould W.D., King M., Mohapatra B.R., Cameron R.A., Kapoor A., Koren D.W. (2012). A critical review on destruction of thiocyanate in mining effluents. Miner Eng.

[19] Hirakata Y., Oshiki M., Kuroda K., Hatamoto M., Kubota K., Yamaguchi T., Harada H., Araki N. (2016). Effects of predation by protists on prokaryotic community function, structure, and diversity in anaerobic granular sludge. Microbes Environ.

[20] Hussain A., Ogawa T., Saito M., Sekine T., Nameki M., Matsushita Y., Hayashi T., Katayama Y. (2013). Cloning and expression of a gene encoding a novel thermostable thiocyanate-degrading enzyme from a mesophilic alphaproteobacteria strain THI201. Microbiology.

[21] Ishihama Y., Oda Y., Tabata T., Sato T., Nagase T., Rappsilber J., Mann M. (2005). Exponentially modified protein abundance index (emPAI) for estimation of absolute protein amount in proteomics by the number of sequenced peptides per protein. Mol Cell Proteomics.

[22] Kantor R.S., Huddy R.J., Iyer R., Thomas B.C., Brown C.T., Anantharaman K., Tringe S., Hettich R.L., Harrison S.T.L., Banfield J.F. (2017). Genome-resolved meta-omics ties microbial dynamics to process performance in biotechnology for thiocyanate degradation. Environ Sci Technol.

[23] Kasahara Y., Morimoto H., Kuwano M., Kadoya R. (2012). Genome-wide analytical approaches using semi-quantitative expression proteomics for aromatic hydrocarbon metabolism in *Pseudomonas putida* F1. J Microbiol Methods.

[24] Katayama Y., Kuraishi H. (1978). Characteristics of *Thiobacillus thioparus* and its thiocyanate assimilation. Can J Microbiol.

[25] Katayama Y., Narahara Y., Inoue Y., Amano F., Kanagawa T., Kuraishi H. (1992). A thiocyanate hydrolase of *Thiobacillus thioparus*. J Biol Chem.

[26] Kozliak E.I., Fuchs J.A., Guilloton M.B., Anderson P.M. (1995). Role of bicarbonate/CO_2_ in the inhibition of *Escherichia coli* growth by cyanate. J Bacteriol.

[27] Krogh A., Larsson B., von Heijne G., Sonnhammer E.L.L. (2001). Predicting transmembrane protein topology with a hidden Markov model: Application to complete genomes. J Mol Biol.

[28] Kumar S., Stecher G., Tamura K. (2016). MEGA7: molecular evolutionary genetics analysis version 7.0 for bigger datasets. Mol Biol Evol.

[29] Lee I., Kim Y.O., Park S.C., Chun J. (2015). OrthoANI: An improved algorithm and software for calculating average nucleotide identity. Int J Syst Evol Microbiol.

[30] Lim B.R., Hu H.Y., Huang X., Fujie K. (2002). Effect of seawater on treatment performance and microbial population in a biofilter treating coke-oven wastewater. Process Biochem (Rickmansworth, U K).

[31] Lowe T.M., Eddy S.R. (1997). tRNAscan-SE: A program for improved detection of transfer RNA genes in genomic sequence. Nucleic Acids Res.

[32] Madigan M.T., Martinko J.M., Parker J., Carlson G., Snavely S.L., Wechsler D.A. (2002). Osmotic effects on microbial growth. Brock Biology of Microorganisms.

[33] Masella A.P., Bartram A.K., Truskowski J.M., Brown D.G., Neufeld J.D. (2012). PANDAseq: paired-end assembler for illumina sequences. BMC Bioinformatics.

[34] Michaels G.B., Davidson J.T., Peck H.D. (1970). A flavin-sulfite adduct as an intermediate in the reaction catalyzed by adenylyl sulfate reductase from *Desulfovibrio vulgaris*. Biochem Biophys Res Commun.

[35] Moriya Y., Itoh M., Okuda S., Yoshizawa A., Kanehisa M. (2007). KAAS: an automatic genome annotation and pathway reconstruction server. Nucleic Acids Res.

[36] Noguchi H., Taniguchi T., Itoh T. (2008). MetaGeneAnnotator: detecting species-specific patterns of ribosomal binding site for precise gene prediction in anonymous prokaryotic and phage genomes. DNA Res.

[37] Ogawa T., Noguchi K., Saito M. (2013). Carbonyl sulfide hydrolase from *Thiobacillus thioparus* strain THI115 is one of the β-carbonic anhydrase family enzymes. J Am Chem Soc.

[38] Okumura M., Fujinaga K., Seike Y., Nagashima A. (1998). In situ pre concentration method for thiosulfate and sulphite in environmental water samples using solid-phase extraction followed by a spectrophotometric determination. Bunseki Kagaku.

[39] Oshiki M., Shimokawa M., Fujii N., Satoh H., Okabe S. (2011). Physiological characteristics of the anaerobic ammonium-oxidizing bacterium ‘*Candidatus* Brocadia sinica’. Microbiology.

[40] Oshiki M., Shinyako-Hata K., Satoh H., Okabe S. (2015). Draft genome sequencing of anaerobic ammonium oxidizing bacterium, “*Candidatus* Brocadia sinica”. Genome Announc.

[41] Oshiki M., Fukushima T., Kawano S., Nakagawa J. (2017). Draft genome sequence of *Thiohalobacter thiocyanaticus* strain FOKN1, a neutrophilic halophile capable of thiocyanate degradation. Genome Announc.

[42] Perkins D.N., Pappin D.J., Creasy D.M., Cottrell J.S. (1999). Probability-based protein identification by searching sequence databases using mass spectrometry data. Electrophoresis.

[43] Petersen T.N., Brunak S., von Heijne G., Nielsen H. (2011). SignalP 4.0: Discriminating signal peptides from transmembrane regions. Nat Methods.

[44] Pott A.S., Dahl C. (1998). Sirohaem sulfite reductase and other proteins encoded by genes at the *dsr* locus of *Chromatium vinosum* are involved in the oxidation of intracellular sulfur. Microbiology.

[45] Richter M., Rosselló-Móra R. (2009). Shifting the genomic gold standard for the prokaryotic species definition. Proc Natl Acad Sci USA.

[46] Shoji T., Sueoka K., Satoh H., Mino T. (2014). Identification of the microbial community responsible for thiocyanate and thiosulfate degradation in an activated sludge process. Process Biochem (Oxford, U K).

[47] Shinoda K., Tomita M., Ishihama Y. (2010). emPAI Calc—for the estimation of protein abundance from large-scale identification data by liquid chromatography–tandem mass spectrometry. Bioinformatics.

[48] Simão F.A., Waterhouse R.M., Ioannidis P., Kriventseva E.V., Zdobnov E.M. (2015). BUSCO: assessing genome assembly and annotation completeness with single-copy orthologs. Bioinformatics.

[49] Sorokin D.Y., Tourova T.P., Lysenko A.M., Gijs Kuenen J. (2001). Microbial thiocyanate utilization under highly alkaline conditions. Appl Environ Microbiol.

[50] Sorokin D.Y., Tourova T.P., Lysenko A.M., Mityushina L.L., Kuenen J.G. (2002). *Thioalkalivibrio thiocyanoxidans* sp. nov. and *Thioalkalivibrio paradoxus* sp. nov., novel alkaliphilic, obligately autotrophic, sulfur-oxidizing bacteria capable of growth on thiocyanate, from soda lakes. Int J Syst Evol Microbiol.

[51] Sorokin D.Y., Tourova T.P., Bezsoudnova E.Y., Pol A., Muyzer G. (2007). Denitrification in a binary culture and thiocyanate metabolism in *Thiohalophilus thiocyanoxidans* gen. nov. sp. nov.—a moderately halophilic chemolithoautotrophic sulfur-oxidizing Gammaproteobacterium from hypersaline lakes. Arch Microbiol.

[52] Sorokin D.Y., Kovaleva O.L., Tourova T.P., Muyzer G. (2010). *Thiohalobacter thiocyanaticus* gen. nov., sp. nov., a moderately halophilic, sulfur-oxidizing gammaproteobacterium from hypersaline lakes, that utilizes thiocyanate. Int J Syst Evol Microbiol.

[53] Sörbo B. (1957). A colorimetric method for the determination of thiosulfate. Biochem Biophys Acta.

[54] Staib C., Lant P. (2007). Thiocyanate degradation during activated sludge treatment of coke-ovens wastewater. Biochem Eng J.

[55] Sueoka K., Satoh H., Onuki M., Mino T. (2008). Microorganisms involved in anaerobic phenol degradation in the treatment of synthetic coke-oven wastewater detected by RNA stable-isotope probing. FEMS Microbiol Lett.

[56] Sugawara H., Ohyama A., Mori H., Kurokawa K. (2009). Microbial genome annotation pipeline (MiGAP) for diverse users.

[57] Tchobanoglous G., Burton F.L., Stensel H.D., Burton F., Tchobanoglous G., Burton F.L., Stensel H.D., Metcalf & Eddy Inc (2004). Suspended growth biological treatment processes. Wastewater Engineering: Treatment and Reuse.

[58] Thompson J.D., Higgins D.G., Gibson T.J. (1994). CLUSTAL W: improving the sensitivity of progressive multiple sequence alignment through sequence weighting, position-specific gap penalties and weight matrix choice. Nucleic Acids Res.

[59] Toh S.K., Ashbolt N.J. (2002). Adaptation of anaerobic ammonium-oxidising consortium to synthetic coke-ovens wastewater. Appl Microbiol Biotechnol.

[60] Trüper H.G., Schlegel H.G. (1964). Sulfur metabolism in *Thiorhodaceae*. Quantitative measurements on growing cells of *Chromatium okeanii*. Antonie van Leeuwenhoek.

[61] Tsallagov S.I., Sorokin D.Y., Tikhonova T.V., Popov V.O., Muyzer G. (2019). Comparative genomics of *Thiohalobacter thiocyanaticus* HRh1T and *Guyparkeria* sp. SCN-R1, halophilic chemolithoautotrophic sulfur-oxidizing gammaproteobacteria capable of using thiocyanate as energy source. Front Microbiol.

[62] Zopfi J., Ferdelman T.G., Jørgensen B.B., Teske A., Thamdrup B. (2001). Influence of water column dynamics on sulfide oxidation and other major biogeochemical processes in the chemocline of Mariager Fjord (Denmark). Mar Chem.

