# Supplementary material for: Thiocyanate Degradation by a Highly Enriched Culture of the Neutrophilic Halophile Thiohalobacter sp. Strain FOKN1 from Activated Sludge and Genomic Insights into Thiocyanate Metabolism
Source: Microbes Environ. 2019 Dec 27;34(4):402–12. doi: 10.1264/jsme2.ME19068 (PMC6934394; doi:10.1264/jsme2.ME19068)
Supplement: Supplementary file 1 [file 34_402_s1.pdf]

Supplemental material

Thiocyanate degradation by a highly enriched culture of the neutrophilic halophile *Thiohalobacter sp.* strain FOKN1 from activated sludge and genomic insights into thiocyanate metabolism

Mamoru Oshiki[1*], Toshikazu Fukushima[2], Shuichi Kawano[3], Yasuhiro Kasahara[4] & Junichi Nakagawa[2]

[1]Department of Civil Engineering, National Institute of Technology, Nagaoka College, Japan

[2]Advanced Technology Research Laboratories, Research & Development, Nippon Steel & Sumitomo Metal Corporation., Japan

[3]Department of Computer and Network Engineering Graduate School of Informatics and Engineering, The University of Electro-Communications., Japan

[4] Institute of Low-Temperature Science, Hokkaido University, Sapporo, Japan

[*]**Corresponding author:**

Mamoru Oshiki (Ph.D.)

E-mai; oshiki@nagaoka-ct.ac.jp

Tel/Fax; +81-258-34-9277/9284

<u>This file contains 5 figures, 5 tables and 1 text.</u>

Supplementary Figure

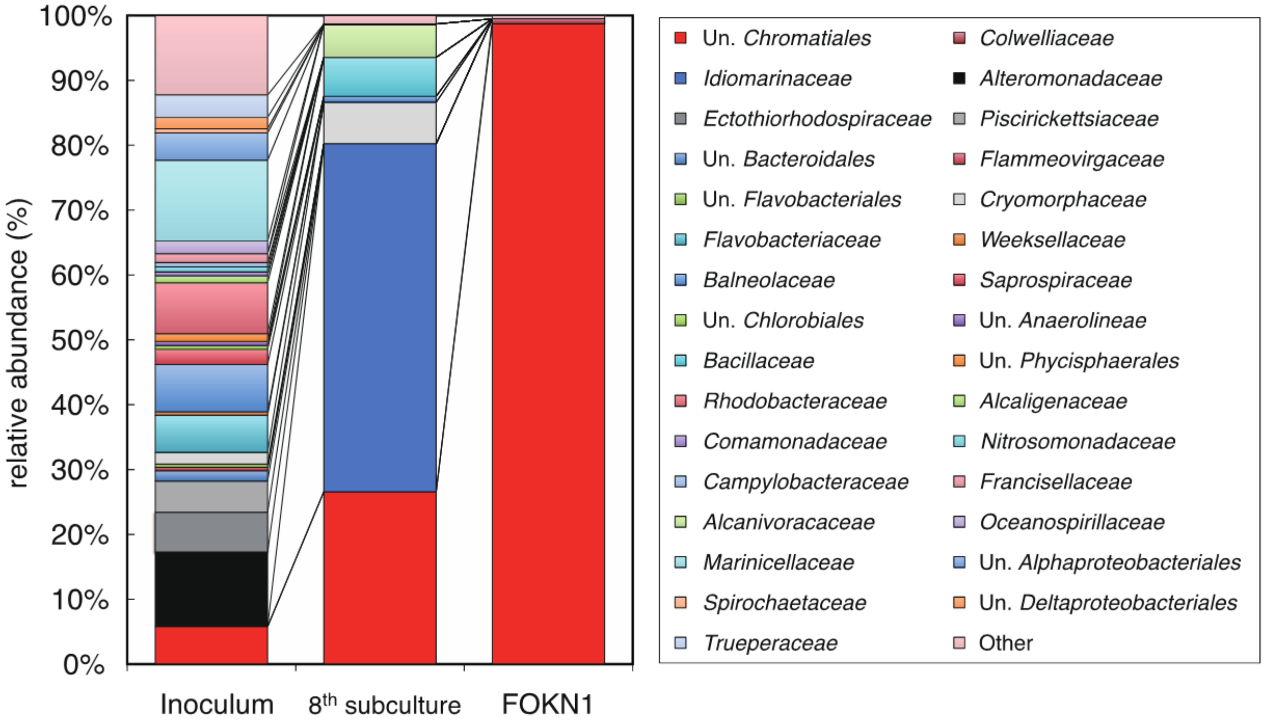

Fig. S1 (Oshiki et al.)

Figure S1 Relative abundance of partial 16S rRNA gene sequences detected from the inoculum, the culture after 8th subculturing (labeled as 8th subculture), and the enrichment culture obtained after serial dilution (FOKN1). The serial dilution was performed thrice in total to obtain the FOKN1 culture. Number of total sequence reads was 73,782, 38,567, and 21,752 for the inoculum, 8th subculture, and FOKN1 culture, respectively. Relative abundance of 16S rRNA gene sequences from each bacterial genus/family is shown. Un.; uncultured.

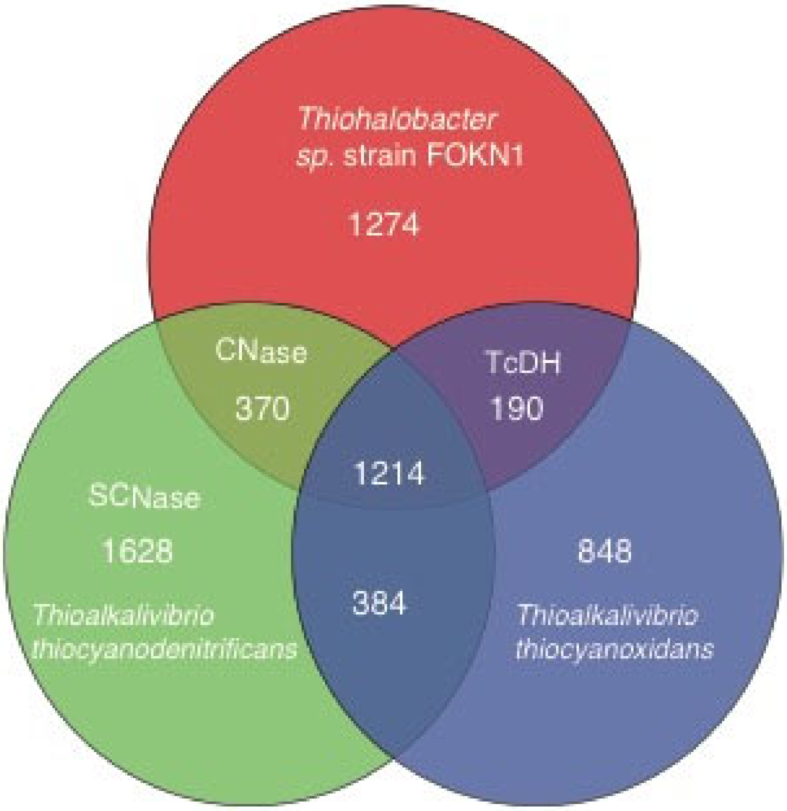

Fig. S2 (Oshiki *et al.*)

Figure S2. A venn diagram showing shared genes among thiocyanate-degrading bacterial genomes. Shared and unique genes among *Thiohalobacter sp.* strain FOKN1, *Thioalkalivibrio thiocyanodenitrificans* ARhD1 (GenBank accession number; GCA_000378965.1), and *Tv. thiocyanoxidans* ARh2 (GCA_000227685.2) were identified by blastP search with a threshold of 25% identity and 30% overlap ratio. TcDH: thiocyanate dehydrogenase, SCNase: thiocyanate hydrolase, and CNase: cyanate lyase.

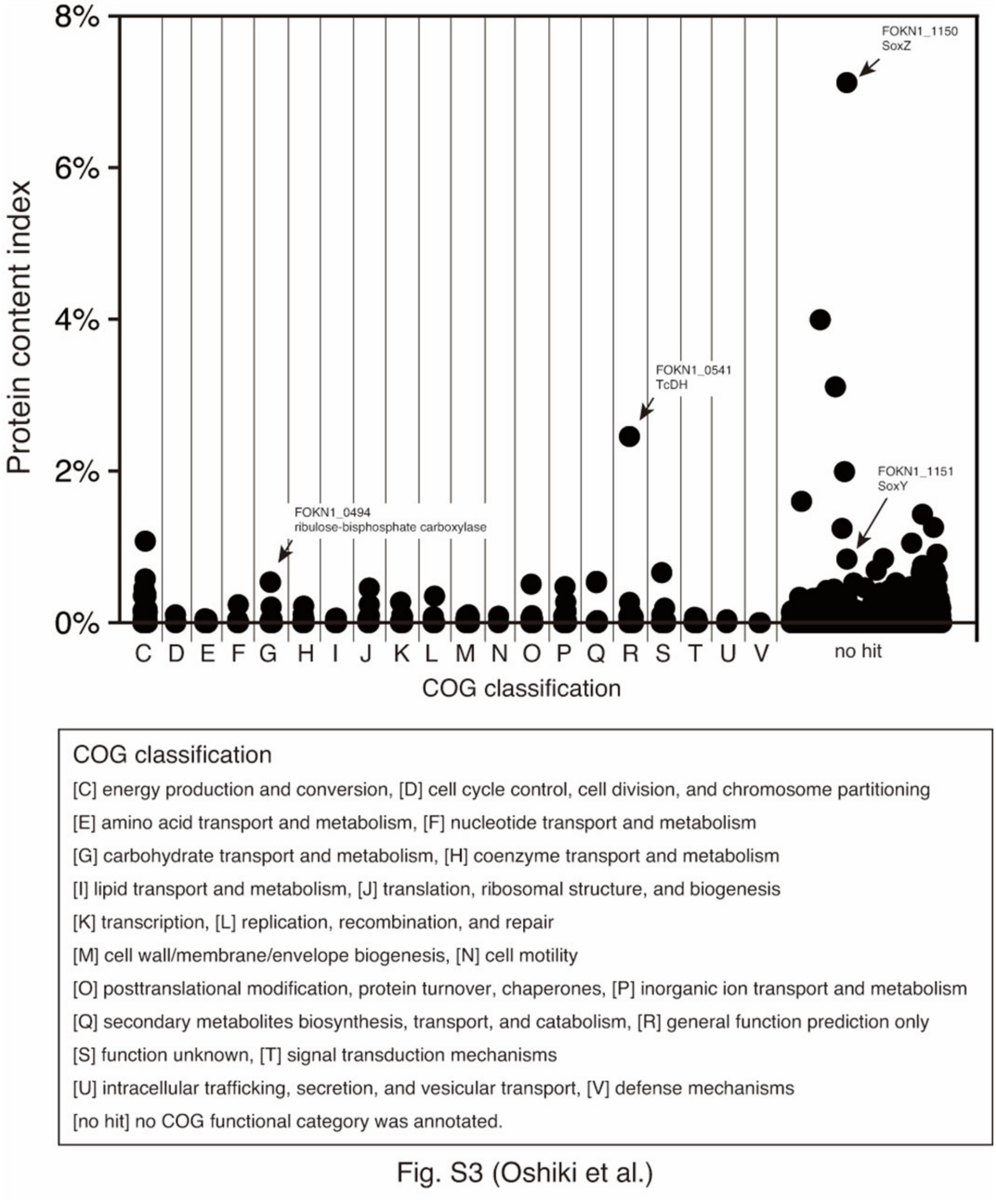

Figure S3. Proteome of the *Thiohalobacter sp.* strain FOKN1 cells. Total proteins were extracted from FOKN1 cells harvested at the late-exponential growth phase, which were analyzed by one-dimensional sodium dodecyl sulphate polyacrylamide gel electrophoresis (SDS-PAGE) and nanoscale liquid chromatography with tandem mass spectrometry (nanoLC-MS/MS). Protein abundance is represented as the protein content index (PCI). Proteins were classified into clusters of

orthologous groups (COGs). Some identified proteins are highlighted with locus tag numbers and gene/product names. TcDH; thiocyanate dehydrogenase, SoxYZ; sulfur-oxidizing protein Y and Z, respectively.

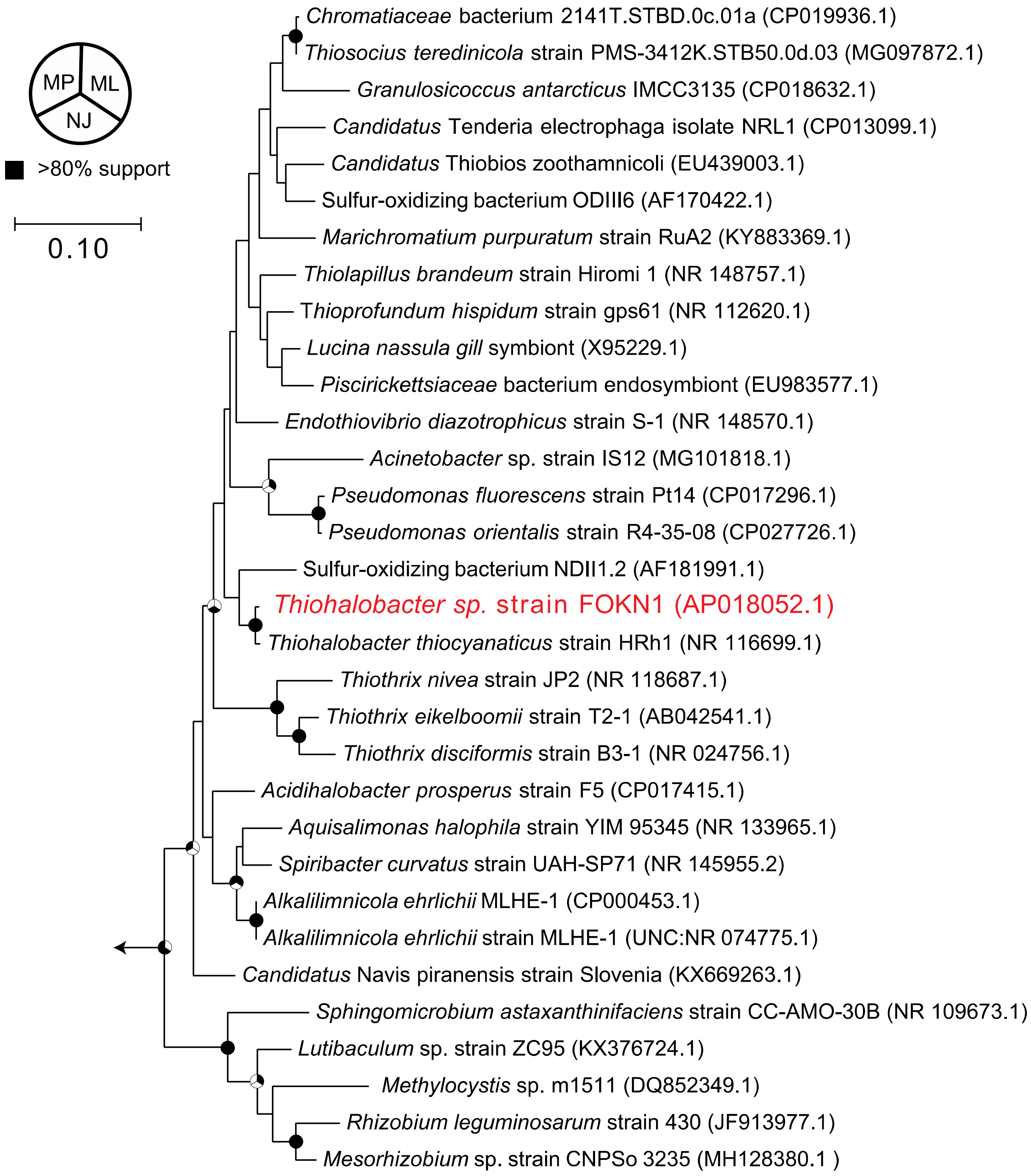

Fig. S4 (Oshiki *et al.*)

Figure S4. Phylogeny of *Thiohalobacter sp.* strain FOKN1 estimated based on 16S rRNA gene sequence. The phylogenetic tree was constructed by the maximum likelihood (ML) method (250 bootstrap iterations), neighbour joining (NJ) method (500 iterations), and maximum parsimony (MP)

method (250 iterations) using the 16S rRNA gene sequence of *Aquifex pyrophilus* (accession number M83548) as an outgroup (sequence is not shown in the tree). The phylogenetic tree calculated by the ML method is shown here. The phylogenetic position of *Thiohalobacter sp.* strain FOKN1 is highlighted in red. Pie charts at nodes represent confidence levels of branch topology, and bootstrap values greater than 80% are filled with black color (the MP method for the upper-left sector, ML method for upper-right sector, and NJ method for the bottom sector). The scale bar represents 10% sequence divergence.

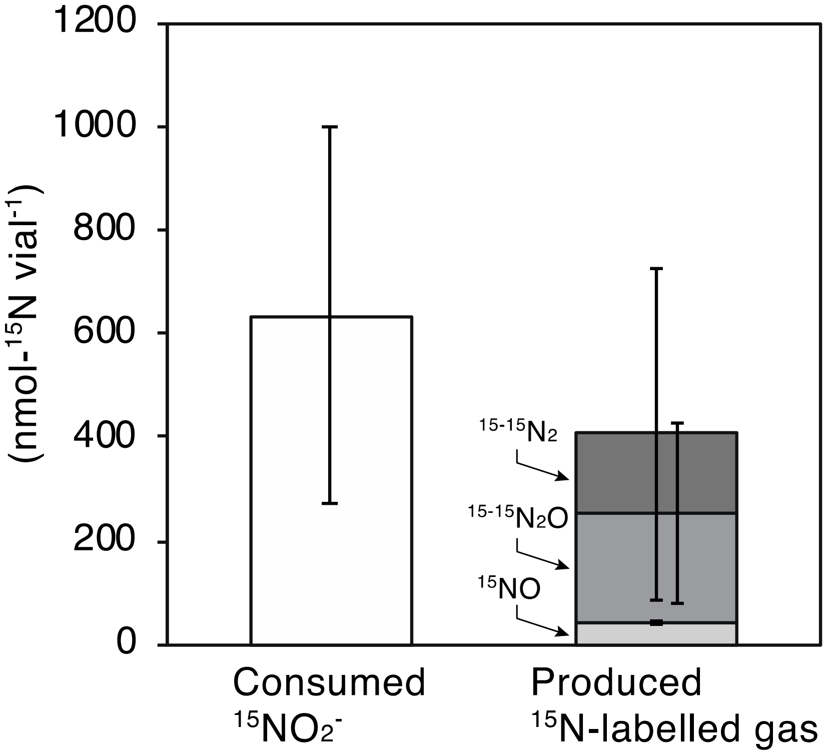

Fig. S5 (Oshiki et al.)

Figure S5. $^{15}NO_2^-$ reduction by *Thiohalobacter sp.* strain FOKN1. FOKN1 cells were anoxically incubated in closed vials in presence of 1 mM $^{15}NO_2^-$. After 65 h of incubation, concentrations of ^{15}NO, $^{15\text{-}15}N_2O$, and $^{15\text{-}15}N_2$ in the headspace were determined by gas chromatography mass spectrometry. Incubations were performed in triplicate, and the error bars represent standard deviation.

2. Supplementary Table

Table S1. Gene annotation of gene-coding sequences (CDSs) and protein expression in the *Thiohalobacter sp.* strain FOKN1 cells. SignalP; prediction of signal peptide sequence, Y; positive, N; negative, COG; clusters of orthologous group, KO; KEGG orthology annotated using the KAAS, PCI; protein content index, n.d; not detected.

(This table is composed of large data set, and thus is available as a separated file.)

Table S2. Completeness of *Thiohalobacter sp.* strain FOKN1 and *Thiohalobacter thiocyanaticus* Hrh1 genomes examined using Benchmarking Universal Single-Copy Orthologs (BUSCO) software. The gammaproteobacteria_odh9 dataset was used as the lineage dataset. The GenBank assembly accession of the *Thiohalobacter* sp. strain FOKN1 and *Thiohalobacter thiocyanaticus* Hrh1 genomes are GCA_002356355.1 and GCA_003932505.1, respectively.

Genome	FOKN1	Hrh1
Total BUSCO groups	452	452
Complete BUSCOs	434	403
Completeness	96.0%	89.2%
Missing BUSCOs	POG090900BT<br>POG090900E3<br>POG090900ZM<br>POG0909017T<br>POG090901DP<br>POG090901NG<br>POG0909022J<br>POG090902B6<br>POG090902DH<br>POG090902KA<br>POG090902MG<br>POG090902NM<br>POG090902SD<br>POG090903DM	POG0909002X, POG090901DJ, POG09090284<br>POG0909009G, POG090901DP, POG090902B6<br>POG090900B6, POG090901EB, POG090902D8<br>POG090900BT, POG090901ED, POG090902DH<br>POG090900E3, POG090901HP, POG090902KA<br>POG090900EQ, POG090901L6, POG090902MG<br>POG090900LM, POG090901ND, POG090902NM<br>POG090900OW, POG090901NG, POG090902SD<br>POG090900Q6, POG090901NI, POG090902UD<br>POG090900TD, POG090901OK, POG090902VV<br>POG090900UU, POG090901XM, POG0909030Z<br>POG090900ZM, POG0909022J, POG09090325<br>POG0909017T, POG09090268, POG0909032A<br>POG090903CJ, POG090903DM, POG0909038A<br>POG090903F3

Table S3 Comparison of average nucleotide identity (ANI) values among the known SCN^--degrading bacterial genomes. FOKN1; *Thiohalobacter sp.* strain FOKN1, Hrh1; *Thiohalobacter thiocyanaticus* Hrh1, ARhD1; *Thioalkalivibrio thiocyanodenitrificans* ARhD1, and ARh2; *Thioalkalivibrio thiocyanoxidans* ARh2.

	FOKN1	Hrh1	ARhD1	ARh2
FOKN1				
Hrh1	86.1			
ARhD1	71.6	71.6		
ARh2	71.0	71.0	71.5	

Table S4. *Thiohalobacter* sp. strain FOKN1 proteins identified on the basis of two or more peptides. emPAI; exponentially modified protein abundance index, PCI; protein content index.

(This table is composed of large data set, and thus is available as a separated file.)

Table S5. Gene sets required for NO_2^- reduction to N_2 of *Thiohalobacter sp.* strain FOKN1.

Protein (gene)	locus_tag	product	blast hit product	organisms	accession number	*e*-value	identity
cytochrome cd_1-type nitrite reductase (NirS)							
(nirS)	FOKN1_0459	cytochrome cd_1 type nitrite reductase	nitrite reductase	*Marinobacter nitratireducens*	WP_036129651.1	0	78%
(nirC)	FOKN1_0461	cytochrome *c*	cytochrome *c*	*Thauera chlorobenzoica*	WP_075148782.1	2.00E-32	63%
(nirF)	FOKN1_0462	heme d_1 synthesis protein	protein nirF	*Acidithiobacillales bacterium* SG8_45	KPK11657.1	0	70%
(nirL)	FOKN1_0464	heme d_1 synthesis protein	Lrp/AsnC family transcriptional regulator	*Thioalkalivibrio denitrificans*	WP_077278262.1	4.00E-70	59%
(nirG)	FOKN1_0465	transcriptional regulator	transcriptional regulator, AsnC family	*Halomonas ilicicola* DSM 19980	SHE31039.1	3.00E-60	61%
(nirJ)	FOKN1_0467	heme d_1 synthesis protein	heme d1 biosynthesis radical SAM protein NirJ	*Candidatus* Competibacter denitrificans	WP_048676816.1	0	70%
(nirN)	FOKN1_0468	cytochrome *c*	cytochrome Cbb_3	endosymbiont of unidentified scaly snail isolate Monju	WP_043108187.1	0	73%
nitric oxide reductase (NorB)							
(norC)	FOKN1_0471	nitric-oxide reductase subunit C	cytochrome *c*	*Ketobacter alkanivorans*	WP_101894046.1	2.00E-86	81%
(norB)	FOKN1_0472	nitric oxide reductase, subunit B	nitric oxide reductase	endosymbiont of unidentified scaly snail isolate Monju	WP_043108184.1	0	88%
nitrous oxide reductase (NosZ)							
(nosR)	FOKN1_0424	nitrous oxide reductase maturation protein	regulatory protein NosR	*Halomonas xianhensis*	WP_092847785.1	0	58%
(nosZ)	FOKN1_0425	nitrous-oxide reductase	nitrous-oxide reductase	*Halomonas xianhensis*	WP_092847782.1	0	74%
(nosD)	FOKN1_0426	nitrous oxide reductase maturation protein	nitrous oxide reductase family maturation protein NosD	*Thioalkalivibrio paradoxus*	WP_006747564.1	1.00E-163	59%
(nosF)	FOKN1_0427	nitrous oxide reductase maturation protein	ABC transporter ATP-binding protein	*Thioalkalivibrio nitratireducens*	WP_015258430.1	2.00E-110	60%
(nosY)	FOKN1_0428	nitrous oxide reductase maturation transmembrane	nitrous oxide reductase maturation protein NosY	*Thioalkalivibrio nitratireducens*	WP_015258431.1	1.00E-99	59%

			blast hit				
Protein (gene)	locus_tag	product	product	organisms	accession number	*e*-value	identity
		protein					
(nosL)	FOKN1_0430	protein disulfide isomerase	nitrous oxide reductase accessory protein NosL	*Vibrio tubiashii*	WP_004747062.1	9.00E-53	51%

3. Supplementary Text

3.1 $^{15}NO_2^-$ reduction by FOKN1 cells

NO_2^- reduction by *Thiohalobacter sp.* strain FOKN1 was examined because the following gene clusters were located in the genome; *nirSCFLGJN*, *norBC*, and *nosRZDFYL* (**Table S5**). *nirS*, *norB*, and *nosZ* encode NO-forming cytochrome *cd$_1$*-type nitrite reductase, nitric oxide reductase, and nitrous oxide reductase (4, 6). No gene encoding canonical nitrate reductase (i.e., Nar and Nap) was found. The presence of *nir*, *nor* and *nos* suggests that *Thiohalobacter sp.* strain FOKN1 is a denitrifier reducing NO_2^- to N_2. Nitrite reduction of *Thiohalobacter sp.* strain FOKN1 was examined using a $^{15}NO_2^-$ tracer as previously described (3). FOKN1 culture suspended in inorganic medium was dispensed into 15-mL glass serum vials. After sealing with butyl rubber stoppers and aluminum caps, headspace was replaced by vacuuming and purging with He gas (>99.99995%). Anoxic stock solution of $^{15}NO_2^-$ (Cambridge isotope laboratories, Tewksbury, MA, USA) was dispensed using a gas tight syringe at a final concentration of 1 mM. Vials were incubated at 30°C in dark. Ten microliters of headspace gas was collected using a gas-tight glass syringe, and immediately injected to a GCMS-QP2010SE gas chromatograph (Shimadzu, Kyoto, Japan) equipped with a CP-Pora BOND Q fused silica capillary column (Agilent Technologies, Santa Clara, CA, USA) to measure ^{15}N-labeled N_2, NO, and N_2O gas concentrations (1). Standard curves for $^{15\text{-}15}N_2$ gas and ^{15}NO and $^{15\text{-}15}N_2O$ gas quantification were prepared with $^{15\text{-}15}N_2$ (Cambridge isotope laboratories), ^{14}NO (GL Science, Tokyo, Japan), and $^{14\text{-}14}N_2O$ (Shimakyu, Nagaoka, Japan) gases. As for NO_2^- concentration, the concentration was determined using naphthylethylenediamine methods. Liquid samples filtered with a 0.45-μm pore PDVF filter were mixed with 4.9 mM naphthylethylenediamine solution and absorbance was measured at 540 nm.

FOKN1 cells were anoxically incubated with 1 mM $^{15}NO_2^-$ and 3.44 mM SCN^-, and $^{15}NO_2^-$ was reduced to $^{15\text{-}15}N_2$ and $^{15\text{-}15}N_2O$ during 65 h of incubation (**Fig. S5**). $^{15}NO_2^-$ reduction halted after

65 h of incubation although $^{15}NO_2^-$ was still available (ca. 0.6 mM). This observation suggests that NO_2^- reduction (i.e., nitrite respiration) cannot support growth of *Thiohalobacter sp.* strain FOKN1.

3.2 Isolation of Thiohalobacter sp. **strain FOKN1 TcDH and activity assay**

Thiohalobacter sp. strain FOKN1 cells (3.35 g-wet) were suspended at concentrations of 0.11 g-wet ml^{-1} in 50 mM phosphate buffer (pH 7.5) containing 1 mM phenylmethylsulfonyl fluoride, and disrupted thrice by French press (11,000 psi) (SLM Aminco, Urbana, IL, USA). The suspension was centrifuged at 110,000 g and 4°C for 60 min, and the supernatant was collected as soluble protein fraction. TcDH was found in the soluble protein fraction, which was purified with Q sepharose XL media (GE healthcare, Little Chalfont, UK) equilibrated with 20 mM Tris-HCl buffer (pH 7.5). Binding proteins were eluted by linearly increasing NaCl concentration in the Tris buffer. Proteins eluted at the concentration of 0.1 M NaCl contained TcDH, which were further purified by gel chromatography using a Superdex 200 gel filtration column (GE healthcare) equilibrated a 20 mM phosphate buffer (pH7.5) containing 0.15 M NaCl. The fractions containing TcDH were pooled and subjected to SDS-PAGE analysis to examine purity of TcDH in the collected fraction. Proteins were separated on a 10% SDS-containing polyacrylamide gel, and stained with CBB Protein Safe Stain (Takarabio, Shiga, Japan) following the instruction manual supplied by manufactures. Identification of TcDH was conducted by excising protein bands from the gel and subjected to matrix assisted laser desorption ionization-time of flight mass spectrometry (MALDI-TOF MS) analysis after in-gel tryptic digestion for protein identification as previously described (2). Peptide mass fingerprints were analysed using the MASCOT search program version 2.3.01 (5). The amino acid sequences of genes located in the *Thiohalobacter sp.* strain FOKN1 genome (accession number: AP018052) were used as the reference database. The protein fractions containing TcDH were dialyzed using a mini dialysis kit (1 kDa MWCO, GE healthcare) against a buffer containing 50 mM Tris-HCl buffer (pH 7.5), 0.15 M NaCl, 0.5 mM $CuSO_4$ for 70 h for reconstitution of Cu-binding sites of TcDH. The protein

solution was dialyzed again using the above buffer without $CuSO_4$ at 4°C for 4 h, and the dialyzed TcDH (7.3 mg ml^{-1}) was subjected to activity assay.

SCN oxidation by the obtained TcDH was examined by a batch incubation of TcDH with addition of SCN. The assay buffer containing 50 mM PO_4 (pH 7), 2 mg ml^{-1} TcDH, 0.2 mg ml^{-1} cytochrome *c* (from equine heart) (FUJIFILM Wako pure chemical corporation, Osaka, Japan), 2 mM SCN^- was aerobically incubated at 37°C in triplicates. After 60 min of the incubation, SCN concentration in liquid phase was determined colorimetrically.

Reference

1. Isobe, K., K. Koba, S. Ueda, K. Senoo, S. Harayama, and Y. Suwa. 2011. A simple and rapid GC/MS method for the simultaneous determination of gaseous metabolites. J. Microbiol. Methods 84:46-51.
2. Kobayashi, S., D. Hira, K. Yoshida, M. Toyofuku, Y. Shida, W. Ogasawara, T. Yamaguchi, N. Araki, and M. Oshiki. 2018. Nitric oxide production from nitrite reduction and hydroxylamine oxidation by copper-containing dissimilatory nitrite reductase (NirK) from the aerobic ammonia-oxidizing archaeon, *Nitrososphaera viennensis*. Microbes Environ. 33:428-434.
3. Oshiki, M., M. Ali, K. Shinyako-Hata, H. Satoh, and S. Okabe. 2016. Hydroxylamine-dependent anaerobic ammonium oxidation (anammox) by "*Candidatus* Brocadia sinica. Environ. Microbiol. 18:3133-3143.
4. Oshiki, M., T. Segawa, and S. Ishii. 2018. Nitrogen cycle evaluation (NiCE) chip for the simultaneous analysis of multiple N-cycle associated genes. Appl. Environ. Microbiol. 84:e02615-17.
5. Perkins, D.N., D.J. Pappin, D.M. Creasy, and J.S. Cottrell. 1999. Probability-based protein identification by searching sequence databases using mass spectrometry data. Electrophoresis 20:3551-3567.

6. Zumft, W.G. 1997. Cell biology and molecular basis of denitrification. Microbiol. Mol. Biol. Rev. 61:533-616.
