# Supplementary material for: Thiocyanate Degradation by a Highly Enriched Culture of the Neutrophilic Halophile Thiohalobacter sp. Strain FOKN1 from Activated Sludge and Genomic Insights into Thiocyanate Metabolism
Source: Microbes Environ. 2019 Dec 27;34(4):402–12. doi: 10.1264/jsme2.ME19068 (PMC6934394; doi:10.1264/jsme2.ME19068)
Supplement: Supplementary file 2 [file 34_402_s2.pdf]

Table S1. Gene annotation of gene-coding sequences (CDSs) and protein expression in the *Thiohalobacter sp.* strain FOKN1 cells. SignalP; prediction of signal peptide sequence, Y; positive, N; negative, COG; clusters of orthologous group, KO; KEGG orthology annotated using the KAAS, PCI; protein content index, n.d; not detected.

Locus_tag	Product	SignalP	COG	KO	PCI (%)
FOKN1_0001	phosphate-selective porin O and P	Y	no hit		n.d
FOKN1_0002	formate-dependent nitrite reductase, periplasmic cytochrome c552 subunit	N	no hit		n.d
FOKN1_0003	uncharacterized protein	Y	no hit		n.d
FOKN1_0004	uncharacterized protein	N	no hit		n.d
FOKN1_0005	rhodanese-related sulfurtransferase	N	no hit		0.020%
FOKN1_0006	polyketide synthase	N	L		n.d
FOKN1_0007	Fe-S oxidoreductase	N	C		n.d
FOKN1_0008	uncharacterized protein	N	L		n.d
FOKN1_0009	sirA family protein	N	R		n.d
FOKN1_0010	transporter component	N	R	K07112	n.d
FOKN1_0011	sirA family protein	N	R		0.086%
FOKN1_0012	alkyl hydroperoxide reductase	N	no hit	K03386	0.136%
FOKN1_0013	uncharacterized protein	N	no hit		n.d
FOKN1_0014	uncharacterized protein	N	no hit	K09803	n.d
FOKN1_0015	penicillin-binding protein-related factor A, putative recombinase	N	no hit		n.d
FOKN1_0016	phage-related protein, tail component	N	no hit		0.134%
FOKN1_0017	uncharacterized protein	N	no hit		0.027%
FOKN1_0018	transposase	N	no hit		n.d
FOKN1_0019	Cu2+-exporting ATPase	N	no hit	K01533	n.d
FOKN1_0020	integral membrane protein	N	P		n.d
FOKN1_0021	iron-sulfur cluster-binding protein	N	C		n.d
FOKN1_0022	cbb3-type cytochrome c oxidase subunit	N	no hit	K00406	0.158%
FOKN1_0023	Na+-driven multidrug efflux pump	N	no hit		n.d
FOKN1_0024	cytochrome c oxidase cbb3-type subunit II	N	no hit	K00405	0.056%
FOKN1_0025	cbb3-type cytochrome c oxidase subunit I	N	no hit		n.d
FOKN1_0026	polyprenyltransferase	N	no hit	K02257	n.d
FOKN1_0027	uncharacterized protein	N	no hit		n.d
FOKN1_0028	uncharacterized protein	N	no hit		n.d
FOKN1_0029	uncharacterized protein	N	no hit		n.d
FOKN1_0030	cytochrome c oxidase subunit III	N	no hit	K02276	n.d
FOKN1_0031	cytochrome c oxidase assembly protein CtaG	N	no hit	K02258	n.d
FOKN1_0032	cytochrome c oxidase subunit I	N	no hit	K02274	n.d

Table S1. Gene annotation of gene-coding sequences (CDSs) and protein expression in the *Thiohalobacter sp.* strain FOKN1 cells. SignalP; prediction of signal peptide sequence, Y; positive, N; negative, COG; clusters of orthologous group, KO; KEGG orthology annotated using the KAAS, PCI; protein content index, n.d; not detected.

Locus_tag	Product	SignalP	COG	KO	PCI (%)
FOKN1_0033	cytochrome B559 subunit alpha	N	no hit	K02275	n.d
FOKN1_0034	integral membrane protein	N	no hit		n.d
FOKN1_0035	ATP-dependent dethiobiotin synthetase BioD	N	no hit	K01935	n.d
FOKN1_0036	malonyl-[acyl-carrier protein] O-methyltransferase	N	no hit	K02169	0.012%
FOKN1_0037	pimeloyl-[acyl-carrier protein] methyl ester esterase	N	no hit	K02170	n.d
FOKN1_0038	8-amino-7-oxononanoate synthase	N	no hit	K00652	n.d
FOKN1_0039	biotin synthase	N	no hit	K01012	n.d
FOKN1_0040	heme/copper-type cytochrome/quinol oxidase, subunit 3	N	C		n.d
FOKN1_0041	putative amidophosphoribosyltransferase	N	no hit		n.d
FOKN1_0042	integral membrane protein	N	no hit		n.d
FOKN1_0043	uncharacterized protein	N	no hit		n.d
FOKN1_0044	truncated hemoglobins	N	no hit	K06886	n.d
FOKN1_0045	4-hydroxybenzoate octaprenyltransferase	N	no hit	K03179	n.d
FOKN1_0046	putative chorismate-pyruvate lyase	N	no hit	K03181	n.d
FOKN1_0047	ATP-dependent DNA helicase RecG	N	no hit	K03655	n.d
FOKN1_0048	endoribonuclease	N	no hit	K09022	0.031%
FOKN1_0049	GTP diphosphokinase	N	no hit	K01139	n.d
FOKN1_0050	DNA-directed RNA polymerase subunit omega	N	no hit	K03060	n.d
FOKN1_0051	guanylate kinase	N	no hit	K00942	0.066%
FOKN1_0052	uncharacterized protein	N	no hit		0.090%
FOKN1_0053	serine/threonine protein kinase	N	no hit	K12132	n.d
FOKN1_0054	serine/threonine protein phosphatase	N	J	K20074	n.d
FOKN1_0055	ribonuclease PH	N	J	K00989	0.023%
FOKN1_0056	xanthosine triphosphate pyrophosphatase	N	no hit	K02428	n.d
FOKN1_0057	coproporphyrinogen III oxidase and related Fe-S oxidoreductases	N	no hit		n.d
FOKN1_0058	uncharacterized protein	N	no hit		n.d
FOKN1_0059	transcriptional antiterminator	N	no hit		n.d
FOKN1_0060	2-methylthioadenine synthetase	N	no hit	K14441	n.d
FOKN1_0061	uncharacterized protein	N	no hit		n.d
FOKN1_0062	50S ribosomal protein L31	N	no hit	K02909	n.d
FOKN1_0063	thermonuclease	Y	no hit		n.d
FOKN1_0064	Zn-dependent protease	N	no hit		0.008%
FOKN1_0065	malate dehydrogenase	N	no hit	K00029	0.009%

Table S1. Gene annotation of gene-coding sequences (CDSs) and protein expression in the *Thiohalobacter sp.* strain FOKN1 cells. SignalP; prediction of signal peptide sequence, Y; positive, N; negative, COG; clusters of orthologous group, KO; KEGG orthology annotated using the KAAS, PCI; protein content index, n.d; not detected.

Locus_tag	Product	SignalP	COG	KO	PCI (%)
FOKN1_0066	uncharacterized protein	N	no hit		n.d
FOKN1_0067	membrane carboxypeptidase/penicillin-binding protein	N	no hit	K05366	0.010%
FOKN1_0068	type IV pilus assembly protein PilM	N	no hit	K02662	0.142%
FOKN1_0069	type IV pilus assembly protein PilN	N	no hit	K02663	0.125%
FOKN1_0070	pilus assembly protein PilO	N	no hit	K02664	0.082%
FOKN1_0071	pilus assembly protein PilP	Y	no hit	K02665	0.126%
FOKN1_0072	type IV pilus assembly protein PilQ	N	no hit	K02666	0.092%
FOKN1_0073	shikimate kinase	N	no hit	K00891	n.d
FOKN1_0074	3-dehydroquinate synthase	N	no hit	K01735	0.017%
FOKN1_0075	dGTP triphosphohydrolase	N	no hit	K01129	0.021%
FOKN1_0076	uncharacterized protein	N	E		n.d
FOKN1_0077	ATPase	N	E	K03112	n.d
FOKN1_0078	glutamate synthase, large subunit	N	E	K00265	0.028%
FOKN1_0079	glutamate synthase, small subunit	N	no hit	K00266	0.015%
FOKN1_0080	uroporphyrinogen decarboxylase	N	no hit	K01599	0.037%
FOKN1_0081	signal transduction protein	N	no hit		n.d
FOKN1_0082	chloride channel protein EriC	Y	no hit		n.d
FOKN1_0083	fructosamine-3-kinase	N	no hit	K15523	n.d
FOKN1_0084	restriction endonuclease	N	no hit		n.d
FOKN1_0085	NAD/NADP transhydrogenase alpha subunit	N	C	K00324	0.158%
FOKN1_0086	NAD/NADP transhydrogenase alpha subunit	N	C	K00324	n.d
FOKN1_0087	NAD/NADP transhydrogenase beta subunit	N	C	K00325	0.038%
FOKN1_0088	phospholipase A1	Y	no hit	K01058	n.d
FOKN1_0089	DNA helicase PriA	N	no hit	K04066	n.d
FOKN1_0090	arginyl-tRNA synthetase	N	J	K01887	0.054%
FOKN1_0091	cell division protein	N	J		n.d
FOKN1_0092	tonB-dependent receptor	Y	P	K02014	0.005%
FOKN1_0093	uncharacterized protein	N	S		n.d
FOKN1_0094	uncharacterized protein	N	no hit	K06995	n.d
FOKN1_0095	2-hydroxy-3-oxopropionate reductase	N	no hit	K00020	n.d
FOKN1_0096	arginine decarboxylase	N	no hit	K01585	0.018%
FOKN1_0097	spermidine synthase	N	no hit	K00797	0.076%
FOKN1_0098	ABC-type uncharacterized transporter, permease component	N	no hit	K02069	n.d

Table S1. Gene annotation of gene-coding sequences (CDSs) and protein expression in the *Thiohalobacter sp.* strain FOKN1 cells. SignalP; prediction of signal peptide sequence, Y; positive, N; negative, COG; clusters of orthologous group, KO; KEGG orthology annotated using the KAAS, PCI; protein content index, n.d; not detected.

Locus_tag	Product	SignalP	COG	KO	PCI (%)
FOKN1_0099	ABC-type uncharacterized transporter, ATPase component	N	no hit	K02068	n.d
FOKN1_0100	TRAP-type mannitol/chloroaromatic compound transporter, periplasmic component	Y	no hit		0.052%
FOKN1_0101	signal transduction protein	N	T		0.008%
FOKN1_0102	uncharacterized protein	Y	no hit		n.d
FOKN1_0103	DNA helicase II	N	no hit	K03657	0.005%
FOKN1_0104	uncharacterized protein	Y	no hit		n.d
FOKN1_0105	N-acetyltransferase	N	no hit		n.d
FOKN1_0106	ABC-type multidrug transporter, permease component	N	no hit	K01992	n.d
FOKN1_0107	ABC-type multidrug transporter, ATPase component	N	no hit	K13926	0.013%
FOKN1_0108	secretion protein	N	no hit	K01993	0.026%
FOKN1_0109	transposase	N	no hit		n.d
FOKN1_0110	transposase	N	L	K07497	n.d
FOKN1_0111	xylanase/chitin deacetylase	N	no hit		0.026%
FOKN1_0112	extradiol ring-cleavage dioxygenase class III protein subunit B	N	no hit	K15777	n.d
FOKN1_0113	N-acetyltransferase GCN5	N	no hit		0.039%
FOKN1_0114	ferredoxin-dependent glutamate synthase	N	E	K22083	0.052%
FOKN1_0115	sensory protein	N	no hit	K05770	n.d
FOKN1_0116	transcription elongation factor	N	no hit	K06140	n.d
FOKN1_0117	threonine dehydrogenase and related Zn-dependent dehydrogenases	N	no hit		n.d
FOKN1_0118	uncharacterized protein	N	no hit	K11940	n.d
FOKN1_0119	uncharacterized protein	N	no hit	K09768	n.d
FOKN1_0120	biotin carboxylase	N	R		n.d
FOKN1_0121	acyl esterases	N	R	K06978	n.d
FOKN1_0122	cobyrinic acid a,c-diamide synthase	N	no hit		n.d
FOKN1_0123	glucan biosynthesis protein G	N	no hit	K03670	n.d
FOKN1_0124	chorismate synthase	N	no hit		n.d
FOKN1_0125	glucosyl transferase	N	no hit	K03669	n.d
FOKN1_0126	DNA polymerase III, alpha subunit	Y	no hit		n.d
FOKN1_0127	hemerythrin	N	no hit	K07216	n.d
FOKN1_0128	heme/copper-type cytochrome/quinol oxidases, subunit 1	N	no hit		n.d

Table S1. Gene annotation of gene-coding sequences (CDSs) and protein expression in the *Thiohalobacter sp.* strain FOKN1 cells. SignalP; prediction of signal peptide sequence, Y; positive, N; negative, COG; clusters of orthologous group, KO; KEGG orthology annotated using the KAAS, PCI; protein content index, n.d; not detected.

Locus_tag	Product	SignalP	COG	KO	PCI (%)
FOKN1_0129	pyrrolidone-carboxylate peptidase	N	S	K01304	n.d
FOKN1_0130	cell wall-associated hydrolases	Y	S	K19303	n.d
FOKN1_0131	signal transduction protein	N	S		n.d
FOKN1_0132	membrane protein	N	S	K22736	n.d
FOKN1_0133	uncharacterized protein	N	S		n.d
FOKN1_0134	acyl-CoA synthetase	N	C	K09181	0.013%
FOKN1_0135	deacetylases	N	C		n.d
FOKN1_0136	ATP-dependent DNA helicase RecG	N	no hit		n.d
FOKN1_0137	Fe2+/Pb2+ permease	N	no hit	K07243	n.d
FOKN1_0138	cytochrome c, mono- and diheme variants	Y	no hit		0.075%
FOKN1_0139	2-polyprenylphenol hydroxylase and related flavodoxin oxidoreductases	N	no hit		0.032%
FOKN1_0140	lPS biosynthesis protein	N	EH		n.d
FOKN1_0141	anthranilate/para-aminobenzoate synthases component I	N	EH		n.d
FOKN1_0142	transcriptional regulator	N	EH		n.d
FOKN1_0143	transcriptional regulator	N	KT		n.d
FOKN1_0144	transcriptional regulator	N	KT		0.017%
FOKN1_0145	surface antigen	N	KT		0.272%
FOKN1_0146	transporter	Y	M	K07799	0.011%
FOKN1_0147	cation/multidrug efflux pump	N	no hit	K03296	0.012%
FOKN1_0148	DNA mismatch endonuclease	N	K		n.d
FOKN1_0149	DNA-directed RNA polymerase, sigma subunit	N	K		n.d
FOKN1_0150	helicase	N	no hit		0.035%
FOKN1_0151	uncharacterized protein	N	no hit		n.d
FOKN1_0152	superfamily I DNA/RNA helicases	N	no hit		n.d
FOKN1_0153	C-5 cytosine-specific DNA methylase	N	no hit	K00558	n.d
FOKN1_0154	acyl-CoA dehydrogenase	N	T		n.d
FOKN1_0156	uncharacterized protein	N	no hit		n.d
FOKN1_0157	electron transfer flavoprotein, alpha subunit	N	no hit		n.d
FOKN1_0158	cytochrome c class I	Y	no hit		0.076%
FOKN1_0159	SAM-dependent methyltransferases	N	QR		n.d
FOKN1_0160	ATP-dependent DNA helicase	N	R	K03656	0.010%
FOKN1_0161	uncharacterized protein	N	R		n.d
FOKN1_0162	uncharacterized protein	N	no hit	K02742	n.d

Table S1. Gene annotation of gene-coding sequences (CDSs) and protein expression in the *Thiohalobacter sp.* strain FOKN1 cells. SignalP; prediction of signal peptide sequence, Y; positive, N; negative, COG; clusters of orthologous group, KO; KEGG orthology annotated using the KAAS, PCI; protein content index, n.d; not detected.

Locus_tag	Product	SignalP	COG	KO	PCI (%)
FOKN1_0163	rRNA methylase	N	no hit	K03216	n.d
FOKN1_0164	dihydroorotate dehydrogenase	N	F	K17828	n.d
FOKN1_0165	methionine sulfoxide reductase B	N	F		n.d
FOKN1_0166	MSHA biogenesis protein MshJ	N	F		n.d
FOKN1_0167	succinate dehydrogenase/fumarate reductase, flavoprotein subunit	Y	F		n.d
FOKN1_0168	type II secretory pathway, component PulD	N	no hit	K02280	n.d
FOKN1_0169	type II secretory pathway, component ExeA	N	no hit		n.d
FOKN1_0170	uncharacterized protein	N	no hit		n.d
FOKN1_0171	type II secretory pathway, ATPase PulE/TfP pilus assembly pathway, ATPase PilB	N	no hit	K02454	n.d
FOKN1_0172	type II secretory pathway, component PulF	N	no hit	K02455	0.019%
FOKN1_0173	type II secretory pathway, pseudopilin PulG	N	no hit		n.d
FOKN1_0174	type II secretory pathway, pseudopilin PulG	N	no hit		n.d
FOKN1_0175	type II secretory pathway, pseudopilin PulG	N	no hit		n.d
FOKN1_0176	uncharacterized protein	N	no hit		n.d
FOKN1_0177	uncharacterized protein	N	no hit		n.d
FOKN1_0178	biogenesis protein MshI	N	no hit		n.d
FOKN1_0179	nitrogen regulation protein	N	no hit	K07712	0.012%
FOKN1_0180	signal transduction histidine kinase	N	no hit	K07708	n.d
FOKN1_0181	uncharacterized protein	N	no hit		n.d
FOKN1_0182	glutamine synthetase	N	no hit	K01915	0.334%
FOKN1_0183	Co/Zn/Cd cation transporters	N	no hit		n.d
FOKN1_0184	tRNA 5-methylaminomethyl-2-thiouridine synthase TusA	N	J		n.d
FOKN1_0185	tRNA (5-methylaminomethyl-2-thiouridylate)-methyltransferase	N	J	K00566	0.019%
FOKN1_0186	translation initiation inhibitor	N	J		n.d
FOKN1_0187	uncharacterized protein	N	no hit		n.d
FOKN1_0188	glycyl-tRNA synthetase subunit alpha	N	J	K01878	0.044%
FOKN1_0189	glycine-tRNA ligase	N	J	K01879	0.007%
FOKN1_0190	pyrophosphatase	N	R		n.d
FOKN1_0191	D,D-heptose 1,7-bisphosphate phosphatase	N	R	K03273	n.d
FOKN1_0192	phospholipid/glycerol acyltransferase	N	no hit	K00655	n.d
FOKN1_0193	DNA gyrase subunit B	N	no hit	K02470	0.050%

Table S1. Gene annotation of gene-coding sequences (CDSs) and protein expression in the *Thiohalobacter sp.* strain FOKN1 cells. SignalP; prediction of signal peptide sequence, Y; positive, N; negative, COG; clusters of orthologous group, KO; KEGG orthology annotated using the KAAS, PCI; protein content index, n.d; not detected.

Locus_tag	Product	SignalP	COG	KO	PCI (%)
FOKN1_0194	DNA replication and repair protein RecF	N	no hit	K03629	n.d
FOKN1_0195	DNA polymerase III subunit beta	N	no hit	K02338	0.155%
FOKN1_0196	chromosome replication initiator DnaA	N	no hit	K02313	n.d
FOKN1_0197	50S ribosomal protein L34	N	no hit		n.d
FOKN1_0198	protein C5 component of RNase P	N	no hit	K03536	n.d
FOKN1_0199	putative membrane protein insertion efficiency factor	N	no hit	K08998	n.d
FOKN1_0200	membrane protein insertase YidC	N	no hit	K03217	n.d
FOKN1_0201	tRNA modification GTPase TrmE	N	no hit	K03650	n.d
FOKN1_0202	phage integrase	N	L		n.d
FOKN1_0203	anti-sigma factor	N	T		n.d
FOKN1_0204	phage transcriptional regulator, AlpA	N	no hit		n.d
FOKN1_0205	GTPase SAR1	N	no hit		n.d
FOKN1_0206	ATP-dependent Zn proteases	N	no hit		n.d
FOKN1_0207	N-6 DNA methylase	N	V		n.d
FOKN1_0208	restriction endonuclease S subunits	N	V	K01154	n.d
FOKN1_0209	transcriptional regulator	N	V		n.d
FOKN1_0210	type I site-specific restriction-modification system, R subunit	N	V	K01153	n.d
FOKN1_0211	CMP/dCMP deaminase zinc-binding protein	N	no hit	K01493	0.048%
FOKN1_0212	cytochrome b561	N	no hit		n.d
FOKN1_0213	uncharacterized protein	Y	no hit		n.d
FOKN1_0214	peptidylprolyl isomerase	N	O	K03775	n.d
FOKN1_0215	transglutaminase	N	no hit	K22452	n.d
FOKN1_0216	uncharacterized protein	N	no hit		n.d
FOKN1_0217	moxR-like ATPase	N	no hit	K03924	n.d
FOKN1_0218	homocysteine S-methyltransferase	N	no hit		n.d
FOKN1_0219	araC family transcriptional regulator	N	no hit		n.d
FOKN1_0220	tRNA uridine 5-carboxymethylaminomethyl modification protein GidA	N	no hit	K03495	n.d
FOKN1_0221	ribosomal RNA small subunit methyltransferase G	N	no hit	K03501	0.082%
FOKN1_0222	parA family protein	N	no hit	K03496	n.d
FOKN1_0223	parB-like nuclease domain family	N	no hit	K03497	0.182%
FOKN1_0224	ATP synthase I chain	N	no hit		n.d
FOKN1_0225	ATP synthase F0F1 subunit A	N	no hit	K02108	n.d

Table S1. Gene annotation of gene-coding sequences (CDSs) and protein expression in the *Thiohalobacter sp.* strain FOKN1 cells. SignalP; prediction of signal peptide sequence, Y; positive, N; negative, COG; clusters of orthologous group, KO; KEGG orthology annotated using the KAAS, PCI; protein content index, n.d; not detected.

Locus_tag	Product	SignalP	COG	KO	PCI (%)
FOKN1_0226	ATP synthase F0F1 subunit C	N	C	K02110	n.d
FOKN1_0227	ATP synthase subunit B	N	no hit	K02109	1.600%
FOKN1_0228	ATP synthase subunit delta	N	no hit	K02113	0.107%
FOKN1_0229	F1 sector of membrane-bound ATP synthase, alpha subunit	N	C	K02111	0.354%
FOKN1_0230	H(+)-transporting two-sector ATPase	N	C	K02115	0.086%
FOKN1_0231	F1 sector of membrane-bound ATP synthase, beta subunit	N	no hit	K02112	0.196%
FOKN1_0232	ATP synthase F1 subunit epsilon	N	no hit	K02114	0.033%
FOKN1_0233	peptidyl-prolyl cis-trans isomerase	N	no hit	K03775	n.d
FOKN1_0234	UDP-N-acetylglucosamine pyrophosphorylase	N	M	K04042	n.d
FOKN1_0235	glucosamine/fructose-6-phosphate aminotransferase	N	M	K00820	0.049%
FOKN1_0236	restriction/modification methyltransferase	N	no hit	K03427	n.d
FOKN1_0237	response regulator	N	no hit		n.d
FOKN1_0238	uncharacterized protein	N	no hit		n.d
FOKN1_0239	polyribonucleotide nucleotidyltransferase	N	no hit		n.d
FOKN1_0240	DNA/RNA helicases	N	no hit		n.d
FOKN1_0241	restriction endonuclease	N	no hit		n.d
FOKN1_0242	uncharacterized protein	N	no hit		n.d
FOKN1_0243	uncharacterized protein	N	no hit		n.d
FOKN1_0244	glutamine synthetase	N	M		n.d
FOKN1_0245	uncharacterized protein	N	M		n.d
FOKN1_0246	transcriptional regulator	N	M		n.d
FOKN1_0247	glucosamine-fructose-6-phosphate aminotransferase	N	M		n.d
FOKN1_0248	restriction endonuclease	N	no hit	K07454	n.d
FOKN1_0249	uncharacterized protein	N	no hit		n.d
FOKN1_0250	uncharacterized protein	N	no hit		n.d
FOKN1_0251	uncharacterized protein	N	no hit		n.d
FOKN1_0252	cAMP-binding proteins	N	T		n.d
FOKN1_0253	transcriptional regulator	N	no hit		n.d
FOKN1_0254	uncharacterized protein	N	no hit		n.d
FOKN1_0255	L-lysine 2,3-aminomutase	N	no hit		n.d
FOKN1_0256	carbohydrate kinase	N	G	K00847	0.018%
FOKN1_0257	sucrose-phosphate synthase	N	no hit	K00696	0.022%
FOKN1_0258	sucrose synthase	N	M	K00695	0.011%

Table S1. Gene annotation of gene-coding sequences (CDSs) and protein expression in the *Thiohalobacter sp.* strain FOKN1 cells. SignalP; prediction of signal peptide sequence, Y; positive, N; negative, COG; clusters of orthologous group, KO; KEGG orthology annotated using the KAAS, PCI; protein content index, n.d; not detected.

Locus_tag	Product	SignalP	COG	KO	PCI (%)
FOKN1_0259	uncharacterized protein	N	no hit		n.d
FOKN1_0260	NADPH-dependent FMN reductase	N	no hit		0.100%
FOKN1_0261	uncharacterized protein	N	no hit		n.d
FOKN1_0262	prolyl-tRNA synthetase	N	no hit		n.d
FOKN1_0263	ABC transporter	N	no hit	K06158	n.d
FOKN1_0264	carbonic anhydrases/acetyltransferases	N	no hit		n.d
FOKN1_0265	uncharacterized protein	N	no hit		n.d
FOKN1_0266	uncharacterized protein	N	P		0.085%
FOKN1_0267	uncharacterized protein	Y	no hit		n.d
FOKN1_0268	pyruvate/2-oxoglutarate dehydrogenase complex	Y	no hit		0.037%
FOKN1_0269	shikimate 5-dehydrogenase	N	no hit	K00014	0.021%
FOKN1_0270	delta-aminolevulinic acid dehydratase	N	no hit	K01698	0.047%
FOKN1_0271	non-ribosomal peptide synthetase	N	no hit		n.d
FOKN1_0272	ABC-type transporter, auxiliary component	N	no hit	K07323	n.d
FOKN1_0273	rubredoxin	N	no hit		n.d
FOKN1_0274	coproporphyrinogen III oxidase	N	no hit	K00228	0.024%
FOKN1_0275	diguanylate cyclase	N	T	K13590	n.d
FOKN1_0276	threonylcarbamoyl-AMP synthase	N	T	K07566	n.d
FOKN1_0277	uncharacterized protein	Y	no hit		n.d
FOKN1_0278	ammonium transporter	N	no hit	K03320	n.d
FOKN1_0279	nitrogen regulatory protein PII	N	no hit	K04752	n.d
FOKN1_0280	uncharacterized protein	N	no hit	K09806	n.d
FOKN1_0281	Mg chelatase subunit ChlI	N	no hit	K07391	n.d
FOKN1_0282	uncharacterized protein	N	no hit		n.d
FOKN1_0283	NAD(P)H-dependent glycerol-3-phosphate dehydrogenase	N	no hit	K00057	0.027%
FOKN1_0284	protein-export protein SecB	N	no hit	K03071	0.027%
FOKN1_0285	glutaredoxin, GrxC family	N	O	K03676	n.d
FOKN1_0286	rhodanese-like protein	N	O	K03972	0.065%
FOKN1_0287	transcriptional regulator	N	no hit		0.118%
FOKN1_0288	ribonucleoside-diphosphate reductase	N	no hit		n.d
FOKN1_0289	phosphoglycerate mutase	N	no hit	K15633	0.043%
FOKN1_0290	peptidase M23	Y	no hit	K22719	n.d
FOKN1_0291	carboxyl-terminal protease	N	no hit	K03797	0.089%

Table S1. Gene annotation of gene-coding sequences (CDSs) and protein expression in the *Thiohalobacter sp.* strain FOKN1 cells. SignalP; prediction of signal peptide sequence, Y; positive, N; negative, COG; clusters of orthologous group, KO; KEGG orthology annotated using the KAAS, PCI; protein content index, n.d; not detected.

Locus_tag	Product	SignalP	COG	KO	PCI (%)
FOKN1_0292	uncharacterized protein	Y	no hit	K09798	n.d
FOKN1_0293	putative intracellular protease/amidase	N	no hit	K03152	n.d
FOKN1_0294	2-polyprenylphenol 6-hydroxylase	N	no hit	K03688	0.013%
FOKN1_0295	sterol-binding domain protein	N	no hit	K03690	0.019%
FOKN1_0296	membrane protein	N	no hit		n.d
FOKN1_0297	ubiquinone/menaquinone biosynthesis methyltransferase	N	R	K03183	0.034%
FOKN1_0298	putative permease	N	R	K07090	0.045%
FOKN1_0299	putative multicopper oxidases	N	no hit		n.d
FOKN1_0300	transcriptional regulator	N	no hit		n.d
FOKN1_0301	uncharacterized protein	N	no hit		n.d
FOKN1_0302	ATP-dependent protease HslVU (ClpYQ), ATPase subunit	N	no hit	K03667	0.053%
FOKN1_0303	ATP-dependent protease HslVU (ClpYQ), peptidase subunit	N	no hit	K01419	0.039%
FOKN1_0304	tyrosine recombinase XerC	N	no hit	K03733	0.012%
FOKN1_0305	uncharacterized protein	N	no hit	K09921	n.d
FOKN1_0306	diaminopimelate epimerase	N	no hit	K01778	0.047%
FOKN1_0307	diguanylate cyclase	N	T		n.d
FOKN1_0308	diaminopimelate decarboxylase	N	no hit	K01586	0.020%
FOKN1_0309	lipoprotein	N	no hit		n.d
FOKN1_0310	esterase	N	no hit	K06999	n.d
FOKN1_0311	cytochrome c, mono- and diheme variants	Y	no hit		0.016%
FOKN1_0312	uncharacterized protein	N	no hit		n.d
FOKN1_0313	transposase	N	no hit	K07491	n.d
FOKN1_0314	transcriptional regulators	N	no hit		n.d
FOKN1_0315	uncharacterized protein	N	no hit		n.d
FOKN1_0316	adenylate cyclase	N	no hit	K05851	n.d
FOKN1_0317	signal transduction protein	N	no hit		n.d
FOKN1_0318	nucleotide pyrophosphohydrolase	N	no hit	K16904	n.d
FOKN1_0319	outer membrane protein	N	M		n.d
FOKN1_0320	signal transduction protein	N	no hit		n.d
FOKN1_0321	argininosuccinate lyase	N	no hit	K01755	0.036%
FOKN1_0322	sensory transduction protein kinase AlgZ	N	no hit	K08082	n.d
FOKN1_0323	response regulator receiver protein	N	no hit	K08083	0.015%

Table S1. Gene annotation of gene-coding sequences (CDSs) and protein expression in the *Thiohalobacter sp.* strain FOKN1 cells. SignalP; prediction of signal peptide sequence, Y; positive, N; negative, COG; clusters of orthologous group, KO; KEGG orthology annotated using the KAAS, PCI; protein content index, n.d; not detected.

Locus_tag	Product	SignalP	COG	KO	PCI (%)
FOKN1_0324	thiol-disulfide isomerase and thioredoxins	Y	no hit		n.d
FOKN1_0325	porphobilinogen deaminase	N	no hit	K01749	0.032%
FOKN1_0326	uroporphyrinogen-III synthase	N	no hit	K01719	0.032%
FOKN1_0327	uroporphyrin-III C-methyltransferase	N	no hit	K02496	0.092%
FOKN1_0328	protoporphyrinogen IX and coproporphyrinogen III oxidase HemY	N	no hit	K02498	0.028%
FOKN1_0329	2-polyprenylphenol hydroxylase and related flavodoxin oxidoreductases	N	no hit	K00523	0.010%
FOKN1_0330	nucleoside-diphosphate-sugar epimerases	N	no hit		n.d
FOKN1_0331	3-octaprenyl-4-hydroxybenzoate carboxylyase	N	no hit	K03182	0.011%
FOKN1_0332	Zn-dependent oligopeptidases	N	no hit	K01414	0.082%
FOKN1_0333	glutathione-disulfide reductase	N	no hit	K00383	0.026%
FOKN1_0334	putative Fe-S protein	N	no hit		n.d
FOKN1_0335	ribonuclease D	N	no hit	K03684	n.d
FOKN1_0336	D-Tyr-tRNAtyr deacylase	N	no hit	K07560	n.d
FOKN1_0337	proline iminopeptidase	N	no hit	K01259	0.017%
FOKN1_0338	uncharacterized protein	N	S		0.660%
FOKN1_0339	rhodanese-related sulfurtransferase	N	no hit		n.d
FOKN1_0340	uncharacterized protein	N	no hit	K14058	n.d
FOKN1_0341	lauroyl/myristoyl acyltransferase	N	no hit	K02517	n.d
FOKN1_0342	uncharacterized protein	N	no hit		n.d
FOKN1_0343	uncharacterized protein	Y	no hit		n.d
FOKN1_0344	polyketide synthase	N	no hit		n.d
FOKN1_0345	sodium/hydrogen exchanger	N	no hit	K11105	n.d
FOKN1_0346	permeases	N	GEPR	K08218	n.d
FOKN1_0347	uncharacterized protein	N	no hit		n.d
FOKN1_0348	Trk-type K+ transporter	N	no hit	K03498	n.d
FOKN1_0349	Trk-type K+ transporter	N	no hit	K03499	0.023%
FOKN1_0350	response regulator	N	T	K13599	0.035%
FOKN1_0351	signal transduction histidine kinase	N	no hit		n.d
FOKN1_0352	uncharacterized protein	Y	no hit		n.d
FOKN1_0353	tRNA and rRNA cytosine-C5-methylases	N	no hit	K03500	0.008%
FOKN1_0354	methionyl-tRNA formyltransferase	N	no hit	K00604	0.020%
FOKN1_0355	N-formylmethionyl-tRNA deformylase	N	no hit	K01462	0.020%

Table S1. Gene annotation of gene-coding sequences (CDSs) and protein expression in the *Thiohalobacter sp.* strain FOKN1 cells. SignalP; prediction of signal peptide sequence, Y; positive, N; negative, COG; clusters of orthologous group, KO; KEGG orthology annotated using the KAAS, PCI; protein content index, n.d; not detected.

Locus_tag	Product	SignalP	COG	KO	PCI (%)
FOKN1_0356	uncharacterized protein	Y	no hit		0.052%
FOKN1_0357	DNA protecting protein DprA	N	no hit	K04096	n.d
FOKN1_0358	uncharacterized protein	N	no hit	K03747	n.d
FOKN1_0359	DNA topoisomerase I	N	no hit	K03168	0.060%
FOKN1_0360	uncharacterized protein	N	T		n.d
FOKN1_0361	TRAP-type mannitol/chloroaromatic compound transporter, large permease component	N	no hit		n.d
FOKN1_0362	TRAP-type mannitol/chloroaromatic compound transporter, small permease component	N	no hit		n.d
FOKN1_0363	uncharacterized protein	Y	no hit		n.d
FOKN1_0364	DNA processing protein A	N	no hit	K06966	0.023%
FOKN1_0365	DNA polymerase I	N	no hit	K02335	0.010%
FOKN1_0366	GTPase	N	no hit	K03978	n.d
FOKN1_0367	cytochrome c subfamily	Y	no hit		0.124%
FOKN1_0368	resB family protein	N	no hit	K07399	0.010%
FOKN1_0369	cytochrome c assembly protein	N	no hit		n.d
FOKN1_0370	thiol:disulfide interchange protein	Y	no hit	K03673	0.036%
FOKN1_0371	acetolactate synthase	N	no hit	K01652	0.053%
FOKN1_0372	metal-dependent hydrolase	N	no hit		n.d
FOKN1_0373	uncharacterized protein	N	no hit	K13590	n.d
FOKN1_0374	glutathione S-transferase	N	O	K07393	n.d
FOKN1_0375	signal transduction protein	N	no hit		n.d
FOKN1_0376	uncharacterized protein	N	no hit		n.d
FOKN1_0377	Sec-independent protein translocase protein TatC	N	no hit	K03118	n.d
FOKN1_0378	Sec-independent protein translocase protein TatB	N	no hit	K03117	0.130%
FOKN1_0379	twin arginine-targeting protein translocase	N	no hit		n.d
FOKN1_0380	phosphoribosyl-ATP pyrophosphatase	N	no hit	K01523	n.d
FOKN1_0381	phosphoribosyl-AMP cyclohydrolase	N	no hit	K01496	0.063%
FOKN1_0382	imidazole glycerol phosphate synthase subunit HisF	N	no hit	K02500	n.d
FOKN1_0383	1-(5-phosphoribosyl)-5-[(5-phosphoribosylamino) methylideneamino]imidazole-4-carboxamide isomerase	N	no hit	K01814	n.d
FOKN1_0384	imidazole glycerol phosphate synthase subunit HisH	N	no hit	K02501	n.d
FOKN1_0385	imidazoleglycerol-phosphate dehydratase	N	no hit	K01693	0.109%
FOKN1_0386	Crp/Fnr family transcriptional regulator	N	no hit	K01420	0.014%

Table S1. Gene annotation of gene-coding sequences (CDSs) and protein expression in the *Thiohalobacter sp.* strain FOKN1 cells. SignalP; prediction of signal peptide sequence, Y; positive, N; negative, COG; clusters of orthologous group, KO; KEGG orthology annotated using the KAAS, PCI; protein content index, n.d; not detected.

Locus_tag	Product	SignalP	COG	KO	PCI (%)
FOKN1_0387	cbb3-type cytochrome c oxidase subunit I	N	no hit	K00404	0.017%
FOKN1_0388	cbb3-type cytochrome c oxidase subunit II	N	no hit	K00405	n.d
FOKN1_0389	cytochrome c oxidase, cbb3-type, CcoQ subunit	N	no hit	K00407	n.d
FOKN1_0390	cytochrome c oxidase, cbb3-type subunit III	N	no hit	K00406	0.072%
FOKN1_0391	polyferredoxin	N	no hit		n.d
FOKN1_0392	uncharacterized protein	N	no hit	K09926	n.d
FOKN1_0393	uncharacterized protein	N	no hit		n.d
FOKN1_0394	ABC-type multidrug transporter, permease component	N	no hit	K01992	n.d
FOKN1_0395	ABC-type multidrug transporter, ATPase component	N	no hit	K01990	n.d
FOKN1_0396	uncharacterized protein	Y	no hit		n.d
FOKN1_0397	two-component system regulatory protein	N	no hit	K07660	n.d
FOKN1_0398	putative histidine kinase	N	no hit	K07637	n.d
FOKN1_0399	glutaredoxin	N	no hit		n.d
FOKN1_0400	uncharacterized protein	N	no hit		n.d
FOKN1_0401	rhodanese domain protein	N	no hit		0.045%
FOKN1_0402	histidine kinase	N	no hit		n.d
FOKN1_0403	signal transduction histidine kinase	N	no hit		n.d
FOKN1_0404	molybdenum cofactor biosynthesis protein B	N	no hit	K03638	0.025%
FOKN1_0405	serine protease	N	no hit		n.d
FOKN1_0406	ribosomal protein S6 glutaminyl transferase	N	HJ		n.d
FOKN1_0407	uncharacterized protein	N	no hit		n.d
FOKN1_0408	permeases	N	no hit		n.d
FOKN1_0409	uncharacterized protein	N	no hit		n.d
FOKN1_0410	TraR/DksA family transcriptional regulator	N	no hit		n.d
FOKN1_0411	diguanylate cyclase	N	no hit	K18968	n.d
FOKN1_0412	chemotaxis protein CheC	N	no hit		n.d
FOKN1_0413	amidohydrolase 2	Y	no hit		n.d
FOKN1_0414	excinuclease ABC C subunit	N	no hit		n.d
FOKN1_0415	membrane protein	N	no hit	K03286	n.d
FOKN1_0416	glutamate dehydrogenase/leucine dehydrogenase	N	no hit	K00261	0.012%
FOKN1_0417	NADPH:quinone reductase	N	no hit		n.d
FOKN1_0418	uncharacterized protein	Y	S		n.d
FOKN1_0419	cold shock proteins	N	no hit	K03704	n.d
FOKN1_0420	phosphoenolpyruvate synthase/pyruvate phosphate	N	no hit		n.d

Table S1. Gene annotation of gene-coding sequences (CDSs) and protein expression in the *Thiohalobacter sp.* strain FOKN1 cells. SignalP; prediction of signal peptide sequence, Y; positive, N; negative, COG; clusters of orthologous group, KO; KEGG orthology annotated using the KAAS, PCI; protein content index, n.d; not detected.

Locus_tag	Product	SignalP	COG	KO	PCI (%)
	dikinase				
FOKN1_0421	integral membrane protein	N	no hit		n.d
FOKN1_0422	fructose-2,6-bisphosphatase	Y	no hit		n.d
FOKN1_0423	methionine synthase	N	no hit	K00548	n.d
FOKN1_0424	nitrous oxide reductase maturation protein NosR	N	no hit	K19339	0.005%
FOKN1_0425	nitrous-oxide reductase	N	no hit	K00376	0.134%
FOKN1_0426	nitrous oxide reductase maturation protein NosD	N	no hit	K07218	n.d
FOKN1_0427	nitrous oxide reductase maturation protein NosF	N	no hit	K19340	n.d
FOKN1_0428	nitrous oxide reductase maturation transmembrane protein NosY	N	no hit	K19341	n.d
FOKN1_0429	biotin carboxylase	N	no hit		n.d
FOKN1_0430	protein disulfide isomerase NosL	N	no hit	K19342	n.d
FOKN1_0431	disulfide bond formation protein DsbB	N	no hit		n.d
FOKN1_0432	uncharacterized protein	N	no hit		n.d
FOKN1_0433	uncharacterized protein	N	no hit		n.d
FOKN1_0434	lysine exporter protein	N	no hit		n.d
FOKN1_0435	RNA polymerase sigma-70 subunit RpoD	N	no hit	K03086	0.038%
FOKN1_0436	DNA primase	N	no hit	K02316	0.006%
FOKN1_0437	uncharacterized protein	N	no hit	K09117	n.d
FOKN1_0438	30S ribosomal protein S21	N	no hit	K02970	0.322%
FOKN1_0439	metal-dependent proteases	N	no hit	K01409	n.d
FOKN1_0440	acyl-phosphate glycerol 3-phosphateacyltransferase	N	no hit	K08591	n.d
FOKN1_0441	dihydroneopterin aldolase	N	no hit	K01633	0.061%
FOKN1_0442	2-amino-4-hydroxy-6-hydroxymethyldihydropteridine diphosphokinase	N	no hit	K00950	n.d
FOKN1_0443	uncharacterized protein	N	no hit		n.d
FOKN1_0444	undecaprenyl pyrophosphate phosphatase	N	no hit	K06153	n.d
FOKN1_0445	modular polyketide synthase BFAS2	N	no hit		n.d
FOKN1_0446	tRNA nucleotidyltransferase	N	no hit	K00974	n.d
FOKN1_0447	NAD-dependent epimerase/dehydratase	N	no hit	K00356	n.d
FOKN1_0448	formate hydrogenlyase subunit 3/Multisubunit Na+ /H+ antiporter, MnhD subunit	N	CP		n.d
FOKN1_0449	formate hydrogenlyase subunit 3/Multisubunit Na+ /H+ antiporter, MnhD subunit	N	CP	K05568	n.d

Table S1. Gene annotation of gene-coding sequences (CDSs) and protein expression in the *Thiohalobacter sp.* strain FOKN1 cells. SignalP; prediction of signal peptide sequence, Y; positive, N; negative, COG; clusters of orthologous group, KO; KEGG orthology annotated using the KAAS, PCI; protein content index, n.d; not detected.

Locus_tag	Product	SignalP	COG	KO	PCI (%)
FOKN1_0450	formate hydrogenlyase subunit 3/Multisubunit Na+ /H+ antiporter, MnhD subunit	N	no hit	K00343	n.d
FOKN1_0451	multisubunit Na+/H+ antiporter, MnhC subunit	N	no hit		n.d
FOKN1_0452	multisubunit Na+/H+ antiporter, MnhB subunit	N	P		n.d
FOKN1_0453	multisubunit Na+/H+ antiporter, MnhG subunit	N	P		n.d
FOKN1_0454	multisubunit Na+/H+ antiporter, MnhF subunit	N	P		n.d
FOKN1_0455	multisubunit Na+/H+ antiporter, MnhE subunit	N	no hit		n.d
FOKN1_0456	soluble lytic murein transglycosylase	Y	no hit	K08309	n.d
FOKN1_0457	succinate-semialdehyde dehydrogenase	N	no hit	K00135	0.108%
FOKN1_0458	putative permease	N	no hit	K07090	n.d
FOKN1_0459	cytochrome cd1 nitrite reductase	Y	no hit	K15864	0.085%
FOKN1_0460	uroporphyrinogen-III methylase	N	no hit	K13542	n.d
FOKN1_0461	cytochrome c, mono- and diheme variants	Y	no hit	K19344	n.d
FOKN1_0462	cytochrome d1 heme region	N	no hit	K19345	n.d
FOKN1_0463	asnC family transcriptional regulator	N	no hit		n.d
FOKN1_0464	heme d1 biosynthesis protein NirL	N	no hit		n.d
FOKN1_0465	transcriptional regulator protein NirG	N	no hit		n.d
FOKN1_0466	transcriptional regulator	N	no hit		n.d
FOKN1_0467	heme d1 biosynthesis protein NirJ	N	no hit		n.d
FOKN1_0468	nitrite reductase	Y	no hit		n.d
FOKN1_0469	carbamoylphosphate synthase large subunit	N	no hit		n.d
FOKN1_0470	Crp/Fnr family transcriptional regulator Dnr	N	no hit	K21563	0.092%
FOKN1_0471	nitric-oxide reductase subunit C	N	no hit	K02305	n.d
FOKN1_0472	nitric oxide reductase, NorB subunit apoprotein	N	no hit	K04561	n.d
FOKN1_0473	additional subunit of nitric oxide reductase complex membrane protein	N	no hit	K02164	n.d
FOKN1_0474	uncharacterized protein	N	no hit		n.d
FOKN1_0475	ATPase	N	no hit	K04748	n.d
FOKN1_0476	uncharacterized protein	N	C		n.d
FOKN1_0477	polyferredoxin	N	C		n.d
FOKN1_0478	nitric oxide reductase activation protein	N	no hit	K02448	n.d
FOKN1_0479	uncharacterized protein involved in response to NO	N	no hit	K07234	n.d
FOKN1_0480	radical SAM protein	N	no hit	K07139	n.d
FOKN1_0481	collagenase-like protease	N	no hit	K08303	n.d

Table S1. Gene annotation of gene-coding sequences (CDSs) and protein expression in the *Thiohalobacter sp.* strain FOKN1 cells. SignalP; prediction of signal peptide sequence, Y; positive, N; negative, COG; clusters of orthologous group, KO; KEGG orthology annotated using the KAAS, PCI; protein content index, n.d; not detected.

Locus_tag	Product	SignalP	COG	KO	PCI (%)
FOKN1_0482	collagenase-like protease	N	no hit		n.d
FOKN1_0483	K+ transporter	N	no hit		0.025%
FOKN1_0484	uncharacterized protein	N	no hit		n.d
FOKN1_0485	uncharacterized protein	Y	no hit		n.d
FOKN1_0486	sterol-binding protein	N	no hit		n.d
FOKN1_0487	glutamine amidotransferase subunit PdxT	N	no hit	K08681	n.d
FOKN1_0488	pyridoxal 5-phosphate synthase, synthase subunit Pdx1	N	H	K06215	n.d
FOKN1_0489	molybdenum cofactor biosynthesis protein A	N	C	K03639	n.d
FOKN1_0490	DMSO reductase anchor subunit	N	C	K21309	n.d
FOKN1_0491	4Fe-4S ferredoxin iron-sulfur binding domain protein	N	C	K21308	n.d
FOKN1_0492	molybdopterin oxidoreductase family protein	N	C	K21307	0.029%
FOKN1_0493	phosphate ABC transporter, periplasmic phosphate-binding protein	Y	G	K02040	n.d
FOKN1_0494	ribulose-bisphosphate carboxylase	N	G	K01601	0.537%
FOKN1_0495	ribulose-bisphosphate carboxylase	N	C	K01602	0.455%
FOKN1_0496	ATPase	N	no hit	K14572	0.086%
FOKN1_0497	rubisco activation protein CbbO	N	P		0.025%
FOKN1_0498	methyltransferase	N	no hit	K00564	n.d
FOKN1_0499	signal transduction protein	N	no hit		n.d
FOKN1_0500	transcriptional regulator	N	no hit	K21711	n.d
FOKN1_0501	NADPH-glutathione reductase	N	C	K00383	n.d
FOKN1_0502	glutaredoxin family protein	N	no hit	K03386	n.d
FOKN1_0503	phosphoribosylaminoimidazole carboxylase ATPase subunit	N	no hit	K01589	0.010%
FOKN1_0504	phosphoribosylaminoimidazole carboxylase catalytic subunit	N	F	K01588	0.038%
FOKN1_0505	phosphoribosylaminoimidazole-succinocarboxamide synthase	N	no hit	K01923	0.019%
FOKN1_0506	acyl-CoA synthetases (AMP-forming)/AMP-acid ligases II	Y	no hit		n.d
FOKN1_0507	Cu(I)/Ag(I) efflux system outer membrane protein CusC	N	no hit		0.075%
FOKN1_0508	membrane fusion protein	N	no hit	K07798	n.d
FOKN1_0509	putative silver efflux pump	N	no hit	K07787	n.d
FOKN1_0510	5,10-methylene-tetrahydrofolate dehydrogenase/methenyl	N	no hit		n.d

Table S1. Gene annotation of gene-coding sequences (CDSs) and protein expression in the *Thiohalobacter sp.* strain FOKN1 cells. SignalP; prediction of signal peptide sequence, Y; positive, N; negative, COG; clusters of orthologous group, KO; KEGG orthology annotated using the KAAS, PCI; protein content index, n.d; not detected.

Locus_tag	Product	SignalP	COG	KO	PCI (%)
	tetrahydrofolate cyclohydrolase				
FOKN1_0511	nitrogen regulatory protein	N	no hit		n.d
FOKN1_0512	uncharacterized protein	Y	no hit		n.d
FOKN1_0513	uncharacterized protein	N	no hit		n.d
FOKN1_0514	thiol-disulfide isomerase and thioredoxins	N	P		n.d
FOKN1_0515	copper-translocating P-type ATPase	N	P	K01533	0.006%
FOKN1_0516	permeases of the drug/metabolite transporter	N	GER		n.d
FOKN1_0517	drug exporter	Y	no hit		0.081%
FOKN1_0518	uncharacterized protein	N	no hit		n.d
FOKN1_0519	dithiol-disulfide isomeras	N	no hit		n.d
FOKN1_0520	cation diffusion facilitator family transporter	N	Q	K16264	n.d
FOKN1_0521	copper resistance protein A	N	Q		0.015%
FOKN1_0522	copper resistance protein B	Y	no hit	K07233	n.d
FOKN1_0523	uncharacterized protein	N	no hit		n.d
FOKN1_0524	uncharacterized protein	N	no hit		n.d
FOKN1_0525	ABC-type amino acid transport/signal transduction systems, periplasmic component	Y	F		n.d
FOKN1_0526	putative signal transduction protein	N	F		n.d
FOKN1_0527	GMP synthase	N	F		n.d
FOKN1_0528	uncharacterized protein	N	no hit		n.d
FOKN1_0529	copper-exporting P-type ATPase A	N	no hit	K17686	n.d
FOKN1_0530	DNA-directed RNA polymerase	N	no hit		n.d
FOKN1_0531	uncharacterized protein	N	no hit		n.d
FOKN1_0532	lipoprotein	N	no hit		n.d
FOKN1_0533	NADPH:quinone reductase and related Zn-dependent oxidoreductases	N	no hit		n.d
FOKN1_0534	aconitate hydratase	Y	M		n.d
FOKN1_0535	uncharacterized protein	N	M		0.085%
FOKN1_0536	uncharacterized protein	Y	M		0.041%
FOKN1_0537	heavy metal efflux pump CzcA	N	T		0.071%
FOKN1_0538	multi-sensor signal transduction histidine kinase	N	T	K07698	0.008%
FOKN1_0539	two component transcriptional regulator	N	T		0.030%
FOKN1_0540	thiol-disulfide isomerase and thioredoxins	N	OC		0.509%
FOKN1_0541	thiocyanate dehydrogenase	Y	R		2.455%

Table S1. Gene annotation of gene-coding sequences (CDSs) and protein expression in the *Thiohalobacter sp.* strain FOKN1 cells. SignalP; prediction of signal peptide sequence, Y; positive, N; negative, COG; clusters of orthologous group, KO; KEGG orthology annotated using the KAAS, PCI; protein content index, n.d; not detected.

Locus_tag	Product	SignalP	COG	KO	PCI (%)
FOKN1_0542	ATPase	Y	R		0.268%
FOKN1_0543	uncharacterized protein	N	S	K21919	0.103%
FOKN1_0544	signal transduction protein	N	no hit		0.012%
FOKN1_0545	molybdopterin biosynthesis enzyme	N	H		n.d
FOKN1_0546	heme A synthase	N	O	K02259	n.d
FOKN1_0547	putative threonine efflux protein	Y	no hit		n.d
FOKN1_0548	aldo/keto reductase	N	no hit		0.024%
FOKN1_0549	uncharacterized protein	N	no hit		n.d
FOKN1_0550	phosphoribulokinase	N	no hit	K00855	0.246%
FOKN1_0551	tRNA (guanine-N(7)-)-methyltransferase	N	no hit	K03439	n.d
FOKN1_0552	uncharacterized protein	N	no hit	K05802	n.d
FOKN1_0553	Mg2+ transporter protein	N	no hit	K03284	n.d
FOKN1_0554	thiazole biosynthesis family protein	N	H	K03149	n.d
FOKN1_0555	thiamine biosynthesis protein	N	no hit	K03154	n.d
FOKN1_0556	AMP-forming long-chain acyl-CoA synthetase	N	no hit	K01897	n.d
FOKN1_0557	uncharacterized protein	N	S		0.019%
FOKN1_0558	uncharacterized protein	N	S		n.d
FOKN1_0559	transcriptional regulator	N	T		n.d
FOKN1_0560	uncharacterized protein	Y	NU		n.d
FOKN1_0562	type II secretory pathway protein	N	NU	K02652	n.d
FOKN1_0563	uncharacterized protein	N	no hit		0.022%
FOKN1_0564	uncharacterized protein	N	no hit	K07289	0.130%
FOKN1_0565	adenine glycosylase	N	no hit	K03575	n.d
FOKN1_0566	Fe(2+) trafficking protein	N	no hit		n.d
FOKN1_0568	phosphomannomutase	N	G	K01840	n.d
FOKN1_0569	uncharacterized protein	N	no hit		n.d
FOKN1_0570	uncharacterized protein	N	no hit		n.d
FOKN1_0571	transcriptional regulator	N	no hit		n.d
FOKN1_0572	uncharacterized protein	N	no hit		n.d
FOKN1_0573	bifunctional sulfate adenylyltransferase subunit 1/adenylylsulfate kinase	N	no hit	K00958	0.038%
FOKN1_0574	uncharacterized protein	N	no hit		n.d
FOKN1_0575	uncharacterized protein	N	no hit		n.d
FOKN1_0576	uncharacterized protein	N	S		n.d

Table S1. Gene annotation of gene-coding sequences (CDSs) and protein expression in the *Thiohalobacter sp.* strain FOKN1 cells. SignalP; prediction of signal peptide sequence, Y; positive, N; negative, COG; clusters of orthologous group, KO; KEGG orthology annotated using the KAAS, PCI; protein content index, n.d; not detected.

Locus_tag	Product	SignalP	COG	KO	PCI (%)
FOKN1_0577	tRNA-dihydrouridine synthase	N	S		n.d
FOKN1_0578	uncharacterized protein	N	no hit		n.d
FOKN1_0579	SAM-dependent methyltransferases	N	no hit		n.d
FOKN1_0580	sulfotransferase	N	no hit	K07808	n.d
FOKN1_0581	uncharacterized protein	N	no hit		n.d
FOKN1_0582	uncharacterized protein	N	no hit		n.d
FOKN1_0583	uncharacterized protein	N	no hit		n.d
FOKN1_0584	glycosyltransferase	N	no hit		n.d
FOKN1_0585	glycosyltransferase	N	M		n.d
FOKN1_0586	thymidylate kinase	N	M		n.d
FOKN1_0587	glycosyltransferase	N	M		n.d
FOKN1_0588	glycosyltransferase	N	no hit	K03208	0.009%
FOKN1_0589	uncharacterized protein	N	M		n.d
FOKN1_0590	undecaprenyl-phosphate alpha-N-acetylglucosaminyl 1-phosphate transferase	N	M	K02851	n.d
FOKN1_0591	GDP-mannose 4,6-dehydratase	N	no hit	K01711	0.026%
FOKN1_0592	NAD-dependent epimerase/dehydratase	N	MG	K02377	0.025%
FOKN1_0593	uncharacterized protein	N	R		0.022%
FOKN1_0594	uncharacterized protein	N	R		n.d
FOKN1_0595	signal transduction histidine kinase	N	T		n.d
FOKN1_0596	response regulator receiver protein	N	T	K02481	0.020%
FOKN1_0597	excinuclease ABC subunit C	N	no hit	K07461	n.d
FOKN1_0598	thiol:disulfide interchange protein DsbD	N	no hit	K04084	0.015%
FOKN1_0599	periplasmic divalent cation tolerance protein	N	P	K03926	n.d
FOKN1_0600	fxsA cytoplasmic membrane protein	N	P	K07113	n.d
FOKN1_0601	co-chaperonin GroES	N	no hit	K04078	0.170%
FOKN1_0602	chaperonin GroEL	N	no hit	K04077	3.995%
FOKN1_0603	transmembrane anti-sigma factor	N	no hit		n.d
FOKN1_0604	RNA polymerase, sigma-24 subunit, ECF subfamily	N	no hit	K03088	n.d
FOKN1_0605	TfP pilus assembly protein, ATPase PilU	N	no hit		0.019%
FOKN1_0606	glutamate-ammonia-ligase adenylyltransferase	N	no hit	K00982	0.005%
FOKN1_0607	branched-chain amino acid aminotransferase	N	no hit	K00826	0.054%
FOKN1_0608	uncharacterized protein	N	no hit		n.d
FOKN1_0609	nucleoside-diphosphate-sugar epimerase	N	M		n.d

Table S1. Gene annotation of gene-coding sequences (CDSs) and protein expression in the *Thiohalobacter sp.* strain FOKN1 cells. SignalP; prediction of signal peptide sequence, Y; positive, N; negative, COG; clusters of orthologous group, KO; KEGG orthology annotated using the KAAS, PCI; protein content index, n.d; not detected.

Locus_tag	Product	SignalP	COG	KO	PCI (%)
FOKN1_0610	glycosyltransferase	N	no hit		n.d
FOKN1_0611	uncharacterized protein	N	no hit		n.d
FOKN1_0612	uncharacterized protein	N	no hit		n.d
FOKN1_0613	glycosyltransferase	N	no hit		n.d
FOKN1_0614	ADP-heptose:LPS heptosyltransferase	N	M	K02849	n.d
FOKN1_0615	O-antigen polymerase	N	M		n.d
FOKN1_0616	lauroyl/myristoyl acyltransferase	N	no hit	K02517	n.d
FOKN1_0617	3-deoxy-D-manno-octulosonic-acid transferase	N	no hit	K02527	n.d
FOKN1_0618	uncharacterized protein	N	no hit		n.d
FOKN1_0619	uncharacterized protein	Y	no hit	K12340	0.084%
FOKN1_0620	rhodanese-related sulfurtransferase	N	no hit		n.d
FOKN1_0621	protein-L-isoaspartate(D-aspartate) O-methyltransferase	N	no hit	K00573	n.d
FOKN1_0622	tonB-dependent receptor	Y	no hit	K02014	0.346%
FOKN1_0623	glutamate synthase	N	no hit		n.d
FOKN1_0624	thiamine biosynthesis protein	N	no hit	K03147	0.028%
FOKN1_0625	uncharacterized protein	N	D		n.d
FOKN1_0626	polyketide synthase	N	D		0.103%
FOKN1_0627	ATPases	N	D		n.d
FOKN1_0628	uncharacterized protein	N	S		n.d
FOKN1_0629	transposase	N	no hit		n.d
FOKN1_0630	transcription elongation factor	N	no hit		n.d
FOKN1_0631	translation elongation factors	N	no hit		0.024%
FOKN1_0632	transcription elongation factor	N	no hit		n.d
FOKN1_0633	uncharacterized protein	N	no hit		n.d
FOKN1_0634	uncharacterized protein	N	no hit		n.d
FOKN1_0635	uncharacterized protein	N	no hit		n.d
FOKN1_0636	transcriptional regulator	N	K		n.d
FOKN1_0637	transcriptional regulator	N	K	K22106	0.020%
FOKN1_0638	uncharacterized protein	N	no hit		n.d
FOKN1_0639	uncharacterized protein	N	no hit		n.d
FOKN1_0640	uncharacterized protein	N	no hit		n.d
FOKN1_0641	uncharacterized protein	Y	no hit		0.023%
FOKN1_0642	large-conductance mechanosensitive channel	N	no hit	K03282	n.d
FOKN1_0643	uncharacterized protein	N	no hit		0.004%

Table S1. Gene annotation of gene-coding sequences (CDSs) and protein expression in the *Thiohalobacter sp.* strain FOKN1 cells. SignalP; prediction of signal peptide sequence, Y; positive, N; negative, COG; clusters of orthologous group, KO; KEGG orthology annotated using the KAAS, PCI; protein content index, n.d; not detected.

Locus_tag	Product	SignalP	COG	KO	PCI (%)
FOKN1_0644	sodium-dependent transporter	N	R	K03453	n.d
FOKN1_0645	signal-transduction protein	N	R		n.d
FOKN1_0646	uncharacterized protein	Y	no hit	K12460	n.d
FOKN1_0647	uncharacterized protein	N	no hit		n.d
FOKN1_0648	methyl-accepting chemotaxis protein	N	no hit	K03406	n.d
FOKN1_0649	small integral membrane protein	N	no hit		n.d
FOKN1_0650	uncharacterized protein	Y	S		n.d
FOKN1_0651	uncharacterized protein	N	S	K09966	n.d
FOKN1_0652	diguanylate cyclase/phosphodiesterase	N	no hit	K14051	n.d
FOKN1_0653	antibiotic biosynthesis monooxygenase	N	no hit	K21481	n.d
FOKN1_0654	arylsulfatase	N	HE		n.d
FOKN1_0655	uncharacterized protein	N	HE		n.d
FOKN1_0656	TRAP-type C4-dicarboxylate transporter, large permease component	N	HE		n.d
FOKN1_0657	topoisomerase	Y	HE		n.d
FOKN1_0658	transcriptional regulator/sugar kinase	N	G		0.018%
FOKN1_0659	phosphoenolpyruvate-protein phosphotransferase	N	G		n.d
FOKN1_0660	superoxide dismutase	N	no hit	K04564	0.072%
FOKN1_0661	ATPase	Y	no hit		0.025%
FOKN1_0662	type IV secretory pathway, VirB6 components	N	U		n.d
FOKN1_0663	permeases	N	no hit		n.d
FOKN1_0664	sulfite dehydrogenase cytochrome subunit SoxD	N	no hit		n.d
FOKN1_0665	ATPase	N	no hit	K06915	0.033%
FOKN1_0666	ABC-type antimicrobial peptide transporter, permease component	Y	V		n.d
FOKN1_0667	uncharacterized protein	N	R	K16482	n.d
FOKN1_0668	cobalamin biosynthesis protein	N	I		n.d
FOKN1_0669	adenosylmethionine-8-amino-7-oxononanoate aminotransferase	Y	I		n.d
FOKN1_0670	thioesterase	Y	no hit		n.d
FOKN1_0671	uncharacterized protein	Y	no hit		n.d
FOKN1_0672	phosphonate ABC transporter, periplasmic phosphonate-binding protein	N	no hit		n.d
FOKN1_0673	signal transduction protein	N	no hit		n.d

Table S1. Gene annotation of gene-coding sequences (CDSs) and protein expression in the *Thiohalobacter sp.* strain FOKN1 cells. SignalP; prediction of signal peptide sequence, Y; positive, N; negative, COG; clusters of orthologous group, KO; KEGG orthology annotated using the KAAS, PCI; protein content index, n.d; not detected.

Locus_tag	Product	SignalP	COG	KO	PCI (%)
FOKN1_0674	diguanylate cyclase/phosphodiesterase	N	no hit		0.003%
FOKN1_0675	diguanylate cyclase	N	T	K21085	n.d
FOKN1_0676	transcriptional regulators	N	no hit		n.d
FOKN1_0677	ribulose bisphosphate carboxylase small subunit	N	no hit		n.d
FOKN1_0678	uncharacterized protein	N	no hit		n.d
FOKN1_0679	3-dehydroquinate dehydratase II	N	no hit		n.d
FOKN1_0680	putative cysteine protease	N	no hit		n.d
FOKN1_0681	sulfurtransferase	N	no hit		n.d
FOKN1_0682	uncharacterized protein	Y	S		n.d
FOKN1_0683	amidohydrolase 2	N	S		n.d
FOKN1_0684	ion transport protein	N	S	K21862	n.d
FOKN1_0685	leucyl-tRNA synthetase	Y	no hit		n.d
FOKN1_0686	thiol-disulfide isomerase and thioredoxins	Y	OC		n.d
FOKN1_0687	uncharacterized protein	N	no hit		n.d
FOKN1_0688	glycine betaine/L-proline ABC transporterATP-binding protein	N	no hit	K02000	0.025%
FOKN1_0689	glycine betaine/L-proline ABC transporter permease	N	E	K02001	n.d
FOKN1_0690	proline/glycine betaine ABC transporter substrate-binding protein	Y	E	K02002	0.019%
FOKN1_0691	uncharacterized protein	Y	no hit		n.d
FOKN1_0692	uncharacterized protein	N	no hit		0.056%
FOKN1_0693	NTP pyrophosphohydrolases	N	no hit		n.d
FOKN1_0694	NADH:flavin oxidoreductase/NADH oxidase	N	C	K10680	0.070%
FOKN1_0695	alpha/beta hydrolase fold protein	N	no hit		n.d
FOKN1_0696	uncharacterized protein	N	no hit		n.d
FOKN1_0697	acetolactate synthase	N	no hit	K01652	n.d
FOKN1_0698	permeases	N	no hit	K07085	n.d
FOKN1_0699	uncharacterized protein	N	no hit		n.d
FOKN1_0700	uncharacterized protein	N	no hit		n.d
FOKN1_0701	cation/multidrug efflux pump	N	no hit		n.d
FOKN1_0702	ABC transporter ATP-binding protein	N	no hit	K02003	n.d
FOKN1_0703	ABC transporter permease protein	N	no hit	K02004	n.d
FOKN1_0704	uncharacterized protein	N	no hit		0.021%
FOKN1_0705	cytochrome c, mono- and diheme variants	Y	no hit		n.d

Table S1. Gene annotation of gene-coding sequences (CDSs) and protein expression in the *Thiohalobacter sp.* strain FOKN1 cells. SignalP; prediction of signal peptide sequence, Y; positive, N; negative, COG; clusters of orthologous group, KO; KEGG orthology annotated using the KAAS, PCI; protein content index, n.d; not detected.

Locus_tag	Product	SignalP	COG	KO	PCI (%)
FOKN1_0706	DNA/RNA helicases	N	no hit		n.d
FOKN1_0707	nitrite reductase	Y	no hit		n.d
FOKN1_0708	ribulose 1,5-bisphosphate carboxylase, large subunit	N	G	K01601	0.532%
FOKN1_0709	ribulose bisphosphate carboxylase, small chain	N	C	K01602	n.d
FOKN1_0710	carboxysome shell protein CsoS2	N	C		0.193%
FOKN1_0711	carboxysome shell protein CsoS3	N	no hit		0.015%
FOKN1_0712	carboxysome peptide A	N	QC		n.d
FOKN1_0713	carboxysome peptide B	N	QC		n.d
FOKN1_0714	microcompartments protein	N	QC		0.528%
FOKN1_0715	microcompartments protein	N	QC	K08696	0.545%
FOKN1_0716	microcompartments protein	N	QC	K08696	n.d
FOKN1_0717	bacterioferritin	N	no hit		n.d
FOKN1_0718	pterin-4a-carbinolamine dehydratase	N	no hit		n.d
FOKN1_0719	ATPase	N	no hit	K03496	n.d
FOKN1_0720	uncharacterized protein	N	no hit		n.d
FOKN1_0721	sulfate transporter	N	K		n.d
FOKN1_0722	transcriptional regulator	N	K	K21703	0.031%
FOKN1_0723	uncharacterized protein	N	K		n.d
FOKN1_0724	uncharacterized protein	N	no hit		n.d
FOKN1_0725	cytochrome c class I	Y	no hit		n.d
FOKN1_0726	sulfide dehydrogenase (flavocytochrome c), flavoprotein subunit	N	no hit		n.d
FOKN1_0727	cation transport regulator chaB	N	no hit	K06197	n.d
FOKN1_0728	uncharacterized protein	N	no hit	K09165	n.d
FOKN1_0729	uncharacterized protein	N	G		n.d
FOKN1_0730	uncharacterized protein	N	no hit		n.d
FOKN1_0731	ABC transporter	N	no hit	K15738	n.d
FOKN1_0732	galactose mutarotase	N	G		n.d
FOKN1_0733	phage-related tail protein	N	no hit		n.d
FOKN1_0734	uncharacterized protein	Y	no hit		n.d
FOKN1_0735	uncharacterized protein	Y	T		n.d
FOKN1_0736	diadenosine tetraphosphatase	N	T		n.d
FOKN1_0737	phosphate-selective porin O protein	Y	T		n.d
FOKN1_0738	pseudouridine synthase	N	J	K06183	n.d

Table S1. Gene annotation of gene-coding sequences (CDSs) and protein expression in the *Thiohalobacter sp.* strain FOKN1 cells. SignalP; prediction of signal peptide sequence, Y; positive, N; negative, COG; clusters of orthologous group, KO; KEGG orthology annotated using the KAAS, PCI; protein content index, n.d; not detected.

Locus_tag	Product	SignalP	COG	KO	PCI (%)
FOKN1_0739	P pilus assembly protein	N	no hit		n.d
FOKN1_0740	diguanylate cyclase	N	no hit	K13069	n.d
FOKN1_0741	homoserine O-succinyltransferase	N	no hit	K00651	0.010%
FOKN1_0742	ABC-type multidrug transporter, ATPase component	N	no hit		0.029%
FOKN1_0743	uncharacterized protein	N	no hit		0.017%
FOKN1_0744	intracellular septation protein A	N	D		n.d
FOKN1_0745	uncharacterized protein	N	D		n.d
FOKN1_0746	stringent starvation protein B	N	no hit		n.d
FOKN1_0747	metal dependent phosphohydrolase	N	no hit		n.d
FOKN1_0748	uncharacterized protein	N	no hit		n.d
FOKN1_0749	uncharacterized protein	Y	no hit		n.d
FOKN1_0750	uridylyltransferase	N	O		n.d
FOKN1_0751	Asp-tRNA Asn/Glu-tRNA Gln amidotransferase A subunit	N	J		n.d
FOKN1_0752	uncharacterized protein	N	T		n.d
FOKN1_0753	molybdenum cofactor biosynthesis protein MoaC	N	no hit	K03637	0.024%
FOKN1_0754	molybdopterin synthase subunit MoaD	N	no hit		n.d
FOKN1_0755	molybdopterin biosynthesis protein MoaE	N	no hit	K03635	n.d
FOKN1_0756	inorganic pyrophosphatase	N	no hit	K01507	n.d
FOKN1_0757	inorganic pyrophosphatase	N	no hit	K15987	0.006%
FOKN1_0758	6-phosphofructokinase	N	no hit	K21071	0.426%
FOKN1_0759	adenylate kinase	N	F	K00939	n.d
FOKN1_0760	phospholipase D/transphosphatidylase	N	F	K06131	n.d
FOKN1_0761	site-specific recombinase	N	no hit		n.d
FOKN1_0762	UDP-N-acetylmuramate:L-alanyl-gamma-D-glutamyl-meso-diaminopimelate ligase	N	no hit	K02558	0.012%
FOKN1_0763	3-polyprenyl-4-hydroxybenzoate decarboxylase	N	no hit	K03186	n.d
FOKN1_0764	peptidase S16, ion domain protein	N	no hit	K07157	n.d
FOKN1_0765	putative phosphoesterase PA-phosphatase-like protein	N	no hit	K19302	n.d
FOKN1_0766	glutamate-1-semialdehyde aminotransferase	N	no hit	K01845	0.091%
FOKN1_0767	thiamine-phosphate synthase	N	no hit	K00788	n.d
FOKN1_0768	phosphomethylpyrimidine kinase	N	no hit	K00941	0.025%
FOKN1_0769	ribulose-5-phosphate 4-epimerase-like epimerase	N	no hit	K01628	0.022%
FOKN1_0770	type II secretory pathway, pseudopilin PulG	N	no hit	K02456	n.d

Table S1. Gene annotation of gene-coding sequences (CDSs) and protein expression in the *Thiohalobacter sp.* strain FOKN1 cells. SignalP; prediction of signal peptide sequence, Y; positive, N; negative, COG; clusters of orthologous group, KO; KEGG orthology annotated using the KAAS, PCI; protein content index, n.d; not detected.

Locus_tag	Product	SignalP	COG	KO	PCI (%)
FOKN1_0771	type II secretory pathway, pseudopilin PulG	N	no hit		n.d
FOKN1_0772	type II secretory pathway, pseudopilin PulG	N	no hit		n.d
FOKN1_0773	type II secretory pathway, component PulJ	N	no hit	K02459	0.054%
FOKN1_0774	type II secretory pathway, component PulK	N	no hit	K02460	0.012%
FOKN1_0775	type II secretory pathway, component PulL	N	no hit	K02461	n.d
FOKN1_0776	type II secretory pathway, component PulM	N	no hit		0.043%
FOKN1_0777	type II secretion system protein N	N	no hit		0.055%
FOKN1_0778	Fe-S-cluster oxidoreductase	N	R		n.d
FOKN1_0779	hydrolase	N	no hit	K20881	0.016%
FOKN1_0780	NTP pyrophosphohydrolases	N	no hit	K08312	n.d
FOKN1_0781	3'-phosphoadenosine 5'-phosphosulfate (PAPS) 3'-phosphatase	N	no hit	K01082	0.033%
FOKN1_0782	uncharacterized protein	N	S		n.d
FOKN1_0783	type II secretory pathway, component ExeA	N	no hit		0.008%
FOKN1_0784	ATPases	N	no hit	K16692	n.d
FOKN1_0785	uncharacterized protein	N	no hit		n.d
FOKN1_0786	phosphate uptake regulator	N	P		n.d
FOKN1_0787	uncharacterized protein	Y	M	K01991	0.020%
FOKN1_0788	uncharacterized protein	Y	no hit		n.d
FOKN1_0789	long-chain fatty acid transport protein	Y	I	K06076	0.026%
FOKN1_0790	fructose-1-phosphate kinase	Y	I		n.d
FOKN1_0791	uncharacterized protein	N	R		n.d
FOKN1_0792	methyltransferase	N	QR		0.006%
FOKN1_0793	SAM-dependent methyltransferases	N	QR		n.d
FOKN1_0794	O-linked N-acetylglucosamine transferase	N	O	K09667	0.007%
FOKN1_0795	uncharacterized protein	N	R		n.d
FOKN1_0796	acetyl-CoA acetyltransferase	N	no hit		n.d
FOKN1_0797	peptidyl-prolyl cis-trans isomerase	N	no hit	K12737	n.d
FOKN1_0798	3-dehydroquinate dehydratase, type II	N	I	K03786	n.d
FOKN1_0799	acetyl-CoA carboxylase, biotin carboxyl carrier protein	N	I	K02160	n.d
FOKN1_0800	uncharacterized protein	N	I		n.d
FOKN1_0801	acetyl-CoA carboxylase, biotin carboxylase	N	I	K01961	0.059%
FOKN1_0802	ribosomal protein L11 methyltransferase	N	no hit	K02687	n.d
FOKN1_0803	uncharacterized protein	N	no hit		n.d

Table S1. Gene annotation of gene-coding sequences (CDSs) and protein expression in the *Thiohalobacter sp.* strain FOKN1 cells. SignalP; prediction of signal peptide sequence, Y; positive, N; negative, COG; clusters of orthologous group, KO; KEGG orthology annotated using the KAAS, PCI; protein content index, n.d; not detected.

Locus_tag	Product	SignalP	COG	KO	PCI (%)
FOKN1_0804	tRNA-dihydrouridine synthase	N	no hit	K05540	n.d
FOKN1_0805	transcriptional regulator	N	no hit	K03557	n.d
FOKN1_0806	phosphoribosyl aminoimidazole carboxamide formyltransferase/IMP cyclohydrolase	N	no hit	K00602	0.071%
FOKN1_0807	phosphoribosylamine-glycine ligase	N	no hit	K01945	0.045%
FOKN1_0808	transcription termination factor	N	no hit	K03628	0.262%
FOKN1_0809	thiol-disulfide isomerase and thioredoxins	N	no hit	K03671	n.d
FOKN1_0810	DNA/RNA helicase	N	LKJ	K03732	0.044%
FOKN1_0811	general secretion pathway protein C	N	LKJ		0.080%
FOKN1_0812	general secretion pathway protein D	Y	no hit	K02453	0.076%
FOKN1_0813	general secretory pathway protein E	N	no hit	K02454	0.069%
FOKN1_0814	general secretion pathway protein F	N	no hit	K02455	n.d
FOKN1_0815	arabinose efflux permease family protein	N	GEPR		n.d
FOKN1_0816	uncharacterized protein	Y	GEPR		0.010%
FOKN1_0817	fumarate lyase	N	no hit	K01679	n.d
FOKN1_0818	3',5'-cyclic adenosine monophosphate phosphodiesterase CpdA	N	no hit	K03651	n.d
FOKN1_0819	N-acetylglutamate synthase	N	no hit	K14682	0.025%
FOKN1_0820	acetylornithine deacetylase	N	no hit	K01438	0.010%
FOKN1_0821	acetyltransferases	N	no hit		n.d
FOKN1_0822	glutathione synthase	N	no hit		n.d
FOKN1_0823	methyl-accepting chemotaxis sensory transducer	N	no hit	K03776	n.d
FOKN1_0824	dihydroxyacid dehydratase	N	no hit	K01687	0.042%
FOKN1_0825	uncharacterized protein	N	no hit		0.240%
FOKN1_0826	N-acetyl-anhydromuranmyl-L-alanine amidase	N	no hit	K03806	n.d
FOKN1_0827	cobalamin biosynthesis protein	N	no hit		n.d
FOKN1_0828	fructose-2,6-bisphosphatase	N	no hit	K02226	n.d
FOKN1_0829	excinuclease ATPase subunit	N	no hit		n.d
FOKN1_0830	exodeoxyribonuclease III	N	no hit	K01142	0.026%
FOKN1_0831	signal transduction protein	N	no hit		0.017%
FOKN1_0832	phosphoesterase	N	R		n.d
FOKN1_0833	uncharacterized protein	Y	R		n.d
FOKN1_0834	uncharacterized protein	N	no hit		n.d
FOKN1_0835	uncharacterized protein	N	no hit		0.169%

Table S1. Gene annotation of gene-coding sequences (CDSs) and protein expression in the *Thiohalobacter sp.* strain FOKN1 cells. SignalP; prediction of signal peptide sequence, Y; positive, N; negative, COG; clusters of orthologous group, KO; KEGG orthology annotated using the KAAS, PCI; protein content index, n.d; not detected.

Locus_tag	Product	SignalP	COG	KO	PCI (%)
FOKN1_0836	tRNA guanosine-2'-O-methyltransferase	N	no hit	K00556	n.d
FOKN1_0837	uncharacterized protein	N	no hit	K07168	n.d
FOKN1_0838	glycerate dehydrogenase	N	no hit	K00018	n.d
FOKN1_0839	arginyl-tRNA synthetase	N	no hit	K03406	n.d
FOKN1_0840	putative two-component response regulator	N	TK	K15012	n.d
FOKN1_0841	sensor histidine kinase	N	T	K15011	n.d
FOKN1_0842	outer membrane receptor	N	T		0.005%
FOKN1_0843	uncharacterized protein	Y	no hit		n.d
FOKN1_0844	uncharacterized protein	N	no hit		n.d
FOKN1_0845	aspartyl/asparaginyl-tRNA synthetases	N	no hit		n.d
FOKN1_0846	NTP pyrophosphohydrolases	N	no hit	K01515	n.d
FOKN1_0847	pyrimidine reductase	N	no hit		n.d
FOKN1_0848	spermidine synthase	N	no hit	K00797	n.d
FOKN1_0849	transcriptional regulator	N	no hit	K17472	0.028%
FOKN1_0850	putative regulator	N	no hit	K07740	n.d
FOKN1_0851	protein-S-isoprenylcysteine methyltransferase	N	no hit		n.d
FOKN1_0852	fructose-1,6-bisphosphatase	N	no hit	K03841	0.046%
FOKN1_0853	signal transduction histidine kinase	N	no hit		n.d
FOKN1_0854	hydroxyacylglutathione hydrolase	N	no hit	K01069	n.d
FOKN1_0855	ATPases	N	no hit	K06916	n.d
FOKN1_0856	dissimilatory sulfite reductase (desulfoviridin), gamma subunit	N	no hit		n.d
FOKN1_0857	uncharacterized protein	N	no hit		n.d
FOKN1_0858	ADP-ribose pyrophosphatase	N	no hit		n.d
FOKN1_0859	pyruvate/2-oxoglutarate dehydrogenase complex	N	no hit		0.170%
FOKN1_0860	type IV pilus assembly PilZ	N	no hit		n.d
FOKN1_0861	fructose-1,6-bisphosphatase	N	no hit	K01092	n.d
FOKN1_0862	ABC-type antimicrobial peptide transporter	N	no hit		n.d
FOKN1_0863	uncharacterized protein	N	no hit	K08997	n.d
FOKN1_0864	ferric iron ABC transporter, iron-binding protein	Y	no hit	K02012	0.235%
FOKN1_0865	ferric iron ABC transporter, permease	N	no hit	K02011	n.d
FOKN1_0866	ferric iron ABC transporter, ATPase	N	no hit	K02010	n.d
FOKN1_0867	peptidyl-prolyl cis-trans isomerase	N	no hit		n.d
FOKN1_0868	peptide methionine sulfoxide reductase relatedprotein	N	no hit	K07305	n.d

Table S1. Gene annotation of gene-coding sequences (CDSs) and protein expression in the *Thiohalobacter sp.* strain FOKN1 cells. SignalP; prediction of signal peptide sequence, Y; positive, N; negative, COG; clusters of orthologous group, KO; KEGG orthology annotated using the KAAS, PCI; protein content index, n.d; not detected.

Locus_tag	Product	SignalP	COG	KO	PCI (%)
FOKN1_0869	3'-phosphoadenosine 5'-phosphosulfate (PAPS) 3'-phosphatase	N	P		n.d
FOKN1_0870	uncharacterized protein	N	no hit		n.d
FOKN1_0871	uncharacterized protein	N	no hit		n.d
FOKN1_0872	uncharacterized protein	N	no hit		n.d
FOKN1_0873	ferredoxin	N	no hit		n.d
FOKN1_0874	uncharacterized protein	N	no hit		n.d
FOKN1_0875	uncharacterized protein	N	no hit		n.d
FOKN1_0876	Fe-S oxidoreductase	N	no hit		n.d
FOKN1_0877	oligoendopeptidase	N	no hit		n.d
FOKN1_0878	rhodanese-related sulfurtransferase	N	P	K07376	n.d
FOKN1_0879	ATPases	N	no hit		n.d
FOKN1_0880	acetylornithine deacetylase/succinyl-diaminopimelate desuccinylase	N	no hit		n.d
FOKN1_0881	uncharacterized protein	N	no hit		n.d
FOKN1_0882	heat shock protein DnaJ	N	no hit		n.d
FOKN1_0883	uncharacterized protein	Y	no hit		n.d
FOKN1_0884	uncharacterized protein	Y	no hit		n.d
FOKN1_0885	ABC-type metal ion transporter	Y	P	K02077	n.d
FOKN1_0886	ABC-type Mn2+/Zn2+ transporter	N	P	K02075	n.d
FOKN1_0887	uncharacterized protein	N	no hit		n.d
FOKN1_0888	adenosylhomocysteinase	N	no hit		0.033%
FOKN1_0889	4-hydroxy-3-methylbut-2-en-1-yl diphosphatesynthase	N	no hit	K03526	0.015%
FOKN1_0890	transcriptional regulator	N	no hit	K13771	0.110%
FOKN1_0891	dissimilatory sulfite reductase	N	no hit		n.d
FOKN1_0892	rhodanese-related sulfurtransferase	N	no hit	K01011	n.d
FOKN1_0893	uncharacterized protein	N	no hit		n.d
FOKN1_0894	uncharacterized protein	N	no hit	K03744	0.021%
FOKN1_0895	uncharacterized protein	N	KT		n.d
FOKN1_0896	SOS-response transcriptional repressors	N	KT	K01356	n.d
FOKN1_0897	nucleotidyltransferase/DNA polymerase	N	L	K02346	n.d
FOKN1_0899	geranylgeranyl pyrophosphate synthase	N	no hit	K02523	n.d
FOKN1_0900	50S ribosomal protein L21	N	no hit	K02888	0.285%
FOKN1_0901	50S ribosomal protein L27	N	no hit	K02899	0.443%

Table S1. Gene annotation of gene-coding sequences (CDSs) and protein expression in the *Thiohalobacter sp.* strain FOKN1 cells. SignalP; prediction of signal peptide sequence, Y; positive, N; negative, COG; clusters of orthologous group, KO; KEGG orthology annotated using the KAAS, PCI; protein content index, n.d; not detected.

Locus_tag	Product	SignalP	COG	KO	PCI (%)
FOKN1_0902	GTPase	N	no hit	K03979	n.d
FOKN1_0903	glutamate 5-kinase	N	no hit	K00931	0.011%
FOKN1_0904	ribosomal protein S20	N	no hit	K02968	0.147%
FOKN1_0905	uncharacterized protein	N	no hit	K03980	0.007%
FOKN1_0906	FAD synthase	N	no hit	K11753	n.d
FOKN1_0907	isoleucyl-tRNA synthetase	N	no hit	K01870	0.040%
FOKN1_0908	lipoprotein signal peptidase	N	no hit	K03101	n.d
FOKN1_0909	peptidyl-prolyl cis-trans isomerase	N	no hit	K03774	n.d
FOKN1_0910	hydroxymethylbutenyl pyrophosphate reductase	N	no hit	K03527	0.037%
FOKN1_0911	type IV pilus biogenesis protein PilE	N	no hit		n.d
FOKN1_0912	P pilus assembly protein, porin PapC	Y	no hit		n.d
FOKN1_0913	TfP pilus assembly protein, tip-associated adhesin PilY1	N	no hit	K02674	0.016%
FOKN1_0914	type IV pilus assembly protein PilX	N	no hit		n.d
FOKN1_0915	TfP pilus assembly protein PilW	N	no hit	K02672	n.d
FOKN1_0916	TfP pilus assembly protein PilV	N	no hit		n.d
FOKN1_0917	TfP pilus assembly protein FimT	N	M		0.037%
FOKN1_0918	TfP pilus assembly protein PilV	N	M		n.d
FOKN1_0919	uncharacterized protein	N	M		n.d
FOKN1_0920	type II secretory pathway, component PulJ	N	no hit		n.d
FOKN1_0921	type II secretory pathway, pseudopilin PulG	N	no hit		n.d
FOKN1_0922	TfP pilus assembly protein PilE	N	no hit		n.d
FOKN1_0923	glycine/D-amino acid oxidases	N	no hit	K03153	0.042%
FOKN1_0924	Fe2+/Zn2+ uptake regulation protein	N	E	K09826	n.d
FOKN1_0925	uncharacterized protein	N	E		n.d
FOKN1_0926	hydantoinase/carbamoylase family amidase	N	E	K06016	0.009%
FOKN1_0927	isochorismatase hydrolase	N	no hit		n.d
FOKN1_0928	Na+/solute symporter	N	F	K20989	n.d
FOKN1_0929	aspartate carbamoyltransferase	N	F	K00609	0.015%
FOKN1_0930	asparagine synthase	N	no hit	K01953	0.099%
FOKN1_0932	uncharacterized protein	N	no hit	K09946	3.110%
FOKN1_0933	uncharacterized protein	N	no hit		n.d
FOKN1_0934	transcriptional regulator	N	no hit	K15836	n.d
FOKN1_0935	ABC-type nitrate/sulfonate/bicarbonate transporters, periplasmic components	N	no hit	K22067	n.d

Table S1. Gene annotation of gene-coding sequences (CDSs) and protein expression in the *Thiohalobacter sp.* strain FOKN1 cells. SignalP; prediction of signal peptide sequence, Y; positive, N; negative, COG; clusters of orthologous group, KO; KEGG orthology annotated using the KAAS, PCI; protein content index, n.d; not detected.

Locus_tag	Product	SignalP	COG	KO	PCI (%)
FOKN1_0936	ABC-type nitrate/sulfonate/bicarbonate transporter, permease component	N	no hit	K15577	n.d
FOKN1_0937	ABC-type nitrate/sulfonate/bicarbonate transporter, ATPase component	N	P	K15579	n.d
FOKN1_0938	cyanate lyase	N	KT	K01725	n.d
FOKN1_0939	uncharacterized protein	N	KT		n.d
FOKN1_0940	carbonic anhydrase	N	KT	K01673	n.d
FOKN1_0941	transcriptional regulator	N	KT	K03721	n.d
FOKN1_0942	uncharacterized protein	N	V		n.d
FOKN1_0943	cation/multidrug efflux pump	N	V	K03296	n.d
FOKN1_0944	uncharacterized protein	Y	V		n.d
FOKN1_0945	diguanylate cyclase	N	V		n.d
FOKN1_0946	putative signal transduction protein	N	no hit		0.032%
FOKN1_0947	exonuclease	N	no hit	K07576	0.089%
FOKN1_0948	nicotinate-nucleotide pyrophosphorylase	N	no hit	K00767	0.013%
FOKN1_0949	Na+/alanine symporter	N	no hit	K03310	n.d
FOKN1_0950	aspartic protease	N	no hit	K06985	n.d
FOKN1_0951	uncharacterized protein	N	no hit		n.d
FOKN1_0952	uridylyltransferase	N	no hit		n.d
FOKN1_0953	nitrogen regulatory protein P-II	N	E		n.d
FOKN1_0954	signal transduction protein	N	no hit		n.d
FOKN1_0955	DNA-binding response regulator PhoB	N	no hit	K07657	0.032%
FOKN1_0956	histidine kinase	N	no hit	K07636	0.017%
FOKN1_0957	ABC-type phosphate transporter, periplasmic component	Y	no hit	K02040	n.d
FOKN1_0958	uncharacterized protein	N	no hit		n.d
FOKN1_0959	exopolyphosphatase	N	no hit	K01524	0.016%
FOKN1_0960	uncharacterized protein	N	no hit		0.040%
FOKN1_0961	uncharacterized protein	N	no hit	K09929	0.014%
FOKN1_0962	uncharacterized protein	N	no hit	K09930	n.d
FOKN1_0963	uncharacterized protein	Y	no hit		n.d
FOKN1_0964	DNA glycosylase and apyrimidinic (AP) lyase	N	no hit	K10773	n.d
FOKN1_0965	diguanylate cyclase/phosphodiesterase	N	no hit	K21084	n.d
FOKN1_0966	NADH:ubiquinone oxidoreductase, subunit RnfE	N	no hit	K03613	n.d
FOKN1_0967	NADH:ubiquinone oxidoreductase, subunit RnfG	N	no hit	K03612	0.029%

Table S1. Gene annotation of gene-coding sequences (CDSs) and protein expression in the *Thiohalobacter sp.* strain FOKN1 cells. SignalP; prediction of signal peptide sequence, Y; positive, N; negative, COG; clusters of orthologous group, KO; KEGG orthology annotated using the KAAS, PCI; protein content index, n.d; not detected.

Locus_tag	Product	SignalP	COG	KO	PCI (%)
FOKN1_0968	NADH:ubiquinone oxidoreductase, subunit RnfD	N	no hit	K03614	n.d
FOKN1_0969	NADH:ubiquinone oxidoreductase, subunit RnfC	N	no hit	K03615	n.d
FOKN1_0970	NADH:ubiquinone oxidoreductase, subunit RnfB	N	no hit	K03616	n.d
FOKN1_0971	NADH:ubiquinone oxidoreductase, subunit RnfA	N	no hit	K03617	n.d
FOKN1_0972	methionyl-tRNA synthetase	N	no hit	K01874	0.023%
FOKN1_0973	ATPase	N	no hit	K03593	0.048%
FOKN1_0974	deoxycytidine deaminase	N	K	K01494	n.d
FOKN1_0975	cobalamin biosynthesis protein	N	K		n.d
FOKN1_0976	phosphoenolpyruvate carboxylase	N	no hit	K01595	0.033%
FOKN1_0977	glycogen/starch synthase	N	G	K00703	0.008%
FOKN1_0978	6-phosphogluconate dehydrogenase-like protein	N	G	K00033	0.048%
FOKN1_0979	glucose-6-phosphate 1-dehydrogenase	N	no hit	K00036	0.035%
FOKN1_0980	glucose-6-phosphate isomerase	N	no hit	K01810	0.018%
FOKN1_0981	uncharacterized protein	N	no hit		n.d
FOKN1_0982	2-methylthioadenine synthetase	N	J	K06168	n.d
FOKN1_0983	phosphate starvation-inducible protein PhoH, ATPase	N	J	K06217	0.018%
FOKN1_0984	endoribonuclease YbeY	N	no hit	K07042	n.d
FOKN1_0985	Mg2+ and Co2+ transporter	N	no hit	K06189	0.013%
FOKN1_0986	apolipoprotein N-acyltransferase	N	no hit	K03820	n.d
FOKN1_0987	uncharacterized protein	N	no hit		n.d
FOKN1_0988	uncharacterized protein	N	no hit		0.033%
FOKN1_0989	leucyl-tRNA synthetase	N	no hit	K01869	0.015%
FOKN1_0990	uncharacterized protein	N	no hit		0.072%
FOKN1_0991	DNA polymerase III, delta subunit	N	no hit	K02340	n.d
FOKN1_0992	gamma-glutamyl phosphate reductase	N	no hit	K00147	0.018%
FOKN1_0993	nicotinic acid mononucleotide adenylyltransferase	N	no hit	K00969	n.d
FOKN1_0994	uncharacterized protein	N	no hit	K09710	n.d
FOKN1_0995	uncharacterized protein	N	no hit	K00783	n.d
FOKN1_0996	uncharacterized protein	N	no hit	K06287	n.d
FOKN1_0997	ribonuclease G	N	no hit	K08301	0.028%
FOKN1_0998	uncharacterized protein	N	no hit		n.d
FOKN1_0999	uncharacterized protein	N	no hit		n.d
FOKN1_1000	nitrilase/cyanide hydratase and apolipoprotein N-acyltransferase	N	no hit	K11206	0.054%

Table S1. Gene annotation of gene-coding sequences (CDSs) and protein expression in the *Thiohalobacter sp.* strain FOKN1 cells. SignalP; prediction of signal peptide sequence, Y; positive, N; negative, COG; clusters of orthologous group, KO; KEGG orthology annotated using the KAAS, PCI; protein content index, n.d; not detected.

Locus_tag	Product	SignalP	COG	KO	PCI (%)
FOKN1_1001	Zn-dependent proteases	N	no hit	K03568	0.051%
FOKN1_1002	multimeric flavodoxin	Y	no hit		n.d
FOKN1_1003	ABC-type multidrug transporter, ATPase and permease components	N	no hit		n.d
FOKN1_1004	Kef-type K+ transporters	N	no hit		n.d
FOKN1_1005	cytochrome P460	Y	no hit		n.d
FOKN1_1006	cation transport ATPase	N	no hit		n.d
FOKN1_1007	uncharacterized protein	Y	no hit		n.d
FOKN1_1008	uncharacterized protein	N	no hit	K09889	n.d
FOKN1_1009	uncharacterized protein	N	no hit		n.d
FOKN1_1010	Zn-dependent proteases	N	no hit	K03592	0.039%
FOKN1_1011	ribosomal protein S20	N	no hit		n.d
FOKN1_1012	tryptophan synthase subunit beta	N	R	K06001	n.d
FOKN1_1013	beta-lactamase-like protein	N	no hit	K17725	n.d
FOKN1_1014	uncharacterized protein	Y	R		0.033%
FOKN1_1015	Mg/Co/Ni transporter MgtE	N	no hit	K06213	n.d
FOKN1_1016	phosphoenolpyruvate-protein phosphotransferase	N	no hit	K08483	0.006%
FOKN1_1017	phosphotransferase system, HPR-related proteins	N	no hit	K11189	n.d
FOKN1_1018	phosphotransferase system, mannose/fructose-specific component IIA	N	no hit	K02793	n.d
FOKN1_1019	uncharacterized protein	N	no hit	K06958	n.d
FOKN1_1020	serine kinase	N	no hit	K06023	n.d
FOKN1_1021	phosphotransferase system mannitol/fructose-specific IIA domain	N	no hit	K02806	n.d
FOKN1_1022	ribosome-associated protein Y	N	no hit	K05808	n.d
FOKN1_1023	RNA polymerase sigma-54 subunit RpoN	N	no hit	K03092	0.012%
FOKN1_1024	ABC-type transporter, ATPase component	N	no hit	K06861	n.d
FOKN1_1025	ABC transporter substrate-binding protein	Y	no hit	K09774	n.d
FOKN1_1026	uncharacterized protein	N	no hit	K11719	n.d
FOKN1_1027	3-deoxy-D-manno-octulosonate 8-phosphatephosphatase	N	no hit	K03270	0.085%
FOKN1_1028	arabinose 5-phosphate isomerase	N	no hit	K06041	0.037%
FOKN1_1029	copper chaperone	N	no hit		n.d
FOKN1_1030	Ca2+/Na+ antiporter	N	no hit	K07301	n.d
FOKN1_1031	uncharacterized protein	N	no hit		n.d

Table S1. Gene annotation of gene-coding sequences (CDSs) and protein expression in the *Thiohalobacter sp.* strain FOKN1 cells. SignalP; prediction of signal peptide sequence, Y; positive, N; negative, COG; clusters of orthologous group, KO; KEGG orthology annotated using the KAAS, PCI; protein content index, n.d; not detected.

Locus_tag	Product	SignalP	COG	KO	PCI (%)
FOKN1_1032	ABC-type transporter, ATPase component	N	no hit	K02065	0.061%
FOKN1_1033	ABC-type transporter, permease component	N	no hit	K02066	n.d
FOKN1_1034	ABC-type transporter, periplasmic component	N	no hit	K02067	0.027%
FOKN1_1035	anti-anti-sigma regulatory factor	N	no hit		n.d
FOKN1_1036	transcriptional regulator	N	no hit		n.d
FOKN1_1037	UDP-N-acetylglucosamine1-carboxyvinyltransferase	N	no hit	K00790	0.028%
FOKN1_1038	ATP phosphoribosyltransferase catalytic subunit	N	no hit	K00765	0.064%
FOKN1_1039	histidinol dehydrogenase	N	no hit	K00013	0.013%
FOKN1_1040	histidinol-phosphate aminotransferase	N	no hit	K00817	0.011%
FOKN1_1041	uncharacterized protein	Y	no hit		n.d
FOKN1_1042	phosphoribosylglycinamide formyltransferase	N	no hit	K11175	n.d
FOKN1_1043	phosphoribosylaminoimidazole synthetase	N	no hit	K01933	n.d
FOKN1_1044	uncharacterized protein	Y	no hit	K09938	0.032%
FOKN1_1045	phosphatidylglycerophosphate synthase	N	no hit		n.d
FOKN1_1046	permease	N	no hit		n.d
FOKN1_1047	ATPase	N	no hit	K10763	n.d
FOKN1_1048	uncharacterized protein	N	no hit		n.d
FOKN1_1049	multimeric flavodoxin	N	no hit	K03809	0.055%
FOKN1_1050	arsenate reductase	N	no hit	K00537	n.d
FOKN1_1051	uncharacterized protein	Y	no hit		n.d
FOKN1_1052	uncharacterized protein	Y	no hit		1.243%
FOKN1_1053	uncharacterized protein	N	no hit		0.014%
FOKN1_1054	thiol-disulfide isomerase and thioredoxins	Y	no hit		n.d
FOKN1_1055	thioredoxin-related protein	Y	no hit		n.d
FOKN1_1056	uncharacterized protein	N	no hit		0.048%
FOKN1_1057	uncharacterized protein	Y	M		0.017%
FOKN1_1058	acylphosphatase	N	C	K01512	n.d
FOKN1_1059	L-aspartate oxidase	N	no hit	K00278	0.010%
FOKN1_1060	RNA polymerase sigma factor	N	no hit	K03088	n.d
FOKN1_1061	negative regulatory protein	N	no hit		n.d
FOKN1_1062	negative regulatory protein	Y	no hit	K03598	0.096%
FOKN1_1063	positive regulator	N	no hit		n.d
FOKN1_1064	trypsin-like serine proteases	Y	no hit	K04771	0.082%
FOKN1_1065	thiol-disulfide isomerase and thioredoxins	N	no hit		n.d

Table S1. Gene annotation of gene-coding sequences (CDSs) and protein expression in the *Thiohalobacter sp.* strain FOKN1 cells. SignalP; prediction of signal peptide sequence, Y; positive, N; negative, COG; clusters of orthologous group, KO; KEGG orthology annotated using the KAAS, PCI; protein content index, n.d; not detected.

Locus_tag	Product	SignalP	COG	KO	PCI (%)
FOKN1_1066	membrane GTPase	N	no hit	K03596	n.d
FOKN1_1067	signal peptidase I	N	no hit	K03100	n.d
FOKN1_1068	uncharacterized protein	N	no hit		0.197%
FOKN1_1069	ribonuclease III	N	no hit	K03685	0.028%
FOKN1_1070	GTPase	N	no hit	K03595	n.d
FOKN1_1071	DNA repair protein	N	no hit	K03584	n.d
FOKN1_1072	pyridoxal phosphate biosynthesis protein	N	no hit	K03474	0.014%
FOKN1_1073	phosphopantetheinyl transferase	N	no hit	K00997	n.d
FOKN1_1074	signal transduction histidine kinase	N	no hit	K07678	n.d
FOKN1_1075	ABC-type uncharacterized transporter, periplasmic component	Y	no hit		n.d
FOKN1_1076	uncharacterized protein	Y	no hit	K02014	n.d
FOKN1_1077	cysteine synthase B	N	no hit	K12339	0.104%
FOKN1_1078	tryptophanyl-tRNA synthetase	N	no hit		n.d
FOKN1_1079	uncharacterized protein	N	no hit		n.d
FOKN1_1080	3'-5' exonuclease	N	no hit	K07501	0.074%
FOKN1_1081	SAM-dependent methyltransferases	N	E	K03215	n.d
FOKN1_1082	nitrogen regulatory protein PII	N	E		n.d
FOKN1_1083	uncharacterized protein	N	E	K09822	n.d
FOKN1_1084	NADH:ubiquinone oxidoreductase subunit 5	N	CP		n.d
FOKN1_1085	NADH:ubiquinone oxidoreductase subunit 5	N	CP	K00341	n.d
FOKN1_1086	soluble lytic murein transglycosylase	Y	CP		n.d
FOKN1_1087	NTP pyrophosphohydrolases	N	CP	K08310	n.d
FOKN1_1088	long-chain fatty acid transport protein	Y	no hit		n.d
FOKN1_1089	uncharacterized protein	N	no hit		n.d
FOKN1_1090	Holliday junction resolvasome, endonuclease subunit	N	no hit	K01159	n.d
FOKN1_1091	Holliday junction resolvasome, DNA-binding subunit	N	no hit	K03550	n.d
FOKN1_1092	Holliday junction resolvasome, helicase subunit	N	no hit	K03551	n.d
FOKN1_1093	thioesterase	N	no hit	K07107	n.d
FOKN1_1094	biopolymer transport proteins	N	no hit	K03562	0.037%
FOKN1_1095	biopolymer transport protein	N	no hit	K03560	0.031%
FOKN1_1096	uncharacterized protein	N	no hit		0.028%
FOKN1_1097	biopolymer transport protein	N	no hit	K03641	0.197%
FOKN1_1098	peptidoglycan-associated outer membrane lipoprotein	N	no hit	K03640	1.992%

Table S1. Gene annotation of gene-coding sequences (CDSs) and protein expression in the *Thiohalobacter sp.* strain FOKN1 cells. SignalP; prediction of signal peptide sequence, Y; positive, N; negative, COG; clusters of orthologous group, KO; KEGG orthology annotated using the KAAS, PCI; protein content index, n.d; not detected.

Locus_tag	Product	SignalP	COG	KO	PCI (%)
FOKN1_1099	uncharacterized protein	Y	no hit		n.d
FOKN1_1100	uncharacterized protein	N	no hit	K10026	n.d
FOKN1_1101	uncharacterized protein	N	no hit	K06920	0.026%
FOKN1_1102	uncharacterized protein	N	no hit		n.d
FOKN1_1104	response regulator	N	T	K02481	n.d
FOKN1_1105	Zn-dependent hydrolases	N	T		n.d
FOKN1_1106	uncharacterized protein	Y	no hit		n.d
FOKN1_1107	uncharacterized protein	Y	no hit		n.d
FOKN1_1108	cation/multidrug efflux pump	N	no hit		n.d
FOKN1_1109	cytochrome bd-type quinol oxidase, subunit 1	N	no hit	K00425	n.d
FOKN1_1110	cytochrome bd-type quinol oxidase, subunit 2	N	no hit	K00426	n.d
FOKN1_1111	uncharacterized protein	N	P		n.d
FOKN1_1112	C4-dicarboxylate ABC transporter	N	P	K03304	n.d
FOKN1_1113	uncharacterized protein	Y	P		n.d
FOKN1_1114	thiosulfate reductase cytochrome B subunit	N	no hit	K08354	n.d
FOKN1_1115	rhodanese-related sulfurtransferase	Y	no hit		n.d
FOKN1_1116	uncharacterized protein	N	no hit		n.d
FOKN1_1117	enoyl-CoA hydratase/carnithine racemase	N	I		n.d
FOKN1_1118	uncharacterized protein	N	no hit		n.d
FOKN1_1119	transcriptional regulator	N	G	K04761	n.d
FOKN1_1120	2,3-bisphosphoglycerate-dependent phosphoglycerate mutase	N	G	K01834	n.d
FOKN1_1121	DNA-binding ferritin-like protein	N	G	K04047	0.207%
FOKN1_1122	signal transduction histidine kinase	N	no hit		n.d
FOKN1_1123	putative heme degradation protein	N	no hit	K07225	0.024%
FOKN1_1124	hemin uptake protein	N	no hit		n.d
FOKN1_1125	acyl-CoA dehydrogenases	Y	no hit		0.050%
FOKN1_1126	uncharacterized protein	N	R		0.008%
FOKN1_1127	tonB-denpendent receptor	N	R	K16087	0.140%
FOKN1_1128	glycosyltransferases	Y	no hit		0.013%
FOKN1_1129	pyridoxamine 5'-phosphate oxidase-related FMN-binding protein	N	no hit	K07226	0.275%
FOKN1_1130	ABC-type hemin transporter, periplasmic component	Y	no hit	K02016	0.105%
FOKN1_1131	ABC-type transporter, integral membrane subunit	N	no hit	K02015	n.d

Table S1. Gene annotation of gene-coding sequences (CDSs) and protein expression in the *Thiohalobacter sp.* strain FOKN1 cells. SignalP; prediction of signal peptide sequence, Y; positive, N; negative, COG; clusters of orthologous group, KO; KEGG orthology annotated using the KAAS, PCI; protein content index, n.d; not detected.

Locus_tag	Product	SignalP	COG	KO	PCI (%)
FOKN1_1132	iron-chelate-transporting ATPase	N	no hit	K02013	0.014%
FOKN1_1133	putative heme iron utilization protein	N	no hit		0.095%
FOKN1_1134	multidrug ABC transporter ATPase/permease	N	no hit	K06148	n.d
FOKN1_1135	ribonucleoside-diphosphate reductase, beta subunit	N	no hit	K00526	0.009%
FOKN1_1136	ribonucleoside-diphosphate reductase alpha subunit	N	no hit	K00525	0.362%
FOKN1_1137	transcriptional regulator	N	no hit	K03576	n.d
FOKN1_1138	methionine synthase	N	no hit	K00549	0.292%
FOKN1_1139	DNA-directed RNA polymerase	N	P	K03088	n.d
FOKN1_1140	Fe2+-dicitrate sensor protein, membrane component	N	no hit	K07165	n.d
FOKN1_1141	uncharacterized protein	Y	P	K02014	0.039%
FOKN1_1142	uncharacterized protein	N	no hit		n.d
FOKN1_1143	uncharacterized protein	N	no hit		n.d
FOKN1_1144	uncharacterized protein	N	no hit		n.d
FOKN1_1145	hemin receptor	N	no hit	K19611	n.d
FOKN1_1146	uncharacterized protein	N	R		n.d
FOKN1_1147	beta-lactamase	N	R		n.d
FOKN1_1148	5'-nucleotidase/2',3'-cyclic phosphodiesterase	N	no hit	K17224	0.077%
FOKN1_1149	diheme cytochrome SoxA	N	no hit	K17222	0.339%
FOKN1_1150	sulfur oxidation protein SoxZ	N	no hit	K17227	7.121%
FOKN1_1151	sulfur oxidation protein SoxY-like protein	N	no hit	K17226	0.839%
FOKN1_1152	sulfur oxidation protein SoxX	N	no hit		n.d
FOKN1_1153	putative Zn-dependent protease	Y	no hit	K01423	n.d
FOKN1_1154	rhodanese-like protein	N	P		n.d
FOKN1_1155	redox protein	N	P	K04085	0.475%
FOKN1_1156	long-chain fatty acid transporter	Y	no hit	K06076	0.017%
FOKN1_1157	long-chain fatty acid transporter	Y	no hit		0.027%
FOKN1_1158	uncharacterized protein	Y	no hit		0.089%
FOKN1_1159	methyl-accepting chemotaxis protein	N	no hit		n.d
FOKN1_1160	methyl-accepting chemotaxis sensory transducer	N	no hit	K03406	n.d
FOKN1_1161	uncharacterized protein	Y	no hit	K21137	0.030%
FOKN1_1162	cation/multidrug efflux pump	N	no hit		n.d
FOKN1_1163	transcriptional regulator	N	no hit		0.031%
FOKN1_1164	ATPase components	N	no hit		n.d
FOKN1_1165	permease	N	no hit	K03548	n.d

Table S1. Gene annotation of gene-coding sequences (CDSs) and protein expression in the *Thiohalobacter sp.* strain FOKN1 cells. SignalP; prediction of signal peptide sequence, Y; positive, N; negative, COG; clusters of orthologous group, KO; KEGG orthology annotated using the KAAS, PCI; protein content index, n.d; not detected.

Locus_tag	Product	SignalP	COG	KO	PCI (%)
FOKN1_1166	ATPase	N	no hit	K07175	0.026%
FOKN1_1167	peroxiredoxin	N	no hit	K03564	0.176%
FOKN1_1168	glycine cleavage system regulatory protein	N	no hit	K03567	n.d
FOKN1_1169	galactose-1-phosphate uridyl transferase	N	no hit	K00965	0.034%
FOKN1_1170	dihydrodipicolinate synthase	N	no hit	K01714	0.030%
FOKN1_1171	uncharacterized protein	Y	no hit	K07287	0.147%
FOKN1_1172	metal-dependent hydrolase	N	no hit		n.d
FOKN1_1173	phosphoribosylformylglycinamidine synthase	N	no hit	K01952	0.023%
FOKN1_1174	sulfite oxidase subunit YedY	N	no hit	K07147	n.d
FOKN1_1175	phosphomannomutase	N	S		n.d
FOKN1_1176	sulfoxide reductase heme-binding subunit YedZ	N	S	K17247	n.d
FOKN1_1177	uncharacterized protein	N	no hit		n.d
FOKN1_1178	transcriptional regulator	N	no hit		0.031%
FOKN1_1179	ATPase components of ABC transporters	N	no hit		0.040%
FOKN1_1180	uncharacterized protein	N	S	K09122	n.d
FOKN1_1181	glycine/serine hydroxymethyltransferase	N	no hit	K00600	0.233%
FOKN1_1182	transcriptional regulator	N	no hit	K07738	0.034%
FOKN1_1183	pyrimidine deaminase	N	no hit	K11752	n.d
FOKN1_1184	riboflavin synthase alpha chain	N	no hit	K00793	n.d
FOKN1_1185	3,4-dihydroxy-2-butanone 4-phosphate synthase	N	no hit	K14652	0.021%
FOKN1_1186	riboflavin synthase beta-chain	N	no hit	K00794	n.d
FOKN1_1187	transcription termination factor	N	no hit	K03625	0.036%
FOKN1_1188	thiamine-monophosphate kinase	N	no hit	K00946	n.d
FOKN1_1189	phosphatidylglycerophosphatase A	N	no hit	K01095	n.d
FOKN1_1190	uncharacterized protein	N	no hit		n.d
FOKN1_1191	GTP cyclohydrolase I	N	no hit	K09007	0.082%
FOKN1_1192	6-pyruvoyl-tetrahydropterin synthase	N	H	K01737	0.033%
FOKN1_1193	1-deoxy-D-xylulose-5-phosphate synthase	N	no hit	K01662	n.d
FOKN1_1194	geranylgeranyl pyrophosphate synthase	N	no hit	K13789	n.d
FOKN1_1195	exonuclease VII small subunit	N	no hit	K03602	n.d
FOKN1_1196	transcriptional regulator	N	no hit		n.d
FOKN1_1197	ABC-type bacteriocin/lantibiotic exporter	N	no hit	K11004	n.d
FOKN1_1198	uncharacterized protein	N	no hit	K11003	n.d
FOKN1_1199	aminopeptidase C	N	no hit		n.d

Table S1. Gene annotation of gene-coding sequences (CDSs) and protein expression in the *Thiohalobacter sp.* strain FOKN1 cells. SignalP; prediction of signal peptide sequence, Y; positive, N; negative, COG; clusters of orthologous group, KO; KEGG orthology annotated using the KAAS, PCI; protein content index, n.d; not detected.

Locus_tag	Product	SignalP	COG	KO	PCI (%)
FOKN1_1200	bacteriocin	N	no hit		n.d
FOKN1_1201	catalase/hydroperoxidase	Y	no hit	K03782	0.037%
FOKN1_1202	rubrerythrin	N	no hit		n.d
FOKN1_1203	Fe-S oxidoreductase	N	no hit	K00113	n.d
FOKN1_1204	uncharacterized protein	N	no hit		n.d
FOKN1_1205	uncharacterized protein	Y	no hit		n.d
FOKN1_1206	DNA topoisomerase IV subunit B	N	no hit	K02622	0.034%
FOKN1_1207	DNA topoisomerase IV subunit A	N	no hit	K02621	0.038%
FOKN1_1208	uroporphyrinogen-III decarboxylase	N	H		n.d
FOKN1_1209	uncharacterized protein	N	no hit		n.d
FOKN1_1210	uncharacterized protein	N	no hit		n.d
FOKN1_1211	cytochrome c biogenesis factor	Y	no hit		n.d
FOKN1_1212	adenylate cyclase	N	no hit	K01768	n.d
FOKN1_1213	Zn-dependent protease	N	no hit	K03799	0.019%
FOKN1_1214	uncharacterized protein	N	no hit		0.013%
FOKN1_1215	putative diguanylate phosphodiesterase	N	no hit	K21025	0.090%
FOKN1_1216	acetyltransferase	N	no hit	K03824	0.025%
FOKN1_1217	uncharacterized protein	N	no hit	K23127	n.d
FOKN1_1218	lysine 2,3-aminomutase	N	E	K19810	n.d
FOKN1_1219	elongation factor EF-P	N	no hit	K02356	n.d
FOKN1_1220	lysyl-tRNA synthetase	N	no hit	K04568	n.d
FOKN1_1221	ABC-type transporter, permease component	N	Q	K02066	n.d
FOKN1_1222	ABC-type transporter, ATPase component	N	Q	K02065	n.d
FOKN1_1223	ABC-type transporter, periplasmic component	N	no hit	K02067	n.d
FOKN1_1224	ABC-type transporter, auxiliary component	N	no hit		n.d
FOKN1_1225	tRNA/rRNA methyltransferase	N	no hit	K15396	n.d
FOKN1_1226	serine O-acetyltransferase	N	no hit	K00640	n.d
FOKN1_1227	iron-sulfur cluster assembly transcription factor IscR	N	no hit	K13643	n.d
FOKN1_1228	uncharacterized protein	N	no hit		n.d
FOKN1_1229	nucleoside-diphosphate kinase	N	no hit	K00940	0.030%
FOKN1_1230	radical SAM protein	N	no hit	K06941	n.d
FOKN1_1231	type IV pilus biogenesis protein PilF	N	no hit	K02656	0.025%
FOKN1_1232	uncharacterized protein	N	no hit		n.d
FOKN1_1233	histidyl-tRNA synthetase	N	no hit	K01892	0.019%

Table S1. Gene annotation of gene-coding sequences (CDSs) and protein expression in the *Thiohalobacter sp.* strain FOKN1 cells. SignalP; prediction of signal peptide sequence, Y; positive, N; negative, COG; clusters of orthologous group, KO; KEGG orthology annotated using the KAAS, PCI; protein content index, n.d; not detected.

Locus_tag	Product	SignalP	COG	KO	PCI (%)
FOKN1_1234	uncharacterized protein	N	no hit		0.072%
FOKN1_1235	uncharacterized protein	Y	no hit	K17713	0.037%
FOKN1_1236	GTPase	N	no hit	K03977	0.008%
FOKN1_1237	sulfate permease	N	no hit	K03321	n.d
FOKN1_1238	ABC-type transporter, permease component	N	no hit	K02004	n.d
FOKN1_1239	ABC-type transporter, ATPase component	N	no hit	K02003	0.018%
FOKN1_1240	acyl-CoA thioesterase	Y	C	K10804	n.d
FOKN1_1241	pyruvate:ferredoxin oxidoreductase	N	C	K03737	0.017%
FOKN1_1242	dihydroorotate dehydrogenase	N	F	K00226	0.018%
FOKN1_1243	diguanylate cyclase/phosphodiesterase	N	no hit		n.d
FOKN1_1244	uncharacterized protein	Y	no hit		n.d
FOKN1_1245	exonuclease VII, large subunit	N	no hit	K03601	n.d
FOKN1_1246	IMP dehydrogenase/GMP reductase	N	no hit	K00088	0.171%
FOKN1_1247	GMP synthase	N	no hit	K01951	0.044%
FOKN1_1248	S-adenosylmethionine decarboxylase	N	no hit		n.d
FOKN1_1249	zinc transporter ZupT	N	no hit	K07238	n.d
FOKN1_1250	hydrogenase maturation factor	N	no hit		n.d
FOKN1_1251	selenocysteine protein	N	no hit	K07022	n.d
FOKN1_1252	SAM-dependent methyltransferases	N	no hit		n.d
FOKN1_1253	histidinol-phosphate aminotransferase	N	S		n.d
FOKN1_1254	uncharacterized protein	N	S		0.065%
FOKN1_1255	metal-dependent hydrolases	Y	R	K06897	n.d
FOKN1_1256	uncharacterized protein	N	no hit		n.d
FOKN1_1257	Fe2+/Zn2+ uptake regulation proteins	N	no hit		n.d
FOKN1_1258	cytosine/adenosine deaminases	N	no hit	K11991	n.d
FOKN1_1259	membrane-bound lytic murein transglycosylase F	N	no hit	K18691	n.d
FOKN1_1261	uncharacterized protein	N	P		0.056%
FOKN1_1262	dissimilatory sulfite reductase, gamma subunit	N	P		n.d
FOKN1_1263	transcriptional regulator	N	no hit		0.013%
FOKN1_1264	guanosine polyphosphate pyrophosphohydrolases/synthetases	N	O		n.d
FOKN1_1265	subtilisin-like serine proteases	Y	O	K14645	n.d
FOKN1_1266	acyl-CoA synthase	N	MG	K01904	n.d
FOKN1_1267	nucleoside-diphosphate-sugar epimerases	N	MG		n.d

Table S1. Gene annotation of gene-coding sequences (CDSs) and protein expression in the *Thiohalobacter sp.* strain FOKN1 cells. SignalP; prediction of signal peptide sequence, Y; positive, N; negative, COG; clusters of orthologous group, KO; KEGG orthology annotated using the KAAS, PCI; protein content index, n.d; not detected.

Locus_tag	Product	SignalP	COG	KO	PCI (%)
FOKN1_1268	acyl carrier protein	N	IQ		n.d
FOKN1_1269	7-keto-8-aminopelargonate synthetase	N	H	K00639	0.108%
FOKN1_1270	phosphohydrolases	N	R		n.d
FOKN1_1271	permeases	N	no hit		n.d
FOKN1_1272	permeases	N	no hit		n.d
FOKN1_1273	ABC-type multidrug transporter, ATPase and permease components	N	no hit	K06148	n.d
FOKN1_1274	nucleoside-diphosphate-sugar epimerase	N	M		n.d
FOKN1_1275	lauroyl/myristoyl acyltransferase	N	M		0.023%
FOKN1_1276	sterol desaturase	N	M	K19703	n.d
FOKN1_1277	lauroyl/myristoyl acyltransferase	N	M	K02517	n.d
FOKN1_1278	uncharacterized protein	N	M		n.d
FOKN1_1279	uncharacterized protein	N	no hit		n.d
FOKN1_1280	cobalamin-5-phosphate synthase	N	H		n.d
FOKN1_1281	putative sulfotransferase protein	N	no hit		n.d
FOKN1_1282	nucleoside-diphosphate-sugar epimerase	N	no hit	K07276	n.d
FOKN1_1283	uncharacterized protein	N	no hit		0.076%
FOKN1_1284	uncharacterized protein	N	no hit		n.d
FOKN1_1285	ABC-type metal ion transporter, periplasmic component/surface adhesin	N	no hit		n.d
FOKN1_1286	uncharacterized protein	N	no hit		n.d
FOKN1_1287	uncharacterized protein	Y	no hit		n.d
FOKN1_1288	nucleoside-diphosphate-sugar epimerase	N	M		n.d
FOKN1_1289	putative sulfotransferase protein	N	M	K22523	0.039%
FOKN1_1290	uncharacterized protein	N	M		0.023%
FOKN1_1291	periplasmic protein TonB	N	U		n.d
FOKN1_1292	biopolymer transport protein	N	U		n.d
FOKN1_1293	biopolymer transport protein	N	U		n.d
FOKN1_1294	biopolymer transport proteins	N	U		0.038%
FOKN1_1295	tonB-dependent receptor protein	Y	no hit	K16091	0.523%
FOKN1_1296	transposase and inactivated derivatives	Y	no hit		n.d
FOKN1_1297	putative sulfotransferase protein	N	no hit		0.038%
FOKN1_1298	O-antigen polymerase	N	no hit		n.d
FOKN1_1299	putative sulfotransferase protein	N	no hit	K22523	n.d

Table S1. Gene annotation of gene-coding sequences (CDSs) and protein expression in the *Thiohalobacter sp.* strain FOKN1 cells. SignalP; prediction of signal peptide sequence, Y; positive, N; negative, COG; clusters of orthologous group, KO; KEGG orthology annotated using the KAAS, PCI; protein content index, n.d; not detected.

Locus_tag	Product	SignalP	COG	KO	PCI (%)
FOKN1_1300	uncharacterized protein	N	no hit	K05303	0.040%
FOKN1_1301	SAM-dependent methyltransferases	N	no hit		n.d
FOKN1_1302	cytochrome c	Y	no hit	K19713	n.d
FOKN1_1303	cytochrome c553	N	no hit		n.d
FOKN1_1304	SAM-dependent methyltransferases	N	no hit		n.d
FOKN1_1305	uncharacterized protein	N	no hit		n.d
FOKN1_1306	sulfite reductase alpha subunit	N	C	K11180	n.d
FOKN1_1307	sulfite reductase beta subunit	N	C	K11181	n.d
FOKN1_1308	cyclopropane fatty acyl phospholipid synthase	N	C	K00574	n.d
FOKN1_1309	plasmid stability protein	N	no hit	K21495	n.d
FOKN1_1310	plasmid stability protein	N	no hit	K07062	n.d
FOKN1_1311	Zn-dependent hydrolases	Y	no hit		0.012%
FOKN1_1312	chromate transporter	N	no hit	K07240	n.d
FOKN1_1313	uncharacterized protein	N	no hit		n.d
FOKN1_1314	uncharacterized protein	N	no hit		n.d
FOKN1_1315	uncharacterized protein	Y	no hit		n.d
FOKN1_1316	ABC-type multidrug transporter, ATPase and permease components	N	no hit		n.d
FOKN1_1317	uncharacterized protein	N	no hit		0.008%
FOKN1_1318	silver efflux pump	N	P		n.d
FOKN1_1319	uncharacterized protein	N	P		n.d
FOKN1_1320	uncharacterized protein	N	no hit		n.d
FOKN1_1321	helicases	N	no hit		n.d
FOKN1_1322	uncharacterized protein	N	R		n.d
FOKN1_1323	uncharacterized protein	N	no hit		n.d
FOKN1_1324	uncharacterized protein	N	no hit		0.020%
FOKN1_1325	CRISPR-associated protein	N	no hit		0.170%
FOKN1_1326	uncharacterized protein	N	no hit		n.d
FOKN1_1327	CRISPR-associated endonuclease	N	no hit		n.d
FOKN1_1328	uncharacterized protein	N	no hit		n.d
FOKN1_1329	uncharacterized protein	N	no hit		n.d
FOKN1_1330	uncharacterized protein	N	no hit		n.d
FOKN1_1331	uncharacterized protein	N	no hit		n.d
FOKN1_1332	uncharacterized protein	N	no hit		n.d

Table S1. Gene annotation of gene-coding sequences (CDSs) and protein expression in the *Thiohalobacter sp.* strain FOKN1 cells. SignalP; prediction of signal peptide sequence, Y; positive, N; negative, COG; clusters of orthologous group, KO; KEGG orthology annotated using the KAAS, PCI; protein content index, n.d; not detected.

Locus_tag	Product	SignalP	COG	KO	PCI (%)
FOKN1_1333	uncharacterized protein	N	S		n.d
FOKN1_1334	cytochrome bd-type quinol oxidase, subunit 2	N	S		n.d
FOKN1_1335	exonuclease III	N	L		0.009%
FOKN1_1336	aminopeptidase	N	E		n.d
FOKN1_1337	alanine racemase	N	no hit		n.d
FOKN1_1338	AAA ATPase central domain protein	N	no hit		n.d
FOKN1_1339	ATPase	N	no hit		n.d
FOKN1_1340	phosphopantetheinyl transferase	N	Q		n.d
FOKN1_1341	acetate kinase	N	no hit		n.d
FOKN1_1342	transcriptional factor	N	no hit		n.d
FOKN1_1343	uncharacterized protein	N	no hit		n.d
FOKN1_1344	glycerol uptake facilitator and related permeases	N	no hit		0.013%
FOKN1_1345	response regulator	N	T		n.d
FOKN1_1346	type I site-specific restriction-modification system, R (restriction) subunit	N	no hit	K01153	n.d
FOKN1_1347	type I site-specific restriction-modification system, R (restriction) subunit	N	no hit		n.d
FOKN1_1348	DNA recombination protein rmuC	N	no hit	K09760	0.010%
FOKN1_1349	type I restriction-modification system methyltransferase subunit	N	no hit	K03427	n.d
FOKN1_1350	restriction endonuclease S subunits	N	no hit	K01154	n.d
FOKN1_1351	5'-nucleotidase	N	no hit	K01081	0.018%
FOKN1_1352	uncharacterized protein	N	no hit		0.011%
FOKN1_1353	uncharacterized protein	N	no hit		n.d
FOKN1_1354	flagellar motor protein	N	no hit		n.d
FOKN1_1355	uncharacterized protein	N	no hit		n.d
FOKN1_1356	DNA/RNA helicases	N	no hit	K11654	n.d
FOKN1_1357	ATPase	N	no hit		n.d
FOKN1_1358	signal transduction histidine kinase	N	no hit		n.d
FOKN1_1359	uncharacterized protein	N	no hit		n.d
FOKN1_1360	endonuclease I	N	no hit	K01150	n.d
FOKN1_1361	apolipoprotein N-acyltransferase	N	no hit		n.d
FOKN1_1362	ATPase	N	IQ		n.d
FOKN1_1363	3-oxoacyl-(acyl-carrier-protein) synthase	N	IQ		0.031%

Table S1. Gene annotation of gene-coding sequences (CDSs) and protein expression in the *Thiohalobacter sp.* strain FOKN1 cells. SignalP; prediction of signal peptide sequence, Y; positive, N; negative, COG; clusters of orthologous group, KO; KEGG orthology annotated using the KAAS, PCI; protein content index, n.d; not detected.

Locus_tag	Product	SignalP	COG	KO	PCI (%)
FOKN1_1364	short-chain alcohol dehydrogenase	N	no hit		n.d
FOKN1_1365	glycosidases	N	G		n.d
FOKN1_1366	ADP-ribose pyrophosphatase	N	G		n.d
FOKN1_1367	uncharacterized protein	N	G		n.d
FOKN1_1368	uncharacterized protein	N	S		n.d
FOKN1_1369	dihydroorotase and related cyclic amidohydrolases	N	no hit		n.d
FOKN1_1370	uncharacterized protein	N	no hit		n.d
FOKN1_1371	integrase	N	no hit		n.d
FOKN1_1372	geranylgeranyl pyrophosphate synthase	N	no hit		n.d
FOKN1_1373	phosphoglyceromutase	N	K		n.d
FOKN1_1374	uncharacterized protein	N	K		n.d
FOKN1_1375	prophage maintenance system killer protein	N	K		n.d
FOKN1_1376	transcriptional regulators	N	K		0.099%
FOKN1_1377	DNA polymerase elongation subunit	N	L		n.d
FOKN1_1378	DNA/RNA helicases	N	K		n.d
FOKN1_1379	transcription regulator	N	K		n.d
FOKN1_1380	uncharacterized protein	N	L		n.d
FOKN1_1381	uncharacterized protein	N	L		n.d
FOKN1_1382	ATP-dependent exonuclease V, alpha subunit	N	L		n.d
FOKN1_1383	histone acetyltransferase HPA2	N	KR		n.d
FOKN1_1384	phosphoesterase	N	no hit		n.d
FOKN1_1385	uncharacterized protein	N	no hit		n.d
FOKN1_1386	uncharacterized protein	N	no hit		n.d
FOKN1_1387	transcription regulator	N	no hit		n.d
FOKN1_1388	O-methyltransferase	N	no hit		n.d
FOKN1_1389	exonuclease RNase T and DNA polymerase III	N	no hit		n.d
FOKN1_1390	serine/threonine protein phosphatase	N	no hit		n.d
FOKN1_1391	transcriptional regulator	N	no hit		n.d
FOKN1_1392	serine/threonine protein phosphatase	N	I		n.d
FOKN1_1393	chemotaxis protein histidine kinase	N	I		n.d
FOKN1_1394	serine/threonine protein phosphatase	N	I	K17505	n.d
FOKN1_1395	acyl-CoA dehydrogenase	N	I		n.d
FOKN1_1396	3'-5' exonuclease	N	no hit		n.d
FOKN1_1397	amidases	N	no hit		n.d

Table S1. Gene annotation of gene-coding sequences (CDSs) and protein expression in the *Thiohalobacter sp.* strain FOKN1 cells. SignalP; prediction of signal peptide sequence, Y; positive, N; negative, COG; clusters of orthologous group, KO; KEGG orthology annotated using the KAAS, PCI; protein content index, n.d; not detected.

Locus_tag	Product	SignalP	COG	KO	PCI (%)
FOKN1_1398	phosphoribulokinase	N	no hit		n.d
FOKN1_1399	integrase	N	no hit		0.019%
FOKN1_1401	uncharacterized protein	N	no hit		n.d
FOKN1_1402	uncharacterized protein	N	no hit		n.d
FOKN1_1403	20S proteasome subunits A/B	N	no hit	K07395	0.051%
FOKN1_1404	uncharacterized protein	N	no hit		0.015%
FOKN1_1405	uncharacterized protein	N	no hit		0.049%
FOKN1_1406	NTP pyrophosphohydrolases	N	no hit		n.d
FOKN1_1407	ABC-type multidrug transporter, permease component	N	no hit	K09694	n.d
FOKN1_1408	ABC-type multidrug transporter, ATPase component	N	no hit	K09695	n.d
FOKN1_1409	Na+/H+ antiporter	N	no hit		n.d
FOKN1_1410	transcription elongation factor	N	no hit	K04760	0.022%
FOKN1_1411	3'-phosphate cyclase	N	no hit	K01974	n.d
FOKN1_1412	uncharacterized protein	N	no hit		n.d
FOKN1_1413	uncharacterized protein	N	no hit		n.d
FOKN1_1414	dinucleotide-utilizing enzyme	N	no hit	K22132	n.d
FOKN1_1415	spermidine synthase	N	no hit		n.d
FOKN1_1416	ferredoxin-NADP reductase	N	L	K00528	0.025%
FOKN1_1417	deoxyribodipyrimidine photolyase	N	L	K01669	n.d
FOKN1_1418	thiol-disulfide isomerase and thioredoxins	N	OC		n.d
FOKN1_1419	peptide methionine sulfoxide reductase	N	O	K07304	n.d
FOKN1_1420	uncharacterized protein	N	S		n.d
FOKN1_1421	phosphatidylinositol-4-phosphate 5-kinase	N	S		n.d
FOKN1_1422	Kef-type K+ transporter	N	no hit	K10716	n.d
FOKN1_1423	uncharacterized protein	N	no hit		n.d
FOKN1_1424	response regulator receiver	N	no hit		n.d
FOKN1_1425	radical SAM protein	N	no hit		n.d
FOKN1_1426	uncharacterized protein	N	S		n.d
FOKN1_1427	uncharacterized protein	N	R		n.d
FOKN1_1428	metallophosphoesterase	N	R	K07098	n.d
FOKN1_1429	CDP-diglyceride synthetase	N	I		n.d
FOKN1_1430	serine phosphatase	N	TK	K07315	n.d
FOKN1_1431	serine phosphatase	N	TK		n.d
FOKN1_1432	anti-sigma factor antagonist	N	TK		n.d

Table S1. Gene annotation of gene-coding sequences (CDSs) and protein expression in the *Thiohalobacter sp.* strain FOKN1 cells. SignalP; prediction of signal peptide sequence, Y; positive, N; negative, COG; clusters of orthologous group, KO; KEGG orthology annotated using the KAAS, PCI; protein content index, n.d; not detected.

Locus_tag	Product	SignalP	COG	KO	PCI (%)
FOKN1_1433	ABC-type transporter, permease component	N	no hit	K02066	n.d
FOKN1_1434	ABC-type transporter, ATPase component	N	Q	K02065	n.d
FOKN1_1435	ABC-type transporter, periplasmic component	N	Q		n.d
FOKN1_1436	uncharacterized protein	Y	Q		n.d
FOKN1_1437	auxin efflux carrier	N	no hit		n.d
FOKN1_1438	guanosine polyphosphate pyrophosphohydrolases/synthetases	N	no hit	K00951	0.005%
FOKN1_1439	aspartate-semialdehyde dehydrogenase	N	E		n.d
FOKN1_1440	signal transduction histidine kinase	N	T	K02486	n.d
FOKN1_1441	response regulator receiver protein	N	T	K02485	n.d
FOKN1_1442	chloride channel protein EriC	N	no hit		n.d
FOKN1_1444	type IV pilus assembly PilZ	N	no hit		n.d
FOKN1_1445	flagellar biosynthesis/type III secretory pathway chaperone	N	no hit		n.d
FOKN1_1446	negative regulator	N	no hit		n.d
FOKN1_1447	flagellar basal body P-ring biosynthesis protein-like protein	N	no hit	K02386	n.d
FOKN1_1448	chemotaxis protein CheV	N	no hit	K03415	n.d
FOKN1_1449	chemotaxis protein methyltransferase	N	no hit	K00575	n.d
FOKN1_1450	flagellar basal body rod protein FlgB	N	no hit	K02387	n.d
FOKN1_1451	flagellar basal-body rod protein FlgC	N	no hit	K02388	n.d
FOKN1_1452	flagellar hook capping protein	N	no hit	K02389	n.d
FOKN1_1453	flagellar basal body FlaE domain protein	N	no hit	K02390	n.d
FOKN1_1454	flagellar basal-body rod protein FlgF	N	no hit	K02391	n.d
FOKN1_1455	flagellar basal-body rod protein FlgG	N	no hit	K02392	n.d
FOKN1_1456	flagellar L-ring protein	Y	no hit	K02393	n.d
FOKN1_1457	flagellar P-ring protein	Y	no hit	K02394	n.d
FOKN1_1458	flagellar rod assembly protein/muramidase FlgJ	N	no hit	K02395	0.011%
FOKN1_1459	flagellar hook-associated protein FlgK	N	no hit	K02396	n.d
FOKN1_1460	flagellar hook-associated protein 3 FlgL	N	no hit	K02397	n.d
FOKN1_1461	signal transduction protein	N	no hit		0.008%
FOKN1_1462	flagellin	N	no hit	K02406	0.027%
FOKN1_1463	flagellar protein	N	no hit	K06603	n.d
FOKN1_1464	flagellar capping protein	N	no hit	K02407	n.d

Table S1. Gene annotation of gene-coding sequences (CDSs) and protein expression in the *Thiohalobacter sp.* strain FOKN1 cells. SignalP; prediction of signal peptide sequence, Y; positive, N; negative, COG; clusters of orthologous group, KO; KEGG orthology annotated using the KAAS, PCI; protein content index, n.d; not detected.

Locus_tag	Product	SignalP	COG	KO	PCI (%)
FOKN1_1465	flagellin-specific chaperone	N	no hit	K02422	n.d
FOKN1_1466	uncharacterized protein	N	no hit		n.d
FOKN1_1467	serine/threonine protein kinase	N	no hit		n.d
FOKN1_1468	response regulator	N	no hit	K10941	0.077%
FOKN1_1469	signal transduction histidine kinase	N	no hit	K10942	0.010%
FOKN1_1470	flagellar regulatory protein FleQ	N	no hit	K10943	0.012%
FOKN1_1471	flagellar hook-basal body complex protein FliE	N	no hit	K02408	n.d
FOKN1_1472	flagellar M-ring protein	N	no hit	K02409	n.d
FOKN1_1473	flagellar motor switch protein FliG	N	no hit	K02410	n.d
FOKN1_1474	flagellar assembly protein FliH	N	no hit	K02411	n.d
FOKN1_1475	flagellar protein export ATPase FliI	N	no hit	K02412	n.d
FOKN1_1476	flagellar export protein FliJ	N	no hit		n.d
FOKN1_1477	flagellar hook-length control protein	N	no hit	K02414	n.d
FOKN1_1478	signal transduction protein	N	no hit		n.d
FOKN1_1479	uncharacterized protein	N	no hit		n.d
FOKN1_1480	uncharacterized protein	N	no hit		n.d
FOKN1_1481	uncharacterized protein	N	no hit		n.d
FOKN1_1482	DNA/RNA helicases	N	no hit	K11927	n.d
FOKN1_1483	spermidine synthase	N	no hit		n.d
FOKN1_1484	uncharacterized protein	N	no hit	K09912	n.d
FOKN1_1485	flagellar basal body-associated protein FliL	N	no hit	K02415	n.d
FOKN1_1486	flagellar motor switch protein FliM	N	no hit	K02416	n.d
FOKN1_1487	surface presentation of antigens (SpoA) protein	N	no hit	K02417	n.d
FOKN1_1488	flagellar biosynthesis protein FliO	N	no hit		n.d
FOKN1_1489	flagellar biosynthetic protein FliP	Y	no hit	K02419	n.d
FOKN1_1490	flagellar biosynthesis pathway, component FliQ	N	no hit	K02420	n.d
FOKN1_1491	flagellar biosynthetic protein fliR	N	no hit	K02421	n.d
FOKN1_1492	flagellar biosynthetic protein FlhB	N	no hit	K02401	n.d
FOKN1_1493	flagellar biosynthesis protein FlhA	N	no hit	K02400	n.d
FOKN1_1494	flagellar biosynthesis protein FlhF	N	no hit	K02404	n.d
FOKN1_1495	cobyrinic acid a, c-diamide synthase	N	no hit	K04562	n.d
FOKN1_1496	DNA-directed RNA polymerase	N	no hit	K02405	n.d
FOKN1_1497	chemotaxis protein CheY	N	no hit	K03413	n.d
FOKN1_1498	protein phosphatase CheZ	N	no hit	K03414	n.d

Table S1. Gene annotation of gene-coding sequences (CDSs) and protein expression in the *Thiohalobacter sp.* strain FOKN1 cells. SignalP; prediction of signal peptide sequence, Y; positive, N; negative, COG; clusters of orthologous group, KO; KEGG orthology annotated using the KAAS, PCI; protein content index, n.d; not detected.

Locus_tag	Product	SignalP	COG	KO	PCI (%)
FOKN1_1499	cheA signal transduction histidine kinase	N	no hit	K03407	n.d
FOKN1_1500	chemotaxis response regulator	N	no hit	K03412	n.d
FOKN1_1501	ATPases	N	no hit	K03496	n.d
FOKN1_1502	uncharacterized protein	N	no hit	K03408	n.d
FOKN1_1503	uncharacterized protein	N	no hit		n.d
FOKN1_1504	chemotaxis signal transduction protein	N	no hit	K03408	0.038%
FOKN1_1505	DNA-directed RNA polymerase	N	no hit		n.d
FOKN1_1506	uncharacterized protein	N	no hit		n.d
FOKN1_1507	ATP-dependent DNA ligase	N	no hit	K01971	n.d
FOKN1_1508	flagellar protein FhlB-like protein	N	no hit	K04061	n.d
FOKN1_1509	uncharacterized protein	N	no hit		n.d
FOKN1_1510	uncharacterized protein	N	R		0.045%
FOKN1_1511	flavoproteins	N	R		n.d
FOKN1_1512	transcriptional regulator	N	no hit	K22041	n.d
FOKN1_1513	uncharacterized protein	N	no hit		n.d
FOKN1_1514	uncharacterized protein	N	no hit	K09141	n.d
FOKN1_1515	dioxygenase	N	R	K06990	n.d
FOKN1_1516	pyruvate formate lyase-activating enzyme PflA	N	no hit	K04069	0.010%
FOKN1_1517	K+ transporter	N	no hit		n.d
FOKN1_1518	uncharacterized protein	Y	R		n.d
FOKN1_1519	1,4-alpha-glucan-branching protein	N	no hit	K00700	0.028%
FOKN1_1520	ADP-glucose pyrophosphorylase	N	no hit	K00975	0.039%
FOKN1_1521	alpha-amylase/alpha-mannosidase	N	G		0.012%
FOKN1_1522	4-alpha-glucanotransferase	N	G	K00705	n.d
FOKN1_1523	glucan phosphorylase	N	G	K00688	0.019%
FOKN1_1525	S-adenosylmethionine-tRNA ribosyltransferase-isomerase	N	no hit	K07568	n.d
FOKN1_1526	queuine tRNA-ribosyltransferase	N	no hit	K00773	n.d
FOKN1_1527	preprotein translocase subunit YajC	N	no hit	K03210	0.460%
FOKN1_1528	preprotein translocase subunit SecD	N	no hit	K03072	0.006%
FOKN1_1529	protein-export membrane protein SecF	N	no hit	K03074	n.d
FOKN1_1530	Co/Z/Cd efflux system component	N	no hit		n.d
FOKN1_1531	Fe-S-cluster oxidoreductase	N	no hit		n.d
FOKN1_1532	uncharacterized protein	N	no hit		n.d
FOKN1_1533	fructose-1,6-bisphosphatase	N	no hit	K01092	0.091%

Table S1. Gene annotation of gene-coding sequences (CDSs) and protein expression in the *Thiohalobacter sp.* strain FOKN1 cells. SignalP; prediction of signal peptide sequence, Y; positive, N; negative, COG; clusters of orthologous group, KO; KEGG orthology annotated using the KAAS, PCI; protein content index, n.d; not detected.

Locus_tag	Product	SignalP	COG	KO	PCI (%)
FOKN1_1534	phosphatidylserine decarboxylase	N	no hit	K01613	0.018%
FOKN1_1535	uncharacterized protein	Y	S	K09796	n.d
FOKN1_1536	uncharacterized protein	N	S	K07152	n.d
FOKN1_1537	N5-glutamine S-adenosyl-L-methionine-dependent methyltransferase	N	no hit	K07320	n.d
FOKN1_1538	coproporphyrinogen III oxidase	N	no hit	K02495	0.008%
FOKN1_1539	intein/homing endonuclease	N	no hit		n.d
FOKN1_1540	chorismate synthase	N	no hit	K01736	0.027%
FOKN1_1541	response regulator receiver	N	T		n.d
FOKN1_1542	permease	N	no hit	K05820	n.d
FOKN1_1543	fructose-bisphosphate aldolase	N	no hit	K11645	0.152%
FOKN1_1544	diguanylate cyclase/phosphodiesterase	N	no hit		n.d
FOKN1_1545	ABC-type phosphate/phosphonate transporter, periplasmic component	Y	P		n.d
FOKN1_1546	transcriptional regulator, LysR family	N	no hit		n.d
FOKN1_1547	3-isopropylmalate dehydratase large subunit	N	no hit	K01703	0.012%
FOKN1_1548	3-isopropylmalate dehydratase small subunit	N	no hit	K01704	0.018%
FOKN1_1549	isocitrate/isopropylmalate dehydrogenase	N	no hit	K00052	0.017%
FOKN1_1550	aspartate-semialdehyde dehydrogenase	N	no hit	K00133	0.030%
FOKN1_1551	TfP pilus assembly protein FimV	Y	no hit	K08086	0.053%
FOKN1_1552	uncharacterized protein	Y	no hit		n.d
FOKN1_1553	transcription antiterminator	N	no hit		n.d
FOKN1_1554	uncharacterized protein	N	no hit		0.047%
FOKN1_1555	ABC-type cobalt transporter, permease component CbiQ	N	P		n.d
FOKN1_1556	pseudouridylate synthase	N	no hit	K06173	n.d
FOKN1_1557	phosphoribosylanthranilate isomerase	N	no hit	K01817	n.d
FOKN1_1558	tryptophan synthase beta chain	N	no hit	K01696	0.032%
FOKN1_1559	tryptophan synthase alpha chain	N	no hit	K01695	0.106%
FOKN1_1560	acetyl-CoA carboxylase beta subunit	N	no hit	K01963	0.027%
FOKN1_1561	folylpolyglutamate synthetase	N	no hit	K11754	n.d
FOKN1_1562	uncharacterized protein	N	no hit		0.021%
FOKN1_1563	colicin V production protein	N	no hit	K03558	n.d
FOKN1_1564	glutamine phosphoribosyl pyrophosphate amidotransferase	N	no hit	K00764	0.044%

Table S1. Gene annotation of gene-coding sequences (CDSs) and protein expression in the *Thiohalobacter sp.* strain FOKN1 cells. SignalP; prediction of signal peptide sequence, Y; positive, N; negative, COG; clusters of orthologous group, KO; KEGG orthology annotated using the KAAS, PCI; protein content index, n.d; not detected.

Locus_tag	Product	SignalP	COG	KO	PCI (%)
FOKN1_1565	cystathionine beta-lyases/cystathionine gamma-synthases	N	no hit	K10764	0.036%
FOKN1_1566	putative archaeal flagellar protein G	N	no hit		n.d
FOKN1_1567	hydroxypyruvate reductase	N	no hit	K11529	n.d
FOKN1_1568	uncharacterized protein	N	no hit		n.d
FOKN1_1569	uncharacterized protein	Y	no hit		n.d
FOKN1_1570	uncharacterized protein	Y	no hit		n.d
FOKN1_1571	UDP-2,3-diacylglucosamine hydrolase	N	no hit	K03269	n.d
FOKN1_1572	peptidyl-prolyl cis-trans isomerase	Y	no hit	K03768	0.088%
FOKN1_1573	glutamate-tRNA ligase	N	J	K09698	n.d
FOKN1_1574	cysteinyl-tRNA synthetase	N	J	K01883	0.052%
FOKN1_1575	permease	N	T		n.d
FOKN1_1576	diguanylate cyclase/phosphodiesterase	N	T		n.d
FOKN1_1577	5,10-methylene-tetrahydrofolate dehydrogenase/methenyl tetrahydrofolate cyclohydrolase	N	no hit	K01491	n.d
FOKN1_1583	peptidyl-prolyl cis-trans isomerase	N	no hit	K03545	0.028%
FOKN1_1584	ATP-dependent Clp protease proteolytic subunit	N	no hit	K01358	0.101%
FOKN1_1585	ATP-dependent protease ATP-binding subunit	N	no hit	K03544	0.101%
FOKN1_1586	ATP-dependent Lon protease	N	no hit	K01338	0.109%
FOKN1_1587	DNA-binding transcriptional regulator subunit beta	N	no hit	K03530	0.060%
FOKN1_1590	peptidyl-prolyl cis-trans isomerase	N	no hit	K03770	0.057%
FOKN1_1591	Na+/H+ dicarboxylate symporter	N	C	K11102	n.d
FOKN1_1592	enoyl-[acyl-carrier-protein] reductase	N	C	K00208	0.201%
FOKN1_1593	ABC-type dipeptide transporter, periplasmic component	N	E	K15580	n.d
FOKN1_1594	ABC-type dipeptide/oligopeptide/nickel transporters, permease components	N	E	K15581	n.d
FOKN1_1595	ABC-type dipeptide/oligopeptide/nickel transporters, permease components	N	E	K02034	n.d
FOKN1_1596	peptide ABC transporter ATPase	N	E	K02032	0.017%
FOKN1_1597	subtilisin-like serine proteases	Y	no hit		n.d
FOKN1_1598	serine-pyruvate aminotransferase	N	no hit	K00830	0.095%
FOKN1_1599	phage-related tail protein	N	no hit		n.d
FOKN1_1600	alpha/beta hydrolase	N	no hit	K07019	n.d
FOKN1_1601	ABC-type dipeptide/oligopeptide/nickel transporters, permease components	N	EP		n.d

Table S1. Gene annotation of gene-coding sequences (CDSs) and protein expression in the *Thiohalobacter sp.* strain FOKN1 cells. SignalP; prediction of signal peptide sequence, Y; positive, N; negative, COG; clusters of orthologous group, KO; KEGG orthology annotated using the KAAS, PCI; protein content index, n.d; not detected.

Locus_tag	Product	SignalP	COG	KO	PCI (%)
FOKN1_1602	uncharacterized protein	N	no hit		n.d
FOKN1_1603	uncharacterized protein	N	no hit		n.d
FOKN1_1604	uncharacterized protein	Y	no hit		n.d
FOKN1_1605	cold-shock DNA-binding domain-containing protein	N	no hit	K03704	n.d
FOKN1_1606	cell division protein FtsI	N	no hit		n.d
FOKN1_1607	uncharacterized protein	N	no hit		n.d
FOKN1_1608	uncharacterized protein	N	no hit	K15034	n.d
FOKN1_1609	uncharacterized protein	N	no hit	K18912	n.d
FOKN1_1610	methyltransferase	N	no hit	K18911	n.d
FOKN1_1611	ATP-dependent DNA helicase RecQ	N	no hit	K03654	n.d
FOKN1_1612	ATP-dependent DNA helicase RecQ	N	no hit	K03654	n.d
FOKN1_1613	uncharacterized protein	N	no hit		n.d
FOKN1_1614	cytochrome c553	Y	no hit		n.d
FOKN1_1615	cytochrome c553	Y	no hit		n.d
FOKN1_1616	Zn-dependent protease	N	R		n.d
FOKN1_1617	uncharacterized protein	Y	R		n.d
FOKN1_1618	uncharacterized protein	Y	R		n.d
FOKN1_1619	sulfotransferase	N	no hit		n.d
FOKN1_1620	glycosyltransferase	N	M	K19354	n.d
FOKN1_1621	acetylornithine deacetylase/succinyl-diaminopimelate desuccinylase	N	R	K01438	n.d
FOKN1_1622	acetyltransferases	N	R		0.023%
FOKN1_1623	S-adenosylhomocysteine hydrolase	N	H	K01251	0.009%
FOKN1_1624	SAM-dependent methyltransferases	N	QR	K03183	n.d
FOKN1_1625	cyclopropane fatty acid synthase	N	QR	K00574	n.d
FOKN1_1626	geranylgeranyl reductase	N	QR	K17830	0.010%
FOKN1_1627	uncharacterized protein	N	no hit		n.d
FOKN1_1628	F0F1-type ATP synthase, beta subunit	N	no hit		n.d
FOKN1_1629	excinuclease ABC subunit A	N	no hit		0.013%
FOKN1_1630	restriction endonuclease	N	E		n.d
FOKN1_1631	methionine synthase II	N	E		n.d
FOKN1_1632	glutathione S-transferase P subunit	N	no hit	K00799	n.d
FOKN1_1633	catalase/hydroperoxidase	N	no hit	K03782	n.d
FOKN1_1634	cell division GTPase	Y	no hit		0.012%

Table S1. Gene annotation of gene-coding sequences (CDSs) and protein expression in the *Thiohalobacter sp.* strain FOKN1 cells. SignalP; prediction of signal peptide sequence, Y; positive, N; negative, COG; clusters of orthologous group, KO; KEGG orthology annotated using the KAAS, PCI; protein content index, n.d; not detected.

Locus_tag	Product	SignalP	COG	KO	PCI (%)
FOKN1_1635	uncharacterized protein	N	no hit		n.d
FOKN1_1636	uncharacterized protein	N	no hit		n.d
FOKN1_1637	uncharacterized protein	N	no hit		n.d
FOKN1_1638	uncharacterized protein	Y	no hit		n.d
FOKN1_1639	glutaredoxin	N	O		n.d
FOKN1_1640	rhodanese-related sulfurtransferase	N	no hit		n.d
FOKN1_1641	sterol desaturase	N	no hit		n.d
FOKN1_1642	uncharacterized protein	N	S		n.d
FOKN1_1643	radical SAM domain protein	N	S		n.d
FOKN1_1644	glycosyltransferase	N	S		n.d
FOKN1_1645	uncharacterized protein	N	S	K09931	n.d
FOKN1_1646	SAM-dependent methyltransferases	N	no hit	K07755	n.d
FOKN1_1647	thiol-disulfide isomerase and thioredoxins	N	OC	K03672	0.048%
FOKN1_1648	diguanylate cyclase/phosphodiesterase	N	P		n.d
FOKN1_1649	phosphate/sulphate permeases	N	P	K03306	n.d
FOKN1_1650	uncharacterized protein	Y	no hit		n.d
FOKN1_1651	transcriptional regulators	N	no hit		n.d
FOKN1_1652	zinc/iron permease	N	O		n.d
FOKN1_1653	peroxiredoxin	N	O	K03386	0.093%
FOKN1_1654	uncharacterized protein	N	no hit		n.d
FOKN1_1655	rhodanese-related sulfurtransferase	N	no hit		n.d
FOKN1_1656	sodium/calcium exchanger membrane region	N	no hit		n.d
FOKN1_1657	Na+/Ca+ antiporter	N	no hit	K07301	n.d
FOKN1_1658	isocitrate dehydrogenase	N	no hit	K00031	0.048%
FOKN1_1659	uncharacterized protein	N	T	K21088	n.d
FOKN1_1660	exonuclease I	N	T	K01141	n.d
FOKN1_1661	Fe-S oxidoreductase	N	C		0.075%
FOKN1_1662	adenosine phosphosulfate reductase subunit beta	N	C	K00395	0.578%
FOKN1_1663	adenylylsulfate reductase subunit alpha	N	C	K00394	1.075%
FOKN1_1664	ABC-type metal ion transporter, periplasmic component/surface antigen	N	C		0.054%
FOKN1_1665	peptidyl-prolyl cis-trans isomerase	N	no hit		n.d
FOKN1_1666	alpha-mannosidase	N	no hit		n.d
FOKN1_1667	polyribonucleotide nucleotidyltransferase	N	no hit		n.d

Table S1. Gene annotation of gene-coding sequences (CDSs) and protein expression in the *Thiohalobacter sp.* strain FOKN1 cells. SignalP; prediction of signal peptide sequence, Y; positive, N; negative, COG; clusters of orthologous group, KO; KEGG orthology annotated using the KAAS, PCI; protein content index, n.d; not detected.

Locus_tag	Product	SignalP	COG	KO	PCI (%)
FOKN1_1668	uncharacterized protein	N	no hit		n.d
FOKN1_1669	Na+/H+ and K+/H+ antiporters	N	no hit		n.d
FOKN1_1670	ATPase	N	no hit		n.d
FOKN1_1671	uncharacterized protein	N	no hit		n.d
FOKN1_1672	uncharacterized protein	N	no hit		n.d
FOKN1_1673	Fe-S oxidoreductase	N	no hit		n.d
FOKN1_1674	transcriptional regulator	N	no hit	K07733	n.d
FOKN1_1675	single-stranded DNA-specific exonuclease	N	no hit		n.d
FOKN1_1676	uncharacterized protein	N	no hit		n.d
FOKN1_1677	uncharacterized protein	N	no hit		n.d
FOKN1_1678	DNA/RNA helicases	N	no hit		n.d
FOKN1_1679	uncharacterized protein	N	no hit		n.d
FOKN1_1680	DNA methylase	N	no hit	K07316	n.d
FOKN1_1681	restriction endonuclease	N	no hit	K01156	0.015%
FOKN1_1682	integrase	N	no hit		n.d
FOKN1_1683	uncharacterized protein	N	no hit	K03664	n.d
FOKN1_1684	Na+-dependent transporters	N	R	K03308	n.d
FOKN1_1685	oligoketide cyclase/lipid transport protein	N	no hit		n.d
FOKN1_1686	uncharacterized protein	N	S	K09801	n.d
FOKN1_1687	uncharacterized protein	N	no hit	K06186	n.d
FOKN1_1688	DNA-binding transcriptional dual regulator	N	no hit	K03711	0.100%
FOKN1_1689	DNA repair protein RecN	N	no hit	K03631	0.015%
FOKN1_1690	NAD kinase	N	no hit	K00858	n.d
FOKN1_1691	heat-inducible transcription repressor HrcA	N	no hit	K03705	n.d
FOKN1_1692	molecular chaperone GrpE	N	no hit	K03687	n.d
FOKN1_1693	chaperone Hsp70, co-chaperone with DnaJ	N	no hit	K04043	0.243%
FOKN1_1694	chaperone protein DnaJ	N	no hit	K03686	n.d
FOKN1_1695	dihydrodipicolinate reductase	N	no hit	K00215	0.077%
FOKN1_1696	carbamoyl-phosphate synthase, small subunit	N	no hit	K01956	0.027%
FOKN1_1697	carbamoyl-phosphate synthase large subunit	N	no hit	K01955	0.125%
FOKN1_1698	transcription elongation factor GreA	N	no hit	K03624	n.d
FOKN1_1699	uncharacterized protein	N	no hit		n.d
FOKN1_1700	uncharacterized protein	N	no hit	K07574	n.d
FOKN1_1701	ribosomal RNA methyltransferase RrmJ/FtsJ	N	no hit	K02427	n.d

Table S1. Gene annotation of gene-coding sequences (CDSs) and protein expression in the *Thiohalobacter sp.* strain FOKN1 cells. SignalP; prediction of signal peptide sequence, Y; positive, N; negative, COG; clusters of orthologous group, KO; KEGG orthology annotated using the KAAS, PCI; protein content index, n.d; not detected.

Locus_tag	Product	SignalP	COG	KO	PCI (%)
FOKN1_1702	ATP-dependent metalloprotease FtsH	N	no hit	K03798	0.080%
FOKN1_1703	dihydropteroate synthase	N	no hit	K00796	n.d
FOKN1_1704	phosphoglucosamine mutase	N	no hit	K03431	n.d
FOKN1_1705	triose-phosphate isomerase	N	no hit	K01803	0.073%
FOKN1_1706	uncharacterized protein	N	no hit		n.d
FOKN1_1708	NADH dehydrogenase I subunit A	N	C	K00330	n.d
FOKN1_1709	NADH dehydrogenase subunit B	N	C	K00331	0.067%
FOKN1_1710	NADH dehydrogenase subunit C	N	no hit	K00332	0.023%
FOKN1_1711	NADH dehydrogenase subunit D	N	no hit	K00333	0.037%
FOKN1_1712	NADH-ubiquinone oxidoreductase subunit E	N	no hit	K00334	n.d
FOKN1_1713	NADH:ubiquinone oxidoreductase subunit F	N	no hit	K00335	0.017%
FOKN1_1714	NADH-quinone oxidoreductase subunit G	N	no hit	K00336	0.021%
FOKN1_1715	NADH:ubiquinone oxidoreductase, subunit H	N	no hit	K00337	n.d
FOKN1_1716	NADH:ubiquinone oxidoreductase, subunit I	N	no hit	K00338	0.024%
FOKN1_1717	NADH:ubiquinone oxidoreductase, subunit J	N	no hit	K00339	n.d
FOKN1_1718	NADH:ubiquinone oxidoreductase, subunit K	N	no hit	K00340	n.d
FOKN1_1719	NADH:ubiquinone oxidoreductase, subunit L	N	no hit	K00341	n.d
FOKN1_1720	NADH:ubiquinone oxidoreductase, subunit M	N	no hit	K00342	n.d
FOKN1_1721	NADH:ubiquinone oxidoreductase, subunit N	N	no hit	K00343	n.d
FOKN1_1722	uncharacterized protein	N	no hit		n.d
FOKN1_1724	translation elongation factors	N	no hit		n.d
FOKN1_1725	ABC-type transporter, auxiliary component	N	no hit	K07323	0.060%
FOKN1_1726	phosphoketolase	N	G	K01621	n.d
FOKN1_1727	acetate kinase	N	G	K00925	n.d
FOKN1_1728	cation transport ATPase	N	G		n.d
FOKN1_1729	uncharacterized protein	N	G		n.d
FOKN1_1730	adenylate kinase family protein	N	F	K00939	0.237%
FOKN1_1731	surface antigen	Y	no hit	K07278	n.d
FOKN1_1732	uncharacterized protein	N	no hit	K09800	0.003%
FOKN1_1733	precorrin-6B methylase 2	Y	no hit		n.d
FOKN1_1734	lipoprotein	Y	no hit		0.121%
FOKN1_1735	Na+/melibiose symporter	N	M		n.d
FOKN1_1736	cyclopropane-fatty-acyl-phospholipid synthase	N	M	K00574	n.d
FOKN1_1737	uncharacterized protein	N	R	K09701	n.d

Table S1. Gene annotation of gene-coding sequences (CDSs) and protein expression in the *Thiohalobacter sp.* strain FOKN1 cells. SignalP; prediction of signal peptide sequence, Y; positive, N; negative, COG; clusters of orthologous group, KO; KEGG orthology annotated using the KAAS, PCI; protein content index, n.d; not detected.

Locus_tag	Product	SignalP	COG	KO	PCI (%)
FOKN1_1738	amine oxidase	N	R	K06954	n.d
FOKN1_1739	putative transcriptional regulator	N	no hit	K22491	0.033%
FOKN1_1740	dehydrogenase	N	no hit		0.016%
FOKN1_1741	uncharacterized protein	N	no hit		0.100%
FOKN1_1742	uncharacterized protein	N	K		n.d
FOKN1_1743	di-/tricarboxylate transporter	N	K		n.d
FOKN1_1744	transcriptional regulator	N	K		n.d
FOKN1_1745	efflux transporter	N	K	K03585	0.079%
FOKN1_1746	cation/multidrug efflux pump	N	no hit	K18138	n.d
FOKN1_1747	transcriptional regulator	N	no hit		0.141%
FOKN1_1748	ribosome maturation factor RimP	N	no hit	K09748	n.d
FOKN1_1749	transcription elongation factor NusA	N	no hit	K02600	0.066%
FOKN1_1750	translation initiation factor IF-2	N	no hit	K02519	0.122%
FOKN1_1751	ribosome-binding factor A	N	no hit	K02834	n.d
FOKN1_1752	tRNA pseudouridine synthase B	N	no hit	K03177	n.d
FOKN1_1753	30S ribosomal protein S15	N	no hit	K02956	n.d
FOKN1_1754	polynucleotide phosphorylase/polyadenylase	N	no hit	K00962	0.245%
FOKN1_1755	amino acid transporters	N	no hit		n.d
FOKN1_1756	uncharacterized protein	Y	no hit		n.d
FOKN1_1757	response regulator	N	no hit		0.045%
FOKN1_1758	acyl-coenzyme A synthetases/AMP-fatty acid ligases	N	no hit	K01895	0.203%
FOKN1_1759	uncharacterized protein	Y	no hit	K09004	n.d
FOKN1_1760	glutamyl- and glutaminyl-tRNA synthetases	N	no hit	K01894	0.012%
FOKN1_1761	RNA polymerase-binding transcription factor DksA	N	no hit	K06204	0.053%
FOKN1_1762	phosphatase/phosphohexomutase	N	no hit		n.d
FOKN1_1763	aspartate/tyrosine/aromatic aminotransferase	N	no hit		n.d
FOKN1_1764	uncharacterized protein	N	no hit	K06203	n.d
FOKN1_1765	putative carbohydrate kinase	N	no hit	K22935	n.d
FOKN1_1766	7-cyano-7-deazaguanine reductase	N	no hit	K09457	0.054%
FOKN1_1767	ATPase	N	no hit	K03529	0.037%
FOKN1_1768	cell division protein ZipA	N	no hit	K03528	n.d
FOKN1_1769	NAD-dependent DNA ligase	N	no hit	K01972	0.018%
FOKN1_1770	thymidine phosphorylase	N	no hit		0.004%
FOKN1_1771	type IV pilus assembly PilZ	N	no hit		n.d

Table S1. Gene annotation of gene-coding sequences (CDSs) and protein expression in the *Thiohalobacter sp.* strain FOKN1 cells. SignalP; prediction of signal peptide sequence, Y; positive, N; negative, COG; clusters of orthologous group, KO; KEGG orthology annotated using the KAAS, PCI; protein content index, n.d; not detected.

Locus_tag	Product	SignalP	COG	KO	PCI (%)
FOKN1_1772	uncharacterized protein	Y	no hit	K16291	n.d
FOKN1_1773	murein lipoprotein	Y	no hit		0.692%
FOKN1_1774	uncharacterized protein	Y	no hit		n.d
FOKN1_1775	FAD dependent oxidoreductase	N	no hit	K00116	n.d
FOKN1_1776	glycolate oxidase iron-sulfur subunit	N	no hit	K11473	n.d
FOKN1_1777	glycolate oxidase subunit GlcE	N	no hit	K11472	n.d
FOKN1_1778	glycolate oxidase subunit GlcD	N	R	K00104	n.d
FOKN1_1779	thiopurine S-methyltransferase	N	R	K00569	n.d
FOKN1_1780	uncharacterized protein	Y	R		n.d
FOKN1_1781	myosin	N	no hit		0.007%
FOKN1_1782	membrane carboxypeptidase	N	no hit	K05365	n.d
FOKN1_1783	uncharacterized protein	Y	no hit		n.d
FOKN1_1784	phosphoribosylformylglycinamidine synthase	N	no hit		n.d
FOKN1_1785	Rad3-related DNA helicases	N	no hit	K03722	n.d
FOKN1_1786	peptidase M22, glycoprotease	N	no hit	K14742	n.d
FOKN1_1787	uncharacterized protein	N	no hit		n.d
FOKN1_1788	biotin synthase	Y	no hit		0.038%
FOKN1_1789	uncharacterized protein	N	T		0.054%
FOKN1_1790	uncharacterized protein	N	S		n.d
FOKN1_1791	peptide methionine sulfoxide reductase	N	no hit		n.d
FOKN1_1792	transcriptional regulator	N	no hit		n.d
FOKN1_1793	uncharacterized protein	N	no hit	K07003	n.d
FOKN1_1794	FAD-dependent pyridine nucleotide-disulfideoxidoreductase	N	KT	K17218	0.087%
FOKN1_1795	putative transcriptional regulator	N	KT		n.d
FOKN1_1796	rhodanese-like protein	Y	no hit		0.109%
FOKN1_1797	uncharacterized protein	Y	no hit		n.d
FOKN1_1798	3-deoxy-D-arabino-heptulosonate 7-phosphate synthase	N	no hit	K03856	0.025%
FOKN1_1799	adenosylmethionine-8-amino-7-oxononanoate aminotransferase	N	no hit	K00833	n.d
FOKN1_1800	nitrilase/cyanide hydratase and apolipoprotein N-acyltransferase	N	no hit	K12251	0.021%
FOKN1_1801	agmatine deiminase	N	no hit	K10536	n.d
FOKN1_1802	uncharacterized protein	Y	no hit		n.d

Table S1. Gene annotation of gene-coding sequences (CDSs) and protein expression in the *Thiohalobacter sp.* strain FOKN1 cells. SignalP; prediction of signal peptide sequence, Y; positive, N; negative, COG; clusters of orthologous group, KO; KEGG orthology annotated using the KAAS, PCI; protein content index, n.d; not detected.

Locus_tag	Product	SignalP	COG	KO	PCI (%)
FOKN1_1803	sodium-type flagellar protein MotY	Y	no hit	K21218	n.d
FOKN1_1804	ABC-type transporter, permease component	N	no hit	K09808	n.d
FOKN1_1805	ABC-type antimicrobial peptide transporter, ATPase component	N	no hit	K09810	0.028%
FOKN1_1806	uncharacterized protein	N	no hit	K09928	n.d
FOKN1_1807	uncharacterized protein	N	no hit	K02238	n.d
FOKN1_1808	biopolymer transport proteins	N	no hit	K03561	0.088%
FOKN1_1809	biopolymer transport protein ExbD/TolR	N	no hit		n.d
FOKN1_1810	ABC-type multidrug transporter, ATPase and permease components	N	no hit	K11085	n.d
FOKN1_1811	tetraacyldisaccharide 4'-kinase	N	M	K00912	n.d
FOKN1_1812	uncharacterized protein	N	no hit	K09791	n.d
FOKN1_1813	3-deoxy-D-manno-octulosonatecytidylyltransferase	N	no hit	K00979	n.d
FOKN1_1814	uncharacterized protein	N	no hit		n.d
FOKN1_1815	permease	N	no hit		n.d
FOKN1_1816	uncharacterized protein	N	no hit		n.d
FOKN1_1817	triphosphoribosyl-dephospho-CoA synthetase	N	no hit		n.d
FOKN1_1818	diguanylate cyclase	N	no hit		n.d
FOKN1_1819	uncharacterized protein	N	S		n.d
FOKN1_1820	methyl-accepting chemotaxis protein	N	no hit		n.d
FOKN1_1821	protein-tyrosine-phosphatase	N	no hit	K01104	n.d
FOKN1_1822	ribonuclease E	N	no hit	K08300	0.076%
FOKN1_1823	pseudouridine synthase	N	J	K06179	n.d
FOKN1_1824	haloacid dehalogenase superfamily enzyme	N	no hit	K01091	n.d
FOKN1_1825	periplasmic serine proteases	N	no hit	K04773	n.d
FOKN1_1826	uncharacterized protein	N	no hit		n.d
FOKN1_1827	nucleotide-binding protein	N	no hit		n.d
FOKN1_1828	pyruvate/oxaloacetate carboxyltransferase	N	no hit	K01960	n.d
FOKN1_1829	cytochrome c peroxidase	Y	no hit		n.d
FOKN1_1830	biotin carboxylase	N	no hit	K01959	n.d
FOKN1_1831	putative metal-binding protein	N	no hit	K07040	n.d
FOKN1_1832	50S ribosomal protein L32	N	no hit	K02911	n.d
FOKN1_1833	glycerol-3-phosphate acyltransferase PlsX	N	no hit	K03621	n.d
FOKN1_1834	3-oxoacyl-ACP synthase	N	I	K00648	0.025%

Table S1. Gene annotation of gene-coding sequences (CDSs) and protein expression in the *Thiohalobacter sp.* strain FOKN1 cells. SignalP; prediction of signal peptide sequence, Y; positive, N; negative, COG; clusters of orthologous group, KO; KEGG orthology annotated using the KAAS, PCI; protein content index, n.d; not detected.

Locus_tag	Product	SignalP	COG	KO	PCI (%)
FOKN1_1835	malonyl CoA-acyl carrier protein transacylase	N	I	K00645	n.d
FOKN1_1836	3-ketoacyl-(acyl-carrier-protein) reductase	N	IQR	K00059	0.060%
FOKN1_1837	acyl carrier protein	N	no hit	K02078	n.d
FOKN1_1838	3-oxoacyl-[acyl-carrier-protein] synthase II	N	no hit	K09458	0.015%
FOKN1_1839	anthranilate synthase component I	N	no hit	K01665	n.d
FOKN1_1840	aminodeoxychorismate lyase apoprotein	N	no hit	K02619	n.d
FOKN1_1841	aminodeoxychorismate lyase	N	no hit	K07082	n.d
FOKN1_1842	uncharacterized protein	Y	no hit		n.d
FOKN1_1843	citrate synthase	N	no hit	K01647	0.047%
FOKN1_1844	aminotransferase	N	P		n.d
FOKN1_1845	cytochrome c5	Y	no hit		0.391%
FOKN1_1846	pseudouridine synthase	N	J	K06181	n.d
FOKN1_1847	thymidylate kinase	N	no hit	K00943	0.021%
FOKN1_1848	aminodeoxychorismate lyase	N	no hit	K02341	n.d
FOKN1_1849	type IV pilus assembly PilZ	N	no hit	K02676	n.d
FOKN1_1850	Mg-dependent DNase	N	no hit	K03424	0.014%
FOKN1_1851	L-aminopeptidase/D-esterase	N	no hit		n.d
FOKN1_1852	acyl-CoA hydrolase	N	no hit	K10806	n.d
FOKN1_1853	long-chain acyl-CoA synthetases	N	I	K01897	0.014%
FOKN1_1854	3-hydroxyacyl-CoA dehydrogenase	N	I	K01782	0.011%
FOKN1_1855	acetyl-CoA acetyltransferase	N	I	K00626	n.d
FOKN1_1856	acyl-CoA dehydrogenases	N	no hit	K06445	0.007%
FOKN1_1857	uncharacterized protein	N	no hit		n.d
FOKN1_1858	selenocysteine lyase	N	no hit		0.022%
FOKN1_1859	alkyl hydroperoxide reductase	N	O		n.d
FOKN1_1860	alkyl hydroperoxide reductase	N	no hit	K03386	0.050%
FOKN1_1861	L-seryl-tRNA selenium transferase	N	no hit	K01042	n.d
FOKN1_1862	selenocysteine-specific translation elongation factor	N	no hit	K03833	0.013%
FOKN1_1864	selenophosphate synthetase	N	no hit	K01008	n.d
FOKN1_1865	excinuclease ABC, C subunit-like protein	N	no hit	K07461	n.d
FOKN1_1866	uncharacterized protein	N	no hit		n.d
FOKN1_1867	Na+-transporting NADH:ubiquinone oxidoreductase, subunit NqrA	N	C		n.d
FOKN1_1868	phosphatase	N	IQR		n.d

Table S1. Gene annotation of gene-coding sequences (CDSs) and protein expression in the *Thiohalobacter sp.* strain FOKN1 cells. SignalP; prediction of signal peptide sequence, Y; positive, N; negative, COG; clusters of orthologous group, KO; KEGG orthology annotated using the KAAS, PCI; protein content index, n.d; not detected.

Locus_tag	Product	SignalP	COG	KO	PCI (%)
FOKN1_1869	dehydrogenases	N	IQR	K00001	n.d
FOKN1_1870	cytochrome B561	N	IQR		n.d
FOKN1_1871	uncharacterized protein	Y	no hit		0.019%
FOKN1_1872	uncharacterized protein	N	no hit		n.d
FOKN1_1873	alpha-L-glutamate ligase	N	no hit	K05844	n.d
FOKN1_1874	succinylglutamate desuccinylase/aspartoacylase	N	no hit	K06987	n.d
FOKN1_1875	serine/threonine protein kinase	N	no hit		n.d
FOKN1_1876	serine/threonine protein phosphatase	N	P		n.d
FOKN1_1877	sulfate adenylyltransferase	N	P	K00958	0.273%
FOKN1_1878	SAM-dependent methyltransferases	N	no hit		n.d
FOKN1_1879	uncharacterized protein	N	no hit		n.d
FOKN1_1880	pyruvate/2-oxoglutarate dehydrogenase complex, dihydrolipoamide dehydrogenase (E3) component	N	no hit	K00382	0.021%
FOKN1_1881	pyruvate/2-oxoglutarate dehydrogenase complex, dihydrolipoamide acyltransferase (E2) component	N	no hit	K00658	n.d
FOKN1_1882	alpha-ketoglutarate decarboxylase	N	no hit	K00164	0.006%
FOKN1_1883	mechanosensitive ion channel protein MscS	N	M	K22044	n.d
FOKN1_1884	6-phosphofructokinase	N	M		n.d
FOKN1_1885	histone acetyltransferase	N	O		n.d
FOKN1_1886	quinolinate synthase A	N	O	K03517	n.d
FOKN1_1887	putative protein-S-isoprenylcysteine methyltransferase	N	O	K21310	n.d
FOKN1_1888	membrane protease	N	O		0.025%
FOKN1_1889	serine protease	Y	no hit	K07403	n.d
FOKN1_1890	flavodoxin reductases	N	no hit	K11933	n.d
FOKN1_1891	fructose-1,6-bisphosphate aldolase	N	no hit	K01623	n.d
FOKN1_1892	uncharacterized protein	N	no hit		n.d
FOKN1_1896	CDP-diacylglycerol-glycerol-3-phosphate 3-phosphatidyltransferase	N	no hit	K00995	0.019%
FOKN1_1897	excinuclease ABC subunit C	N	no hit	K03703	n.d
FOKN1_1898	uracil-DNA glycosylase	N	no hit	K21929	n.d
FOKN1_1899	response regulator	N	no hit	K07689	0.018%
FOKN1_1900	uncharacterized protein	Y	no hit		n.d
FOKN1_1901	beta-mannanase	Y	no hit		n.d
FOKN1_1902	ABC-type dipeptide/oligopeptide/nickel transporter,	Y	no hit		n.d

Table S1. Gene annotation of gene-coding sequences (CDSs) and protein expression in the *Thiohalobacter sp.* strain FOKN1 cells. SignalP; prediction of signal peptide sequence, Y; positive, N; negative, COG; clusters of orthologous group, KO; KEGG orthology annotated using the KAAS, PCI; protein content index, n.d; not detected.

Locus_tag	Product	SignalP	COG	KO	PCI (%)
	permease components				
FOKN1_1903	double-glycine peptidase	Y	no hit	K06992	n.d
FOKN1_1904	bifunctional proline dehydrogenase/pyrroline-5-carboxylate dehydrogenase	Y	O		n.d
FOKN1_1905	uncharacterized protein	Y	no hit		n.d
FOKN1_1906	response regulator	N	no hit		n.d
FOKN1_1907	transcriptional regulators	N	no hit		n.d
FOKN1_1908	integrase	N	no hit		n.d
FOKN1_1909	metal-dependent Rnase	N	no hit		n.d
FOKN1_1910	uncharacterized protein	N	R		n.d
FOKN1_1911	uncharacterized protein	N	R		n.d
FOKN1_1912	ATPase	N	R	K06919	n.d
FOKN1_1913	ATPase	N	L		n.d
FOKN1_1914	coenzyme F420-dependent N5, N10-methylene tetrahydromethanopterin reductase	N	E		n.d
FOKN1_1915	tRNA-dihydrouridine synthase	N	J		n.d
FOKN1_1916	capsid-like protein	N	no hit		0.009%
FOKN1_1917	Zn-dependent peptidases	N	R		n.d
FOKN1_1918	uncharacterized protein	N	R		n.d
FOKN1_1919	uncharacterized protein	N	no hit		n.d
FOKN1_1922	integral membrane protein, interacts with FtsH	N	no hit	K19416	n.d
FOKN1_1923	glucose dehydrogenase	N	no hit		n.d
FOKN1_1924	diadenosine tetraphosphatase and related serine /threonine protein phosphatases	N	T		n.d
FOKN1_1925	exonuclease VII small subunit	N	no hit		n.d
FOKN1_1926	uncharacterized protein	N	R	K07126	n.d
FOKN1_1927	uncharacterized protein	N	R		n.d
FOKN1_1928	protein kinase	N	R		n.d
FOKN1_1929	subtilisin-like serine proteases	N	O	K01342	n.d
FOKN1_1930	ornithine/acetylornithine aminotransferase	Y	O		n.d
FOKN1_1931	uncharacterized protein	N	no hit		0.051%
FOKN1_1932	uncharacterized protein	N	no hit		n.d
FOKN1_1933	2OG-Fe(II) oxygenase	N	no hit		n.d
FOKN1_1934	galactoside O-acetyltransferase	N	no hit		n.d

Table S1. Gene annotation of gene-coding sequences (CDSs) and protein expression in the *Thiohalobacter sp.* strain FOKN1 cells. SignalP; prediction of signal peptide sequence, Y; positive, N; negative, COG; clusters of orthologous group, KO; KEGG orthology annotated using the KAAS, PCI; protein content index, n.d; not detected.

Locus_tag	Product	SignalP	COG	KO	PCI (%)
FOKN1_1935	permeases	N	no hit		n.d
FOKN1_1936	cyclic nucleotide-binding protein	N	T		n.d
FOKN1_1937	glutamate dehydrogenase/leucine dehydrogenase	N	S		n.d
FOKN1_1938	pyruvate:ferredoxin oxidoreductase and related 2-oxoacid:ferredoxin oxidoreductases, beta subunit	N	C		n.d
FOKN1_1939	oxidoreductase, 2OG-Fe(II) oxygenase family	N	S	K07336	n.d
FOKN1_1940	uncharacterized protein	N	no hit		n.d
FOKN1_1941	dissimilatory sulfite reductase (desulfoviridin), gamma subunit	N	no hit		0.033%
FOKN1_1942	Fe2+/Pb2+ permease	N	P		0.015%
FOKN1_1943	cation/multidrug efflux pump	N	P		n.d
FOKN1_1944	taurine catabolism dioxygenase	N	C		n.d
FOKN1_1945	sulfite reductase alpha subunit	N	C	K11180	0.343%
FOKN1_1946	sulfite reductase beta subunit	N	C	K11181	0.380%
FOKN1_1947	tRNA 5-methylaminomethyl-2-thiouridine synthase TusD	N	no hit	K07235	0.846%
FOKN1_1948	sulfur relay protein TusC/DsrF	N	no hit	K07236	0.064%
FOKN1_1949	sulfur relay protein TusB/DsrH	N	no hit		0.066%
FOKN1_1950	sulfite reductase, dissimilatory-type subunit gamma	N	no hit	K11179	0.153%
FOKN1_1951	nitrate reductase subunit gamma	N	C	K18500	0.050%
FOKN1_1952	Fe-S oxidoreductase	N	C	K18501	0.066%
FOKN1_1953	NADPH-dependent glutamate synthase beta chain	N	no hit		0.096%
FOKN1_1954	sulfur oxidation protein DsrJ	Y	C		n.d
FOKN1_1955	4Fe-4S ferredoxin	N	C	K00184	0.087%
FOKN1_1956	polysulfide reductase NrfD	N	C	K00185	n.d
FOKN1_1957	sulfur oxidation protein DsrP	N	H		n.d
FOKN1_1958	cobyrinic acid a,c-diamide synthase	N	H	K02224	n.d
FOKN1_1959	sulfur oxidation protein DsrR	N	S		n.d
FOKN1_1960	signal transduction histidine kinase	N	no hit	K07673	0.032%
FOKN1_1961	transcriptional regulator	N	G	K07684	n.d
FOKN1_1962	rhodanese-related sulfurtransferase	N	G		n.d
FOKN1_1963	alpha amylase catalytic subunit	N	G	K05343	0.008%
FOKN1_1964	FAD-dependent pyridine nucleotide-disulfideoxidoreductase	N	no hit	K03885	n.d
FOKN1_1965	uncharacterized protein	Y	no hit		n.d

Table S1. Gene annotation of gene-coding sequences (CDSs) and protein expression in the *Thiohalobacter sp.* strain FOKN1 cells. SignalP; prediction of signal peptide sequence, Y; positive, N; negative, COG; clusters of orthologous group, KO; KEGG orthology annotated using the KAAS, PCI; protein content index, n.d; not detected.

Locus_tag	Product	SignalP	COG	KO	PCI (%)
FOKN1_1966	peptidase S24/S26A/S26B	N	no hit		0.033%
FOKN1_1967	uroporphyrinogen-III C-methyltransferase /precorrin-2 dehydrogenase	N	no hit	K02302	0.051%
FOKN1_1968	uncharacterized protein	Y	no hit		n.d
FOKN1_1969	aminopeptidase N	N	no hit	K01256	0.022%
FOKN1_1970	dissimilatory sulfite reductase (desulfoviridin), gamma subunit	N	no hit		n.d
FOKN1_1971	ABC-type oligopeptide transporter, ATPase component	N	no hit		n.d
FOKN1_1972	signal transduction histidine kinase	N	no hit		0.015%
FOKN1_1973	putative diguanylate cyclase	N	no hit		n.d
FOKN1_1974	phosphohistidine phosphatase SixA	N	no hit	K08296	n.d
FOKN1_1975	seryl-tRNA synthetase	N	no hit	K01875	0.027%
FOKN1_1976	uncharacterized protein	N	S		n.d
FOKN1_1977	integral membrane protein	N	no hit		n.d
FOKN1_1978	recombination factor protein RarA	N	no hit	K07478	n.d
FOKN1_1979	outer-membrane lipoprotein carrier protein	Y	no hit	K03634	n.d
FOKN1_1980	DNA translocase FtsK	N	no hit	K03466	n.d
FOKN1_1981	alanine dehydrogenase	N	S	K00259	n.d
FOKN1_1982	thioredoxin reductase	N	S	K00384	n.d
FOKN1_1983	uncharacterized protein	N	S	K09919	n.d
FOKN1_1984	leucyl/phenylalanyl-tRNA-protein transferase	N	no hit	K00684	n.d
FOKN1_1985	putative arginyl-tRNA-protein transferase	N	no hit	K21420	n.d
FOKN1_1986	translation initiation factor IF-1	N	no hit	K02518	n.d
FOKN1_1987	ATPase and specificity subunit of ClpA-ClpPATP-dependent serine protease	N	no hit	K03694	0.044%
FOKN1_1988	ATP-dependent Clp protease adaptor protein ClpS	N	S	K06891	n.d
FOKN1_1989	cold-shock DNA-binding domain-containing protein	N	no hit	K03704	n.d
FOKN1_1990	NADP-dependent isocitrate dehydrogenase	N	no hit	K00031	0.052%
FOKN1_1991	ATP-dependent DNA ligase	Y	no hit		0.104%
FOKN1_1992	NTP pyrophosphohydrolases	N	no hit	K12152	0.038%
FOKN1_1993	tRNA (5-methylaminomethyl-2-thiouridylate)-methyltransferase	N	no hit	K00566	n.d
FOKN1_1994	uncharacterized protein	N	no hit	K07153	n.d
FOKN1_1995	putative diguanylate cyclase	N	no hit	K21085	n.d

Table S1. Gene annotation of gene-coding sequences (CDSs) and protein expression in the *Thiohalobacter sp.* strain FOKN1 cells. SignalP; prediction of signal peptide sequence, Y; positive, N; negative, COG; clusters of orthologous group, KO; KEGG orthology annotated using the KAAS, PCI; protein content index, n.d; not detected.

Locus_tag	Product	SignalP	COG	KO	PCI (%)
FOKN1_1996	aconitate hydratase 1	N	no hit	K01681	0.026%
FOKN1_1997	adenylosuccinate lyase	N	no hit	K01756	0.029%
FOKN1_1998	GCN5-related N-acetyltransferase	N	no hit		n.d
FOKN1_1999	uncharacterized protein	N	R		n.d
FOKN1_2000	S-formylglutathione hydrolase	N	R	K01070	n.d
FOKN1_2001	S-(hydroxymethyl) glutathione dehydrogenase/classIII alcohol dehydrogenase	N	C	K00121	n.d
FOKN1_2002	permeases of the drug/metabolite transporter	N	C		n.d
FOKN1_2003	cytochrome B561	N	C		n.d
FOKN1_2004	uncharacterized protein	N	C		n.d
FOKN1_2005	putative membrane protein	N	no hit		n.d
FOKN1_2006	uncharacterized protein	N	no hit		n.d
FOKN1_2007	permeases of the drug/metabolite transporter	N	no hit		n.d
FOKN1_2008	nickel-dependent hydrogenases b-type cytochrome subunit	N	no hit		n.d
FOKN1_2009	HAD-superfamily hydrolase	N	no hit	K22223	n.d
FOKN1_2010	Mg/Co/Ni transporter MgtE	N	no hit	K06213	n.d
FOKN1_2011	universal stress protein uspA	N	no hit		n.d
FOKN1_2012	universal stress protein uspA	N	no hit		0.082%
FOKN1_2013	universal stress protein uspA	N	no hit	K14055	0.036%
FOKN1_2014	uncharacterized protein	Y	no hit		0.014%
FOKN1_2015	asparagine synthetase A	N	no hit		n.d
FOKN1_2016	GTPases	N	no hit		n.d
FOKN1_2017	archaeal/vacuolar-type H+-ATPase subunit I	N	no hit	K02123	n.d
FOKN1_2018	archaeal/vacuolar-type H+-ATPase, subunit K	N	no hit		n.d
FOKN1_2019	archaeal/vacuolar-type H+-ATPase subunit F	N	no hit		n.d
FOKN1_2020	archaeal/vacuolar-type H+-ATPase subunit E	N	no hit		n.d
FOKN1_2021	archaeal/vacuolar-type H+-ATPase subunit A	N	C	K02117	n.d
FOKN1_2022	archaeal/vacuolar-type H+-ATPase subunit B	N	C	K02118	0.016%
FOKN1_2023	archaeal/vacuolar-type H+-ATPase subunit D	N	C		n.d
FOKN1_2024	uncharacterized protein	N	no hit		n.d
FOKN1_2025	aspartyl/asparaginyl beta-hydroxylase	N	no hit		n.d
FOKN1_2026	diadenosine tetraphosphate hydrolase	N	no hit		n.d
FOKN1_2027	Na+/H+ and K+/H+ antiporters	N	no hit		n.d

Table S1. Gene annotation of gene-coding sequences (CDSs) and protein expression in the *Thiohalobacter sp.* strain FOKN1 cells. SignalP; prediction of signal peptide sequence, Y; positive, N; negative, COG; clusters of orthologous group, KO; KEGG orthology annotated using the KAAS, PCI; protein content index, n.d; not detected.

Locus_tag	Product	SignalP	COG	KO	PCI (%)
FOKN1_2028	uncharacterized protein	Y	no hit		0.258%
FOKN1_2029	putative peptidase	Y	no hit		n.d
FOKN1_2030	peptidoglycan-binding protein	Y	no hit	K21470	n.d
FOKN1_2031	single-stranded-DNA-specific exonuclease RecJ	N	no hit	K07462	n.d
FOKN1_2032	uncharacterized protein	N	no hit		n.d
FOKN1_2033	threonine synthase	N	no hit	K01733	0.069%
FOKN1_2034	homoserine dehydrogenase	N	no hit	K00003	0.103%
FOKN1_2035	aspartate/tyrosine/aromatic aminotransferase	N	no hit	K14261	n.d
FOKN1_2036	uncharacterized protein	N	no hit		n.d
FOKN1_2037	DNA-directed RNA polymerase, sigma subunit	N	no hit	K03087	n.d
FOKN1_2038	lipoprotein NlpD	N	no hit	K06194	n.d
FOKN1_2039	membrane protein	N	no hit		n.d
FOKN1_2040	protein-L-isoaspartate O-methyltransferase	N	no hit	K00573	n.d
FOKN1_2041	acid phosphatase	N	no hit	K03787	n.d
FOKN1_2042	uncharacterized protein	N	no hit		n.d
FOKN1_2043	tRNA pseudouridine synthase D	N	S	K06176	n.d
FOKN1_2044	uncharacterized protein	N	S		n.d
FOKN1_2045	taurine catabolism dioxygenase	N	no hit		n.d
FOKN1_2046	uncharacterized protein	N	no hit		n.d
FOKN1_2047	permeases	N	no hit		n.d
FOKN1_2048	uncharacterized protein	N	no hit		n.d
FOKN1_2049	type I site-specific restriction-modification system, R (restriction) subunit	N	no hit	K01153	n.d
FOKN1_2050	transposase	N	L	K07491	n.d
FOKN1_2051	restriction endonuclease S subunits	N	no hit		n.d
FOKN1_2052	type I restriction-modification system methyltransferase subunit	N	V		n.d
FOKN1_2053	2-C-methyl-D-erythritol 2,4-cyclodiphosphate synthase	N	V	K01770	n.d
FOKN1_2054	4-diphosphocytidyl-2-methyl-D-erithritol synthase	N	V	K00991	n.d
FOKN1_2055	septum formation initiator	N	no hit	K05589	n.d
FOKN1_2056	enolase	N	no hit	K01689	0.098%
FOKN1_2057	3-deoxy-D-manno-octulosonate 8-phosphate synthase	N	no hit	K01627	0.114%
FOKN1_2058	CTP synthetase	N	no hit	K01937	0.068%
FOKN1_2059	putative tRNA(Ile)-lysidine synthase	N	no hit	K04075	n.d

Table S1. Gene annotation of gene-coding sequences (CDSs) and protein expression in the *Thiohalobacter sp.* strain FOKN1 cells. SignalP; prediction of signal peptide sequence, Y; positive, N; negative, COG; clusters of orthologous group, KO; KEGG orthology annotated using the KAAS, PCI; protein content index, n.d; not detected.

Locus_tag	Product	SignalP	COG	KO	PCI (%)
FOKN1_2060	acetyl-CoA carboxylase carboxyltransferase subunit alpha	N	no hit	K01962	0.073%
FOKN1_2061	DNA polymerase III subunit alpha	N	no hit	K02337	0.014%
FOKN1_2062	ABC transporter membrane protein	N	no hit	K02004	n.d
FOKN1_2063	ABC transporter-like protein	N	M	K02003	n.d
FOKN1_2064	RND family efflux pump membrane fusion protein	N	M	K02005	0.009%
FOKN1_2065	RND family efflux pump outer membrane lipoprotein	N	no hit		0.017%
FOKN1_2066	ribonuclease H	N	no hit	K03470	n.d
FOKN1_2067	lipid-A-disaccharide synthase	N	no hit	K00748	n.d
FOKN1_2068	acyl-(acyl-carrier-protein)-UDP-N-acetylglucosamine O-acyltransferase	N	no hit	K00677	n.d
FOKN1_2069	beta-hydroxyacyl-(ACP) dehydratase FabZ	N	no hit	K02372	0.099%
FOKN1_2070	UDP-3-O-(3-hydroxymyristoyl)-glucosamine N-acyltransferase	N	no hit	K02536	n.d
FOKN1_2071	outer membrane protein OmpH	Y	no hit	K06142	0.108%
FOKN1_2072	outer membrane protein assembly factor BamA	Y	no hit	K07277	0.020%
FOKN1_2073	membrane-associated zinc metalloprotease	N	no hit	K11749	n.d
FOKN1_2074	1-deoxy-d-xylulose 5-phosphate reductoisomerase (dxpreductoisomerase) (1-deoxyxylulose-5-phosphatereductoisomerase) (2-c-methyl-d-erythritol 4-phosphate synthase) protein	N	no hit	K00099	n.d
FOKN1_2075	CDP-diglyceride synthetase	N	no hit	K00981	n.d
FOKN1_2076	undecaprenyl pyrophosphate synthase	N	no hit	K00806	n.d
FOKN1_2077	ribosome recycling factor	N	no hit	K02838	0.052%
FOKN1_2078	uridylate kinase	N	no hit	K09903	0.069%
FOKN1_2079	translation elongation factor Ts	N	no hit	K02357	0.031%
FOKN1_2080	ribosomal protein S2	N	no hit	K02967	0.433%
FOKN1_2081	methionine aminopeptidase	N	no hit	K01265	0.037%
FOKN1_2082	bifunctional uridylyltransferase	N	no hit	K00990	n.d
FOKN1_2083	dissimilatory sulfite reductase (desulfoviridin), gamma subunit	N	no hit		0.036%
FOKN1_2084	glutathione S-transferase family protein	N	no hit		n.d
FOKN1_2085	glyoxalase/bleomycin resistance protein/dioxygenase	N	no hit	K08234	n.d
FOKN1_2086	permeases	N	no hit		n.d
FOKN1_2087	aspartate/tyrosine/aromatic aminotransferase	N	no hit	K14267	0.019%

Table S1. Gene annotation of gene-coding sequences (CDSs) and protein expression in the *Thiohalobacter sp.* strain FOKN1 cells. SignalP; prediction of signal peptide sequence, Y; positive, N; negative, COG; clusters of orthologous group, KO; KEGG orthology annotated using the KAAS, PCI; protein content index, n.d; not detected.

Locus_tag	Product	SignalP	COG	KO	PCI (%)
FOKN1_2088	tetrahydrodipicolinate N-succinyltransferase	N	no hit	K00674	0.179%
FOKN1_2089	NADH:ubiquinone oxidoreductase subunit 5	N	no hit	K05559	n.d
FOKN1_2090	multisubunit Na+/H+ antiporter, MnhC subunit	N	no hit	K05560	n.d
FOKN1_2091	multisubunit Na+/H+ antiporter, MnhD subunit	N	no hit	K05561	n.d
FOKN1_2092	multisubunit Na+/H+ antiporter, MnhE subunit	N	no hit	K05562	n.d
FOKN1_2093	multisubunit Na+/H+ antiporter, MnhF subunit	N	no hit	K05563	n.d
FOKN1_2094	multisubunit Na+/H+ antiporter, MnhG subunit	N	no hit	K05564	n.d
FOKN1_2095	uncharacterized protein	N	no hit		n.d
FOKN1_2096	translational repressor	Y	no hit		0.006%
FOKN1_2097	uncharacterized protein	N	no hit		0.029%
FOKN1_2098	cAMP-binding proteins	N	no hit		n.d
FOKN1_2099	glucose/sorbosone dehydrogenases	Y	no hit	K21430	0.015%
FOKN1_2100	streptogramin lyase	Y	no hit	K18235	0.011%
FOKN1_2101	uncharacterized protein	N	P	K09004	n.d
FOKN1_2102	arsenite efflux pump ACR3 and related permeases	N	P	K03325	n.d
FOKN1_2103	protein tyrosine phosphatase	N	T	K03741	n.d
FOKN1_2104	arsR family transcriptional regulator	N	no hit	K03892	n.d
FOKN1_2105	response regulator receiver	N	no hit		n.d
FOKN1_2106	arsenate reductase	N	no hit		n.d
FOKN1_2107	succinyl-diaminopimelate desuccinylase	N	no hit	K01439	0.010%
FOKN1_2108	integral membrane protein	N	no hit		0.012%
FOKN1_2109	TRAP-type C4-dicarboxylate transporter, periplasmic component	Y	no hit		n.d
FOKN1_2110	TRAP-type C4-dicarboxylate transporter, small permease component	N	no hit		n.d
FOKN1_2111	TRAP-type C4-dicarboxylate transporter, large permease component	N	no hit	K11690	n.d
FOKN1_2112	sirohydrochlorin cobaltochelatase	N	no hit	K03794	0.083%
FOKN1_2113	uncharacterized protein	Y	no hit		n.d
FOKN1_2114	uncharacterized protein	N	no hit	K09160	n.d
FOKN1_2115	SAM-dependent methyltransferases	N	no hit	K15256	n.d
FOKN1_2116	SAM-dependent methyltransferases	N	no hit	K15257	n.d
FOKN1_2117	transcription-repair coupling factor	N	no hit	K03723	0.004%
FOKN1_2118	uncharacterized protein	Y	no hit		0.023%

Table S1. Gene annotation of gene-coding sequences (CDSs) and protein expression in the *Thiohalobacter sp.* strain FOKN1 cells. SignalP; prediction of signal peptide sequence, Y; positive, N; negative, COG; clusters of orthologous group, KO; KEGG orthology annotated using the KAAS, PCI; protein content index, n.d; not detected.

Locus_tag	Product	SignalP	COG	KO	PCI (%)
FOKN1_2119	uncharacterized protein	N	no hit	K19160	n.d
FOKN1_2120	uncharacterized protein	N	no hit	K19161	n.d
FOKN1_2121	DNA/RNA helicases	Y	no hit		n.d
FOKN1_2122	uncharacterized protein	N	no hit		n.d
FOKN1_2123	uncharacterized protein	N	no hit		n.d
FOKN1_2124	amidohydrolase	N	R		n.d
FOKN1_2125	alkyl hydroperoxide reductase	N	R	K03564	0.071%
FOKN1_2126	5'-methylthioadenosine phosphorylase	N	no hit	K00772	0.040%
FOKN1_2127	hypoxanthine-guanine phosphoribosyltransferase	N	no hit	K00760	n.d
FOKN1_2128	esterase	N	no hit	K07001	0.019%
FOKN1_2129	small-conductance mechanosensitive channel	N	no hit	K16052	n.d
FOKN1_2130	beta-N-acetylhexosaminidase	N	no hit	K01207	n.d
FOKN1_2131	ABC-type dipeptide/oligopeptide/nickel transporter, permease components	N	EP	K02034	n.d
FOKN1_2132	ABC-type dipeptide/oligopeptide/nickel transporter, permease components	N	no hit	K02033	n.d
FOKN1_2133	uncharacterized protein	N	no hit		n.d
FOKN1_2134	ABC-type dipeptide transporter, periplasmic component	Y	no hit	K12368	n.d
FOKN1_2135	adenylate cyclase	N	no hit		n.d
FOKN1_2136	uncharacterized protein	N	no hit		n.d
FOKN1_2137	succinate dehydrogenase/fumarate reductase, Fe-S protein subunit	N	no hit	K00240	0.014%
FOKN1_2138	succinate dehydrogenase/fumarate reductase, flavoprotein subunit	N	no hit	K00239	0.019%
FOKN1_2139	succinate dehydrogenase, hydrophobic anchor subunit	N	no hit		n.d
FOKN1_2140	succinate dehydrogenase/fumarate reductase, cytochrome b subunit	N	no hit		n.d
FOKN1_2141	aminomethyl transferase	N	no hit	K06980	0.017%
FOKN1_2142	phosphodiesterase transmembrane protein	N	no hit		0.022%
FOKN1_2143	lysyl-tRNA synthetase	N	no hit	K04567	0.044%
FOKN1_2144	peptide chain release factor	N	J	K02836	n.d
FOKN1_2145	phospho-2-dehydro-3-deoxyheptonate aldolase	N	no hit	K01626	0.028%
FOKN1_2146	surface lipoprotein	N	no hit	K04754	0.018%
FOKN1_2148	transcriptional regulator	N	no hit		n.d

Table S1. Gene annotation of gene-coding sequences (CDSs) and protein expression in the *Thiohalobacter sp.* strain FOKN1 cells. SignalP; prediction of signal peptide sequence, Y; positive, N; negative, COG; clusters of orthologous group, KO; KEGG orthology annotated using the KAAS, PCI; protein content index, n.d; not detected.

Locus_tag	Product	SignalP	COG	KO	PCI (%)
FOKN1_2149	DNA-binding protein	N	no hit	K04764	0.064%
FOKN1_2150	phenylalanine tRNA synthetase, beta-subunit	N	no hit	K01890	0.023%
FOKN1_2151	phenylalanyl-tRNA synthetase, alpha subunit	N	no hit	K01889	0.016%
FOKN1_2152	50S ribosomal protein L20	N	no hit	K02887	0.313%
FOKN1_2153	50S ribosomal protein L35	N	no hit	K02916	n.d
FOKN1_2154	translation initiation factor IF-3	N	no hit	K02520	0.025%
FOKN1_2155	threonyl-tRNA synthetase	N	no hit	K01868	0.069%
FOKN1_2157	cytochrome-c peroxidase	Y	no hit		n.d
FOKN1_2158	metallophosphoesterase	N	no hit		n.d
FOKN1_2159	cytochrome c	Y	no hit		n.d
FOKN1_2160	excinuclease ABC subunit B	N	E	K03702	n.d
FOKN1_2161	aspartate/tyrosine/aromatic aminotransferase	N	E	K00812	0.031%
FOKN1_2163	UTP-glucose-1-phosphate uridylyltransferase	N	no hit	K00963	0.067%
FOKN1_2164	UDP-N-acetylglucosamine 4,6-dehydratase	N	no hit	K19421	n.d
FOKN1_2165	aminotransferase protein	N	no hit		n.d
FOKN1_2166	sugar transferase	N	no hit		n.d
FOKN1_2167	uncharacterized protein	N	no hit		n.d
FOKN1_2168	glycosyltransferase	N	E		n.d
FOKN1_2169	asparagine synthase	N	E	K01953	n.d
FOKN1_2170	uncharacterized protein	N	E		n.d
FOKN1_2171	SAM-dependent methyltransferases	N	QR		0.029%
FOKN1_2172	uncharacterized protein	N	no hit		n.d
FOKN1_2173	mannose-1-phosphate guanylyltransferase	N	no hit	K16011	0.020%
FOKN1_2174	uncharacterized protein	Y	no hit	K02237	n.d
FOKN1_2175	orotidine 5'-phosphate decarboxylase	N	no hit	K01591	0.017%
FOKN1_2176	N-acetylglucosaminyl transferase	N	no hit	K19804	n.d
FOKN1_2177	putative membrane protein	N	S		n.d
FOKN1_2178	DNA-binding protein	N	S	K05788	0.192%
FOKN1_2179	30S ribosomal protein S1	N	no hit	K02945	0.145%
FOKN1_2180	cytidylate kinase	N	no hit	K00945	n.d
FOKN1_2181	5-enolpyruvylshikimate-3-phosphate synthase	N	no hit	K00800	0.044%
FOKN1_2182	prephenate dehydrogenase	N	no hit	K04517	n.d
FOKN1_2183	histidinol-phosphate aminotransferase	N	no hit	K00817	0.021%
FOKN1_2184	prephenate dehydratase	N	no hit	K14170	0.027%

Table S1. Gene annotation of gene-coding sequences (CDSs) and protein expression in the *Thiohalobacter sp.* strain FOKN1 cells. SignalP; prediction of signal peptide sequence, Y; positive, N; negative, COG; clusters of orthologous group, KO; KEGG orthology annotated using the KAAS, PCI; protein content index, n.d; not detected.

Locus_tag	Product	SignalP	COG	KO	PCI (%)
FOKN1_2185	NAD-binding D-isomer specific 2-hydroxyacid dehydrogenase	N	HE	K00058	0.082%
FOKN1_2186	phosphoserine aminotransferase	N	no hit	K00831	0.063%
FOKN1_2187	DNA gyrase subunit A	N	no hit	K02469	0.064%
FOKN1_2188	translation initiation factor	N	no hit	K08963	n.d
FOKN1_2189	transcriptional regulator	N	no hit		0.015%
FOKN1_2190	ompA/MotB domain protein	Y	no hit		0.525%
FOKN1_2191	mechanosensitive ion channel protein MscS	N	no hit	K03442	0.027%
FOKN1_2192	N-ethylammeline chlorohydrolase	N	no hit	K12960	0.029%
FOKN1_2193	3-demethylubiquinone-9 3-methyltransferase	N	no hit	K00568	0.016%
FOKN1_2194	2-phosphoglycolate phosphatase	N	no hit	K22292	n.d
FOKN1_2195	uncharacterized protein	N	S	K09760	0.017%
FOKN1_2196	phytoene/squalene synthetase	N	no hit		n.d
FOKN1_2197	phytoene/squalene synthetase	N	no hit	K02291	n.d
FOKN1_2198	amine oxidase	N	no hit	K06954	n.d
FOKN1_2199	short chain dehydrogenase	N	no hit		0.014%
FOKN1_2200	thioredoxin-related protein	N	O	K03981	0.017%
FOKN1_2201	diguanylate cyclase	N	no hit		n.d
FOKN1_2202	endonuclease III	N	no hit	K07457	n.d
FOKN1_2203	16S rRNA uridine-516 pseudouridylate synthase	N	no hit	K06178	n.d
FOKN1_2204	transcriptional regulator	N	no hit	K06024	n.d
FOKN1_2205	uncharacterized protein	N	no hit	K05896	n.d
FOKN1_2206	tryptophanyl-tRNA synthetase	N	no hit	K01867	0.024%
FOKN1_2207	Zn-dependent proteases	N	no hit		n.d
FOKN1_2208	translation factor	N	no hit		n.d
FOKN1_2209	metal-dependent phosphoesterases	N	no hit	K07053	n.d
FOKN1_2210	intracellular septation protein A	N	no hit	K06190	n.d
FOKN1_2211	uncharacterized protein	N	no hit	K09780	n.d
FOKN1_2212	stress-induced morphogen	N	no hit		n.d
FOKN1_2213	PpiC-type peptidyl-prolyl cis-trans isomerase	Y	no hit	K03769	n.d
FOKN1_2214	ATPase	N	no hit	K04562	n.d
FOKN1_2215	thioredoxin	N	no hit		n.d
FOKN1_2216	uncharacterized protein	N	no hit		n.d
FOKN1_2217	Mg-dependent DNase	N	no hit	K03424	n.d

Table S1. Gene annotation of gene-coding sequences (CDSs) and protein expression in the *Thiohalobacter sp.* strain FOKN1 cells. SignalP; prediction of signal peptide sequence, Y; positive, N; negative, COG; clusters of orthologous group, KO; KEGG orthology annotated using the KAAS, PCI; protein content index, n.d; not detected.

Locus_tag	Product	SignalP	COG	KO	PCI (%)
FOKN1_2218	uncharacterized protein	N	no hit	K09902	n.d
FOKN1_2219	permeases	N	R	K07090	n.d
FOKN1_2220	uncharacterized protein	N	R		n.d
FOKN1_2221	universal stress protein	N	no hit		0.120%
FOKN1_2222	response regulator	N	no hit	K07713	n.d
FOKN1_2223	signal transduction histidine kinase	N	no hit	K02482	n.d
FOKN1_2224	NAD-dependent aldehyde dehydrogenases	N	no hit		n.d
FOKN1_2225	uncharacterized protein	N	no hit		n.d
FOKN1_2226	uncharacterized protein	N	no hit		n.d
FOKN1_2227	transcriptional regulator	N	S		n.d
FOKN1_2228	cytotoxic translational repressor	N	S		n.d
FOKN1_2229	transcriptional regulator	N	S		n.d
FOKN1_2230	uncharacterized protein	N	S		n.d
FOKN1_2231	glucose dehydrogenase	N	L		n.d
FOKN1_2232	nuclease	N	L		n.d
FOKN1_2233	retron-type reverse transcriptase	N	L		0.006%
FOKN1_2234	restriction endonuclease S subunits	N	no hit	K01154	n.d
FOKN1_2235	type I restriction-modification system methyltransferase subunit	N	no hit	K03427	0.014%
FOKN1_2236	type I site-specific restriction-modification system, R subunit	N	V	K01153	n.d
FOKN1_2237	transcriptional regulator	N	V		n.d
FOKN1_2238	uncharacterized protein	N	no hit		0.008%
FOKN1_2239	ABC-type dipeptide transporter, periplasmic component	N	E		n.d
FOKN1_2240	drug exporters	N	K		n.d
FOKN1_2241	uncharacterized protein	N	K		n.d
FOKN1_2242	transcriptional regulator	N	K		n.d
FOKN1_2243	transposase	N	K	K07497	n.d
FOKN1_2244	transposase	N	no hit	K07497	n.d
FOKN1_2245	site-specific recombinases	N	no hit		n.d
FOKN1_2247	cytochrome bd-type quinol oxidase, subunit 2	N	no hit		n.d
FOKN1_2249	ATP-dependent peptidase	N	no hit		0.026%
FOKN1_2250	protein-L-isoaspartate O-methyltransferase	N	no hit	K00573	n.d
FOKN1_2251	uncharacterized protein	N	no hit		n.d

Table S1. Gene annotation of gene-coding sequences (CDSs) and protein expression in the *Thiohalobacter sp.* strain FOKN1 cells. SignalP; prediction of signal peptide sequence, Y; positive, N; negative, COG; clusters of orthologous group, KO; KEGG orthology annotated using the KAAS, PCI; protein content index, n.d; not detected.

Locus_tag	Product	SignalP	COG	KO	PCI (%)
FOKN1_2252	replication factor C subunit	N	L	K14415	n.d
FOKN1_2253	uncharacterized protein	N	L	K02347	n.d
FOKN1_2254	fructose/tagatose bisphosphate aldolase	N	G	K01624	0.044%
FOKN1_2255	uncharacterized protein	N	no hit		n.d
FOKN1_2256	glutamyl- and glutaminyl-tRNA synthetases	N	J	K01885	0.025%
FOKN1_2258	signal transduction protein	N	no hit	K07182	0.014%
FOKN1_2259	DNA polymerase III subunit epsilon-like 3'-5'exonuclease	N	no hit		n.d
FOKN1_2260	choline-glycine betaine transporter	N	no hit	K02168	n.d
FOKN1_2261	universal stress protein	N	no hit		0.031%
FOKN1_2262	uncharacterized protein	N	no hit	K09933	n.d
FOKN1_2263	biopolymer transport proteins	N	no hit		n.d
FOKN1_2264	Zn-dependent protease	N	R		n.d
FOKN1_2265	Zn-dependent proteases	N	R		0.069%
FOKN1_2266	thiosulfate sulfurtransferase	N	P	K01011	0.021%
FOKN1_2267	signal transduction protein	N	no hit		n.d
FOKN1_2268	primosomal protein	Y	no hit		n.d
FOKN1_2269	thiol-disulfide isomerase-like protein	Y	no hit		n.d
FOKN1_2270	soxXA-binding protein SoxK	Y	no hit		n.d
FOKN1_2271	hydrogenase maturation factor	N	O	K04656	n.d
FOKN1_2272	hydrogenase maturation factor	N	no hit	K04655	n.d
FOKN1_2273	hydrogenase maturation protease	N	no hit		n.d
FOKN1_2274	uncharacterized protein	N	no hit	K07028	0.010%
FOKN1_2275	phosphatidylserine/phosphatidylglycerophosphate /cardiolipin synthases	N	no hit		n.d
FOKN1_2276	esterase	N	no hit	K07001	n.d
FOKN1_2277	coenzyme F420-reducing hydrogenase, alpha subunit	N	no hit	K17993	0.021%
FOKN1_2278	coenzyme F420-reducing hydrogenase, gamma subunit	N	no hit	K17994	n.d
FOKN1_2279	2-polyprenylphenol hydroxylase and related flavodoxin oxidoreductases	N	no hit	K16951	0.031%
FOKN1_2280	(NiFe) hydrogenase subunit beta	N	no hit	K17996	n.d
FOKN1_2281	UDP-glucose 4-epimerase	Y	no hit		n.d
FOKN1_2282	Ni,Fe-hydrogenase I large subunit	N	C	K06281	0.074%
FOKN1_2283	Fe-S oxidoreductase	N	C		0.051%
FOKN1_2284	uncharacterized protein	N	C		n.d

Table S1. Gene annotation of gene-coding sequences (CDSs) and protein expression in the *Thiohalobacter sp.* strain FOKN1 cells. SignalP; prediction of signal peptide sequence, Y; positive, N; negative, COG; clusters of orthologous group, KO; KEGG orthology annotated using the KAAS, PCI; protein content index, n.d; not detected.

Locus_tag	Product	SignalP	COG	KO	PCI (%)
FOKN1_2285	Ni,Fe-hydrogenase I small subunit	N	C	K06282	0.058%
FOKN1_2286	uncharacterized protein	N	no hit		n.d
FOKN1_2287	Ni,Fe-hydrogenase maturation factor	N	no hit	K03605	n.d
FOKN1_2288	cytochrome c, mono- and diheme variants	N	no hit		n.d
FOKN1_2289	hydrogenase maturation factor	N	R	K04653	n.d
FOKN1_2290	hydrogenase nickel insertion protein HypA	N	R	K04651	n.d
FOKN1_2291	hydrogenase nickel incorporation protein HypB	N	no hit	K04652	n.d
FOKN1_2292	hydrogenase expression/formation protein	N	no hit	K04654	n.d
FOKN1_2293	metal-dependent membrane protease	N	no hit		n.d
FOKN1_2294	uncharacterized protein	Y	no hit		0.020%
FOKN1_2295	UDP-glucose 4-epimerase	N	M	K01784	0.105%
FOKN1_2296	uncharacterized protein	N	M		n.d
FOKN1_2297	uncharacterized protein	N	no hit		n.d
FOKN1_2298	uncharacterized protein	N	L		n.d
FOKN1_2299	anaerobic dehydrogenases	N	no hit		n.d
FOKN1_2300	trypsin-like serine proteases	Y	no hit		n.d
FOKN1_2301	uncharacterized protein	N	no hit		0.007%
FOKN1_2302	lipopolysaccharide biosynthesis protein	N	no hit	K16695	n.d
FOKN1_2303	sulfotransferase	N	no hit		n.d
FOKN1_2304	arylsulfatase	N	no hit	K01138	n.d
FOKN1_2305	ferritin/ribonucleotide reductase-like protein	N	no hit	K09700	n.d
FOKN1_2306	ABC-type transporter, permease component	N	no hit	K09014	0.056%
FOKN1_2307	ABC-type transporter, ATPase component	N	O	K09013	0.041%
FOKN1_2308	ABC-type transporter, permease component	N	O	K09015	0.018%
FOKN1_2309	iron sulfur cluster assembly protein	N	no hit		n.d
FOKN1_2310	uncharacterized protein	N	no hit	K13628	n.d
FOKN1_2311	sulfur acceptor protein SufE	N	no hit	K02426	n.d
FOKN1_2312	uncharacterized protein	Y	no hit		0.319%
FOKN1_2313	glycosyltransferase	N	M	K13668	0.031%
FOKN1_2314	uncharacterized protein	Y	M		n.d
FOKN1_2315	asparagine synthetase	N	E		0.043%
FOKN1_2316	histone deacetylase complex	N	no hit		n.d
FOKN1_2317	O-antigen ligase	N	no hit		0.017%
FOKN1_2318	coenzyme F390 synthetase	N	H	K01912	n.d

Table S1. Gene annotation of gene-coding sequences (CDSs) and protein expression in the *Thiohalobacter sp.* strain FOKN1 cells. SignalP; prediction of signal peptide sequence, Y; positive, N; negative, COG; clusters of orthologous group, KO; KEGG orthology annotated using the KAAS, PCI; protein content index, n.d; not detected.

Locus_tag	Product	SignalP	COG	KO	PCI (%)
FOKN1_2319	uncharacterized protein	N	M		n.d
FOKN1_2320	glycosyltransferase	N	M	K03857	n.d
FOKN1_2321	glycosyltransferase	N	M		0.009%
FOKN1_2322	asparagine synthase	N	E	K01953	0.013%
FOKN1_2323	glycosyltransferase	N	no hit	K19424	n.d
FOKN1_2324	uncharacterized protein	N	no hit		n.d
FOKN1_2325	glycosyltransferase	N	M		n.d
FOKN1_2326	FAD/FMN-containing dehydrogenases	N	G		0.021%
FOKN1_2327	xylanase/chitin deacetylase	N	G		n.d
FOKN1_2328	UDP-N-acetylglucosamine 2-epimerase	N	M	K01791	0.010%
FOKN1_2329	general secretion pathway protein A	N	T		0.010%
FOKN1_2330	signal transduction histidine kinase	Y	T		0.062%
FOKN1_2331	protein-tyrosine kinase	N	no hit	K00903	0.037%
FOKN1_2332	polysaccharide chain length determinant protein	N	no hit		n.d
FOKN1_2333	polysaccharide export protein	Y	no hit	K01991	0.167%
FOKN1_2334	ABC-type sulfate transporter, permease component	N	O		n.d
FOKN1_2335	dinucleotide-utilizing enzyme	N	no hit		0.025%
FOKN1_2336	uncharacterized protein	N	QR		n.d
FOKN1_2337	SAM-dependent methyltransferases	N	QR		0.021%
FOKN1_2338	uncharacterized protein	N	no hit		0.084%
FOKN1_2339	tetratricopeptide	N	T	K15176	0.028%
FOKN1_2340	response regulator receiver protein	N	T	K02481	0.013%
FOKN1_2341	signal transduction histidine kinase	N	T		n.d
FOKN1_2342	sugar transferase	N	M	K03606	n.d
FOKN1_2343	cAMP-binding proteins	N	no hit		n.d
FOKN1_2344	NAD-dependent epimerase/dehydratase	N	no hit	K08679	0.038%
FOKN1_2345	UDP-N-acetyl-D-mannosaminuronate dehydrogenase	N	no hit		0.049%
FOKN1_2346	UDP-N-acetyl-D-mannosaminuronate dehydrogenase	N	no hit	K22926	0.095%
FOKN1_2347	DNA polymerase III subunit epsilon	N	no hit	K02342	n.d
FOKN1_2348	ribonuclease H	N	no hit	K03469	n.d
FOKN1_2349	SAM-dependent methyltransferases	N	no hit		n.d
FOKN1_2350	membrane-bound lytic murein transglycosylase D	Y	no hit	K08307	0.015%
FOKN1_2351	phytanoyl-CoA dioxygenase	N	no hit	K00477	n.d
FOKN1_2352	uncharacterized protein	N	no hit		n.d

Table S1. Gene annotation of gene-coding sequences (CDSs) and protein expression in the *Thiohalobacter sp.* strain FOKN1 cells. SignalP; prediction of signal peptide sequence, Y; positive, N; negative, COG; clusters of orthologous group, KO; KEGG orthology annotated using the KAAS, PCI; protein content index, n.d; not detected.

Locus_tag	Product	SignalP	COG	KO	PCI (%)
FOKN1_2353	carbon monoxide dehydrogenase accessory protein	N	no hit	K07402	n.d
FOKN1_2354	isoquinoline 1-oxidoreductase, beta subunit	N	no hit	K07303	0.018%
FOKN1_2355	(2Fe-2S)-binding domain-containing protein	N	no hit	K07302	n.d
FOKN1_2356	aldo/keto reductase	N	no hit		n.d
FOKN1_2357	membrane-bound lytic murein transglycosylase F	Y	no hit		n.d
FOKN1_2358	glutaredoxin	N	no hit		0.033%
FOKN1_2359	uncharacterized protein	N	no hit		n.d
FOKN1_2360	uncharacterized protein	N	FGR	K02503	n.d
FOKN1_2361	glucan phosphorylase	N	no hit	K00688	0.008%
FOKN1_2362	uncharacterized protein	N	no hit		n.d
FOKN1_2363	dienelactone hydrolase	Y	no hit		0.040%
FOKN1_2364	7-keto-8-aminopelargonate synthetase	Y	no hit		0.039%
FOKN1_2365	uncharacterized protein	N	no hit		0.078%
FOKN1_2366	transport-associated protein	N	no hit		n.d
FOKN1_2367	solute:sodium symporter small subunit	N	no hit		n.d
FOKN1_2368	ribonuclease E and G	N	no hit	K14393	n.d
FOKN1_2369	cyclic nucleotide-binding protein	N	no hit		n.d
FOKN1_2370	DNA polymerase III epsilon subunit	N	no hit		n.d
FOKN1_2371	signal transduction protein	N	no hit		n.d
FOKN1_2372	signal transduction protein	N	no hit		n.d
FOKN1_2373	signal transduction protein	N	no hit		n.d
FOKN1_2374	autoinducer 2-degrading protein lsrG	N	no hit	K11530	n.d
FOKN1_2375	iron-containing alcohol dehydrogenase	N	no hit	K04072	0.015%
FOKN1_2376	uncharacterized protein	N	no hit		0.019%
FOKN1_2377	iron-sulfur cluster regulator IscR	N	no hit	K13643	n.d
FOKN1_2378	uncharacterized protein	N	no hit		n.d
FOKN1_2379	phosphoglucomutase	N	no hit	K01835	0.034%
FOKN1_2380	putative phosphoenolpyruvate synthase regulatory protein	N	no hit	K09773	n.d
FOKN1_2381	glutamate dehydrogenase	N	no hit	K00262	0.037%
FOKN1_2382	phosphoenolpyruvate synthase	N	no hit	K01007	0.022%
FOKN1_2383	oligoribonuclease	N	no hit	K13288	0.051%
FOKN1_2384	Zn-dependent protease	N	no hit	K06013	n.d
FOKN1_2385	uncharacterized protein	Y	no hit		n.d
FOKN1_2386	pterin-4a-carbinolamine dehydratase	N	no hit	K01724	0.037%

Table S1. Gene annotation of gene-coding sequences (CDSs) and protein expression in the *Thiohalobacter sp.* strain FOKN1 cells. SignalP; prediction of signal peptide sequence, Y; positive, N; negative, COG; clusters of orthologous group, KO; KEGG orthology annotated using the KAAS, PCI; protein content index, n.d; not detected.

Locus_tag	Product	SignalP	COG	KO	PCI (%)
FOKN1_2387	GTPases	N	R	K06949	n.d
FOKN1_2388	diadenosine tetraphosphate (Ap4A) hydrolase	N	no hit	K02503	n.d
FOKN1_2389	guanosine polyphosphate pyrophosphohydrolases/synthetases	N	no hit		n.d
FOKN1_2390	pyruvate:ferredoxin oxidoreductase and related 2-oxoacid:ferredoxin oxidoreductases, beta subunit	N	no hit		n.d
FOKN1_2391	recombination protein RecR	N	no hit	K06187	n.d
FOKN1_2392	DNA-binding protein	N	no hit	K09747	0.122%
FOKN1_2393	DNA polymerase III, subunits gamma and tau	N	no hit	K02343	0.032%
FOKN1_2394	uncharacterized protein	N	no hit		0.049%
FOKN1_2395	SAM-dependent methyltransferases	N	no hit	K00552	0.110%
FOKN1_2396	SAM-dependent methyltransferases	N	no hit	K05928	n.d
FOKN1_2397	S-adenosylmethionine synthetase	N	H	K00789	0.219%
FOKN1_2398	phosphatase	N	H	K07093	n.d
FOKN1_2399	uncharacterized protein	N	no hit	K06911	n.d
FOKN1_2400	FMN-dependent NADH-azoreductase	N	no hit	K01118	n.d
FOKN1_2401	transcriptional regulator	N	no hit		n.d
FOKN1_2402	uncharacterized protein	N	no hit		0.010%
FOKN1_2403	glycosyltransferase	N	no hit	K19002	n.d
FOKN1_2404	type I restriction-modification system methyltransferase subunit	N	no hit		n.d
FOKN1_2405	uncharacterized protein	N	no hit	K09822	0.004%
FOKN1_2406	NADH:ubiquinone oxidoreductase subunit 5	N	no hit		n.d
FOKN1_2407	ADP-ribosylglycohydrolase	N	no hit		n.d
FOKN1_2408	cytochrome c peroxidase	Y	P	K00428	0.149%
FOKN1_2409	outer membrane lipoprotein	N	no hit		0.053%
FOKN1_2410	oxidoreductase	N	no hit		0.014%
FOKN1_2411	response regulator receiver domain protein	N	no hit	K07814	0.020%
FOKN1_2412	signal transduction histidine kinase	N	no hit		0.004%
FOKN1_2413	enoyl-CoA hydratase	N	no hit		n.d
FOKN1_2414	uncharacterized protein	N	no hit		n.d
FOKN1_2415	metal-dependent hydrolases	N	no hit	K00784	n.d
FOKN1_2416	uncharacterized protein	N	no hit		n.d
FOKN1_2417	nucleotidyltransferase	N	no hit		n.d

Table S1. Gene annotation of gene-coding sequences (CDSs) and protein expression in the *Thiohalobacter sp.* strain FOKN1 cells. SignalP; prediction of signal peptide sequence, Y; positive, N; negative, COG; clusters of orthologous group, KO; KEGG orthology annotated using the KAAS, PCI; protein content index, n.d; not detected.

Locus_tag	Product	SignalP	COG	KO	PCI (%)
FOKN1_2419	transmembrane protein	N	no hit		0.044%
FOKN1_2420	alpha-glucan phosphorylase	N	G	K00688	n.d
FOKN1_2422	carbon storage regulator CsrA	N	G	K03563	n.d
FOKN1_2423	aspartate kinase	N	no hit	K00928	0.080%
FOKN1_2424	alanyl-tRNA synthetase	N	no hit	K01872	0.055%
FOKN1_2425	regulatory protein RecX	N	no hit	K03565	n.d
FOKN1_2426	DNA strand exchange and recombination protein	N	no hit	K03553	0.186%
FOKN1_2427	2'-5' RNA ligase	N	no hit	K01975	n.d
FOKN1_2428	uncharacterized protein	N	no hit	K03743	0.043%
FOKN1_2429	type IV pilus assembly PilZ	N	no hit		n.d
FOKN1_2430	DNA mismatch repair protein MutS	N	no hit	K03555	0.013%
FOKN1_2431	methyl-accepting chemotaxis protein	N	NT	K13487	n.d
FOKN1_2432	chemotaxis signal transduction protein	N	no hit	K13488	n.d
FOKN1_2433	methyltransferase	N	no hit	K13486	n.d
FOKN1_2434	chemotaxis signal transduction protein	N	no hit	K13489	n.d
FOKN1_2435	chemotaxis protein histidine kinase	N	NT	K13490	0.004%
FOKN1_2436	chemotaxis response regulator	N	NT	K13491	n.d
FOKN1_2437	two-component response regulator	N	no hit	K11444	n.d
FOKN1_2438	4Fe-4S ferredoxin	N	no hit	K05524	n.d
FOKN1_2439	DNA helicase/exodeoxyribonuclease V, subunit B	N	no hit		n.d
FOKN1_2440	ATP-dependent exonuclease V beta subunit	N	no hit	K16898	n.d
FOKN1_2441	thioredoxin	N	no hit	K05838	n.d
FOKN1_2442	protein-disulfide isomerase	Y	no hit	K03981	0.059%
FOKN1_2443	tyrosine recombinase XerD	N	no hit	K04763	n.d
FOKN1_2444	acyl-CoA synthetases (AMP-forming)/AMP-acid ligases II	N	no hit		n.d
FOKN1_2445	uncharacterized protein	N	no hit	K19055	n.d
FOKN1_2446	ribosomal subunit interface protein	N	no hit		n.d
FOKN1_2447	uncharacterized protein	Y	no hit		0.018%
FOKN1_2448	molybdopterin-guanine dinucleotide biosynthesis protein A	N	H	K03752	n.d
FOKN1_2449	molybdopterin-guanine dinucleotide biosynthesis protein	N	no hit	K03753	n.d
FOKN1_2450	molybdopterin biosynthesis protein MoeA	N	no hit	K03750	0.014%
FOKN1_2451	ATP-dependent Lon protease	N	no hit	K01338	0.019%

Table S1. Gene annotation of gene-coding sequences (CDSs) and protein expression in the *Thiohalobacter sp.* strain FOKN1 cells. SignalP; prediction of signal peptide sequence, Y; positive, N; negative, COG; clusters of orthologous group, KO; KEGG orthology annotated using the KAAS, PCI; protein content index, n.d; not detected.

Locus_tag	Product	SignalP	COG	KO	PCI (%)
FOKN1_2452	thiol-disulfide isomerase and thioredoxins	N	no hit	K00384	n.d
FOKN1_2453	uncharacterized protein	N	no hit		n.d
FOKN1_2454	signal transduction protein	N	no hit		n.d
FOKN1_2455	uncharacterized protein	N	S		0.020%
FOKN1_2456	ATPase	N	R		0.076%
FOKN1_2457	uncharacterized protein	N	no hit		n.d
FOKN1_2458	ADP-ribose pyrophosphatase	N	no hit	K03574	n.d
FOKN1_2459	sulfate transporter	N	no hit	K03321	0.017%
FOKN1_2460	universal stress protein	N	no hit		0.014%
FOKN1_2461	heat shock protein 15	N	no hit	K04762	n.d
FOKN1_2462	dithiol-disulfide isomerase	N	no hit		n.d
FOKN1_2463	heat shock protein 90	N	no hit	K04079	0.030%
FOKN1_2464	cysteine methyltransferase	N	no hit	K00567	n.d
FOKN1_2465	tetratricopeptide TPR_4	Y	no hit		n.d
FOKN1_2466	50S ribosomal protein L19	N	no hit	K02884	0.412%
FOKN1_2467	tRNA (guanine-N(1)-)-methyltransferase	N	no hit	K00554	n.d
FOKN1_2468	16S rRNA processing protein RimM	N	no hit	K02860	n.d
FOKN1_2469	30S ribosomal protein S16	N	no hit	K02959	0.283%
FOKN1_2470	signal recognition particle protein	N	no hit	K03106	0.008%
FOKN1_2471	cAMP phosphodiesterase	N	no hit		n.d
FOKN1_2472	cytochrome c assembly protein	N	no hit		n.d
FOKN1_2473	Mg2+ and Co2+ transporter CorB	N	no hit		n.d
FOKN1_2474	uncharacterized protein	N	no hit		n.d
FOKN1_2475	DNA repair protein RadA	N	no hit	K04485	0.013%
FOKN1_2476	uncharacterized protein	N	no hit		n.d
FOKN1_2477	adenylate cyclase	N	no hit	K01768	n.d
FOKN1_2478	uncharacterized protein	Y	no hit		n.d
FOKN1_2479	uncharacterized protein	Y	no hit		n.d
FOKN1_2480	alanine racemase	N	no hit	K01775	n.d
FOKN1_2481	replicative DNA helicase	N	no hit	K02314	0.008%
FOKN1_2482	50S ribosomal protein L9	N	no hit	K02939	0.276%
FOKN1_2483	uncharacterized protein	N	no hit		n.d
FOKN1_2484	30S ribosomal protein S18	N	no hit	K02963	0.473%
FOKN1_2485	primosomal replication protein N	N	no hit	K02686	n.d

Table S1. Gene annotation of gene-coding sequences (CDSs) and protein expression in the *Thiohalobacter sp.* strain FOKN1 cells. SignalP; prediction of signal peptide sequence, Y; positive, N; negative, COG; clusters of orthologous group, KO; KEGG orthology annotated using the KAAS, PCI; protein content index, n.d; not detected.

Locus_tag	Product	SignalP	COG	KO	PCI (%)
FOKN1_2486	30S ribosomal protein S6	N	no hit	K02990	0.135%
FOKN1_2487	23S rRNA (guanosine-2'-O-)-methyltransferase RlmB	N	no hit	K03218	0.033%
FOKN1_2488	outer membrane protein A	N	no hit		0.107%
FOKN1_2489	exoribonuclease R	N	no hit	K12573	0.057%
FOKN1_2491	Zn-dependent hydrolases	Y	no hit		0.011%
FOKN1_2492	adenylosuccinate synthase	N	no hit	K01939	0.070%
FOKN1_2493	histidyl-tRNA synthetase 2	N	no hit	K02502	0.019%
FOKN1_2494	uncharacterized protein	N	no hit		n.d
FOKN1_2495	membrane protease	N	no hit	K04087	0.251%
FOKN1_2496	membrane protease	N	no hit	K04088	0.173%
FOKN1_2497	GTPase	N	no hit	K03665	0.008%
FOKN1_2498	uncharacterized protein	N	no hit	K03666	0.125%
FOKN1_2499	tRNA isopentenyltransferase MiaA	N	J	K00791	n.d
FOKN1_2500	DNA mismatch repair protein MutL	N	no hit	K03572	0.006%
FOKN1_2501	N-acetylmuramoyl-L-alanine amidase	N	no hit	K01448	n.d
FOKN1_2502	uncharacterized protein	N	no hit	K06925	n.d
FOKN1_2503	carbohydrate kinase	N	no hit	K17758	0.008%
FOKN1_2504	4Fe-4S binding protein	N	no hit	K18979	n.d
FOKN1_2505	collagenase and related proteases	N	no hit	K08303	n.d
FOKN1_2506	uroporphyrin-III C/tetrapyrrole(Corrin/Porphyrin) methyltransferase	N	no hit	K07056	0.022%
FOKN1_2507	lipoprotein	N	no hit	K07121	0.025%
FOKN1_2508	endonuclease	N	no hit	K07460	n.d
FOKN1_2509	phosphoheptose isomerase	N	no hit	K03271	0.021%
FOKN1_2510	uncharacterized protein	Y	no hit		1.050%
FOKN1_2511	uncharacterized protein	N	no hit		n.d
FOKN1_2512	diguanylate phosphodiesterase	N	no hit		n.d
FOKN1_2513	signal transduction histidine kinase	N	no hit		0.010%
FOKN1_2514	uncharacterized protein	N	P		n.d
FOKN1_2515	ATP adenylyltransferase	N	F	K00988	n.d
FOKN1_2516	translation initiation factor	N	F		n.d
FOKN1_2517	threonine efflux protein	N	F		n.d
FOKN1_2518	aspartate-semialdehyde dehydrogenase	Y	O		n.d
FOKN1_2519	phenazine biosynthesis PhzC/PhzF protein	N	no hit		n.d

Table S1. Gene annotation of gene-coding sequences (CDSs) and protein expression in the *Thiohalobacter sp.* strain FOKN1 cells. SignalP; prediction of signal peptide sequence, Y; positive, N; negative, COG; clusters of orthologous group, KO; KEGG orthology annotated using the KAAS, PCI; protein content index, n.d; not detected.

Locus_tag	Product	SignalP	COG	KO	PCI (%)
FOKN1_2520	uncharacterized protein	Y	no hit		n.d
FOKN1_2521	putative mitomycin resistance protein	N	no hit		n.d
FOKN1_2522	cation/cationic drug transporter	N	no hit	K11741	n.d
FOKN1_2523	plasmid maintenance system antidote protein	N	no hit	K21498	n.d
FOKN1_2524	plasmid maintenance system killer protein	N	no hit	K07334	n.d
FOKN1_2525	uncharacterized protein	N	G		n.d
FOKN1_2526	transposase	N	no hit	K07497	n.d
FOKN1_2527	transposase	N	no hit		n.d
FOKN1_2528	transposase	N	no hit		n.d
FOKN1_2529	uncharacterized protein	N	no hit		n.d
FOKN1_2530	uncharacterized protein	N	no hit		n.d
FOKN1_2531	membrane protein	Y	no hit		n.d
FOKN1_2532	uncharacterized protein	N	no hit		n.d
FOKN1_2533	NADH:ubiquinone oxidoreductase, NADH-binding subunit	N	no hit		n.d
FOKN1_2534	C4-dicarboxylate transporter	Y	no hit		n.d
FOKN1_2535	cation/multidrug efflux pump	N	no hit		n.d
FOKN1_2536	alpha-glucosidases	N	no hit		n.d
FOKN1_2537	transposase	N	no hit		n.d
FOKN1_2538	transposase	N	no hit		n.d
FOKN1_2539	integron integrase	N	no hit		n.d
FOKN1_2540	stringent starvation protein B	N	no hit	K03600	n.d
FOKN1_2541	glutathione S-transferase	N	no hit	K03599	0.077%
FOKN1_2542	cytochrome c1	Y	no hit	K00413	n.d
FOKN1_2543	cytochrome B subunit of cytochrome bc1	N	no hit	K00412	n.d
FOKN1_2544	ubiquinol-cytochrome c reductase, iron-sulfur subunit	N	no hit	K00411	0.055%
FOKN1_2545	putative GTP cyclohydrolase 1 type 2	N	no hit		n.d
FOKN1_2546	2-alkenal reductase	N	no hit	K04691	n.d
FOKN1_2547	glutaredoxin-related protein	N	no hit	K07390	n.d
FOKN1_2548	asparagine synthase	N	no hit	K01953	n.d
FOKN1_2549	periplasmic protein	Y	no hit		0.057%
FOKN1_2550	N-methylhydantoinase B/acetone carboxylase, alpha subunit	N	E	K01474	n.d
FOKN1_2551	uncharacterized protein	N	E		n.d

Table S1. Gene annotation of gene-coding sequences (CDSs) and protein expression in the *Thiohalobacter sp.* strain FOKN1 cells. SignalP; prediction of signal peptide sequence, Y; positive, N; negative, COG; clusters of orthologous group, KO; KEGG orthology annotated using the KAAS, PCI; protein content index, n.d; not detected.

Locus_tag	Product	SignalP	COG	KO	PCI (%)
FOKN1_2552	ornithine/acetylornithine aminotransferase	N	E	K00821	0.010%
FOKN1_2553	ornithine carbamoyltransferase	N	no hit	K00611	0.099%
FOKN1_2554	phosphate uptake regulator PhoU	N	no hit	K02039	n.d
FOKN1_2555	phosphate ABC transporter ATPase subunit	N	no hit	K02036	n.d
FOKN1_2556	phosphate ABC transporter inner membrane subunit PstA	N	no hit	K02038	n.d
FOKN1_2557	ABC-type uncharacterized transporter, permease component	N	no hit	K02037	n.d
FOKN1_2558	polyphosphate kinase	N	no hit	K00937	0.028%
FOKN1_2559	argininosuccinate synthase	N	no hit	K01940	0.090%
FOKN1_2560	uncharacterized protein	N	no hit		n.d
FOKN1_2561	pyruvate/2-oxoglutarate dehydrogenase complex, dihydrolipoamide dehydrogenase (E3) component	N	C	K00627	0.064%
FOKN1_2562	pyruvate/2-oxoglutarate dehydrogenase complex, dehydrogenase (E1) component, eukaryotic type, beta subunit	N	C	K00162	0.111%
FOKN1_2563	pyruvate/2-oxoglutarate dehydrogenase complex, dehydrogenase (E1) component, eukaryotic type, alpha subunit	N	C	K00161	0.177%
FOKN1_2564	dihydroorotase (dhoase) protein	N	no hit	K01465	0.042%
FOKN1_2565	uncharacterized protein	N	S	K09973	0.012%
FOKN1_2566	ribonuclease T	N	S	K03683	n.d
FOKN1_2567	ABC-type sulfate transporter, periplasmic component	N	S		n.d
FOKN1_2568	signal transduction histidine kinase	N	no hit		n.d
FOKN1_2569	response regulator receiver	N	no hit		n.d
FOKN1_2570	uncharacterized protein	Y	no hit		n.d
FOKN1_2571	cytochrome B561	N	no hit		n.d
FOKN1_2572	aspartate 1-decarboxylase	N	no hit	K01579	n.d
FOKN1_2573	pantothenate synthetase	N	no hit	K01918	n.d
FOKN1_2574	3-methyl-2-oxobutanoatehydroxymethyltransferase	N	no hit	K00606	n.d
FOKN1_2575	DNA polymerase III subunit epsilon	N	no hit	K15518	0.016%
FOKN1_2576	2-amino-4-hydroxy-6-hydroxymethyldihydropteridine pyrophosphokinase	N	no hit	K00950	n.d
FOKN1_2577	tRNA nucleotidyltransferase/poly(A) polymerase	N	no hit	K00970	n.d
FOKN1_2579	uncharacterized protein	N	no hit		n.d

Table S1. Gene annotation of gene-coding sequences (CDSs) and protein expression in the *Thiohalobacter sp.* strain FOKN1 cells. SignalP; prediction of signal peptide sequence, Y; positive, N; negative, COG; clusters of orthologous group, KO; KEGG orthology annotated using the KAAS, PCI; protein content index, n.d; not detected.

Locus_tag	Product	SignalP	COG	KO	PCI (%)
FOKN1_2580	uncharacterized protein	N	no hit		n.d
FOKN1_2581	uncharacterized protein	N	no hit		n.d
FOKN1_2582	membrane protein	N	no hit		n.d
FOKN1_2583	permease	N	no hit	K11720	n.d
FOKN1_2584	permease	N	no hit	K07091	n.d
FOKN1_2585	leucyl aminopeptidase	N	no hit	K01255	0.195%
FOKN1_2586	DNA polymerase III subunit chi	N	no hit	K02339	n.d
FOKN1_2587	valyl-tRNA synthetase	N	no hit	K01873	0.027%
FOKN1_2588	response regulator receiver	N	no hit	K07814	0.010%
FOKN1_2589	protoheme ferro-lyase	N	no hit	K01772	0.019%
FOKN1_2590	uncharacterized protein	N	no hit		n.d
FOKN1_2591	H+-ATPase subunit I	N	C		n.d
FOKN1_2592	uncharacterized protein	N	C		n.d
FOKN1_2593	uncharacterized protein	Y	no hit		n.d
FOKN1_2594	glucose-1-phosphate adenylyltransferase	N	no hit	K00975	n.d
FOKN1_2595	peptide chain release factor RF-3	N	no hit	K02837	n.d
FOKN1_2596	acetyltransferases	N	no hit	K03789	n.d
FOKN1_2597	uracil-DNA glycosylase	N	no hit	K21929	n.d
FOKN1_2598	2-isopropylmalate synthase	N	no hit	K01649	0.129%
FOKN1_2599	glycosyltransferase	Y	no hit		n.d
FOKN1_2600	superfamily II helicase	Y	no hit		n.d
FOKN1_2601	uncharacterized protein	Y	no hit		0.019%
FOKN1_2602	uncharacterized protein	Y	T		n.d
FOKN1_2603	metal-dependent phosphohydrolase	N	T		0.011%
FOKN1_2604	CDP-diacylglycerol/serineO-phosphatidyltransferase	N	no hit	K17103	n.d
FOKN1_2605	phosphatidylserine decarboxylase proenzyme	N	no hit	K01613	n.d
FOKN1_2606	ketol-acid reductoisomerase	N	no hit	K00053	0.372%
FOKN1_2607	acetolactate synthase isozyme III (small subunit) protein	N	no hit	K01653	0.130%
FOKN1_2608	acetolactate synthase isozyme III (large subunit) protein	N	no hit	K01652	0.050%
FOKN1_2609	uncharacterized protein	Y	no hit		n.d
FOKN1_2610	prolyl-tRNA synthetase	N	no hit	K01881	0.042%
FOKN1_2611	aspartyl-tRNA synthetase	N	no hit	K01876	0.126%
FOKN1_2612	regulatory protein	N	no hit		n.d
FOKN1_2613	uncharacterized protein	N	no hit		n.d

Table S1. Gene annotation of gene-coding sequences (CDSs) and protein expression in the *Thiohalobacter sp.* strain FOKN1 cells. SignalP; prediction of signal peptide sequence, Y; positive, N; negative, COG; clusters of orthologous group, KO; KEGG orthology annotated using the KAAS, PCI; protein content index, n.d; not detected.

Locus_tag	Product	SignalP	COG	KO	PCI (%)
FOKN1_2614	ABC-type transporter, permease and ATPase components	N	no hit	K06147	n.d
FOKN1_2615	uncharacterized protein	N	no hit		n.d
FOKN1_2616	RNA polymerase sigma-70 factor	N	no hit		n.d
FOKN1_2617	uncharacterized protein	Y	no hit		n.d
FOKN1_2618	uncharacterized protein	Y	C		n.d
FOKN1_2619	uncharacterized protein	N	no hit		n.d
FOKN1_2620	flagellar motor protein	N	no hit	K02557	n.d
FOKN1_2621	flagellar motor component	N	no hit	K02556	n.d
FOKN1_2622	thymidine phosphorylase	N	no hit	K18931	0.022%
FOKN1_2623	phosphoribosylpyrophosphate synthetase	N	S	K00948	n.d
FOKN1_2624	uncharacterized protein	N	S		n.d
FOKN1_2625	short-chain dehydrogenase/reductase	N	no hit		0.012%
FOKN1_2626	uncharacterized protein	N	no hit		n.d
FOKN1_2627	type I phosphodiesterase/nucleotide pyrophosphatase	N	no hit		n.d
FOKN1_2628	ATPases, ATP-binding subunit	N	no hit	K03695	0.043%
FOKN1_2629	uncharacterized protein	N	S		n.d
FOKN1_2630	N-formylmethionyl-tRNA deformylase	N	S	K01462	n.d
FOKN1_2631	amino acid-binding ACT domain protein	N	S		0.025%
FOKN1_2632	zinc transporter	N	no hit	K14716	n.d
FOKN1_2633	laccase	N	no hit	K05810	n.d
FOKN1_2634	pseudouridylate synthases	N	no hit	K06180	n.d
FOKN1_2635	DNA uptake lipoprotein	Y	no hit	K05807	0.045%
FOKN1_2636	nitrogen regulatory protein P-II	N	no hit	K04751	0.183%
FOKN1_2637	NAD+ synthetase	N	no hit	K01916	0.011%
FOKN1_2638	succinyl-CoA synthetase subunit alpha	N	no hit	K01902	0.024%
FOKN1_2639	succinyl-CoA synthetase subunit beta	N	no hit	K01903	0.016%
FOKN1_2640	uncharacterized protein	N	no hit		n.d
FOKN1_2641	signal transduction histidine kinase	N	no hit	K02668	n.d
FOKN1_2642	response regulator	N	no hit	K02667	0.017%
FOKN1_2643	TfP pilus assembly protein, major pilin PilA	N	no hit		0.211%
FOKN1_2644	uncharacterized protein	N	O		n.d
FOKN1_2645	uncharacterized protein	N	R		0.015%
FOKN1_2646	amidohydrolase	N	R		n.d
FOKN1_2647	glycosyltransferase	N	R	K00721	n.d

Table S1. Gene annotation of gene-coding sequences (CDSs) and protein expression in the *Thiohalobacter sp.* strain FOKN1 cells. SignalP; prediction of signal peptide sequence, Y; positive, N; negative, COG; clusters of orthologous group, KO; KEGG orthology annotated using the KAAS, PCI; protein content index, n.d; not detected.

Locus_tag	Product	SignalP	COG	KO	PCI (%)
FOKN1_2648	type II secretory pathway, ATPase PulE/TfP pilus assembly pathway, ATPase PilB	N	no hit	K02652	n.d
FOKN1_2649	type II secretory pathway, component PulF	N	no hit	K02653	n.d
FOKN1_2650	type II secretory pathway, prepilin signal peptidase PulO	N	no hit	K02654	n.d
FOKN1_2651	dephospho-CoA kinase	N	no hit	K00859	n.d
FOKN1_2652	uncharacterized protein	N	no hit	K18778	0.015%
FOKN1_2653	DNA gyrase inhibitor YacG	N	no hit	K09862	n.d
FOKN1_2654	uncharacterized protein	N	no hit		0.025%
FOKN1_2655	thiamine monophosphate synthase	N	no hit	K03574	n.d
FOKN1_2656	ornithine acetyltransferase/N-acetylglutamate synthase	N	no hit	K00620	0.031%
FOKN1_2657	preprotein translocase subunit SecA	N	no hit	K03070	0.047%
FOKN1_2658	metalloendopeptidase-like membrane protein	N	no hit		n.d
FOKN1_2659	uncharacterized protein	N	no hit		n.d
FOKN1_2660	UDP-3-O-(3-hydroxymyristoyl) N-acetylglucosaminedeacetylase	N	no hit	K02535	n.d
FOKN1_2661	GTPase	N	no hit	K03531	0.010%
FOKN1_2662	cell division protein FtsA	N	no hit	K03590	0.040%
FOKN1_2663	cell division protein FtsQ	N	no hit	K03589	n.d
FOKN1_2664	D-alanine-D-alanine ligase	N	no hit	K01921	0.057%
FOKN1_2665	UDP-N-acetylenolpyruvoylglucosamine reductase	N	M	K00075	0.012%
FOKN1_2666	uncharacterized protein	N	no hit		n.d
FOKN1_2667	UDP-N-acetylmuramate-alanine ligase	N	no hit	K01924	0.025%
FOKN1_2668	UDP-N-acetylglucosamine:LPS N-acetylglucosamine transferase	N	no hit	K02563	n.d
FOKN1_2669	cell division membrane protein	N	no hit	K03588	n.d
FOKN1_2670	UDP-N-acetylmuramoylalanine-D-glutamate ligase	N	no hit	K01925	0.009%
FOKN1_2671	UDP-N-acetylmuramyl pentapeptide phosphotransferase/UDP-N-acetylglucosamine-1-phosphate transferase	N	no hit	K01000	n.d
FOKN1_2672	UDP-N-acetylmuramyl pentapeptide synthase	N	no hit	K01929	0.013%
FOKN1_2673	UDP-N-acetylmuramyl tripeptide synthase	N	no hit	K01928	n.d
FOKN1_2674	cell division protein FtsI/penicillin-binding protein 2	N	no hit	K03587	n.d
FOKN1_2675	cell division protein FtsL	N	no hit	K03586	n.d
FOKN1_2676	S-adenosylmethionine-dependent methyltransferase	N	no hit	K03438	0.012%

Table S1. Gene annotation of gene-coding sequences (CDSs) and protein expression in the *Thiohalobacter sp.* strain FOKN1 cells. SignalP; prediction of signal peptide sequence, Y; positive, N; negative, COG; clusters of orthologous group, KO; KEGG orthology annotated using the KAAS, PCI; protein content index, n.d; not detected.

Locus_tag	Product	SignalP	COG	KO	PCI (%)
FOKN1_2677	uncharacterized protein	N	no hit	K03925	0.027%
FOKN1_2678	transcriptional regulator	N	L		n.d
FOKN1_2679	single-strand DNA binding protein	N	L	K03111	0.350%
FOKN1_2680	permease	N	no hit		n.d
FOKN1_2681	excinuclease ABC subunit A	N	no hit	K03701	0.013%
FOKN1_2682	uncharacterized protein	N	no hit	K22205	n.d
FOKN1_2683	50S ribosomal protein L17	N	no hit	K02879	0.291%
FOKN1_2684	DNA-directed RNA polymerase, alpha subunit	N	no hit	K03040	0.198%
FOKN1_2685	30S ribosomal protein S4	N	no hit	K02986	0.152%
FOKN1_2686	30S ribosomal protein S11	N	no hit	K02948	0.201%
FOKN1_2687	30S ribosomal protein S13	N	no hit	K02952	0.661%
FOKN1_2688	preprotein translocase subunit SecY	N	no hit	K03076	n.d
FOKN1_2689	50S ribosomal protein L15	N	no hit	K02876	0.536%
FOKN1_2690	50S ribosomal protein L30	N	no hit	K02907	0.090%
FOKN1_2691	30S ribosomal protein S5	N	no hit	K02988	0.505%
FOKN1_2692	50S ribosomal protein L18	N	no hit	K02881	0.334%
FOKN1_2693	50S ribosomal protein L6	N	no hit	K02933	0.377%
FOKN1_2694	30s ribosomal protein s8	N	J	K02994	0.333%
FOKN1_2695	30S ribosomal protein S14p	N	no hit	K02954	0.135%
FOKN1_2696	50S ribosomal protein L5	N	no hit	K02931	0.442%
FOKN1_2697	50S ribosomal protein L24	N	no hit	K02895	0.389%
FOKN1_2698	50S ribosomal protein L14	N	no hit	K02874	0.400%
FOKN1_2699	30S ribosomal protein S17	N	no hit	K02961	0.700%
FOKN1_2700	50S ribosomal protein L29	N	no hit	K02904	0.059%
FOKN1_2701	50S ribosomal protein L16	N	no hit	K02878	0.044%
FOKN1_2702	30S ribosomal protein S3	N	no hit	K02982	0.260%
FOKN1_2703	50S ribosomal protein L22	N	no hit	K02890	0.334%
FOKN1_2704	30S ribosomal protein S19	N	no hit	K02965	0.163%
FOKN1_2705	50S ribosomal protein L2	N	no hit	K02886	0.250%
FOKN1_2706	50S ribosomal protein L23	N	no hit	K02892	0.558%
FOKN1_2707	50S ribosomal protein L4	N	no hit	K02926	n.d
FOKN1_2708	50S ribosomal protein L3	N	no hit	K02906	1.430%
FOKN1_2709	30S ribosomal protein S10	N	no hit	K02946	0.219%
FOKN1_2710	translation elongation factor Tu	N	no hit	K02358	0.754%

Table S1. Gene annotation of gene-coding sequences (CDSs) and protein expression in the *Thiohalobacter sp.* strain FOKN1 cells. SignalP; prediction of signal peptide sequence, Y; positive, N; negative, COG; clusters of orthologous group, KO; KEGG orthology annotated using the KAAS, PCI; protein content index, n.d; not detected.

Locus_tag	Product	SignalP	COG	KO	PCI (%)
FOKN1_2711	translation elongation factor G	N	no hit	K02355	0.195%
FOKN1_2712	30S ribosomal protein S7	N	J	K02992	0.239%
FOKN1_2713	30S ribosomal protein S12	N	no hit	K02950	0.369%
FOKN1_2714	DNA-directed RNA polymerase subunit beta	N	no hit	K03046	0.253%
FOKN1_2715	DNA-directed RNA polymerase subunit beta	N	no hit	K03043	0.213%
FOKN1_2716	50S ribosomal protein L7/L12	N	no hit	K02935	0.233%
FOKN1_2717	50S ribosomal protein L10	N	J	K02864	0.457%
FOKN1_2718	50S ribosomal protein L1	N	J	K02863	0.095%
FOKN1_2719	50S ribosomal protein L11	N	no hit	K02867	0.475%
FOKN1_2720	nusG antitermination factor	N	no hit	K02601	n.d
FOKN1_2721	protein translocase subunit SecE	N	no hit		n.d
FOKN1_2723	translation elongation factor Tu	N	no hit	K02358	0.685%
FOKN1_2727	UDP-glucose 6-dehydrogenase	N	no hit	K00012	0.081%
FOKN1_2728	prolyl-tRNA synthetase	N	no hit		n.d
FOKN1_2729	ADP-ribose pyrophosphatase	N	no hit	K18453	n.d
FOKN1_2730	Zn-dependent protease	N	no hit	K06973	n.d
FOKN1_2731	alcohol dehydrogenase	N	CR		0.142%
FOKN1_2732	uncharacterized protein	N	no hit		n.d
FOKN1_2733	permease	N	no hit		n.d
FOKN1_2734	ammonium transporter	N	no hit	K03320	n.d
FOKN1_2735	histidine ammonia-lyase	N	no hit		n.d
FOKN1_2736	recA/RadA recombinase	N	no hit		n.d
FOKN1_2737	transcriptional repressor	N	no hit	K01356	0.020%
FOKN1_2738	uncharacterized protein	Y	no hit		n.d
FOKN1_2739	uncharacterized protein	Y	no hit		n.d
FOKN1_2741	uncharacterized protein	N	no hit		n.d
FOKN1_2742	transcriptional regulator	N	no hit	K03525	0.015%
FOKN1_2743	biotin/acetyl-CoA-carboxylase ligase	N	no hit	K03524	n.d
FOKN1_2744	signal transduction protein	N	no hit		n.d
FOKN1_2745	uncharacterized protein	Y	no hit		n.d
FOKN1_2750	transcriptional regulator	N	no hit		n.d
FOKN1_2752	tyrosyl-tRNA synthetase	N	no hit	K01866	0.013%
FOKN1_2753	peptidase M23	N	no hit		n.d
FOKN1_2754	anhydro-N-acetylmuramic acid kinase	N	no hit	K09001	n.d

Table S1. Gene annotation of gene-coding sequences (CDSs) and protein expression in the *Thiohalobacter sp.* strain FOKN1 cells. SignalP; prediction of signal peptide sequence, Y; positive, N; negative, COG; clusters of orthologous group, KO; KEGG orthology annotated using the KAAS, PCI; protein content index, n.d; not detected.

Locus_tag	Product	SignalP	COG	KO	PCI (%)
FOKN1_2755	2-methylcitrate synthase/citrate synthase II	N	C	K01647	n.d
FOKN1_2756	iron-sulfur cluster insertion protein ErpA	N	no hit	K15724	n.d
FOKN1_2757	integral membrane protein CcmA	N	no hit		n.d
FOKN1_2758	uncharacterized protein	N	no hit		n.d
FOKN1_2759	N-acetyl-gamma-glutamyl-phosphate reductase	N	no hit	K00145	0.018%
FOKN1_2760	chloride channel protein	N	no hit		n.d
FOKN1_2761	transmembrane protein	N	no hit		n.d
FOKN1_2762	thiol-disulfide isomerase and thioredoxins	N	no hit		0.037%
FOKN1_2763	uncharacterized protein	N	no hit	K08973	n.d
FOKN1_2764	uncharacterized protein	N	S		n.d
FOKN1_2765	cytochrome c peroxidase	N	no hit		n.d
FOKN1_2766	bacterioferritin	N	no hit	K03594	n.d
FOKN1_2767	bacterioferritin-associated ferredoxin	N	no hit		n.d
FOKN1_2768	signal transduction protein	N	no hit	K08484	0.010%
FOKN1_2769	NTP pyrophosphohydrolases	N	no hit	K08311	n.d
FOKN1_2770	phosphoserine phosphatase	N	no hit		n.d
FOKN1_2771	ABC-type Fe3+-siderophore transporter, permease component	N	no hit	K02015	n.d
FOKN1_2772	ABC-type cobalamin/Fe3+-siderophores transporters, ATPase components	N	PH	K02013	n.d
FOKN1_2773	uncharacterized protein	N	PH		n.d
FOKN1_2774	cell division protein FtsI	N	no hit		n.d
FOKN1_2775	iron complex outer membrane recepter protein	N	no hit	K16092	0.212%
FOKN1_2776	periplasmic protein TonB	N	M	K03832	n.d
FOKN1_2777	cobalamin adenosyltransferase protein	N	no hit	K00798	0.022%
FOKN1_2778	ABC-type Fe3+-hydroxamate transporter, periplasmic component	N	no hit	K06858	n.d
FOKN1_2779	cobyric acid synthase	N	no hit	K02232	n.d
FOKN1_2780	secondary thiamine-phosphate synthase	N	no hit		n.d
FOKN1_2781	GTPase	N	no hit	K06207	0.016%
FOKN1_2782	glycyl-tRNA synthetase, alpha subunit	N	no hit		n.d
FOKN1_2783	thiol-disulfide isomerase and thioredoxins	N	no hit		n.d
FOKN1_2784	uncharacterized protein	N	no hit		n.d
FOKN1_2785	outer membrane protein	N	no hit		n.d

Table S1. Gene annotation of gene-coding sequences (CDSs) and protein expression in the *Thiohalobacter sp.* strain FOKN1 cells. SignalP; prediction of signal peptide sequence, Y; positive, N; negative, COG; clusters of orthologous group, KO; KEGG orthology annotated using the KAAS, PCI; protein content index, n.d; not detected.

Locus_tag	Product	SignalP	COG	KO	PCI (%)
FOKN1_2786	ribonuclease BN	N	no hit	K07058	n.d
FOKN1_2787	neurofilament triplet H1-like protein	N	no hit		n.d
FOKN1_2788	uncharacterized protein	Y	no hit		0.067%
FOKN1_2789	cellulase M	N	no hit		n.d
FOKN1_2790	glycosyltransferase	N	M		n.d
FOKN1_2791	response regulator receiver	N	M	K12129	n.d
FOKN1_2792	3-hydroxydecanoyl-ACP dehydratase	N	no hit	K01716	0.057%
FOKN1_2793	3-oxoacyl-ACP synthase	N	no hit	K00647	0.032%
FOKN1_2794	outer membrane lipoprotein	Y	no hit		n.d
FOKN1_2795	lipoprotein	N	no hit		n.d
FOKN1_2796	ATP-dependent helicase HrpA	N	no hit	K03578	0.009%
FOKN1_2797	uncharacterized protein	Y	no hit		n.d
FOKN1_2798	permease	N	R	K06902	n.d
FOKN1_2799	uncharacterized protein	Y	no hit		n.d
FOKN1_2800	tRNA(Met) cytidine acetyltransferase TmcA	N	no hit	K06957	n.d
FOKN1_2801	DNA/RNA helicases	N	no hit		n.d
FOKN1_2802	uracil-DNA glycosylase	N	no hit		n.d
FOKN1_2803	uncharacterized protein	Y	O	K06888	n.d
FOKN1_2804	Zn-dependent protease	Y	O		n.d
FOKN1_2805	acetylornithine deacetylase/succinyl-diaminopimelate desuccinylase	Y	no hit		n.d
FOKN1_2806	uncharacterized protein	N	no hit		n.d
FOKN1_2807	electron transport protein SCO1/SenC	N	no hit	K07152	n.d
FOKN1_2808	heme/copper-type cytochrome/quinol oxidases, subunit 1	N	T		n.d
FOKN1_2809	heme/copper-type cytochrome/quinol oxidases, subunit 2	N	T		n.d
FOKN1_2810	response regulator	N	T		n.d
FOKN1_2811	CRISPR-associated protein Cas2	N	no hit	K09951	n.d
FOKN1_2812	CRISPR-associated Cas1 family protein	N	no hit	K15342	n.d
FOKN1_2813	CRISPR-associated protein Cse3	N	no hit	K19126	n.d
FOKN1_2814	CRISPR-associated protein Cas5/CasD, subtype TIGR01868	N	no hit	K19125	0.015%
FOKN1_2815	CRISPR-associated protein, Cse4 family	N	no hit	K19124	0.073%
FOKN1_2816	uncharacterized protein	N	no hit		n.d
FOKN1_2817	CRISPR-associated protein, Cse1	N	no hit	K19123	0.010%

Table S1. Gene annotation of gene-coding sequences (CDSs) and protein expression in the *Thiohalobacter sp.* strain FOKN1 cells. SignalP; prediction of signal peptide sequence, Y; positive, N; negative, COG; clusters of orthologous group, KO; KEGG orthology annotated using the KAAS, PCI; protein content index, n.d; not detected.

Locus_tag	Product	SignalP	COG	KO	PCI (%)
FOKN1_2818	O-antigen biosynthesis protein RbfC	N	no hit	K07012	n.d
FOKN1_2819	peroxiredoxin	N	no hit		n.d
FOKN1_2820	uncharacterized protein	N	no hit		n.d
FOKN1_2821	transcriptional regulator	N	no hit		n.d
FOKN1_2822	sugar phosphatases	N	V	K11725	n.d
FOKN1_2823	cation/multidrug efflux pump	N	V		n.d
FOKN1_2824	efflux transporter	N	V		n.d
FOKN1_2825	SAM-dependent methyltransferases	N	no hit		n.d
FOKN1_2826	alpha/beta hydrolase fold protein	Y	no hit		n.d
FOKN1_2827	cysteine desulfurase	N	E	K04487	n.d
FOKN1_2829	GTPase	N	no hit	K06942	0.011%
FOKN1_2830	peptidyl-tRNA hydrolase	N	no hit	K01056	n.d
FOKN1_2831	50S ribosomal protein L25	N	no hit	K02897	n.d
FOKN1_2832	ribose-phosphate pyrophosphokinase	N	no hit	K00948	n.d
FOKN1_2834	4-diphosphocytidyl-2C-methyl-D-erythritolkinase	N	no hit	K00919	n.d
FOKN1_2835	outer membrane lipoprotein	N	no hit	K02494	n.d
FOKN1_2836	uncharacterized protein	N	no hit		0.040%
FOKN1_2837	glutamyl-tRNA reductase	N	no hit	K02492	n.d
FOKN1_2838	peptide chain release factor 1	N	no hit	K02835	n.d
FOKN1_2839	N5-glutamine methyltransferase	N	no hit	K02493	n.d
FOKN1_2840	tartrate dehydratase alpha subunit/fumarate hydratase class I	N	no hit		n.d
FOKN1_2841	dinucleotide-utilizing protein	N	no hit	K21029	n.d
FOKN1_2842	exoribonuclease R	Y	no hit		n.d
FOKN1_2843	membrane protein	N	no hit	K08984	n.d
FOKN1_2844	hydrolase	N	no hit		0.013%
FOKN1_2845	metal-dependent proteiase	N	no hit		n.d
FOKN1_2846	Asp-tRNA Asn/Glu-tRNA Gln amidotransferase B subunit	N	no hit	K02434	0.114%
FOKN1_2847	Asp-tRNA Asn/Glu-tRNA Gln amidotransferase A subunit	N	no hit	K02433	0.103%
FOKN1_2848	Asp-tRNA Asn/Glu-tRNA Gln amidotransferase C subunit	N	no hit	K02435	n.d
FOKN1_2849	rod shape-determining protein MreB	N	no hit	K03569	0.187%

Table S1. Gene annotation of gene-coding sequences (CDSs) and protein expression in the *Thiohalobacter sp.* strain FOKN1 cells. SignalP; prediction of signal peptide sequence, Y; positive, N; negative, COG; clusters of orthologous group, KO; KEGG orthology annotated using the KAAS, PCI; protein content index, n.d; not detected.

Locus_tag	Product	SignalP	COG	KO	PCI (%)
FOKN1_2850	cell shape-determining protein MreC	N	no hit	K03570	n.d
FOKN1_2851	rod shape-determining protein MreD	N	no hit	K03571	n.d
FOKN1_2852	cell division protein FtsI	N	no hit	K05515	n.d
FOKN1_2853	rod shape-determining protein RodA	N	no hit	K05837	n.d
FOKN1_2854	lytic murein transglycosylase B	Y	no hit	K08305	0.012%
FOKN1_2855	octanoyltransferase	Y	no hit	K03642	n.d
FOKN1_2856	D-alanyl-D-alanine carboxypeptidase	Y	no hit	K07258	0.077%
FOKN1_2857	D-amino-acid transaminase	N	P	K00824	0.028%
FOKN1_2858	cation efflux system protein CzcA	N	P	K15726	n.d
FOKN1_2859	cobalt/zinc/cadmium efflux transporter	N	P	K15727	0.018%
FOKN1_2860	outer membrane protein	Y	MU		0.078%
FOKN1_2861	ATPase	N	no hit		n.d
FOKN1_2862	uncharacterized protein	N	no hit	K09158	n.d
FOKN1_2863	lipoate-protein ligase B	N	no hit	K03801	n.d
FOKN1_2864	lipoyl synthase	N	no hit	K03644	n.d
FOKN1_2865	anaerobic dehydrogenases	N	no hit		n.d
FOKN1_2866	DNA polymerase III, alpha subunit	N	no hit		n.d
FOKN1_2867	uncharacterized protein	N	no hit	K12446	n.d
FOKN1_2868	succinylglutamate desuccinylase/aspartoacylase	N	no hit		n.d
FOKN1_2869	uncharacterized protein	N	no hit		n.d
FOKN1_2870	nucleotidyl transferase	N	no hit	K00992	n.d
FOKN1_2871	aminoglycoside phosphotransferase	N	V	K07102	n.d
FOKN1_2872	cation/multidrug efflux pump	N	V	K18989	n.d
FOKN1_2873	efflux membrane fusion protein	N	no hit		n.d
FOKN1_2874	lPS-assembly protein LptD	Y	no hit	K04744	n.d
FOKN1_2875	peptidyl-prolyl isomerase	Y	no hit	K03771	0.021%
FOKN1_2876	4-hydroxythreonine-4-phosphate dehydrogenase	N	no hit	K00097	0.019%
FOKN1_2877	dimethyladenosine transferase	N	no hit	K02528	n.d
FOKN1_2878	leucyl aminopeptidase	N	no hit		n.d
FOKN1_2879	universal stress protein UspA	N	no hit	K06149	0.076%
FOKN1_2880	metal-dependent membrane protease	N	no hit		n.d
FOKN1_2881	uncharacterized protein	N	no hit		n.d
FOKN1_2882	6-phosphogluconolactonase	N	no hit	K01057	n.d
FOKN1_2883	glucokinase	N	G	K00845	n.d

Table S1. Gene annotation of gene-coding sequences (CDSs) and protein expression in the *Thiohalobacter sp.* strain FOKN1 cells. SignalP; prediction of signal peptide sequence, Y; positive, N; negative, COG; clusters of orthologous group, KO; KEGG orthology annotated using the KAAS, PCI; protein content index, n.d; not detected.

Locus_tag	Product	SignalP	COG	KO	PCI (%)
FOKN1_2884	uncharacterized protein	N	no hit	K06195	n.d
FOKN1_2885	bis(5'nucleosyl)-tetraphosphatase ApaH	N	no hit	K01525	n.d
FOKN1_2886	ABC-type sugar transporter, periplasmic component	Y	no hit		n.d
FOKN1_2887	dihydrofolate reductase	N	no hit	K00287	n.d
FOKN1_2888	thymidylate synthase	N	no hit	K00560	n.d
FOKN1_2889	prolipoprotein diacylglyceryl transferase	N	no hit	K13292	n.d
FOKN1_2890	putative SAM-dependent methyltransferase	N	no hit	K06969	n.d
FOKN1_2891	sugar transporter	N	no hit		n.d
FOKN1_2892	glycosyltransferase	N	no hit		n.d
FOKN1_2893	uncharacterized protein	N	no hit		n.d
FOKN1_2894	glycosyltransferase	N	no hit		n.d
FOKN1_2895	uncharacterized protein	N	no hit	K06888	0.016%
FOKN1_2896	uncharacterized protein	N	no hit	K06193	n.d
FOKN1_2897	redox protein	N	no hit	K04085	n.d
FOKN1_2898	transcriptional regulator	N	no hit		n.d
FOKN1_2899	DNA-directed RNA polymerase, beta subunit	N	no hit		n.d
FOKN1_2900	multisensor diguanylate cyclase/phophodiesterase	N	no hit		n.d
FOKN1_2901	sugar kinases	N	no hit	K00856	0.054%
FOKN1_2902	diacylglycerol kinase	N	M	K00901	n.d
FOKN1_2903	glycine dehydrogenase subunit 2	N	no hit	K00283	0.046%
FOKN1_2904	peroxiredoxin	N	no hit	K11065	n.d
FOKN1_2905	glycine dehydrogenase subunit 1	N	no hit	K00282	0.008%
FOKN1_2906	glycine cleavage system protein H	N	E	K02437	n.d
FOKN1_2907	glycine cleavage system aminomethyltransferase T	N	no hit	K00605	0.015%
FOKN1_2908	cytidylate kinase	N	F		0.009%
FOKN1_2909	2-polyprenyl-6-methoxyphenol hydroxylase and related FAD-dependent oxidoreductases	N	HC	K18800	0.015%
FOKN1_2910	2-polyprenyl-6-methoxyphenol hydroxylase and related FAD-dependent oxidoreductases	N	no hit	K03185	n.d
FOKN1_2911	peptidase M24	N	no hit	K01262	n.d
FOKN1_2912	uncharacterized protein	N	no hit	K09895	n.d
FOKN1_2913	uncharacterized protein	N	no hit		n.d
FOKN1_2914	uncharacterized protein	N	no hit	K09888	0.135%
FOKN1_2915	uncharacterized protein	N	no hit		n.d

Table S1. Gene annotation of gene-coding sequences (CDSs) and protein expression in the *Thiohalobacter sp.* strain FOKN1 cells. SignalP; prediction of signal peptide sequence, Y; positive, N; negative, COG; clusters of orthologous group, KO; KEGG orthology annotated using the KAAS, PCI; protein content index, n.d; not detected.

Locus_tag	Product	SignalP	COG	KO	PCI (%)
FOKN1_2916	5-formyltetrahydrofolate cyclo-ligase	N	no hit	K01934	n.d
FOKN1_2917	uncharacterized protein	N	no hit		0.075%
FOKN1_2918	uncharacterized protein	N	no hit		n.d
FOKN1_2919	threonine dehydratase	N	no hit	K01754	0.031%
FOKN1_2920	ribose-5-phosphate isomerase A	N	no hit	K01807	0.055%
FOKN1_2921	FAD/FMN-containing dehydrogenases	N	no hit		0.019%
FOKN1_2922	porin	Y	no hit		1.257%
FOKN1_2923	outer membrane protein	Y	no hit	K07275	n.d
FOKN1_2925	30S ribosomal protein S9	N	no hit	K02996	0.130%
FOKN1_2926	50S ribosomal protein L13	N	no hit	K02871	0.641%
FOKN1_2927	uncharacterized protein	N	no hit		n.d
FOKN1_2928	ferredoxin 2fe-2s protein	N	no hit		n.d
FOKN1_2929	ubiquinone biosynthesis protein COQ7	N	no hit	K06134	n.d
FOKN1_2930	S-adenosylmethionine decarboxylase	N	no hit	K01611	0.062%
FOKN1_2931	lactoylglutathione lyase	N	no hit		n.d
FOKN1_2932	redox protein	N	no hit	K07397	n.d
FOKN1_2933	DNA-binding transcriptional dual regulator Crp	N	no hit	K10914	0.037%
FOKN1_2934	indole-3-glycerol phosphate synthase	N	no hit	K01609	n.d
FOKN1_2935	anthranilate phosphoribosyltransferase	N	no hit	K00766	0.012%
FOKN1_2936	anthranilate synthase component II	N	no hit	K01658	n.d
FOKN1_2937	uncharacterized protein	N	no hit		n.d
FOKN1_2938	anthranilate synthase component I	N	no hit	K01657	0.021%
FOKN1_2939	phosphoglycolate phosphatase	N	no hit	K01091	n.d
FOKN1_2940	ribulose-phosphate 3-epimerase	N	no hit	K01783	0.115%
FOKN1_2941	uncharacterized protein	Y	no hit		n.d
FOKN1_2942	uncharacterized protein	N	no hit		n.d
FOKN1_2943	uncharacterized protein	N	no hit	K05801	n.d
FOKN1_2944	uncharacterized protein	N	L		n.d
FOKN1_2945	deoxyinosine 3'endonuclease	N	L	K05982	n.d
FOKN1_2946	aspartate-semialdehyde dehydrogenase	N	no hit		n.d
FOKN1_2947	uncharacterized protein	N	no hit		n.d
FOKN1_2948	ketohexokinase	N	no hit	K00846	n.d
FOKN1_2949	uncharacterized protein	N	no hit	K07376	n.d
FOKN1_2950	DNA-binding protein	N	no hit		0.686%

Table S1. Gene annotation of gene-coding sequences (CDSs) and protein expression in the *Thiohalobacter sp.* strain FOKN1 cells. SignalP; prediction of signal peptide sequence, Y; positive, N; negative, COG; clusters of orthologous group, KO; KEGG orthology annotated using the KAAS, PCI; protein content index, n.d; not detected.

Locus_tag	Product	SignalP	COG	KO	PCI (%)
FOKN1_2951	uncharacterized protein	N	no hit		n.d
FOKN1_2952	uncharacterized protein	N	no hit		n.d
FOKN1_2953	type II secretory pathway, component ExeA	N	no hit	K02450	0.037%
FOKN1_2954	P pilus assembly protein, porin PapC	N	no hit		n.d
FOKN1_2955	hydrolase	N	no hit	K07018	n.d
FOKN1_2956	transcription-repair coupling factor	N	no hit		n.d
FOKN1_2957	ribonucleotide reductase, alpha subunit	N	no hit		0.034%
FOKN1_2958	ribonucleotide reductase, alpha subunit	N	no hit	K00525	0.040%
FOKN1_2959	membrane protein	N	no hit		n.d
FOKN1_2960	molecular chaperon	N	no hit	K13993	0.148%
FOKN1_2961	molecular chaperon	N	no hit	K05516	n.d
FOKN1_2962	transcriptional regulator	N	no hit		n.d
FOKN1_2963	heat shock protein Hsp20	N	no hit	K13993	0.067%
FOKN1_2964	histone acetyltransferase HPA2	N	KR		0.075%
FOKN1_2965	uncharacterized protein	N	no hit		n.d
FOKN1_2966	ABC-type transporter, auxiliary component	N	no hit	K07323	n.d
FOKN1_2967	uncharacterized protein	N	T		n.d
FOKN1_2968	queuine/archaeosine tRNA-ribosyltransferase	N	T		n.d
FOKN1_2969	sulfate transporter	N	P	K03321	n.d
FOKN1_2970	uncharacterized protein	N	no hit		n.d
FOKN1_2971	ABC-type molybdate transporter, periplasmic component	N	no hit	K02020	0.014%
FOKN1_2972	ABC-type molybdate transporter, permease component	N	no hit	K02018	n.d
FOKN1_2973	ABC-type molybdate transporter, ATPase component	N	no hit	K02017	n.d
FOKN1_2974	serine/threonine protein kinase	N	no hit		n.d
FOKN1_2975	excinuclease ABC C subunit domain-containing protein	N	no hit	K07461	n.d
FOKN1_2976	monovalent cation: proton antiporter-2	N	no hit		n.d
FOKN1_2977	alanine racemase	N	no hit		n.d
FOKN1_2978	uncharacterized protein	N	no hit		n.d
FOKN1_2979	fructose-bisphosphate aldolase	N	no hit	K01624	0.458%
FOKN1_2980	pyruvate kinase	N	no hit	K00873	0.106%
FOKN1_2981	phosphoglycerate kinase	N	no hit	K00927	0.441%
FOKN1_2982	glyceraldehyde-3-phosphate dehydrogenase/erythrose-4-phosphate dehydrogenase	N	no hit	K00134	0.903%
FOKN1_2983	transketolase	N	no hit	K00615	0.431%

Table S1. Gene annotation of gene-coding sequences (CDSs) and protein expression in the *Thiohalobacter sp.* strain FOKN1 cells. SignalP; prediction of signal peptide sequence, Y; positive, N; negative, COG; clusters of orthologous group, KO; KEGG orthology annotated using the KAAS, PCI; protein content index, n.d; not detected.

Locus_tag	Product	SignalP	COG	KO	PCI (%)
FOKN1_2984	glycerol-3-phosphate O-acyltransferase	N	H		n.d
FOKN1_2985	thiol-disulfide isomerase and thioredoxins	N	H		n.d
FOKN1_2986	S-adenosylmethionine synthetase	N	H	K00789	0.015%
FOKN1_2987	S-adenosyl-L-homocysteine hydrolase	N	no hit	K01251	0.615%
FOKN1_2988	5,10-methylenetetrahydrofolate reductase	N	no hit	K00297	0.083%
FOKN1_2989	uncharacterized protein	N	no hit	K09761	n.d
FOKN1_2990	uncharacterized protein	Y	no hit		n.d
FOKN1_2991	response regulator	N	T	K07714	n.d
FOKN1_2992	signal transduction histidine kinase	N	T	K10125	n.d
FOKN1_2993	uncharacterized protein	Y	T		n.d
FOKN1_2994	anion transporter	N	T	K14445	n.d
FOKN1_2995	uncharacterized protein	N	no hit		n.d
FOKN1_2996	uncharacterized protein	N	no hit	K06598	n.d
FOKN1_2997	chemotaxis response regulator	N	no hit	K06597	n.d
FOKN1_2998	chemotaxis protein histidine kinase	N	no hit	K06596	0.002%
FOKN1_2999	methyltransferase	N	no hit	K02661	n.d
FOKN1_3000	methyl-accepting chemotaxis protein	Y	no hit	K02660	0.009%
FOKN1_3001	chemotaxis signal transduction protein	N	no hit	K02659	n.d
FOKN1_3002	twitching motility protein PilH	N	no hit	K02658	n.d
FOKN1_3003	response regulator receiver protein	N	no hit	K02657	n.d
FOKN1_3004	glutamate-cysteine ligase	N	no hit	K01919	n.d
FOKN1_3005	glutathione synthetase	N	no hit	K01920	0.012%
FOKN1_3006	lipoprotein	N	H	K03734	0.043%
FOKN1_3007	uncharacterized protein	N	no hit		n.d
FOKN1_3008	heptaprenyl diphosphate synthase component I	N	no hit		n.d
FOKN1_3009	periplasmic protein TonB	N	M	K03832	n.d
FOKN1_3010	amino acid permease	N	no hit		n.d
FOKN1_3011	transcriptional regulator	N	no hit	K07735	n.d
FOKN1_3012	endonuclease	N	no hit	K07447	n.d
FOKN1_3013	uracil phosphoribosyltransferase	N	no hit	K02825	0.036%
FOKN1_3014	aspartate carbamoyltransferase catalytic subunit	N	no hit	K00609	0.108%
FOKN1_3015	dihydroorotase	N	no hit	K01465	0.039%
FOKN1_3016	2-polyprenylphenol hydroxylase	N	HC	K02823	n.d
FOKN1_3017	uncharacterized protein	N	no hit	K07401	n.d

Table S1. Gene annotation of gene-coding sequences (CDSs) and protein expression in the *Thiohalobacter sp.* strain FOKN1 cells. SignalP; prediction of signal peptide sequence, Y; positive, N; negative, COG; clusters of orthologous group, KO; KEGG orthology annotated using the KAAS, PCI; protein content index, n.d; not detected.

Locus_tag	Product	SignalP	COG	KO	PCI (%)
FOKN1_3018	TfP pilus assembly protein, ATPase PilU	N	no hit	K02670	0.038%
FOKN1_3019	TfP pilus assembly protein, pilus retraction ATPase PilT	N	no hit	K02669	0.128%
FOKN1_3020	uncharacterized protein	N	no hit	K06997	n.d
FOKN1_3021	pyrroline-5-carboxylate reductase	N	no hit	K00286	0.037%
FOKN1_3022	integral membrane protein	N	no hit	K02221	n.d
FOKN1_3023	uncharacterized protein	N	no hit	K09131	n.d
FOKN1_3024	GTPase	N	no hit		n.d
FOKN1_3025	uncharacterized protein	Y	no hit		0.311%
FOKN1_3026	uncharacterized protein	N	no hit	K09792	n.d
FOKN1_3027	exodeoxyribonuclease III	N	no hit	K01142	n.d
FOKN1_3028	orotate phosphoribosyltransferase	N	no hit	K00762	0.028%
FOKN1_3029	permease	N	no hit		n.d
FOKN1_3030	uncharacterized protein	N	no hit		n.d
FOKN1_3031	acetylglutamate kinase	N	no hit	K00930	0.074%
FOKN1_3032	phosphomannomutase	N	no hit	K15778	0.069%
FOKN1_3033	deoxyuridine 5'-triphosphatenucleotidohydrolase	N	no hit	K01520	0.082%
FOKN1_3034	phosphopantothenoylcysteine decarboxylase/phosphopantothenate-cysteine ligase	N	no hit	K13038	0.038%
FOKN1_3035	DNA repair protein RadC	N	no hit	K03630	n.d
FOKN1_3036	50S ribosomal protein L28	N	no hit	K02902	0.251%
FOKN1_3037	50S ribosomal protein L33	N	J	K02913	n.d
FOKN1_3038	fatty acid desaturase	N	no hit		n.d
FOKN1_3039	uncharacterized protein	N	no hit		n.d
FOKN1_3040	diguanylate phosphodiesterase	N	no hit		0.009%
FOKN1_3041	N-methylhydantoinase A/acetone carboxylase subunit beta	N	EQ	K01473	n.d
FOKN1_3042	ABC-type multidrug transporter, ATPase component	N	no hit	K01990	0.012%
FOKN1_3043	ABC-type transporter, permease component	N	no hit		n.d
FOKN1_3044	ABC-type uncharacterized transporte, auxiliary component	N	N		0.086%
FOKN1_3045	transcription-repair coupling factor	N	no hit		n.d
FOKN1_3046	pyruvate/2-oxoglutarate dehydrogenase complex, dihydrolipoamide dehydrogenase (E3) component	N	no hit	K00382	n.d
FOKN1_3047	formamidopyrimidine-DNA glycosylase Fpg	N	no hit	K10563	n.d

Table S1. Gene annotation of gene-coding sequences (CDSs) and protein expression in the *Thiohalobacter sp.* strain FOKN1 cells. SignalP; prediction of signal peptide sequence, Y; positive, N; negative, COG; clusters of orthologous group, KO; KEGG orthology annotated using the KAAS, PCI; protein content index, n.d; not detected.

Locus_tag	Product	SignalP	COG	KO	PCI (%)
FOKN1_3048	gamma-glutamyltransferase	N	no hit	K00681	n.d
FOKN1_3049	uncharacterized protein	N	no hit		n.d
FOKN1_3050	ferredoxin	N	no hit		n.d
FOKN1_3051	pantetheine-phosphate adenylyltransferase	N	no hit	K00954	0.039%
FOKN1_3052	ribosomal RNA small subunit methyltransferase D	N	no hit	K08316	n.d
FOKN1_3053	uncharacterized protein	Y	no hit		n.d
FOKN1_3054	Zn-dependent peptidases	Y	no hit	K07263	0.194%
FOKN1_3055	Zn-dependent peptidases	N	no hit	K07263	0.090%
FOKN1_3056	GTPase	N	no hit	K03110	0.011%
FOKN1_3057	ATPase	N	no hit	K09812	n.d
FOKN1_3058	cell division protein FtsX	N	no hit	K09811	n.d
FOKN1_3059	uncharacterized protein	N	no hit	K02488	n.d
FOKN1_3060	RNA polymerase sigma factor	N	K	K03089	n.d
FOKN1_3061	cbb3-type cytochrome oxidase maturation protein	N	no hit		n.d
FOKN1_3062	uncharacterized protein	N	no hit		n.d
FOKN1_3063	peptide methionine sulfoxide reductase MsrA	N	G	K07304	n.d
FOKN1_3064	mannose-6-phosphate isomerase	N	G		n.d
FOKN1_3065	SAM-dependent methyltransferases	N	no hit		n.d
FOKN1_3066	uncharacterized protein	N	no hit		n.d
FOKN1_3067	RNA-binding proteins	N	no hit		n.d
FOKN1_3068	cold shock proteins	N	no hit	K03704	n.d
FOKN1_3069	carbonic anhydrase	N	P	K01673	n.d
FOKN1_3070	divalent heavy-metal cations transporter	N	D		n.d
FOKN1_3071	uncharacterized protein	Y	D		0.013%
FOKN1_3072	transcriptional regulator	N	T	K02481	0.016%
FOKN1_3073	signal transduction histidine kinase	N	no hit	K07710	n.d
FOKN1_3074	carbamoylphosphate synthase small subunit	N	no hit		n.d
