# Supplementary material for: Thiocyanate Degradation by a Highly Enriched Culture of the Neutrophilic Halophile Thiohalobacter sp. Strain FOKN1 from Activated Sludge and Genomic Insights into Thiocyanate Metabolism
Source: Microbes Environ. 2019 Dec 27;34(4):402–12. doi: 10.1264/jsme2.ME19068 (PMC6934394; doi:10.1264/jsme2.ME19068)
Supplement: Supplementary file 3 [file 34_402_s3.pdf]

Table S4. *Thiohalobacter sp.* strain FOKN1 proteins identified on the basis of two or more peptides. emPAI; exponentially modified protein abundance index, PCI; protein content index.

Locus_tag	Product	Molecular weight (kDa)	pI	Sum of mascot score	% of sequence coverage	Total no. of accepted peptides	No. of unique peptides	Sum of emPAI	PCI (%)
FOKN1_0005	rhodanese-related sulfurtransferase	50.9	4.16	118	8.4	6	4	0.42	0.020%
FOKN1_0011	sirA family protein	11.0	5.43	44	8.1	2	2	1.78	0.086%
FOKN1_0012	alkyl hydroperoxide reductase	22.4	5	508	24.4	15	6	2.82	0.136%
FOKN1_0016	phage-related protein, tail component	19.5	9.04	370	11.5	16	4	2.79	0.134%
FOKN1_0017	uncharacterized protein	17.1	5.09	34	10.8	2	2	0.57	0.027%
FOKN1_0022	cbb3-type cytochrome c oxidase subunit	33.9	4.52	276	27.4	18	10	3.29	0.158%
FOKN1_0024	cytochrome c oxidase cbb3-type subunit II	28.5	5.16	113	15	5	4	1.16	0.056%
FOKN1_0036	malonyl-[acyl-carrier protein] O-methyltransferase	33.1	9.25	40	3.1	2	2	0.26	0.012%
FOKN1_0048	endoribonuclease	13.6	4.46	85	23.8	2	2	0.64	0.031%
FOKN1_0051	guanylate kinase	22.8	5.4	230	17.9	6	4	1.38	0.066%
FOKN1_0052	uncharacterized protein	32.5	4.99	241	21.2	11	7	1.88	0.090%
FOKN1_0055	ribonuclease PH	26.2	5.78	107	5.9	3	2	0.48	0.023%
FOKN1_0064	Zn-dependent protease	53.9	6.08	50	4.1	2	2	0.16	0.008%
FOKN1_0065	malate dehydrogenase	45.8	5	47	5	2	2	0.19	0.009%
FOKN1_0067	membrane carboxypeptidase/penicillin-binding protein	87.8	5.51	109	3	4	4	0.20	0.010%
FOKN1_0068	type IV pilus assembly protein PilM	38.9	4.69	568	34.7	16	15	2.95	0.142%
FOKN1_0069	type IV pilus assembly protein PilN	21.7	6.85	288	25.5	10	6	2.60	0.125%
FOKN1_0070	pilus assembly protein PilO	23.3	4.83	172	18.5	7	5	1.71	0.082%
FOKN1_0071	pilus assembly protein PilP	20.0	4.95	198	40.5	10	7	2.63	0.126%
FOKN1_0072	type IV pilus assembly protein PilQ	78.0	5	506	23.5	17	15	1.92	0.092%

Table S4. *Thiohalobacter sp.* strain FOKN1 proteins identified on the basis of two or more peptides. emPAI; exponentially modified protein abundance index, PCI; protein content index.

Locus_tag	Product	Molecular weight (kDa)	pI	Sum of mascot score	% of sequence coverage	Total no. of accepted peptides	No. of unique peptides	Sum of emPAI	PCI (%)
FOKN1_0074	3-dehydroquinate synthase	38.8	5.28	114	9.4	3	3	0.35	0.017%
FOKN1_0075	dGTP triphosphohydrolase	44.0	6.33	29	4.2	2	2	0.43	0.021%
FOKN1_0078	glutamate synthase, large subunit	170.0	5.52	766	8.5	23	15	0.58	0.028%
FOKN1_0079	glutamate synthase, small subunit	54.4	5.11	99	3.7	4	3	0.31	0.015%
FOKN1_0080	uroporphyrinogen decarboxylase	39.1	5.32	153	11.6	7	5	0.76	0.037%
FOKN1_0085	NAD/NADP transhydrogenase alpha subunit	40.9	5.04	525	28.9	22	13	3.28	0.158%
FOKN1_0087	NAD/NADP transhydrogenase beta subunit	47.4	5.74	108	5.1	4	2	0.78	0.038%
FOKN1_0090	arginyl-tRNA synthetase	65.8	5.39	581	11.1	21	10	1.13	0.054%
FOKN1_0092	tonB-dependent receptor	75.5	7.21	51	2.3	2	2	0.10	0.005%
FOKN1_0096	arginine decarboxylase	70.3	5.18	148	5.9	6	6	0.37	0.018%
FOKN1_0097	spermidine synthase	32.3	5.16	307	18.2	10	7	1.59	0.076%
FOKN1_0100	TRAP-type mannitol/chloroaromatic compound transporter, periplasmic component	40.2	7.1	218	14.4	10	6	1.08	0.052%
FOKN1_0101	signal transduction protein	106.3	5.52	76	1.5	4	3	0.16	0.008%
FOKN1_0103	DNA helicase II	82.6	5.35	66	2.3	2	2	0.10	0.005%
FOKN1_0107	ABC-type multidrug transporter, ATPase component	99.7	6.21	33	2.5	3	2	0.26	0.013%
FOKN1_0108	secretion protein	35.9	5.45	236	14.5	4	4	0.55	0.026%
FOKN1_0111	xylanase/chitin deacetylase	17.8	4.5	64	14.9	2	2	0.54	0.026%
FOKN1_0113	N-acetyltransferase GCN5	19.3	5.24	57	16.4	3	3	0.82	0.039%
FOKN1_0114	ferredoxin-dependent glutamate synthase	55.7	5.27	362	12	13	8	1.09	0.052%

Table S4. *Thiohalobacter sp.* strain FOKN1 proteins identified on the basis of two or more peptides. emPAI; exponentially modified protein abundance index, PCI; protein content index.

Locus_tag	Product	Molecular weight (kDa)	pI	Sum of mascot score	% of sequence coverage	Total no. of accepted peptides	No. of unique peptides	Sum of emPAI	PCI (%)
FOKN1_0134	acyl-CoA synthetase	96.0	6.18	147	5.5	4	4	0.28	0.013%
FOKN1_0138	cytochrome c, mono- and diheme variants	12.3	4.87	44	13.3	2	2	1.57	0.075%
FOKN1_0139	2-polyprenylphenol hydroxylase and related flavodoxin oxidoreductases	54.8	5.04	258	11	8	6	0.66	0.032%
FOKN1_0144	transcriptional regulator	25.2	6.77	101	8.9	2	2	0.36	0.017%
FOKN1_0145	surface antigen	15.8	5.14	721	38.5	20	6	5.66	0.272%
FOKN1_0146	transporter	39.6	5.19	76	5.5	2	2	0.22	0.011%
FOKN1_0147	cation/multidrug efflux pump	113.4	5.08	70	2.9	4	4	0.25	0.012%
FOKN1_0150	helicase	114.8	5.22	267	9.6	14	13	0.73	0.035%
FOKN1_0158	cytochrome c class I	26.5	4.75	329	20	8	6	1.58	0.076%
FOKN1_0160	ATP-dependent DNA helicase	75.1	6.22	93	2.9	4	3	0.20	0.010%
FOKN1_0172	type II secretory pathway, component PulF	45.5	5.96	36	6.6	2	2	0.39	0.019%
FOKN1_0179	nitrogen regulation protein	54.0	5.22	80	5.7	3	3	0.24	0.012%
FOKN1_0182	glutamine synthetase	52.3	5.52	1423	44.6	66	22	6.96	0.334%
FOKN1_0185	tRNA (5-methylaminomethyl-2-thiouridylate)-methyltransferase	39.5	6.24	122	4.9	4	4	0.40	0.019%
FOKN1_0188	glycyl-tRNA synthetase subunit alpha	36.7	5.14	245	12.1	8	4	0.91	0.044%
FOKN1_0189	glycine-tRNA ligase	76.6	4.84	70	1.4	3	2	0.15	0.007%
FOKN1_0193	DNA gyrase subunit B	90.5	5.61	418	10.7	19	13	1.03	0.050%
FOKN1_0195	DNA polymerase III subunit beta	40.4	4.91	604	26.2	19	12	3.23	0.155%
FOKN1_0211	CMP/dCMP deaminase zinc-binding protein	61.6	8.84	173	11	10	7	0.99	0.048%
FOKN1_0221	ribosomal RNA small subunit methyltransferase G	25.0	6.45	215	22.7	9	6	1.71	0.082%

Table S4. *Thiohalobacter sp.* strain FOKN1 proteins identified on the basis of two or more peptides. emPAI; exponentially modified protein abundance index, PCI; protein content index.

Locus_tag	Product	Molecular weight (kDa)	pI	Sum of mascot score	% of sequence coverage	Total no. of accepted peptides	No. of unique peptides	Sum of emPAI	PCI (%)
FOKN1_0223	parB-like nuclease domain family	33.7	9.42	365	27.2	13	11	3.78	0.182%
FOKN1_0227	ATP synthase subunit B	17.7	5.17	1135	101.9	36	23	33.3	1.600%
FOKN1_0228	ATP synthase subunit delta	19.8	5.69	204	33.7	8	6	2.22	0.107%
FOKN1_0229	F1 sector of membrane-bound ATP synthase, alpha subunit	55.5	5.15	1743	43.9	65	27	7.36	0.354%
FOKN1_0230	H(+)-transporting two-sector ATPase	31.9	6.67	178	8	7	5	1.78	0.086%
FOKN1_0231	F1 sector of membrane-bound ATP synthase, beta subunit	52.4	4.64	1170	26	46	15	4.08	0.196%
FOKN1_0232	ATP synthase F1 subunit epsilon	14.8	5.34	84	9.5	3	2	0.68	0.033%
FOKN1_0235	glucosamine/fructose-6-phosphate aminotransferase	66.6	5.51	365	14.9	13	10	1.01	0.049%
FOKN1_0256	carbohydrate kinase	33.8	4.87	142	8.5	3	3	0.38	0.018%
FOKN1_0257	sucrose-phosphate synthase	83.8	8.75	117	5.2	4	4	0.46	0.022%
FOKN1_0258	sucrose synthase	91.4	5.31	102	4	5	4	0.22	0.011%
FOKN1_0260	NADPH-dependent FMN reductase	23.0	5.15	185	22.5	8	5	2.09	0.100%
FOKN1_0266	uncharacterized protein	72.4	4.78	524	21.6	21	14	1.76	0.085%
FOKN1_0268	pyruvate/2-oxoglutarate dehydrogenase complex	28.9	3.97	131	8.8	5	2	0.78	0.037%
FOKN1_0269	shikimate 5-dehydrogenase	30.3	5.35	105	8.2	3	3	0.43	0.021%
FOKN1_0270	delta-aminolevulinic acid dehydratase	37.4	5.33	367	14.1	8	4	0.98	0.047%
FOKN1_0274	coproporphyrinogen III oxidase	35.0	5.63	134	7.6	4	3	0.50	0.024%
FOKN1_0283	NAD(P)H-dependent glycerol-3-phosphate dehydrogenase	35.1	5.53	106	10.5	4	4	0.56	0.027%
FOKN1_0284	protein-export protein SecB	17.2	4.41	119	12.2	3	2	0.56	0.027%
FOKN1_0286	rhodanese-like protein	15.9	10.07	23	14.1	2	2	1.35	0.065%

Table S4. *Thiohalobacter sp.* strain FOKN1 proteins identified on the basis of two or more peptides. emPAI; exponentially modified protein abundance index, PCI; protein content index.

Locus_tag	Product	Molecular weight (kDa)	pI	Sum of mascot score	% of sequence coverage	Total no. of accepted peptides	No. of unique peptides	Sum of emPAI	PCI (%)
FOKN1_0287	transcriptional regulator	12.0	6.82	101	30.2	5	3	2.46	0.118%
FOKN1_0289	phosphoglycerate mutase	57.6	4.88	341	9.5	12	7	0.89	0.043%
FOKN1_0291	carboxyl-terminal protease	47.4	4.93	566	19	18	8	1.86	0.089%
FOKN1_0294	2-polyprenylphenol 6-hydroxylase	63.0	7.22	65	1.6	2	2	0.26	0.013%
FOKN1_0295	sterol-binding domain protein	22.8	5.07	66	10.3	2	2	0.40	0.019%
FOKN1_0297	ubiquinone/menaquinone biosynthesis methyltransferase	32.3	8.83	120	12.2	5	4	0.70	0.034%
FOKN1_0298	putative permease	68.4	6.75	161	4.3	7	4	0.93	0.045%
FOKN1_0302	ATP-dependent protease HslVU (ClpYQ), ATPase subunit	50.6	5.13	271	15.3	8	7	1.11	0.053%
FOKN1_0303	ATP-dependent protease HslVU (ClpYQ), peptidase subunit	19.6	5.52	67	15.5	3	3	0.81	0.039%
FOKN1_0304	tyrosine recombinase XerC	35.1	10.72	38	4.2	2	2	0.25	0.012%
FOKN1_0306	diaminopimelate epimerase	30.1	5.7	266	13.4	7	4	0.98	0.047%
FOKN1_0308	diaminopimelate decarboxylase	45.8	5.14	69	7.9	4	4	0.41	0.020%
FOKN1_0311	cytochrome c, mono- and diheme variants	38.9	4.93	82	5.7	4	3	0.33	0.016%
FOKN1_0321	argininosuccinate lyase	51.7	5.26	207	13.4	8	6	0.74	0.036%
FOKN1_0323	response regulator receiver protein	27.1	5.84	59	5.3	2	2	0.31	0.015%
FOKN1_0325	porphobilinogen deaminase	33.5	5.57	172	8.4	5	3	0.66	0.032%
FOKN1_0326	uroporphyrinogen-III synthase	28.1	6.85	171	10.6	5	3	0.67	0.032%
FOKN1_0327	uroporphyrin-III C-methyltransferase	43.9	5.04	564	34.3	13	12	1.92	0.092%
FOKN1_0328	protoporphyrinogen IX and coproporphyrinogen III oxidase HemY	45.6	9.31	141	3	3	3	0.59	0.028%
FOKN1_0329	2-polyprenylphenol hydroxylase and related flavodoxin oxidoreductases	41.0	5.1	65	6.1	2	2	0.21	0.010%

Table S4. *Thiohalobacter sp.* strain FOKN1 proteins identified on the basis of two or more peptides. emPAI; exponentially modified protein abundance index, PCI; protein content index.

Locus_tag	Product	Molecular weight (kDa)	pI	Sum of mascot score	% of sequence coverage	Total no. of accepted peptides	No. of unique peptides	Sum of emPAI	PCI (%)
FOKN1_0331	3-octaprenyl-4-hydroxybenzoate carboxylyase	55.9	5.51	95	3.9	3	2	0.22	0.011%
FOKN1_0332	Zn-dependent oligopeptidases	77.8	4.97	721	22.4	24	16	1.70	0.082%
FOKN1_0333	glutathione-disulfide reductase	48.6	5.03	181	8.9	6	5	0.54	0.026%
FOKN1_0337	proline iminopeptidase	36.0	5.47	88	5.3	3	2	0.35	0.017%
FOKN1_0338	uncharacterized protein	22.3	4.69	1237	42.4	53	10	13.7	0.660%
FOKN1_0349	Trk-type K+ transporter	50.0	5.17	189	10.9	5	5	0.48	0.023%
FOKN1_0350	response regulator	51.4	5.24	300	8.1	8	4	0.72	0.035%
FOKN1_0353	tRNA and rRNA cytosine-C5-methylases	48.6	7.18	83	3.4	2	2	0.16	0.008%
FOKN1_0354	methionyl-tRNA formyltransferase	33.5	6.07	176	12.7	3	3	0.42	0.020%
FOKN1_0355	N-formylmethionyl-tRNA deformylase	20.4	5.14	46	15.1	2	2	0.42	0.020%
FOKN1_0356	uncharacterized protein	38.0	4.83	297	18	8	6	1.09	0.052%
FOKN1_0359	DNA topoisomerase I	87.9	5.96	282	9.4	11	8	1.24	0.060%
FOKN1_0364	DNA processing protein A	27.0	4.87	103	7.5	3	2	0.48	0.023%
FOKN1_0365	DNA polymerase I	99.6	5.08	170	4.1	5	5	0.21	0.010%
FOKN1_0367	cytochrome c subfamily	21.5	6.41	381	18.1	12	4	2.59	0.124%
FOKN1_0368	resB family protein	75.5	5.68	59	1.5	2	2	0.22	0.010%
FOKN1_0370	thiol:disulfide interchange protein	23.7	5.83	146	8.7	6	3	0.75	0.036%
FOKN1_0371	acetolactate synthase	60.7	5.87	197	20.6	12	11	1.11	0.053%
FOKN1_0378	Sec-independent protein translocase protein TatB	13.6	5.04	311	37.8	7	5	2.70	0.130%
FOKN1_0381	phosphoribosyl-AMP cyclohydrolase	15.2	4.96	215	19.1	5	3	1.32	0.063%

Table S4. *Thiohalobacter sp.* strain FOKN1 proteins identified on the basis of two or more peptides. emPAI; exponentially modified protein abundance index, PCI; protein content index.

Locus_tag	Product	Molecular weight (kDa)	pI	Sum of mascot score	% of sequence coverage	Total no. of accepted peptides	No. of unique peptides	Sum of emPAI	PCI (%)
FOKN1_0385	imidazoleglycerol-phosphate dehydratase	22.2	5.8	299	18.4	10	5	2.27	0.109%
FOKN1_0386	Crp/Fnr family transcriptional regulator	27.9	6.25	48	2.8	2	2	0.30	0.014%
FOKN1_0387	cbb3-type cytochrome c oxidase subunit I	52.8	9.37	49	5.9	2	2	0.35	0.017%
FOKN1_0390	cytochrome c oxidase, cbb3-type subunit III	34.1	5.3	320	15.9	9	5	1.50	0.072%
FOKN1_0401	rhodanese domain protein	15.2	5.1	139	16.2	4	2	0.94	0.045%
FOKN1_0404	molybdenum cofactor biosynthesis protein B	18.5	5.11	75	10.7	2	2	0.51	0.025%
FOKN1_0416	glutamate dehydrogenase/leucine dehydrogenase	52.1	5.73	88	6.4	3	3	0.24	0.012%
FOKN1_0424	nitrous oxide reductase maturation protein NosR	80.8	5.75	36	4.4	2	2	0.10	0.005%
FOKN1_0425	nitrous-oxide reductase	68.8	5.52	826	17.2	40	15	2.79	0.134%
FOKN1_0435	RNA polymerase sigma-70 subunit RpoD	70.2	4.77	126	4.4	6	4	0.78	0.038%
FOKN1_0436	DNA primase	67.4	6.26	59	3.5	2	2	0.12	0.006%
FOKN1_0438	30S ribosomal protein S21	8.8	11.31	99	31	5	3	6.70	0.322%
FOKN1_0441	dihydroneopterin aldolase	13.9	4.92	82	23.6	3	3	1.27	0.061%
FOKN1_0457	succinate-semialdehyde dehydrogenase	49.4	5.33	570	23.4	21	11	2.25	0.108%
FOKN1_0459	cytochrome cd1 nitrite reductase	63.5	5.74	719	23.9	20	13	1.76	0.085%
FOKN1_0470	Crp/Fnr family transcriptional regulator Dnr	25.2	5.19	181	20.4	9	6	1.92	0.092%
FOKN1_0483	K+ transporter	16.9	5.91	64	9.8	2	2	0.51	0.025%
FOKN1_0492	molybdopterin oxidoreductase family protein	110.8	5.86	244	6	9	8	0.61	0.029%
FOKN1_0494	ribulose-bisphosphate carboxylase	52.8	5.77	1993	44.7	97	31	11.2	0.537%
FOKN1_0495	ribulose-bisphosphate carboxylase	14.0	5.46	535	50.4	15	6	9.46	0.455%

Table S4. *Thiohalobacter sp.* strain FOKN1 proteins identified on the basis of two or more peptides. emPAI; exponentially modified protein abundance index, PCI; protein content index.

Locus_tag	Product	Molecular weight (kDa)	pI	Sum of mascot score	% of sequence coverage	Total no. of accepted peptides	No. of unique peptides	Sum of emPAI	PCI (%)
FOKN1_0496	ATPase	30.1	5.16	319	20.8	11	6	1.78	0.086%
FOKN1_0497	rubisco activation protein CbbO	88.7	5.14	241	6.6	11	7	0.52	0.025%
FOKN1_0503	phosphoribosylaminoimidazole carboxylase ATPase subunit	41.2	5.19	37	10.1	2	2	0.21	0.010%
FOKN1_0504	phosphoribosylaminoimidazole carboxylase catalytic subunit	17.5	5.63	97	13.3	3	3	0.79	0.038%
FOKN1_0505	phosphoribosylaminoimidazole-succinocarboxamide synthase	32.8	4.67	84	5.8	4	2	0.40	0.019%
FOKN1_0507	Cu(I)/Ag(I) efflux system outer membrane protein CusC	46.8	8.82	442	8.7	23	3	1.55	0.075%
FOKN1_0515	copper-translocating P-type ATPase	69.2	4.97	46	2.8	2	2	0.12	0.006%
FOKN1_0517	drug exporter	19.1	7.18	80	37	5	5	1.68	0.081%
FOKN1_0521	copper resistance protein A	68.5	5.86	107	6.5	5	4	0.32	0.015%
FOKN1_0535	uncharacterized protein	31.8	5.01	494	23.5	15	6	1.76	0.085%
FOKN1_0536	uncharacterized protein	41.4	4.73	236	13.5	7	6	0.86	0.041%
FOKN1_0537	heavy metal efflux pump CzcA	113.1	4.94	691	7.2	27	14	1.49	0.071%
FOKN1_0538	multi-sensor signal transduction histidine kinase	97.7	6.31	44	1.5	2	2	0.17	0.008%
FOKN1_0539	two component transcriptional regulator	50.4	5.6	198	7.4	5	4	0.62	0.030%
FOKN1_0540	thiol-disulfide isomerase and thioredoxins	19.7	5.45	1371	32.2	43	6	10.6	0.509%
FOKN1_0541	thiocyanate dehydrogenase	54.2	6.35	9396	40.2	489	34	51.1	2.455%
FOKN1_0542	ATPase	17.3	6.39	409	36.9	16	6	5.58	0.268%
FOKN1_0543	uncharacterized protein	20.6	4.2	385	15.4	9	3	2.15	0.103%
FOKN1_0544	signal transduction protein	31.3	5.02	46	7.3	2	2	0.26	0.012%
FOKN1_0548	aldo/keto reductase	38.3	5.25	136	10.1	4	4	0.50	0.024%

Table S4. *Thiohalobacter sp.* strain FOKN1 proteins identified on the basis of two or more peptides. emPAI; exponentially modified protein abundance index, PCI; protein content index.

Locus_tag	Product	Molecular weight (kDa)	pI	Sum of mascot score	% of sequence coverage	Total no. of accepted peptides	No. of unique peptides	Sum of emPAI	PCI (%)
FOKN1_0550	phosphoribulokinase	32.6	5.64	910	29	30	12	5.11	0.246%
FOKN1_0557	uncharacterized protein	42.3	5.36	120	2.3	4	2	0.40	0.019%
FOKN1_0563	uncharacterized protein	18.6	7.08	79	8.6	2	2	0.46	0.022%
FOKN1_0564	uncharacterized protein	74.3	4.63	1434	21.4	41	16	2.70	0.130%
FOKN1_0573	bifunctional sulfate adenylyltransferase subunit 1/adenylylsulfate kinase	64.7	5.68	265	12.7	11	9	0.79	0.038%
FOKN1_0588	glycosyltransferase	45.6	7.66	40	2.7	2	2	0.18	0.009%
FOKN1_0591	GDP-mannose 4,6-dehydratase	41.7	5.28	169	8.8	5	4	0.54	0.026%
FOKN1_0592	NAD-dependent epimerase/dehydratase	35.0	5.48	124	8.8	4	4	0.52	0.025%
FOKN1_0593	uncharacterized protein	89.5	4.78	238	9.9	10	7	0.45	0.022%
FOKN1_0596	response regulator receiver protein	50.8	5.37	214	3.8	5	3	0.41	0.020%
FOKN1_0598	thiol:disulfide interchange protein DsbD	82.9	4.68	114	2.7	4	4	0.32	0.015%
FOKN1_0601	co-chaperonin GroES	10.6	4.89	225	52.1	8	4	3.53	0.170%
FOKN1_0602	chaperonin GroEL	58.2	4.94	8896	76	380	64	83.1	3.995%
FOKN1_0605	TfP pilus assembly protein, ATPase PilU	43.9	5.3	89	7.4	4	4	0.40	0.019%
FOKN1_0606	glutamate-ammonia-ligase adenylyltransferase	109.8	5.36	50	2.1	3	3	0.11	0.005%
FOKN1_0607	branched-chain amino acid aminotransferase	34.6	5.55	221	12.7	10	6	1.13	0.054%
FOKN1_0619	uncharacterized protein	48.2	4.77	459	21.6	12	9	1.74	0.084%
FOKN1_0622	tonB-dependent receptor	84.0	4.69	2700	29.7	82	25	7.20	0.346%
FOKN1_0624	thiamine biosynthesis protein	71.4	5.38	163	11.1	9	8	0.59	0.028%
FOKN1_0626	polyketide synthase	22.6	9.23	119	19.3	5	4	2.15	0.103%

Table S4. ***Thiohalobacter sp.*** **strain FOKN1 proteins identified on the basis of two or more peptides**. emPAI; exponentially modified protein abundance index, PCI; protein content index.

Locus_tag	Product	Molecular weight (kDa)	pI	Sum of mascot score	% of sequence coverage	Total no. of accepted peptides	No. of unique peptides	Sum of emPAI	PCI (%)
FOKN1_0631	translation elongation factors	19.4	5.82	59	14.2	2	2	0.49	0.024%
FOKN1_0637	transcriptional regulator	22.3	5.76	72	7.1	2	2	0.41	0.020%
FOKN1_0641	uncharacterized protein	18.0	4.76	103	9	2	2	0.48	0.023%
FOKN1_0643	uncharacterized protein	92.2	4.7	42	1.5	2	2	0.08	0.004%
FOKN1_0658	transcriptional regulator/sugar kinase	24.7	5.29	67	7.1	2	2	0.37	0.018%
FOKN1_0660	superoxide dismutase	21.4	5.09	145	20.6	6	5	1.49	0.072%
FOKN1_0661	ATPase	25.2	5.26	84	11.4	3	3	0.53	0.025%
FOKN1_0665	ATPase	53.0	7.13	159	8.3	5	5	0.68	0.033%
FOKN1_0674	diguanylate cyclase/phosphodiesterase	113.0	5.53	26	3.1	2	2	0.07	0.003%
FOKN1_0688	glycine betaine/L-proline ABC transporterATP-binding protein	44.4	5.2	113	10.9	5	5	0.51	0.025%
FOKN1_0690	proline/glycine betaine ABC transporter substrate-binding protein	32.5	4.71	82	4.9	4	2	0.40	0.019%
FOKN1_0692	uncharacterized protein	22.7	5.53	261	18.1	7	4	1.17	0.056%
FOKN1_0694	NADH:flavin oxidoreductase/NADH oxidase	39.2	5.23	298	24.7	9	8	1.45	0.070%
FOKN1_0704	uncharacterized protein	42.6	5.14	86	5.5	2	2	0.43	0.021%
FOKN1_0708	ribulose 1,5-bisphosphate carboxylase, large subunit	52.8	5.68	1822	51.1	82	30	11.1	0.532%
FOKN1_0710	carboxysome shell protein CsoS2	80.1	10.02	861	16.3	32	16	4.02	0.193%
FOKN1_0711	carboxysome shell protein CsoS3	56.8	5.74	67	2.4	2	2	0.30	0.015%
FOKN1_0714	microcompartments protein	10.2	5.23	727	68.7	16	6	11.0	0.528%
FOKN1_0715	microcompartments protein	10.6	5.29	711	89.4	16	7	11.3	0.545%
FOKN1_0722	transcriptional regulator	40.6	9.14	57	2.8	3	3	0.65	0.031%

Table S4. ***Thiohalobacter sp.*** **strain FOKN1 proteins identified on the basis of two or more peptides**. emPAI; exponentially modified protein abundance index, PCI; protein content index.

Locus_tag	Product	Molecular weight (kDa)	pI	Sum of mascot score	% of sequence coverage	Total no. of accepted peptides	No. of unique peptides	Sum of emPAI	PCI (%)
FOKN1_0741	homoserine O-succinyltransferase	42.4	5.26	94	6.8	2	2	0.20	0.010%
FOKN1_0742	ABC-type multidrug transporter, ATPase component	32.6	5.41	121	15.4	4	4	0.61	0.029%
FOKN1_0743	uncharacterized protein	37.9	5.48	73	7.9	3	3	0.36	0.017%
FOKN1_0753	molybdenum cofactor biosynthesis protein MoaC	17.2	5.96	76	15.2	2	2	0.50	0.024%
FOKN1_0757	inorganic pyrophosphatase	68.6	5.26	63	2.8	2	2	0.12	0.006%
FOKN1_0758	6-phosphofructokinase	46.6	9.63	702	24.3	32	13	8.87	0.426%
FOKN1_0762	UDP-N-acetylmuramate:L-alanyl-gamma-D-glutamyl-meso-diaminopimelate ligase	50.1	5.53	87	4.1	4	2	0.25	0.012%
FOKN1_0766	glutamate-1-semialdehyde aminotransferase	45.5	5.32	484	24.9	13	10	1.90	0.091%
FOKN1_0768	phosphomethylpyrimidine kinase	28.5	4.43	80	8.3	3	2	0.51	0.025%
FOKN1_0769	ribulose-5-phosphate 4-epimerase-like epimerase	20.4	5.2	56	12	2	2	0.46	0.022%
FOKN1_0773	type II secretory pathway, component PulJ	21.1	5.04	143	12.8	5	4	1.13	0.054%
FOKN1_0774	type II secretory pathway, component PulK	34.7	4.74	44	6.4	2	2	0.25	0.012%
FOKN1_0776	type II secretory pathway, component PulM	18.0	5.28	157	20.2	4	3	0.90	0.043%
FOKN1_0777	type II secretion system protein N	28.7	10.39	129	14.8	5	5	1.13	0.055%
FOKN1_0779	hydrolase	26.6	5.53	59	7.5	2	2	0.34	0.016%
FOKN1_0781	3'-phosphoadenosine 5'-phosphosulfate (PAPS) 3'-phosphatase	29.9	4.61	169	10.1	6	3	0.68	0.033%
FOKN1_0783	type II secretory pathway, component ExeA	51.6	4.95	63	4.5	2	2	0.16	0.008%
FOKN1_0787	uncharacterized protein	20.4	5.11	88	5.9	2	2	0.42	0.020%
FOKN1_0789	long-chain fatty acid transport protein	50.0	4.9	58	3.5	3	2	0.54	0.026%

Table S4. *Thiohalobacter sp.* strain FOKN1 proteins identified on the basis of two or more peptides. emPAI; exponentially modified protein abundance index, PCI; protein content index.

Locus_tag	Product	Molecular weight (kDa)	pI	Sum of mascot score	% of sequence coverage	Total no. of accepted peptides	No. of unique peptides	Sum of emPAI	PCI (%)
FOKN1_0792	methyltransferase	66.2	5.45	52	3.1	2	2	0.13	0.006%
FOKN1_0794	O-linked N-acetylglucosamine transferase	84.8	6.04	117	2.9	3	3	0.15	0.007%
FOKN1_0801	acetyl-CoA carboxylase, biotin carboxylase	49.2	5.7	394	17.9	15	8	1.22	0.059%
FOKN1_0806	phosphoribosyl aminoimidazole carboxamide formyltransferase/IMP cyclohydrolase	56.0	5.1	545	22.5	17	9	1.47	0.071%
FOKN1_0807	phosphoribosylamine-glycine ligase	44.9	5.07	231	15.9	8	6	0.94	0.045%
FOKN1_0808	transcription termination factor	46.8	6.25	1038	39.9	39	19	5.45	0.262%
FOKN1_0810	DNA/RNA helicase	48.5	7.81	257	10.3	6	5	0.92	0.044%
FOKN1_0811	general secretion pathway protein C	31.9	6.22	226	28.8	8	8	1.66	0.080%
FOKN1_0812	general secretion pathway protein D	71.1	4.87	631	20.2	21	14	1.57	0.076%
FOKN1_0813	general secretory pathway protein E	55.9	5.32	370	14.1	16	8	1.43	0.069%
FOKN1_0816	uncharacterized protein	83.7	4.12	100	1.7	4	2	0.20	0.010%
FOKN1_0819	N-acetylglutamate synthase	49.0	8.64	93	2.3	3	2	0.52	0.025%
FOKN1_0820	acetylornithine deacetylase	42.4	5.09	81	5.2	2	2	0.20	0.010%
FOKN1_0824	dihydroxyacid dehydratase	60.2	5.05	339	9.3	13	5	0.87	0.042%
FOKN1_0825	uncharacterized protein	24.1	9.25	656	35.5	17	9	4.99	0.240%
FOKN1_0830	exodeoxyribonuclease III	32.7	5.66	86	6.3	4	3	0.54	0.026%
FOKN1_0831	signal transduction protein	52.2	5.83	131	8.3	4	4	0.35	0.017%
FOKN1_0835	uncharacterized protein	19.2	4.74	320	31.3	14	7	3.51	0.169%
FOKN1_0842	outer membrane receptor	80.0	5.19	26	2.2	2	2	0.10	0.005%

Table S4. *Thiohalobacter sp.* strain FOKN1 proteins identified on the basis of two or more peptides. emPAI; exponentially modified protein abundance index, PCI; protein content index.

Locus_tag	Product	Molecular weight (kDa)	pI	Sum of mascot score	% of sequence coverage	Total no. of accepted peptides	No. of unique peptides	Sum of emPAI	PCI (%)
FOKN1_0849	transcriptional regulator	16.8	9.13	70	14.4	2	2	0.58	0.028%
FOKN1_0852	fructose-1,6-bisphosphatase	37.9	4.82	295	10.2	8	5	0.96	0.046%
FOKN1_0859	pyruvate/2-oxoglutarate dehydrogenase complex	11.1	6.26	218	22.3	9	3	3.53	0.170%
FOKN1_0864	ferric iron ABC transporter, iron-binding protein	37.5	5.36	1243	29.8	39	10	4.89	0.235%
FOKN1_0888	adenosylhomocysteinase	45.7	5.41	203	14.6	6	6	0.68	0.033%
FOKN1_0889	4-hydroxy-3-methylbut-2-en-1-yl diphosphatesynthase	41.3	5.18	100	6.1	3	2	0.31	0.015%
FOKN1_0890	transcriptional regulator	16.1	6.08	165	28.1	7	4	2.28	0.110%
FOKN1_0894	uncharacterized protein	21.7	5.15	67	11.3	2	2	0.43	0.021%
FOKN1_0900	50S ribosomal protein L21	11.4	9.94	99	35.9	5	4	5.93	0.285%
FOKN1_0901	50S ribosomal protein L27	9.1	11.01	259	65.1	8	5	9.22	0.443%
FOKN1_0903	glutamate 5-kinase	39.9	5.97	99	4.8	2	2	0.22	0.011%
FOKN1_0904	ribosomal protein S20	9.8	12.08	126	12.4	3	2	3.07	0.147%
FOKN1_0905	uncharacterized protein	56.6	10.2	35	3.7	2	2	0.14	0.007%
FOKN1_0907	isoleucyl-tRNA synthetase	107.9	5.72	565	9.7	20	13	0.82	0.040%
FOKN1_0910	hydroxymethylbutenyl pyrophosphate reductase	34.5	5.29	200	10	7	5	0.77	0.037%
FOKN1_0913	TfP pilus assembly protein, tip-associated adhesin PilY1	123.8	4.41	308	4.5	10	7	0.34	0.016%
FOKN1_0917	TfP pilus assembly protein FimT	17.9	5.65	163	13.3	3	2	0.78	0.037%
FOKN1_0923	glycine/D-amino acid oxidases	40.2	5.31	65	6.2	4	2	0.87	0.042%
FOKN1_0926	hydantoinase/carbamoylase family amidase	45.5	5.13	30	2.7	2	2	0.18	0.009%
FOKN1_0929	aspartate carbamoyltransferase	39.8	4.83	159	5.9	3	3	0.32	0.015%

Table S4. *Thiohalobacter sp.* strain FOKN1 proteins identified on the basis of two or more peptides. emPAI; exponentially modified protein abundance index, PCI; protein content index.

Locus_tag	Product	Molecular weight (kDa)	pI	Sum of mascot score	% of sequence coverage	Total no. of accepted peptides	No. of unique peptides	Sum of emPAI	PCI (%)
FOKN1_0930	asparagine synthase	76.3	5.3	791	19.3	30	15	2.06	0.099%
FOKN1_0932	uncharacterized protein	11.7	7.82	445	81.8	14	12	64.7	3.110%
FOKN1_0946	putative signal transduction protein	30.4	5.37	93	13.7	4	4	0.67	0.032%
FOKN1_0947	exonuclease	51.9	8.21	255	7.8	11	5	1.85	0.089%
FOKN1_0948	nicotinate-nucleotide pyrophosphorylase	30.2	5.7	82	3.6	2	2	0.28	0.013%
FOKN1_0955	DNA-binding response regulator PhoB	25.9	5.61	81	7	4	4	0.67	0.032%
FOKN1_0956	histidine kinase	49.3	7.75	46	2.3	2	2	0.35	0.017%
FOKN1_0959	exopolyphosphatase	56.2	8.56	46	3.8	2	2	0.33	0.016%
FOKN1_0960	uncharacterized protein	22.3	5.6	46	9.1	2	2	0.83	0.040%
FOKN1_0961	uncharacterized protein	29.4	4.98	27	6.9	2	2	0.30	0.014%
FOKN1_0967	NADH:ubiquinone oxidoreductase, subunit RnfG	24.3	5.84	75	14.2	3	3	0.61	0.029%
FOKN1_0972	methionyl-tRNA synthetase	77.4	5.41	187	8.8	8	6	0.47	0.023%
FOKN1_0973	ATPase	38.9	4.94	159	12.4	8	5	1.00	0.048%
FOKN1_0976	phosphoenolpyruvate carboxylase	108.7	6.52	240	4.4	9	5	0.70	0.033%
FOKN1_0977	glycogen/starch synthase	53.8	6.21	58	3.3	2	2	0.16	0.008%
FOKN1_0978	6-phosphogluconate dehydrogenase-like protein	32.2	5.13	118	21.9	5	5	1.01	0.048%
FOKN1_0979	glucose-6-phosphate 1-dehydrogenase	56.4	5.6	224	9.9	9	5	0.72	0.035%
FOKN1_0980	glucose-6-phosphate isomerase	61.3	5.53	185	10.2	5	5	0.38	0.018%
FOKN1_0983	phosphate starvation-inducible protein PhoH, ATPase	36.2	5.7	114	8.3	3	3	0.38	0.018%
FOKN1_0985	Mg2+ and Co2+ transporter	33.0	4.74	66	6.8	2	2	0.27	0.013%

Table S4. *Thiohalobacter sp.* strain FOKN1 proteins identified on the basis of two or more peptides. emPAI; exponentially modified protein abundance index, PCI; protein content index.

Locus_tag	Product	Molecular weight (kDa)	pI	Sum of mascot score	% of sequence coverage	Total no. of accepted peptides	No. of unique peptides	Sum of emPAI	PCI (%)
FOKN1_0988	uncharacterized protein	22.2	5.17	103	12.9	3	3	0.69	0.033%
FOKN1_0989	leucyl-tRNA synthetase	99.6	5.32	139	6.3	7	7	0.31	0.015%
FOKN1_0990	uncharacterized protein	18.8	5.46	101	20.1	5	4	1.49	0.072%
FOKN1_0992	gamma-glutamyl phosphate reductase	45.9	5.27	137	4.7	4	4	0.37	0.018%
FOKN1_0997	ribonuclease G	54.6	5.07	231	10.6	8	5	0.59	0.028%
FOKN1_1000	nitrilase/cyanide hydratase and apolipoprotein N-acyltransferase	30.8	5.5	144	13.9	7	4	1.12	0.054%
FOKN1_1001	Zn-dependent proteases	50.6	4.93	320	11.6	12	8	1.06	0.051%
FOKN1_1010	Zn-dependent proteases	49.3	5.12	266	14.5	9	7	0.82	0.039%
FOKN1_1014	uncharacterized protein	18.7	5.04	123	7.1	3	3	0.69	0.033%
FOKN1_1016	phosphoenolpyruvate-protein phosphotransferase	65.1	4.98	87	2.1	2	2	0.12	0.006%
FOKN1_1023	RNA polymerase sigma-54 subunit RpoN	55.1	4.64	111	5.7	4	3	0.24	0.012%
FOKN1_1027	3-deoxy-D-manno-octulosonate 8-phosphatephosphatase	20.6	5.41	210	19.4	6	5	1.76	0.085%
FOKN1_1028	arabinose 5-phosphate isomerase	34.3	5.49	148	17	5	5	0.77	0.037%
FOKN1_1032	ABC-type transporter, ATPase component	30.5	5.72	156	15.9	6	5	1.28	0.061%
FOKN1_1034	ABC-type transporter, periplasmic component	16.9	4.19	37	12	2	2	0.57	0.027%
FOKN1_1037	UDP-N-acetylglucosamine1-carboxyvinyltransferase	45.5	5.22	170	8.4	7	5	0.59	0.028%
FOKN1_1038	ATP phosphoribosyltransferase catalytic subunit	23.3	8.63	96	22.5	6	5	1.33	0.064%
FOKN1_1039	histidinol dehydrogenase	46.3	4.83	134	5.3	3	2	0.28	0.013%
FOKN1_1040	histidinol-phosphate aminotransferase	39.6	4.74	70	6.6	2	2	0.22	0.011%
FOKN1_1044	uncharacterized protein	38.3	5.72	100	13.4	6	5	0.67	0.032%

Table S4. *Thiohalobacter sp.* strain FOKN1 proteins identified on the basis of two or more peptides. emPAI; exponentially modified protein abundance index, PCI; protein content index.

Locus_tag	Product	Molecular weight (kDa)	pI	Sum of mascot score	% of sequence coverage	Total no. of accepted peptides	No. of unique peptides	Sum of emPAI	PCI (%)
FOKN1_1049	multimeric flavodoxin	21.4	6.3	164	17.6	5	3	1.15	0.055%
FOKN1_1052	uncharacterized protein	27.1	5.36	2426	47.8	78	22	25.9	1.243%
FOKN1_1053	uncharacterized protein	60.8	6.54	128	7	4	4	0.30	0.014%
FOKN1_1056	uncharacterized protein	50.5	4.9	204	15.1	7	7	1.00	0.048%
FOKN1_1057	uncharacterized protein	26.2	6.02	53	10	2	2	0.35	0.017%
FOKN1_1059	L-aspartate oxidase	61.0	6.32	100	4.8	3	3	0.21	0.010%
FOKN1_1062	negative regulatory protein	37.2	6.03	464	10.5	18	6	2.00	0.096%
FOKN1_1064	trypsin-like serine proteases	51.1	5.04	476	12.1	19	8	1.70	0.082%
FOKN1_1068	uncharacterized protein	14.3	9.45	277	36.3	9	5	4.10	0.197%
FOKN1_1069	ribonuclease III	25.4	5.89	69	12	3	3	0.58	0.028%
FOKN1_1072	pyridoxal phosphate biosynthesis protein	26.9	5.82	104	5.7	2	2	0.30	0.014%
FOKN1_1077	cysteine synthase B	32.0	5.04	326	17.6	12	6	2.16	0.104%
FOKN1_1080	3'-5' exonuclease	29.1	5.46	296	21.3	9	6	1.54	0.074%
FOKN1_1094	biopolymer transport proteins	25.1	5.85	226	10.1	6	2	0.76	0.037%
FOKN1_1095	biopolymer transport protein	15.3	4.96	81	15.3	2	2	0.65	0.031%
FOKN1_1096	uncharacterized protein	38.8	8.54	133	8	5	4	0.58	0.028%
FOKN1_1097	biopolymer transport protein	47.8	6.12	681	45.1	25	20	4.11	0.197%
FOKN1_1098	peptidoglycan-associated outer membrane lipoprotein	19.5	4.86	1993	92.8	57	19	41.4	1.992%
FOKN1_1101	uncharacterized protein	24.5	6.2	167	9.2	4	2	0.54	0.026%
FOKN1_1121	DNA-binding ferritin-like protein	17.9	6.08	623	41.7	17	6	4.31	0.207%

Table S4. *Thiohalobacter sp.* strain FOKN1 proteins identified on the basis of two or more peptides. emPAI; exponentially modified protein abundance index, PCI; protein content index.

Locus_tag	Product	Molecular weight (kDa)	pI	Sum of mascot score	% of sequence coverage	Total no. of accepted peptides	No. of unique peptides	Sum of emPAI	PCI (%)
FOKN1_1123	putative heme degradation protein	36.1	6.3	203	11.7	5	3	0.49	0.024%
FOKN1_1125	acyl-CoA dehydrogenases	25.0	4.15	280	19.2	8	2	1.04	0.050%
FOKN1_1126	uncharacterized protein	77.5	3.91	123	3.1	3	2	0.16	0.008%
FOKN1_1127	tonB-denpendent receptor	73.5	5.09	845	27.7	33	19	2.91	0.140%
FOKN1_1128	glycosyltransferases	32.5	5.13	63	7.5	2	2	0.27	0.013%
FOKN1_1129	pyridoxamine 5'-phosphate oxidase-related FMN-binding protein	22.4	5.39	708	41.7	23	9	5.72	0.275%
FOKN1_1130	ABC-type hemin transporter, periplasmic component	30.9	6.13	343	20.6	13	6	2.19	0.105%
FOKN1_1132	iron-chelate-transporting ATPase	30.2	9.71	50	7.8	2	2	0.29	0.014%
FOKN1_1133	putative heme iron utilization protein	11.8	5.65	82	20.6	4	2	1.97	0.095%
FOKN1_1135	ribonucleoside-diphosphate reductase, beta subunit	45.7	5.27	77	3	2	2	0.18	0.009%
FOKN1_1136	ribonucleoside-diphosphate reductase alpha subunit	106.4	5.58	3680	44.9	129	48	7.53	0.362%
FOKN1_1138	methionine synthase	87.1	5.63	1554	29.9	62	29	6.08	0.292%
FOKN1_1141	uncharacterized protein	87.9	4.85	346	10.5	13	10	0.80	0.039%
FOKN1_1148	5'-nucleotidase/2',3'-cyclic phosphodiesterase	64.6	5.03	813	18.2	24	10	1.60	0.077%
FOKN1_1149	diheme cytochrome SoxA	30.1	6.12	1152	29.6	45	10	7.05	0.339%
FOKN1_1150	sulfur oxidation protein SoxZ	11.6	9.33	3337	91.4	119	14	148	7.121%
FOKN1_1151	sulfur oxidation protein SoxY-like protein	14.2	5.11	1526	41	38	7	17.5	0.839%
FOKN1_1155	redox protein	8.2	6.06	148	35.5	8	4	9.88	0.475%
FOKN1_1156	long-chain fatty acid transporter	49.3	4.49	62	1.7	2	2	0.35	0.017%
FOKN1_1157	long-chain fatty acid transporter	50.5	4.63	42	3.5	3	3	0.57	0.027%

Table S4. ***Thiohalobacter sp.*** **strain FOKN1 proteins identified on the basis of two or more peptides**. emPAI; exponentially modified protein abundance index, PCI; protein content index.

Locus_tag	Product	Molecular weight (kDa)	pI	Sum of mascot score	% of sequence coverage	Total no. of accepted peptides	No. of unique peptides	Sum of emPAI	PCI (%)
FOKN1_1158	uncharacterized protein	33.4	5.12	376	19.9	12	6	1.86	0.089%
FOKN1_1161	uncharacterized protein	37.7	5.54	198	12.3	5	4	0.62	0.030%
FOKN1_1163	transcriptional regulator	34.6	5.67	128	8.6	5	3	0.65	0.031%
FOKN1_1166	ATPase	52.6	4.96	111	9.6	6	6	0.54	0.026%
FOKN1_1167	peroxiredoxin	18.1	8.69	245	43.5	10	7	3.66	0.176%
FOKN1_1169	galactose-1-phosphate uridyl transferase	41.2	5.78	218	9.1	6	4	0.71	0.034%
FOKN1_1170	dihydrodipicolinate synthase	31.8	5.1	94	11	4	3	0.63	0.030%
FOKN1_1171	uncharacterized protein	43.9	4.96	558	33.8	20	13	3.06	0.147%
FOKN1_1173	phosphoribosylformylglycinamidine synthase	142.0	5.33	503	7.2	16	10	0.48	0.023%
FOKN1_1178	transcriptional regulator	31.4	5.65	156	14.9	4	4	0.64	0.031%
FOKN1_1179	ATPase components of ABC transporters	61.9	5.23	202	15.8	9	7	0.84	0.040%
FOKN1_1181	glycine/serine hydroxymethyltransferase	45.6	5.46	904	29.4	34	16	4.84	0.233%
FOKN1_1182	transcriptional regulator	19.6	5.87	114	10.8	3	2	0.70	0.034%
FOKN1_1185	3,4-dihydroxy-2-butanone 4-phosphate synthase	41.4	4.83	134	8.3	4	3	0.43	0.021%
FOKN1_1187	transcription termination factor	18.4	6.98	108	12	3	3	0.75	0.036%
FOKN1_1191	GTP cyclohydrolase I	31.4	5.31	282	22.5	9	8	1.70	0.082%
FOKN1_1192	6-pyruvoyl-tetrahydropterin synthase	14.8	5.58	72	15	2	2	0.68	0.033%
FOKN1_1201	catalase/hydroperoxidase	82.3	4.91	477	9.2	14	7	0.77	0.037%
FOKN1_1206	DNA topoisomerase IV subunit B	69.7	6.25	156	8	7	6	0.70	0.034%
FOKN1_1207	DNA topoisomerase IV subunit A	83.8	6.53	229	10.4	10	7	0.80	0.038%

Table S4. *Thiohalobacter sp.* strain FOKN1 proteins identified on the basis of two or more peptides. emPAI; exponentially modified protein abundance index, PCI; protein content index.

Locus_tag	Product	Molecular weight (kDa)	pI	Sum of mascot score	% of sequence coverage	Total no. of accepted peptides	No. of unique peptides	Sum of emPAI	PCI (%)
FOKN1_1213	Zn-dependent protease	32.1	10.27	197	6.8	4	2	0.40	0.019%
FOKN1_1214	uncharacterized protein	46.3	4.86	138	5.1	3	3	0.27	0.013%
FOKN1_1215	putative diguanylate phosphodiesterase	78.5	4.68	677	18.7	28	18	1.87	0.090%
FOKN1_1216	acetyltransferase	18.5	4.98	48	11	2	2	0.52	0.025%
FOKN1_1229	nucleoside-diphosphate kinase	15.9	5.08	126	19.6	3	2	0.62	0.030%
FOKN1_1231	type IV pilus biogenesis protein PilF	28.0	5.64	47	9.7	3	3	0.52	0.025%
FOKN1_1233	histidyl-tRNA synthetase	46.5	5.26	138	10.1	4	4	0.40	0.019%
FOKN1_1234	uncharacterized protein	23.5	4.92	216	18.3	7	5	1.50	0.072%
FOKN1_1235	uncharacterized protein	41.3	4.75	186	18.3	7	6	0.77	0.037%
FOKN1_1236	GTPase	52.1	9.63	51	3.2	2	2	0.16	0.008%
FOKN1_1239	ABC-type transporter, ATPase component	24.3	5.5	72	11.8	2	2	0.38	0.018%
FOKN1_1241	pyruvate:ferredoxin oxidoreductase	132.4	5.42	345	5.2	12	8	0.36	0.017%
FOKN1_1242	dihydroorotate dehydrogenase	37.2	4.76	128	8.3	3	3	0.37	0.018%
FOKN1_1246	IMP dehydrogenase/GMP reductase	52.1	5.82	1007	29.4	29	13	3.56	0.171%
FOKN1_1247	GMP synthase	58.9	5.73	269	13.1	11	7	0.91	0.044%
FOKN1_1254	uncharacterized protein	18.2	5.58	88	11.9	5	4	1.35	0.065%
FOKN1_1261	uncharacterized protein	22.8	6.05	98	17.8	5	5	1.16	0.056%
FOKN1_1263	transcriptional regulator	32.6	5.8	60	6.9	2	2	0.27	0.013%
FOKN1_1269	7-keto-8-aminopelargonate synthetase	43.5	5.47	558	19.3	19	9	2.24	0.108%
FOKN1_1275	lauroyl/myristoyl acyltransferase	36.5	10.61	45	4.1	2	2	0.48	0.023%

Table S4. *Thiohalobacter sp.* strain FOKN1 proteins identified on the basis of two or more peptides. emPAI; exponentially modified protein abundance index, PCI; protein content index.

Locus_tag	Product	Molecular weight (kDa)	pI	Sum of mascot score	% of sequence coverage	Total no. of accepted peptides	No. of unique peptides	Sum of emPAI	PCI (%)
FOKN1_1283	uncharacterized protein	37.4	7.18	305	14.2	11	5	1.59	0.076%
FOKN1_1289	putative sulfotransferase protein	37.7	8.73	59	7.8	3	3	0.80	0.039%
FOKN1_1290	uncharacterized protein	37.0	6.4	88	9.3	4	4	0.48	0.023%
FOKN1_1294	biopolymer transport proteins	26.4	5.67	123	15.5	4	4	0.80	0.038%
FOKN1_1295	tonB-dependent receptor protein	78.5	4.49	3305	38.5	152	34	10.9	0.523%
FOKN1_1297	putative sulfotransferase protein	38.0	9.08	62	7.4	3	3	0.78	0.038%
FOKN1_1300	uncharacterized protein	28.2	6.53	190	12.1	5	3	0.83	0.040%
FOKN1_1311	Zn-dependent hydrolases	34.5	4.91	61	5.8	2	2	0.25	0.012%
FOKN1_1317	uncharacterized protein	48.9	5.59	35	4.8	2	2	0.17	0.008%
FOKN1_1324	uncharacterized protein	34.2	4.49	173	12.3	3	3	0.41	0.020%
FOKN1_1325	CRISPR-associated protein	41.8	5.71	553	41.6	19	17	3.54	0.170%
FOKN1_1335	exonuclease III	46.3	5.05	72	4.7	2	2	0.18	0.009%
FOKN1_1344	glycerol uptake facilitator and related permeases	31.9	6.27	80	6.6	2	2	0.28	0.013%
FOKN1_1348	DNA recombination protein rmuC	62.2	5.23	105	6	3	3	0.21	0.010%
FOKN1_1351	5'-nucleotidase	35.1	4.99	98	5.7	3	3	0.37	0.018%
FOKN1_1352	uncharacterized protein	38.9	5.56	74	5.7	2	2	0.22	0.011%
FOKN1_1363	3-oxoacyl-(acyl-carrier-protein) synthase	15.2	9.83	33	11.5	2	2	0.65	0.031%
FOKN1_1376	transcriptional regulators	22.3	4.81	117	9.1	5	2	2.07	0.099%
FOKN1_1399	integrase	46.5	9.46	31	4.3	2	2	0.39	0.019%
FOKN1_1403	20S proteasome subunits A/B	27.7	7.05	190	16.7	6	5	1.07	0.051%

Table S4. *Thiohalobacter sp.* strain FOKN1 proteins identified on the basis of two or more peptides. emPAI; exponentially modified protein abundance index, PCI; protein content index.

Locus_tag	Product	Molecular weight (kDa)	pI	Sum of mascot score	% of sequence coverage	Total no. of accepted peptides	No. of unique peptides	Sum of emPAI	PCI (%)
FOKN1_1404	uncharacterized protein	54.9	5.41	101	4.3	4	2	0.31	0.015%
FOKN1_1405	uncharacterized protein	36.2	9.41	131	4.9	4	2	1.02	0.049%
FOKN1_1410	transcription elongation factor	19.0	8.8	41	12	2	2	0.46	0.022%
FOKN1_1416	ferredoxin-NADP reductase	28.2	5.38	93	9.7	3	3	0.51	0.025%
FOKN1_1438	guanosine polyphosphate pyrophosphohydrolases/synthetases	83.5	6.5	50	2	2	2	0.10	0.005%
FOKN1_1458	flagellar rod assembly protein/muramidase FlgJ	39.0	5.85	43	2.5	2	2	0.22	0.011%
FOKN1_1461	signal transduction protein	96.5	5.07	40	3.1	2	2	0.17	0.008%
FOKN1_1462	flagellin	48.7	3.96	398	11.6	6	4	0.56	0.027%
FOKN1_1468	response regulator	50.9	5.78	451	25.6	18	11	1.60	0.077%
FOKN1_1469	signal transduction histidine kinase	42.9	5.29	38	4	2	2	0.20	0.010%
FOKN1_1470	flagellar regulatory protein FleQ	50.2	5.39	54	5.9	3	2	0.24	0.012%
FOKN1_1504	chemotaxis signal transduction protein	17.5	4.25	105	11.4	3	2	0.80	0.038%
FOKN1_1510	uncharacterized protein	14.2	10.81	52	16	3	2	0.93	0.045%
FOKN1_1516	pyruvate formate lyase-activating enzyme PflA	42.1	5.6	31	4.7	2	2	0.20	0.010%
FOKN1_1519	1,4-alpha-glucan-branching protein	83.3	5.62	248	8.1	11	7	0.59	0.028%
FOKN1_1520	ADP-glucose pyrophosphorylase	47.8	5.44	104	6.9	4	3	0.80	0.039%
FOKN1_1521	alpha-amylase/alpha-mannosidase	65.1	4.94	147	5.4	4	3	0.26	0.012%
FOKN1_1523	glucan phosphorylase	97.4	5.85	232	5.7	9	6	0.40	0.019%
FOKN1_1527	preprotein translocase subunit YajC	12.5	6.07	467	55.8	17	6	9.57	0.460%
FOKN1_1528	preprotein translocase subunit SecD	68.2	4.94	72	2.7	2	2	0.12	0.006%

Table S4. *Thiohalobacter sp.* strain FOKN1 proteins identified on the basis of two or more peptides. emPAI; exponentially modified protein abundance index, PCI; protein content index.

Locus_tag	Product	Molecular weight (kDa)	pI	Sum of mascot score	% of sequence coverage	Total no. of accepted peptides	No. of unique peptides	Sum of emPAI	PCI (%)
FOKN1_1533	fructose-1,6-bisphosphatase	29.3	6.72	220	24.8	8	7	1.89	0.091%
FOKN1_1534	phosphatidylserine decarboxylase	33.2	10.69	67	5.1	3	3	0.38	0.018%
FOKN1_1538	coproporphyrinogen III oxidase	52.6	5.23	54	3.9	2	2	0.16	0.008%
FOKN1_1540	chorismate synthase	39.4	6.32	189	9.3	6	4	0.57	0.027%
FOKN1_1543	fructose-bisphosphate aldolase	38.2	5.7	560	22	19	9	3.17	0.152%
FOKN1_1547	3-isopropylmalate dehydratase large subunit	50.2	5.58	83	7.5	3	3	0.25	0.012%
FOKN1_1548	3-isopropylmalate dehydratase small subunit	24.5	4.92	51	9.4	3	2	0.37	0.018%
FOKN1_1549	isocitrate/isopropylmalate dehydrogenase	38.0	4.64	139	10.6	3	3	0.36	0.017%
FOKN1_1550	aspartate-semialdehyde dehydrogenase	37.1	5.35	209	16.2	6	4	0.63	0.030%
FOKN1_1551	TfP pilus assembly protein FimV	87.5	4.21	683	19.8	22	15	1.11	0.053%
FOKN1_1554	uncharacterized protein	29.2	5.02	217	7.5	7	3	0.98	0.047%
FOKN1_1558	tryptophan synthase beta chain	43.7	5.65	283	14.7	6	5	0.66	0.032%
FOKN1_1559	tryptophan synthase alpha chain	29.0	5.12	298	24.7	10	9	2.21	0.106%
FOKN1_1560	acetyl-CoA carboxylase beta subunit	32.1	8.57	79	8.4	4	4	0.57	0.027%
FOKN1_1562	uncharacterized protein	21.3	4.43	54	9.7	3	2	0.44	0.021%
FOKN1_1564	glutamine phosphoribosyl pyrophosphate amidotransferase	57.3	5.91	212	14.3	8	7	0.91	0.044%
FOKN1_1565	cystathionine beta-lyases/cystathionine gamma-synthases	44.4	5.13	280	12.9	7	6	0.74	0.036%
FOKN1_1572	peptidyl-prolyl cis-trans isomerase	23.9	5.65	209	32.6	9	6	1.83	0.088%
FOKN1_1574	cysteinyl-tRNA synthetase	51.9	5.12	312	19.7	12	8	1.08	0.052%
FOKN1_1583	peptidyl-prolyl cis-trans isomerase	48.7	4.62	173	11.5	6	5	0.58	0.028%

Table S4. *Thiohalobacter sp.* strain FOKN1 proteins identified on the basis of two or more peptides. emPAI; exponentially modified protein abundance index, PCI; protein content index.

Locus_tag	Product	Molecular weight (kDa)	pI	Sum of mascot score	% of sequence coverage	Total no. of accepted peptides	No. of unique peptides	Sum of emPAI	PCI (%)
FOKN1_1584	ATP-dependent Clp protease proteolytic subunit	23.5	5.12	180	13.2	10	5	2.10	0.101%
FOKN1_1585	ATP-dependent protease ATP-binding subunit	47.3	4.96	600	21.5	22	12	2.10	0.101%
FOKN1_1586	ATP-dependent Lon protease	91.7	6.17	409	16.4	24	18	2.28	0.109%
FOKN1_1587	DNA-binding transcriptional regulator subunit beta	9.2	9.52	59	23.3	2	2	1.24	0.060%
FOKN1_1590	peptidyl-prolyl cis-trans isomerase	71.2	4.53	450	11.7	18	11	1.19	0.057%
FOKN1_1592	enoyl-[acyl-carrier-protein] reductase	27.8	5.25	789	37.1	23	9	4.19	0.201%
FOKN1_1596	peptide ABC transporter ATPase	74.3	6.7	79	3.4	3	3	0.35	0.017%
FOKN1_1598	serine-pyruvate aminotransferase	43.3	5.68	448	17	16	8	1.98	0.095%
FOKN1_1622	acetyltransferases	19.5	4.99	48	14	3	2	0.48	0.023%
FOKN1_1623	S-adenosylhomocysteine hydrolase	45.2	5.74	87	6	2	2	0.19	0.009%
FOKN1_1626	geranylgeranyl reductase	42.4	7.12	43	2.7	2	2	0.20	0.010%
FOKN1_1629	excinuclease ABC subunit A	208.8	7.57	139	3	6	5	0.26	0.013%
FOKN1_1634	cell division GTPase	51.9	5.99	96	4.5	3	3	0.24	0.012%
FOKN1_1647	thiol-disulfide isomerase and thioredoxins	16.7	9.3	60	15.1	4	2	1.00	0.048%
FOKN1_1653	peroxiredoxin	24.5	5.38	511	25.3	13	5	1.94	0.093%
FOKN1_1658	isocitrate dehydrogenase	81.3	5.23	433	17	18	13	1.00	0.048%
FOKN1_1661	Fe-S oxidoreductase	31.4	9.04	532	3.9	12	2	1.56	0.075%
FOKN1_1662	adenosine phosphosulfate reductase subunit beta	15.9	8.59	1126	68.4	37	10	12.0	0.578%
FOKN1_1663	adenylylsulfate reductase subunit alpha	70.4	5.91	7039	55.6	250	43	22.4	1.075%
FOKN1_1664	ABC-type metal ion transporter, periplasmic component/surface antigen	15.2	5.24	74	21.5	3	3	1.12	0.054%

Table S4. ***Thiohalobacter sp.*** **strain FOKN1 proteins identified on the basis of two or more peptides**. emPAI; exponentially modified protein abundance index, PCI; protein content index.

Locus_tag	Product	Molecular weight (kDa)	pI	Sum of mascot score	% of sequence coverage	Total no. of accepted peptides	No. of unique peptides	Sum of emPAI	PCI (%)
FOKN1_1681	restriction endonuclease	117.6	5.85	75	2.4	4	4	0.30	0.015%
FOKN1_1688	DNA-binding transcriptional dual regulator	16.1	5.44	314	23.9	8	3	2.09	0.100%
FOKN1_1689	DNA repair protein RecN	61.4	5.32	56	6.4	2	2	0.30	0.015%
FOKN1_1693	chaperone Hsp70, co-chaperone with DnaJ	69.6	4.72	1048	28.6	51	23	5.05	0.243%
FOKN1_1695	dihydrodipicolinate reductase	28.4	5.31	218	18.2	7	7	1.61	0.077%
FOKN1_1696	carbamoyl-phosphate synthase, small subunit	41.2	5.17	178	10.3	6	5	0.56	0.027%
FOKN1_1697	carbamoyl-phosphate synthase large subunit	119.4	4.88	1327	19.2	59	25	2.61	0.125%
FOKN1_1702	ATP-dependent metalloprotease FtsH	70.4	5.47	357	21.8	18	14	1.66	0.080%
FOKN1_1705	triose-phosphate isomerase	25.2	4.47	482	24.9	10	5	1.52	0.073%
FOKN1_1709	NADH dehydrogenase subunit B	17.6	9.06	123	21.5	4	3	1.40	0.067%
FOKN1_1710	NADH dehydrogenase subunit C	28.3	4.62	99	8	3	3	0.47	0.023%
FOKN1_1711	NADH dehydrogenase subunit D	48.3	5.56	251	15.8	7	6	0.76	0.037%
FOKN1_1713	NADH:ubiquinone oxidoreductase subunit F	47.8	5.73	105	4.4	4	3	0.36	0.017%
FOKN1_1714	NADH-quinone oxidoreductase subunit G	87.9	5.3	191	11	8	8	0.44	0.021%
FOKN1_1716	NADH:ubiquinone oxidoreductase, subunit I	19.3	7.53	68	6.2	2	2	0.49	0.024%
FOKN1_1725	ABC-type transporter, auxiliary component	24.1	6.92	152	13	5	3	1.24	0.060%
FOKN1_1730	adenylate kinase family protein	23.3	4.86	496	37.6	22	9	4.94	0.237%
FOKN1_1732	uncharacterized protein	134.5	4.99	54	0.8	2	2	0.06	0.003%
FOKN1_1734	lipoprotein	21.6	5.87	295	23.9	9	6	2.51	0.121%
FOKN1_1739	putative transcriptional regulator	33.2	6.39	197	10	6	3	0.69	0.033%

Table S4. ***Thiohalobacter sp.*** **strain FOKN1 proteins identified on the basis of two or more peptides**. emPAI; exponentially modified protein abundance index, PCI; protein content index.

Locus_tag	Product	Molecular weight (kDa)	pI	Sum of mascot score	% of sequence coverage	Total no. of accepted peptides	No. of unique peptides	Sum of emPAI	PCI (%)
FOKN1_1740	dehydrogenase	27.3	6.6	46	4.4	2	2	0.33	0.016%
FOKN1_1741	uncharacterized protein	17.1	6.65	117	27.2	5	4	2.09	0.100%
FOKN1_1745	efflux transporter	39.9	4.97	328	21.4	9	8	1.65	0.079%
FOKN1_1747	transcriptional regulator	22.6	10.24	76	14.4	5	5	2.93	0.141%
FOKN1_1749	transcription elongation factor NusA	55.8	4.31	425	10.8	18	10	1.38	0.066%
FOKN1_1750	translation initiation factor IF-2	92.4	5.51	623	18.6	23	19	2.55	0.122%
FOKN1_1754	polynucleotide phosphorylase/polyadenylase	75.4	5.18	1790	35.4	61	31	5.09	0.245%
FOKN1_1757	response regulator	29.5	4.7	180	14.9	5	4	0.93	0.045%
FOKN1_1758	acyl-coenzyme A synthetases/AMP-fatty acid ligases	72.5	5.18	1707	27.8	62	21	4.22	0.203%
FOKN1_1760	glutamyl- and glutaminyl-tRNA synthetases	33.9	6.25	41	4.3	2	2	0.24	0.012%
FOKN1_1761	RNA polymerase-binding transcription factor DksA	26.0	9.78	103	6.9	4	2	1.11	0.053%
FOKN1_1766	7-cyano-7-deazaguanine reductase	15.1	5.01	107	19.4	3	3	1.13	0.054%
FOKN1_1767	ATPase	133.5	5.28	505	14.1	19	17	0.78	0.037%
FOKN1_1769	NAD-dependent DNA ligase	75.7	6.52	66	5.3	4	3	0.37	0.018%
FOKN1_1770	thymidine phosphorylase	87.5	5.29	83	2.9	3	2	0.09	0.004%
FOKN1_1773	murein lipoprotein	9.7	5.09	1837	45.2	25	5	14.4	0.692%
FOKN1_1781	myosin	55.8	4.09	30	3.1	2	2	0.14	0.007%
FOKN1_1788	biotin synthase	13.0	5.11	76	17.8	2	2	0.79	0.038%
FOKN1_1789	uncharacterized protein	23.2	5.34	105	17.4	5	4	1.13	0.054%
FOKN1_1794	FAD-dependent pyridine nucleotide-disulfideoxidoreductase	46.8	6.09	310	16.6	15	9	1.81	0.087%

Table S4. *Thiohalobacter sp.* strain FOKN1 proteins identified on the basis of two or more peptides. emPAI; exponentially modified protein abundance index, PCI; protein content index.

Locus_tag	Product	Molecular weight (kDa)	pI	Sum of mascot score	% of sequence coverage	Total no. of accepted peptides	No. of unique peptides	Sum of emPAI	PCI (%)
FOKN1_1796	rhodanese-like protein	24.9	7.6	399	19	13	5	2.26	0.109%
FOKN1_1798	3-deoxy-D-arabino-heptulosonate 7-phosphate synthase	42.1	6.05	82	13.1	5	5	0.52	0.025%
FOKN1_1800	nitrilase/cyanide hydratase and apolipoprotein N-acyltransferase	32.7	5.2	112	11.2	3	3	0.43	0.021%
FOKN1_1805	ABC-type antimicrobial peptide transporter, ATPase component	25.3	6.1	83	13	3	3	0.59	0.028%
FOKN1_1808	biopolymer transport proteins	23.2	6.3	275	16.6	9	5	1.84	0.088%
FOKN1_1822	ribonuclease E	93.3	7.66	330	8.5	16	10	1.58	0.076%
FOKN1_1834	3-oxoacyl-ACP synthase	35.2	5.28	127	9	4	4	0.52	0.025%
FOKN1_1836	3-ketoacyl-(acyl-carrier-protein) reductase	25.6	5.64	190	10.9	7	5	1.25	0.060%
FOKN1_1838	3-oxoacyl-[acyl-carrier-protein] synthase II	43.4	5.49	85	8.7	3	3	0.31	0.015%
FOKN1_1843	citrate synthase	49.0	5.44	180	17.9	11	9	0.98	0.047%
FOKN1_1845	cytochrome c5	10.8	5.23	928	24.5	24	3	8.14	0.391%
FOKN1_1847	thymidylate kinase	19.8	4.72	51	17.1	2	2	0.44	0.021%
FOKN1_1850	Mg-dependent DNase	30.2	5.12	83	6.7	2	2	0.29	0.014%
FOKN1_1853	long-chain acyl-CoA synthetases	67.8	5.43	113	2.6	5	4	0.30	0.014%
FOKN1_1854	3-hydroxyacyl-CoA dehydrogenase	74.5	7.74	60	2.5	2	2	0.24	0.011%
FOKN1_1856	acyl-CoA dehydrogenases	86.5	6.21	64	3.2	4	3	0.15	0.007%
FOKN1_1858	selenocysteine lyase	42.3	5.23	110	13.1	4	4	0.45	0.022%
FOKN1_1860	alkyl hydroperoxide reductase	16.6	5.19	152	6	4	2	1.04	0.050%
FOKN1_1862	selenocysteine-specific translation elongation factor	69.9	7.88	47	1.4	2	2	0.26	0.013%
FOKN1_1871	uncharacterized protein	21.3	4.9	62	5.2	2	2	0.40	0.019%

Table S4. *Thiohalobacter sp.* strain FOKN1 proteins identified on the basis of two or more peptides. emPAI; exponentially modified protein abundance index, PCI; protein content index.

Locus_tag	Product	Molecular weight (kDa)	pI	Sum of mascot score	% of sequence coverage	Total no. of accepted peptides	No. of unique peptides	Sum of emPAI	PCI (%)
FOKN1_1877	sulfate adenylyltransferase	44.1	5.3	914	38.9	43	19	5.68	0.273%
FOKN1_1880	pyruvate/2-oxoglutarate dehydrogenase complex, dihydrolipoamide dehydrogenase (E3) component	51.7	5.51	188	9.4	5	5	0.44	0.021%
FOKN1_1882	alpha-ketoglutarate decarboxylase	107.2	5.89	68	1.7	3	3	0.12	0.006%
FOKN1_1888	membrane protease	28.5	5.35	106	12.2	3	3	0.51	0.025%
FOKN1_1896	CDP-diacylglycerol-glycerol-3-phosphate 3-phosphatidyltransferase	21.1	7.85	50	10.9	2	2	0.40	0.019%
FOKN1_1899	response regulator	24.2	5.12	58	7.8	2	2	0.38	0.018%
FOKN1_1916	capsid-like protein	47.6	4.85	34	3	2	2	0.18	0.009%
FOKN1_1931	uncharacterized protein	22.2	4.99	103	13.8	5	3	1.07	0.051%
FOKN1_1941	dissimilatory sulfite reductase (desulfoviridin), gamma subunit	12.9	5.12	43	14	2	2	0.68	0.033%
FOKN1_1942	Fe2+/Pb2+ permease	25.5	5.6	55	7.9	2	2	0.32	0.015%
FOKN1_1945	sulfite reductase alpha subunit	48.1	5.15	1775	46	51	22	7.13	0.343%
FOKN1_1946	sulfite reductase beta subunit	40.9	7.91	1168	46.4	51	19	7.91	0.380%
FOKN1_1947	tRNA 5-methylaminomethyl-2-thiouridine synthase TusD	14.5	5.69	568	60	30	13	17.6	0.846%
FOKN1_1948	sulfur relay protein TusC/DsrF	15.7	4.12	110	16.7	5	3	1.34	0.064%
FOKN1_1949	sulfur relay protein TusB/DsrH	11.1	4.96	101	16.7	3	2	1.37	0.066%
FOKN1_1950	sulfite reductase, dissimilatory-type subunit gamma	12.5	4.92	126	39.1	7	4	3.19	0.153%
FOKN1_1951	nitrate reductase subunit gamma	27.6	10.39	142	7	7	2	1.05	0.050%
FOKN1_1952	Fe-S oxidoreductase	55.2	5.12	294	15.3	15	10	1.37	0.066%
FOKN1_1953	NADPH-dependent glutamate synthase beta chain	73.1	5.05	696	18.3	30	13	2.00	0.096%

Table S4. ***Thiohalobacter sp.*** **strain FOKN1 proteins identified on the basis of two or more peptides**. emPAI; exponentially modified protein abundance index, PCI; protein content index.

Locus_tag	Product	Molecular weight (kDa)	pI	Sum of mascot score	% of sequence coverage	Total no. of accepted peptides	No. of unique peptides	Sum of emPAI	PCI (%)
FOKN1_1955	4Fe-4S ferredoxin	28.4	5.85	228	15.7	10	6	1.80	0.087%
FOKN1_1960	signal transduction histidine kinase	62.0	5.63	179	7.7	6	5	0.67	0.032%
FOKN1_1963	alpha amylase catalytic subunit	76.8	5.6	167	4	3	3	0.16	0.008%
FOKN1_1966	peptidase S24/S26A/S26B	12.9	4.65	49	8	2	2	0.68	0.033%
FOKN1_1967	uroporphyrinogen-III C-methyltransferase /precorrin-2 dehydrogenase	52.0	5.79	243	17.6	10	10	1.06	0.051%
FOKN1_1969	aminopeptidase N	100.6	5.12	246	8.8	10	9	0.46	0.022%
FOKN1_1972	signal transduction histidine kinase	26.2	4.98	39	9.1	2	2	0.32	0.015%
FOKN1_1975	seryl-tRNA synthetase	47.8	5.21	146	8.2	6	4	0.56	0.027%
FOKN1_1987	ATPase and specificity subunit of ClpA-ClpPATP-dependent serine protease	83.7	5.79	454	11	13	9	0.91	0.044%
FOKN1_1990	NADP-dependent isocitrate dehydrogenase	48.3	5.2	211	18.6	9	9	1.08	0.052%
FOKN1_1991	ATP-dependent DNA ligase	32.9	4.57	744	17.4	16	4	2.17	0.104%
FOKN1_1992	NTP pyrophosphohydrolases	17.4	5.07	52	10.5	3	3	0.80	0.038%
FOKN1_1996	aconitate hydratase 1	96.7	4.93	182	9.4	11	10	0.55	0.026%
FOKN1_1997	adenylosuccinate lyase	50.7	5.53	142	13.8	6	6	0.60	0.029%
FOKN1_2012	universal stress protein uspA	33.0	8.53	240	27	9	9	1.71	0.082%
FOKN1_2013	universal stress protein uspA	37.2	5.38	269	12.9	7	5	0.74	0.036%
FOKN1_2014	uncharacterized protein	30.2	6.73	54	6.9	2	2	0.29	0.014%
FOKN1_2022	archaeal/vacuolar-type H+-ATPase subunit B	50.4	5.77	117	6.9	4	3	0.34	0.016%
FOKN1_2028	uncharacterized protein	21.8	5.59	483	28.5	16	6	5.37	0.258%
FOKN1_2033	threonine synthase	41.4	5.73	340	21.4	11	9	1.43	0.069%

Table S4. *Thiohalobacter sp.* strain FOKN1 proteins identified on the basis of two or more peptides. emPAI; exponentially modified protein abundance index, PCI; protein content index.

Locus_tag	Product	Molecular weight (kDa)	pI	Sum of mascot score	% of sequence coverage	Total no. of accepted peptides	No. of unique peptides	Sum of emPAI	PCI (%)
FOKN1_2034	homoserine dehydrogenase	46.8	5.1	586	18	23	10	2.15	0.103%
FOKN1_2056	enolase	45.8	4.75	525	23.9	17	11	2.03	0.098%
FOKN1_2057	3-deoxy-D-manno-octulosonate 8-phosphate synthase	30.5	4.95	309	17.1	13	7	2.38	0.114%
FOKN1_2058	CTP synthetase	61.4	5.4	408	21.2	16	12	1.41	0.068%
FOKN1_2060	acetyl-CoA carboxylase carboxyltransferase subunit alpha	35.9	5.07	281	23.4	9	7	1.51	0.073%
FOKN1_2061	DNA polymerase III subunit alpha	130.7	5.14	265	5.7	9	7	0.30	0.014%
FOKN1_2064	RND family efflux pump membrane fusion protein	45.6	4.79	37	6.9	2	2	0.18	0.009%
FOKN1_2065	RND family efflux pump outer membrane lipoprotein	53.5	5.93	63	2.6	2	2	0.35	0.017%
FOKN1_2069	beta-hydroxyacyl-(ACP) dehydratase FabZ	16.8	6.92	358	23.1	6	3	2.06	0.099%
FOKN1_2071	outer membrane protein OmpH	19.1	5.17	198	18.6	7	3	2.24	0.108%
FOKN1_2072	outer membrane protein assembly factor BamA	85.2	4.62	193	6.6	8	7	0.42	0.020%
FOKN1_2077	ribosome recycling factor	20.9	5.32	86	15.7	4	3	1.09	0.052%
FOKN1_2078	uridylate kinase	25.8	5.35	305	16.1	7	5	1.44	0.069%
FOKN1_2079	translation elongation factor Ts	31.6	4.95	142	13.3	4	4	0.64	0.031%
FOKN1_2080	ribosomal protein S2	27.8	6.55	998	41.1	44	14	9.02	0.433%
FOKN1_2081	methionine aminopeptidase	29.4	6.1	130	9.2	5	3	0.77	0.037%
FOKN1_2083	dissimilatory sulfite reductase (desulfoviridin), gamma subunit	13.5	5.46	19	15.8	2	2	0.75	0.036%
FOKN1_2087	aspartate/tyrosine/aromatic aminotransferase	44.8	5.41	147	8.5	4	3	0.39	0.019%
FOKN1_2088	tetrahydrodipicolinate N-succinyltransferase	29.7	5.15	541	23.1	20	8	3.73	0.179%
FOKN1_2096	translational repressor	70.2	5.41	69	3.3	2	2	0.12	0.006%

Table S4. *Thiohalobacter sp.* strain FOKN1 proteins identified on the basis of two or more peptides. emPAI; exponentially modified protein abundance index, PCI; protein content index.

Locus_tag	Product	Molecular weight (kDa)	pI	Sum of mascot score	% of sequence coverage	Total no. of accepted peptides	No. of unique peptides	Sum of emPAI	PCI (%)
FOKN1_2097	uncharacterized protein	29.5	4.84	160	7.2	4	2	0.60	0.029%
FOKN1_2099	glucose/sorbosone dehydrogenases	40.6	5.87	99	5.4	3	3	0.31	0.015%
FOKN1_2100	streptogramin lyase	38.7	5.69	59	2.9	2	2	0.22	0.011%
FOKN1_2107	succinyl-diaminopimelate desuccinylase	41.0	4.91	71	5.6	2	2	0.21	0.010%
FOKN1_2108	integral membrane protein	33.3	6.01	50	5.7	2	2	0.26	0.012%
FOKN1_2112	sirohydrochlorin cobaltochelatase	13.1	4.7	164	26.9	4	3	1.72	0.083%
FOKN1_2117	transcription-repair coupling factor	130.1	5.37	38	1.4	3	3	0.09	0.004%
FOKN1_2118	uncharacterized protein	27.5	5.42	118	8.5	4	2	0.48	0.023%
FOKN1_2125	alkyl hydroperoxide reductase	16.9	4.6	129	25.3	5	4	1.48	0.071%
FOKN1_2126	5'-methylthioadenosine phosphorylase	27.2	5.72	150	11.7	5	4	0.84	0.040%
FOKN1_2128	esterase	35.0	5.82	91	10.5	3	3	0.40	0.019%
FOKN1_2137	succinate dehydrogenase/fumarate reductase, Fe-S protein subunit	27.2	5.26	48	3.9	2	2	0.30	0.014%
FOKN1_2138	succinate dehydrogenase/fumarate reductase, flavoprotein subunit	65.2	6.1	165	7	6	6	0.39	0.019%
FOKN1_2141	aminomethyl transferase	38.5	4.9	89	5.7	3	3	0.36	0.017%
FOKN1_2142	phosphodiesterase transmembrane protein	72.3	5.09	186	8.1	7	6	0.45	0.022%
FOKN1_2143	lysyl-tRNA synthetase	58.1	5.34	210	17.4	10	9	0.91	0.044%
FOKN1_2145	phospho-2-dehydro-3-deoxyheptonate aldolase	39.7	5.91	204	11.1	6	5	0.58	0.028%
FOKN1_2146	surface lipoprotein	24.5	5.02	81	9.8	2	2	0.37	0.018%
FOKN1_2149	DNA-binding protein	11.3	9.09	121	20.2	3	2	1.33	0.064%
FOKN1_2150	phenylalanine tRNA synthetase, beta-subunit	86.9	5.21	194	7.6	9	9	0.47	0.023%

Table S4. ***Thiohalobacter sp.*** **strain FOKN1 proteins identified on the basis of two or more peptides**. emPAI; exponentially modified protein abundance index, PCI; protein content index.

Locus_tag	Product	Molecular weight (kDa)	pI	Sum of mascot score	% of sequence coverage	Total no. of accepted peptides	No. of unique peptides	Sum of emPAI	PCI (%)
FOKN1_2151	phenylalanyl-tRNA synthetase, alpha subunit	38.5	5.33	93	5.6	3	3	0.34	0.016%
FOKN1_2152	50S ribosomal protein L20	13.6	11.53	338	32	10	7	6.52	0.313%
FOKN1_2154	translation initiation factor IF-3	18.0	9.21	34	11.7	2	2	0.53	0.025%
FOKN1_2155	threonyl-tRNA synthetase	73.9	5.4	444	22.2	19	15	1.44	0.069%
FOKN1_2161	aspartate/tyrosine/aromatic aminotransferase	42.9	5.07	184	12.7	6	5	0.64	0.031%
FOKN1_2163	UTP-glucose-1-phosphate uridylyltransferase	31.5	4.75	187	19.2	8	6	1.39	0.067%
FOKN1_2171	SAM-dependent methyltransferases	37.2	6.02	206	10.4	6	3	0.60	0.029%
FOKN1_2173	mannose-1-phosphate guanylyltransferase	53.7	4.74	211	8.2	5	4	0.42	0.020%
FOKN1_2175	orotidine 5'-phosphate decarboxylase	25.0	5.46	69	9.3	2	2	0.36	0.017%
FOKN1_2178	DNA-binding protein	11.8	9.85	376	34.9	8	4	4.00	0.192%
FOKN1_2179	30S ribosomal protein S1	62.1	4.86	653	24.7	33	17	3.01	0.145%
FOKN1_2181	5-enolpyruvylshikimate-3-phosphate synthase	45.5	4.97	278	15.3	8	6	0.92	0.044%
FOKN1_2183	histidinol-phosphate aminotransferase	40.1	5.53	168	9.5	4	4	0.44	0.021%
FOKN1_2184	prephenate dehydratase	39.8	5.58	213	9.4	6	4	0.56	0.027%
FOKN1_2185	NAD-binding D-isomer specific 2-hydroxyacid dehydrogenase	42.0	5.1	349	16	14	8	1.71	0.082%
FOKN1_2186	phosphoserine aminotransferase	39.6	4.92	305	20.6	10	8	1.32	0.063%
FOKN1_2187	DNA gyrase subunit A	94.6	5.14	508	12.8	18	12	1.33	0.064%
FOKN1_2189	transcriptional regulator	26.5	5.21	58	3.8	2	2	0.32	0.015%
FOKN1_2190	ompA/MotB domain protein	22.3	4.92	940	20.3	33	5	10.9	0.525%
FOKN1_2191	mechanosensitive ion channel protein MscS	30.3	5.06	144	5.4	6	2	0.57	0.027%

Table S4. *Thiohalobacter sp.* strain FOKN1 proteins identified on the basis of two or more peptides. emPAI; exponentially modified protein abundance index, PCI; protein content index.

Locus_tag	Product	Molecular weight (kDa)	pI	Sum of mascot score	% of sequence coverage	Total no. of accepted peptides	No. of unique peptides	Sum of emPAI	PCI (%)
FOKN1_2192	N-ethylammeline chlorohydrolase	47.9	5.12	133	15.3	6	5	0.60	0.029%
FOKN1_2193	3-demethylubiquinone-9 3-methyltransferase	27.1	5.46	27	5.4	2	2	0.33	0.016%
FOKN1_2195	uncharacterized protein	48.5	6.02	102	6.7	4	3	0.36	0.017%
FOKN1_2199	short chain dehydrogenase	28.4	5.04	89	3.8	2	2	0.30	0.014%
FOKN1_2200	thioredoxin-related protein	39.3	4.91	74	5.8	3	2	0.35	0.017%
FOKN1_2206	tryptophanyl-tRNA synthetase	46.2	5.9	160	10.6	5	4	0.49	0.024%
FOKN1_2221	universal stress protein	29.8	5.07	653	22.3	15	6	2.50	0.120%
FOKN1_2233	retron-type reverse transcriptase	140.4	6.04	44	0.8	2	2	0.13	0.006%
FOKN1_2235	type I restriction-modification system methyltransferase subunit	56.3	6.2	121	3.5	4	3	0.29	0.014%
FOKN1_2238	uncharacterized protein	48.4	6.7	40	3.1	2	2	0.16	0.008%
FOKN1_2249	ATP-dependent peptidase	91.1	5.01	215	6.9	11	8	0.54	0.026%
FOKN1_2254	fructose/tagatose bisphosphate aldolase	42.7	4.88	329	13.3	8	6	0.91	0.044%
FOKN1_2256	glutamyl- and glutaminyl-tRNA synthetases	53.6	5.69	93	6.6	6	5	0.53	0.025%
FOKN1_2258	signal transduction protein	72.1	5.67	121	3	5	4	0.30	0.014%
FOKN1_2261	universal stress protein	15.4	5.47	73	12.7	2	2	0.64	0.031%
FOKN1_2265	Zn-dependent proteases	19.9	6.43	169	19.2	4	4	1.44	0.069%
FOKN1_2266	thiosulfate sulfurtransferase	32.5	4.73	109	10.2	3	3	0.43	0.021%
FOKN1_2274	uncharacterized protein	61.2	5.09	108	5.8	3	3	0.21	0.010%
FOKN1_2277	coenzyme F420-reducing hydrogenase, alpha subunit	48.7	6.07	114	7.2	5	5	0.43	0.021%
FOKN1_2279	2-polyprenylphenol hydroxylase and related flavodoxin oxidoreductases	31.0	5.71	103	12.2	4	4	0.65	0.031%

Table S4. *Thiohalobacter sp.* strain FOKN1 proteins identified on the basis of two or more peptides. emPAI; exponentially modified protein abundance index, PCI; protein content index.

Locus_tag	Product	Molecular weight (kDa)	pI	Sum of mascot score	% of sequence coverage	Total no. of accepted peptides	No. of unique peptides	Sum of emPAI	PCI (%)
FOKN1_2282	Ni,Fe-hydrogenase I large subunit	65.8	5.58	666	16.8	22	13	1.54	0.074%
FOKN1_2283	Fe-S oxidoreductase	47.1	5.16	281	13.8	10	7	1.06	0.051%
FOKN1_2285	Ni,Fe-hydrogenase I small subunit	38.6	6.15	213	9.9	11	6	1.20	0.058%
FOKN1_2294	uncharacterized protein	30.7	5.71	46	6.3	3	2	0.42	0.020%
FOKN1_2295	UDP-glucose 4-epimerase	36.4	5.43	300	19.5	15	8	2.18	0.105%
FOKN1_2301	uncharacterized protein	125.1	8.78	22	2.6	2	2	0.15	0.007%
FOKN1_2306	ABC-type transporter, permease component	54.3	5.1	446	16.6	14	9	1.16	0.056%
FOKN1_2307	ABC-type transporter, ATPase component	27.7	4.86	150	11.6	5	3	0.85	0.041%
FOKN1_2308	ABC-type transporter, permease component	49.9	4.68	116	10.5	5	4	0.37	0.018%
FOKN1_2312	uncharacterized protein	17.6	4.87	338	41	13	9	6.63	0.319%
FOKN1_2313	glycosyltransferase	42.3	8.98	81	5.2	3	2	0.65	0.031%
FOKN1_2315	asparagine synthetase	67.7	5.71	334	9.5	13	9	0.90	0.043%
FOKN1_2317	O-antigen ligase	49.7	9.99	48	4.5	2	2	0.35	0.017%
FOKN1_2321	glycosyltransferase	44.8	8.61	62	4.2	2	2	0.19	0.009%
FOKN1_2322	asparagine synthase	70.9	6.4	65	3.4	2	2	0.26	0.013%
FOKN1_2326	FAD/FMN-containing dehydrogenases	39.9	6.91	110	8.6	4	4	0.44	0.021%
FOKN1_2328	UDP-N-acetylglucosamine 2-epimerase	42.1	6.04	49	2.6	2	2	0.20	0.010%
FOKN1_2329	general secretion pathway protein A	41.0	5.49	35	4.5	2	2	0.21	0.010%
FOKN1_2330	signal transduction histidine kinase	57.8	4.87	404	14.2	10	8	1.29	0.062%
FOKN1_2331	protein-tyrosine kinase	33.2	5.1	124	7.6	6	3	0.78	0.037%

Table S4. *Thiohalobacter sp.* strain FOKN1 proteins identified on the basis of two or more peptides. emPAI; exponentially modified protein abundance index, PCI; protein content index.

Locus_tag	Product	Molecular weight (kDa)	pI	Sum of mascot score	% of sequence coverage	Total no. of accepted peptides	No. of unique peptides	Sum of emPAI	PCI (%)
FOKN1_2333	polysaccharide export protein	22.5	4.72	339	29.3	16	6	3.47	0.167%
FOKN1_2335	dinucleotide-utilizing enzyme	72.9	8.9	105	5.2	4	3	0.52	0.025%
FOKN1_2337	SAM-dependent methyltransferases	32.1	5.83	111	9.3	3	3	0.44	0.021%
FOKN1_2338	uncharacterized protein	92.2	5.05	805	19.5	28	20	1.76	0.084%
FOKN1_2339	tetratricopeptide	101.3	5.25	278	9.7	13	10	0.58	0.028%
FOKN1_2340	response regulator receiver protein	50.3	5.13	84	7	3	3	0.27	0.013%
FOKN1_2344	NAD-dependent epimerase/dehydratase	37.9	5.49	279	17	6	5	0.79	0.038%
FOKN1_2345	UDP-N-acetyl-D-mannosaminuronate dehydrogenase	16.3	4.96	88	15.3	3	2	1.02	0.049%
FOKN1_2346	UDP-N-acetyl-D-mannosaminuronate dehydrogenase	29.9	5.26	276	34.1	9	8	1.97	0.095%
FOKN1_2350	membrane-bound lytic murein transglycosylase D	60.7	9.77	36	2.8	2	2	0.30	0.015%
FOKN1_2354	isoquinoline 1-oxidoreductase, beta subunit	78.6	5.07	201	5.4	5	4	0.38	0.018%
FOKN1_2358	glutaredoxin	14.7	9.5	85	15.6	2	2	0.69	0.033%
FOKN1_2361	glucan phosphorylase	96.4	5.27	96	2.9	4	3	0.17	0.008%
FOKN1_2363	dienelactone hydrolase	28.4	5.38	196	10.7	7	3	0.83	0.040%
FOKN1_2364	7-keto-8-aminopelargonate synthetase	19.2	6.62	71	19.6	3	3	0.82	0.039%
FOKN1_2365	uncharacterized protein	47.7	4.72	235	9.8	8	5	1.63	0.078%
FOKN1_2375	iron-containing alcohol dehydrogenase	41.9	5.22	71	7.6	3	3	0.32	0.015%
FOKN1_2376	uncharacterized protein	22.9	4.77	48	9.9	2	2	0.40	0.019%
FOKN1_2379	phosphoglucomutase	59.6	5.01	283	11.2	9	7	0.70	0.034%
FOKN1_2381	glutamate dehydrogenase	49.0	5.99	218	14.1	9	7	0.76	0.037%

Table S4. ***Thiohalobacter sp.*** **strain FOKN1 proteins identified on the basis of two or more peptides**. emPAI; exponentially modified protein abundance index, PCI; protein content index.

Locus_tag	Product	Molecular weight (kDa)	pI	Sum of mascot score	% of sequence coverage	Total no. of accepted peptides	No. of unique peptides	Sum of emPAI	PCI (%)
FOKN1_2382	phosphoenolpyruvate synthase	87.4	4.91	232	8	10	7	0.46	0.022%
FOKN1_2383	oligoribonuclease	21.2	5.39	54	14.4	4	4	1.07	0.051%
FOKN1_2386	pterin-4a-carbinolamine dehydratase	13.1	5.55	85	19.1	2	2	0.78	0.037%
FOKN1_2392	DNA-binding protein	11.5	4.86	57	21.5	3	3	2.54	0.122%
FOKN1_2393	DNA polymerase III, subunits gamma and tau	58.3	5.45	317	6.9	9	4	0.66	0.032%
FOKN1_2394	uncharacterized protein	14.3	5.02	104	20.2	3	3	1.02	0.049%
FOKN1_2395	SAM-dependent methyltransferases	32.0	5.46	273	31	11	9	2.28	0.110%
FOKN1_2397	S-adenosylmethionine synthetase	42.5	5	949	38.1	29	17	4.55	0.219%
FOKN1_2402	uncharacterized protein	84.3	5.61	66	1.7	2	2	0.22	0.010%
FOKN1_2405	uncharacterized protein	92.8	5.51	43	2.5	2	2	0.08	0.004%
FOKN1_2408	cytochrome c peroxidase	40.1	5.68	547	20.4	25	10	3.11	0.149%
FOKN1_2409	outer membrane lipoprotein	17.4	5.71	227	17.1	4	3	1.11	0.053%
FOKN1_2410	oxidoreductase	43.2	7.6	55	7.9	4	3	0.30	0.014%
FOKN1_2411	response regulator receiver domain protein	44.0	5.89	67	4.9	2	2	0.41	0.020%
FOKN1_2412	signal transduction histidine kinase	94.7	5.24	40	2.2	2	2	0.08	0.004%
FOKN1_2419	transmembrane protein	17.8	5.58	50	12.4	3	2	0.91	0.044%
FOKN1_2423	aspartate kinase	44.0	5.08	308	24	14	10	1.66	0.080%
FOKN1_2424	alanyl-tRNA synthetase	96.7	5.14	734	15.7	23	15	1.14	0.055%
FOKN1_2426	DNA strand exchange and recombination protein	37.5	5.09	672	32.7	23	11	3.88	0.186%
FOKN1_2428	uncharacterized protein	18.0	6.2	105	18.1	3	3	0.90	0.043%

Table S4. *Thiohalobacter sp.* strain FOKN1 proteins identified on the basis of two or more peptides. emPAI; exponentially modified protein abundance index, PCI; protein content index.

Locus_tag	Product	Molecular weight (kDa)	pI	Sum of mascot score	% of sequence coverage	Total no. of accepted peptides	No. of unique peptides	Sum of emPAI	PCI (%)
FOKN1_2430	DNA mismatch repair protein MutS	93.6	5.56	104	4.4	4	4	0.26	0.013%
FOKN1_2435	chemotaxis protein histidine kinase	98.3	5.04	27	1.7	2	2	0.08	0.004%
FOKN1_2442	protein-disulfide isomerase	26.9	5.11	219	20.9	7	5	1.22	0.059%
FOKN1_2447	uncharacterized protein	24.9	5.29	71	8.4	2	2	0.37	0.018%
FOKN1_2450	molybdopterin biosynthesis protein MoeA	43.9	5.75	84	5.7	3	2	0.29	0.014%
FOKN1_2451	ATP-dependent Lon protease	90.1	6.18	84	3.5	4	4	0.39	0.019%
FOKN1_2455	uncharacterized protein	45.4	4.87	107	10.3	4	4	0.41	0.020%
FOKN1_2456	ATPase	38.7	5.1	231	19	11	8	1.59	0.076%
FOKN1_2459	sulfate transporter	53.1	5.38	37	5.9	2	2	0.35	0.017%
FOKN1_2460	universal stress protein	30.8	5.4	83	8.6	2	2	0.29	0.014%
FOKN1_2463	heat shock protein 90	71.8	5	310	9.3	11	7	0.63	0.030%
FOKN1_2466	50S ribosomal protein L19	13.0	10.12	426	67.5	11	8	8.56	0.412%
FOKN1_2469	30S ribosomal protein S16	9.5	10.46	228	49.3	6	5	5.89	0.283%
FOKN1_2470	signal recognition particle protein	49.4	9.48	38	4.2	2	2	0.16	0.008%
FOKN1_2475	DNA repair protein RadA	48.4	7.11	109	6.8	3	3	0.28	0.013%
FOKN1_2481	replicative DNA helicase	51.7	4.94	131	4.5	2	2	0.17	0.008%
FOKN1_2482	50S ribosomal protein L9	16.1	4.92	314	50	10	8	5.75	0.276%
FOKN1_2484	30S ribosomal protein S18	8.7	10.39	71	35.1	6	4	9.85	0.473%
FOKN1_2486	30S ribosomal protein S6	14.3	4.9	135	36.5	6	5	2.80	0.135%
FOKN1_2487	23S rRNA (guanosine-2'-O-)-methyltransferase RlmB	27.1	7.85	147	12.7	4	3	0.69	0.033%

Table S4. ***Thiohalobacter sp.*** **strain FOKN1 proteins identified on the basis of two or more peptides**. emPAI; exponentially modified protein abundance index, PCI; protein content index.

Locus_tag	Product	Molecular weight (kDa)	pI	Sum of mascot score	% of sequence coverage	Total no. of accepted peptides	No. of unique peptides	Sum of emPAI	PCI (%)
FOKN1_2488	outer membrane protein A	25.3	5.3	149	11.7	6	2	2.22	0.107%
FOKN1_2489	exoribonuclease R	88.4	9.03	329	8.2	14	9	1.20	0.057%
FOKN1_2491	Zn-dependent hydrolases	37.4	4.76	73	6.5	2	2	0.23	0.011%
FOKN1_2492	adenylosuccinate synthase	48.3	5.6	518	22.7	14	10	1.45	0.070%
FOKN1_2493	histidyl-tRNA synthetase 2	42.8	5.04	143	4.3	4	2	0.40	0.019%
FOKN1_2495	membrane protease	33.4	5.54	639	29.3	26	12	5.22	0.251%
FOKN1_2496	membrane protease	41.6	5.62	818	24.6	26	12	3.59	0.173%
FOKN1_2497	GTPase	49.4	6.31	47	2.1	2	2	0.16	0.008%
FOKN1_2498	uncharacterized protein	9.8	9.3	175	21.3	5	2	2.61	0.125%
FOKN1_2500	DNA mismatch repair protein MutL	66.5	6.88	36	2.5	2	2	0.12	0.006%
FOKN1_2503	carbohydrate kinase	51.2	5.74	89	2.8	2	2	0.16	0.008%
FOKN1_2506	uroporphyrin-III C/tetrapyrrole(Corrin/Porphyrin) methyltransferase	30.7	5.87	84	12	3	3	0.46	0.022%
FOKN1_2507	lipoprotein	75.4	5.72	179	6.6	9	6	0.52	0.025%
FOKN1_2509	phosphoheptose isomerase	21.0	4.88	90	13.6	2	2	0.44	0.021%
FOKN1_2510	uncharacterized protein	21.2	8.64	869	45.4	33	13	21.8	1.050%
FOKN1_2513	signal transduction histidine kinase	40.6	4.94	37	3.3	2	2	0.20	0.010%
FOKN1_2541	glutathione S-transferase	24.0	5.2	277	14.4	9	5	1.60	0.077%
FOKN1_2544	ubiquinol-cytochrome c reductase, iron-sulfur subunit	21.4	5.17	181	14.8	5	3	1.15	0.055%
FOKN1_2549	periplasmic protein	12.6	9.34	146	16.2	3	2	1.18	0.057%
FOKN1_2552	ornithine/acetylornithine aminotransferase	42.2	5.7	121	3.6	2	2	0.20	0.010%

Table S4. ***Thiohalobacter sp.*** **strain FOKN1 proteins identified on the basis of two or more peptides**. emPAI; exponentially modified protein abundance index, PCI; protein content index.

Locus_tag	Product	Molecular weight (kDa)	pI	Sum of mascot score	% of sequence coverage	Total no. of accepted peptides	No. of unique peptides	Sum of emPAI	PCI (%)
FOKN1_2553	ornithine carbamoyltransferase	34.1	5.06	347	17	17	6	2.06	0.099%
FOKN1_2558	polyphosphate kinase	78.9	6.48	211	4.5	5	4	0.59	0.028%
FOKN1_2559	argininosuccinate synthase	45.5	4.99	318	18.8	15	10	1.88	0.090%
FOKN1_2561	pyruvate/2-oxoglutarate dehydrogenase complex, dihydrolipoamide dehydrogenase (E3) component	108.1	5.22	660	12.1	25	17	1.32	0.064%
FOKN1_2562	pyruvate/2-oxoglutarate dehydrogenase complex, dehydrogenase (E1) component, eukaryotic type, beta subunit	35.7	5.13	783	20.9	20	6	2.31	0.111%
FOKN1_2563	pyruvate/2-oxoglutarate dehydrogenase complex, dehydrogenase (E1) component, eukaryotic type, alpha subunit	37.5	5.91	414	21.8	26	11	3.68	0.177%
FOKN1_2564	dihydroorotase (dhoase) protein	37.8	5.85	326	12.5	8	4	0.87	0.042%
FOKN1_2565	uncharacterized protein	32.5	5.06	51	2.8	2	2	0.26	0.012%
FOKN1_2575	DNA polymerase III subunit epsilon	25.3	5.17	52	7.9	2	2	0.34	0.016%
FOKN1_2585	leucyl aminopeptidase	53.7	8.68	1013	22.1	27	16	4.05	0.195%
FOKN1_2587	valyl-tRNA synthetase	107.2	5.16	278	8	14	10	0.57	0.027%
FOKN1_2588	response regulator receiver	41.5	5.59	37	8.3	2	2	0.20	0.010%
FOKN1_2589	protoheme ferro-lyase	42.9	6.77	120	6.8	4	4	0.40	0.019%
FOKN1_2598	2-isopropylmalate synthase	56.4	5.08	844	33.2	25	17	2.68	0.129%
FOKN1_2601	uncharacterized protein	48.5	5.56	35	4.2	2	2	0.39	0.019%
FOKN1_2603	metal-dependent phosphohydrolase	75.3	5.73	86	2.8	2	2	0.24	0.011%
FOKN1_2606	ketol-acid reductoisomerase	37.0	5.28	1256	49.1	40	16	7.74	0.372%

Table S4. *Thiohalobacter sp.* strain FOKN1 proteins identified on the basis of two or more peptides. emPAI; exponentially modified protein abundance index, PCI; protein content index.

Locus_tag	Product	Molecular weight (kDa)	pI	Sum of mascot score	% of sequence coverage	Total no. of accepted peptides	No. of unique peptides	Sum of emPAI	PCI (%)
FOKN1_2607	acetolactate synthase isozyme III (small subunit) protein	18.0	5.16	193	21.5	8	5	2.71	0.130%
FOKN1_2608	acetolactate synthase isozyme III (large subunit) protein	63.0	5.65	399	19.1	13	11	1.03	0.050%
FOKN1_2610	prolyl-tRNA synthetase	62.2	5.06	265	16.1	13	8	0.87	0.042%
FOKN1_2611	aspartyl-tRNA synthetase	68.2	5.27	656	24	35	19	2.63	0.126%
FOKN1_2622	thymidine phosphorylase	59.7	6.01	177	8.2	6	6	0.46	0.022%
FOKN1_2625	short-chain dehydrogenase/reductase	31.0	9.21	85	3.6	2	2	0.26	0.012%
FOKN1_2628	ATPases, ATP-binding subunit	96.4	5.15	475	9.4	20	12	0.90	0.043%
FOKN1_2631	amino acid-binding ACT domain protein	18.4	4.79	81	9.8	2	2	0.52	0.025%
FOKN1_2635	DNA uptake lipoprotein	29.3	8.68	142	7.2	6	4	0.93	0.045%
FOKN1_2636	nitrogen regulatory protein P-II	12.5	5.3	218	37.5	7	4	3.81	0.183%
FOKN1_2637	NAD+ synthetase	60.4	5.29	176	6.4	3	3	0.22	0.011%
FOKN1_2638	succinyl-CoA synthetase subunit alpha	29.6	5.37	73	9	4	3	0.49	0.024%
FOKN1_2639	succinyl-CoA synthetase subunit beta	41.7	4.87	138	7.4	3	3	0.33	0.016%
FOKN1_2642	response regulator	49.4	5.44	98	7.1	4	3	0.35	0.017%
FOKN1_2643	TfP pilus assembly protein, major pilin PilA	14.2	4.43	663	17.8	13	3	4.38	0.211%
FOKN1_2645	uncharacterized protein	59.4	9.14	45	4	2	2	0.30	0.015%
FOKN1_2652	uncharacterized protein	29.2	5.8	37	8.2	3	2	0.31	0.015%
FOKN1_2654	uncharacterized protein	18.4	6.74	22	16.6	2	2	0.52	0.025%
FOKN1_2656	ornithine acetyltransferase/N-acetylglutamate synthase	42.6	4.96	326	11.6	8	6	0.64	0.031%
FOKN1_2657	preprotein translocase subunit SecA	103.1	5.18	302	12.5	17	15	0.98	0.047%

Table S4. ***Thiohalobacter sp.*** **strain FOKN1 proteins identified on the basis of two or more peptides**. emPAI; exponentially modified protein abundance index, PCI; protein content index.

Locus_tag	Product	Molecular weight (kDa)	pI	Sum of mascot score	% of sequence coverage	Total no. of accepted peptides	No. of unique peptides	Sum of emPAI	PCI (%)
FOKN1_2661	GTPase	40.9	4.46	44	5.4	2	2	0.21	0.010%
FOKN1_2662	cell division protein FtsA	44.4	5.37	232	12.4	8	6	0.84	0.040%
FOKN1_2664	D-alanine-D-alanine ligase	34.1	5.02	254	15.2	8	5	1.18	0.057%
FOKN1_2665	UDP-N-acetylenolpyruvoylglucosamine reductase	32.7	6.41	52	5.2	2	2	0.26	0.012%
FOKN1_2667	UDP-N-acetylmuramate-alanine ligase	51.9	5.46	203	6	6	3	0.51	0.025%
FOKN1_2670	UDP-N-acetylmuramoylalanine-D-glutamate ligase	46.9	5.17	78	5.9	2	2	0.18	0.009%
FOKN1_2672	UDP-N-acetylmuramyl pentapeptide synthase	47.6	5.86	120	4.8	3	3	0.27	0.013%
FOKN1_2676	S-adenosylmethionine-dependent methyltransferase	33.7	9.28	76	7.4	2	2	0.26	0.012%
FOKN1_2677	uncharacterized protein	17.2	5.42	70	14	2	2	0.56	0.027%
FOKN1_2679	single-strand DNA binding protein	16.5	5.48	431	51	15	9	7.29	0.350%
FOKN1_2681	excinuclease ABC subunit A	105.6	6	264	5	7	6	0.28	0.013%
FOKN1_2683	50S ribosomal protein L17	15.0	10.65	304	45.8	12	8	6.04	0.291%
FOKN1_2684	DNA-directed RNA polymerase, alpha subunit	36.6	4.92	800	34.6	26	12	4.12	0.198%
FOKN1_2685	30S ribosomal protein S4	24.0	9.98	348	31.3	10	7	3.17	0.152%
FOKN1_2686	30S ribosomal protein S11	13.8	11.04	312	26.4	8	6	4.19	0.201%
FOKN1_2687	30S ribosomal protein S13	13.5	11.57	365	65.3	17	11	13.8	0.661%
FOKN1_2689	50S ribosomal protein L15	15.0	10.62	590	81.3	18	10	11.1	0.536%
FOKN1_2690	50S ribosomal protein L30	6.9	10.21	102	34.4	3	2	1.88	0.090%
FOKN1_2691	30S ribosomal protein S5	17.6	10.05	733	71.5	21	13	10.5	0.505%
FOKN1_2692	50S ribosomal protein L18	13.0	10.66	315	48.3	10	7	6.94	0.334%

Table S4. *Thiohalobacter sp.* strain FOKN1 proteins identified on the basis of two or more peptides. emPAI; exponentially modified protein abundance index, PCI; protein content index.

Locus_tag	Product	Molecular weight (kDa)	pI	Sum of mascot score	% of sequence coverage	Total no. of accepted peptides	No. of unique peptides	Sum of emPAI	PCI (%)
FOKN1_2693	50S ribosomal protein L6	19.6	9.3	691	45.8	24	10	7.84	0.377%
FOKN1_2694	30s ribosomal protein s8	14.3	9.44	364	49.6	9	5	6.93	0.333%
FOKN1_2695	30S ribosomal protein S14p	11.7	10.74	145	12.9	4	2	2.80	0.135%
FOKN1_2696	50S ribosomal protein L5	20.3	9.43	524	36.9	18	9	9.20	0.442%
FOKN1_2697	50S ribosomal protein L24	11.4	9.99	256	49.5	9	8	8.10	0.389%
FOKN1_2698	50S ribosomal protein L14	13.5	10.42	227	60.6	14	8	8.32	0.400%
FOKN1_2699	30S ribosomal protein S17	10.5	9.34	279	65.9	10	8	14.6	0.700%
FOKN1_2700	50S ribosomal protein L29	7.7	10.57	46	13.4	2	2	1.23	0.059%
FOKN1_2701	50S ribosomal protein L16	15.5	11.43	118	13.9	3	2	0.91	0.044%
FOKN1_2702	30S ribosomal protein S3	25.4	10.05	372	32.3	14	11	5.41	0.260%
FOKN1_2703	50S ribosomal protein L22	12.1	10.7	246	46.8	8	5	6.94	0.334%
FOKN1_2704	30S ribosomal protein S19	10.0	11.03	146	21.1	3	3	3.39	0.163%
FOKN1_2705	50S ribosomal protein L2	30.2	11.34	561	28.8	21	8	5.21	0.250%
FOKN1_2706	50S ribosomal protein L23	11.1	9.87	320	68.4	13	8	11.6	0.558%
FOKN1_2708	50S ribosomal protein L3	21.5	10.04	866	85	39	20	29.7	1.430%
FOKN1_2709	30S ribosomal protein S10	11.8	9.81	164	43.7	7	6	4.56	0.219%
FOKN1_2710	translation elongation factor Tu	43.3	4.91	3447	71	112	28	15.7	0.754%
FOKN1_2711	translation elongation factor G	77.7	5.01	1566	35.9	54	28	4.06	0.195%
FOKN1_2712	30S ribosomal protein S7	17.6	10.19	551	44.5	12	6	4.97	0.239%
FOKN1_2713	30S ribosomal protein S12	13.9	11.63	526	41.1	13	6	7.68	0.369%

Table S4. ***Thiohalobacter sp.*** **strain FOKN1 proteins identified on the basis of two or more peptides**. emPAI; exponentially modified protein abundance index, PCI; protein content index.

Locus_tag	Product	Molecular weight (kDa)	pI	Sum of mascot score	% of sequence coverage	Total no. of accepted peptides	No. of unique peptides	Sum of emPAI	PCI (%)
FOKN1_2714	DNA-directed RNA polymerase subunit beta	156.0	5.66	2378	24.3	104	44	5.26	0.253%
FOKN1_2715	DNA-directed RNA polymerase subunit beta	154.1	5.24	2495	25.7	94	44	4.43	0.213%
FOKN1_2716	50S ribosomal protein L7/L12	12.9	4.44	161	25.4	8	5	4.84	0.233%
FOKN1_2717	50S ribosomal protein L10	19.3	9.38	379	50.6	20	12	9.50	0.457%
FOKN1_2718	50S ribosomal protein L1	24.1	9.21	216	32.5	6	6	1.97	0.095%
FOKN1_2719	50S ribosomal protein L11	15.2	8.71	714	53.9	25	9	9.87	0.475%
FOKN1_2723	translation elongation factor Tu	43.3	4.91	3046	71	90	28	14.3	0.685%
FOKN1_2727	UDP-glucose 6-dehydrogenase	49.0	4.99	428	26.4	13	11	1.69	0.081%
FOKN1_2731	alcohol dehydrogenase	35.2	5.94	559	24.5	17	10	2.96	0.142%
FOKN1_2737	transcriptional repressor	21.8	5.45	67	9.2	2	2	0.42	0.020%
FOKN1_2742	transcriptional regulator	28.3	5.97	67	7.6	2	2	0.32	0.015%
FOKN1_2752	tyrosyl-tRNA synthetase	44.9	5.5	53	5.2	3	2	0.27	0.013%
FOKN1_2759	N-acetyl-gamma-glutamyl-phosphate reductase	36.7	6.04	60	8.5	3	3	0.38	0.018%
FOKN1_2762	thiol-disulfide isomerase and thioredoxins	18.2	5.91	84	10.2	3	2	0.76	0.037%
FOKN1_2768	signal transduction protein	81.8	4.95	152	5.6	4	4	0.21	0.010%
FOKN1_2775	iron complex outer membrane recepter protein	64.2	5.23	969	34.9	43	24	4.40	0.212%
FOKN1_2777	cobalamin adenosyltransferase protein	20.2	5.67	112	19.1	3	2	0.46	0.022%
FOKN1_2781	GTPase	68.6	5.35	87	7.6	5	5	0.33	0.016%
FOKN1_2788	uncharacterized protein	18.3	5.07	133	18.6	6	3	1.40	0.067%
FOKN1_2792	3-hydroxydecanoyl-ACP dehydratase	19.8	5.6	114	23.3	5	4	1.18	0.057%

Table S4. *Thiohalobacter sp.* strain FOKN1 proteins identified on the basis of two or more peptides. emPAI; exponentially modified protein abundance index, PCI; protein content index.

Locus_tag	Product	Molecular weight (kDa)	pI	Sum of mascot score	% of sequence coverage	Total no. of accepted peptides	No. of unique peptides	Sum of emPAI	PCI (%)
FOKN1_2793	3-oxoacyl-ACP synthase	43.6	5.26	281	12.5	6	5	0.66	0.032%
FOKN1_2796	ATP-dependent helicase HrpA	147.5	8.64	86	2.6	4	4	0.19	0.009%
FOKN1_2814	CRISPR-associated protein Cas5/CasD, subtype TIGR01868	27.9	5.75	103	7.8	2	2	0.32	0.015%
FOKN1_2815	CRISPR-associated protein, Cse4 family	38.7	5.8	281	10.8	8	6	1.53	0.073%
FOKN1_2817	CRISPR-associated protein, Cse1	61.2	5.87	53	4.1	3	2	0.21	0.010%
FOKN1_2829	GTPase	39.7	4.95	48	5	2	2	0.22	0.011%
FOKN1_2836	uncharacterized protein	64.8	4.68	342	13.3	12	8	0.83	0.040%
FOKN1_2844	hydrolase	31.7	5.35	39	4.6	2	2	0.28	0.013%
FOKN1_2846	Asp-tRNA Asn/Glu-tRNA Gln amidotransferase B subunit	52.7	5.1	543	23.9	21	14	2.38	0.114%
FOKN1_2847	Asp-tRNA Asn/Glu-tRNA Gln amidotransferase A subunit	52.4	5.47	614	25.2	22	13	2.14	0.103%
FOKN1_2849	rod shape-determining protein MreB	37.5	5.29	650	39.6	22	13	3.89	0.187%
FOKN1_2854	lytic murein transglycosylase B	36.8	5.34	85	8.2	2	2	0.24	0.012%
FOKN1_2856	D-alanyl-D-alanine carboxypeptidase	42.6	5.36	309	22.1	13	10	1.61	0.077%
FOKN1_2857	D-amino-acid transaminase	31.6	5.21	168	9.8	4	3	0.58	0.028%
FOKN1_2859	cobalt/zinc/cadmium efflux transporter	46.0	5.58	172	5	4	3	0.38	0.018%
FOKN1_2860	outer membrane protein	46.3	9.39	211	19.7	8	8	1.61	0.078%
FOKN1_2875	peptidyl-prolyl isomerase	49.3	4.91	204	6.9	5	4	0.43	0.021%
FOKN1_2876	4-hydroxythreonine-4-phosphate dehydrogenase	35.4	5.69	78	10.6	3	3	0.39	0.019%
FOKN1_2879	universal stress protein UspA	16.1	5.66	120	21.1	4	3	1.59	0.076%
FOKN1_2895	uncharacterized protein	78.1	4.98	194	5.7	6	5	0.34	0.016%

Table S4. *Thiohalobacter sp.* strain FOKN1 proteins identified on the basis of two or more peptides. emPAI; exponentially modified protein abundance index, PCI; protein content index.

Locus_tag	Product	Molecular weight (kDa)	pI	Sum of mascot score	% of sequence coverage	Total no. of accepted peptides	No. of unique peptides	Sum of emPAI	PCI (%)
FOKN1_2901	sugar kinases	34.7	5.18	342	16.7	8	6	1.12	0.054%
FOKN1_2903	glycine dehydrogenase subunit 2	53.4	5.88	232	18.9	11	8	0.96	0.046%
FOKN1_2905	glycine dehydrogenase subunit 1	49.6	5.25	77	2.2	2	2	0.16	0.008%
FOKN1_2907	glycine cleavage system aminomethyltransferase T	40.2	5.31	112	4.7	4	3	0.32	0.015%
FOKN1_2908	cytidylate kinase	44.8	6.71	37	3.8	2	2	0.18	0.009%
FOKN1_2909	2-polyprenyl-6-methoxyphenol hydroxylase and related FAD-dependent oxidoreductases	44.1	5.53	124	7.6	4	3	0.31	0.015%
FOKN1_2914	uncharacterized protein	12.2	6.59	98	34	6	5	2.81	0.135%
FOKN1_2917	uncharacterized protein	18.2	7.82	86	28.3	5	5	1.57	0.075%
FOKN1_2919	threonine dehydratase	56.1	6.59	86	4	4	3	0.65	0.031%
FOKN1_2920	ribose-5-phosphate isomerase A	23.1	5.44	195	15	5	4	1.14	0.055%
FOKN1_2921	FAD/FMN-containing dehydrogenases	145.4	5.99	136	3.1	6	6	0.39	0.019%
FOKN1_2922	porin	36.3	4.44	3362	46.8	119	14	26.1	1.257%
FOKN1_2925	30S ribosomal protein S9	14.8	11.04	124	22.3	5	4	2.70	0.130%
FOKN1_2926	50S ribosomal protein L13	16.0	9.68	351	63.4	18	13	13.3	0.641%
FOKN1_2930	S-adenosylmethionine decarboxylase	30.9	6.18	236	14.3	8	5	1.30	0.062%
FOKN1_2933	DNA-binding transcriptional dual regulator Crp	24.2	6.91	134	11.2	4	2	0.76	0.037%
FOKN1_2935	anthranilate phosphoribosyltransferase	36.0	5.32	88	7	2	2	0.24	0.012%
FOKN1_2938	anthranilate synthase component I	54.8	4.99	179	10.1	5	5	0.43	0.021%
FOKN1_2940	ribulose-phosphate 3-epimerase	24.8	5.09	343	19.3	13	8	2.39	0.115%

Table S4. *Thiohalobacter sp.* strain FOKN1 proteins identified on the basis of two or more peptides. emPAI; exponentially modified protein abundance index, PCI; protein content index.

Locus_tag	Product	Molecular weight (kDa)	pI	Sum of mascot score	% of sequence coverage	Total no. of accepted peptides	No. of unique peptides	Sum of emPAI	PCI (%)
FOKN1_2950	DNA-binding protein	12.7	10.51	375	72.7	16	10	14.3	0.686%
FOKN1_2953	type II secretory pathway, component ExeA	63.5	6.7	244	9	11	9	0.77	0.037%
FOKN1_2957	ribonucleotide reductase, alpha subunit	26.1	5.46	68	8.3	4	3	0.70	0.034%
FOKN1_2958	ribonucleotide reductase, alpha subunit	81.3	5.39	339	14.9	12	10	0.83	0.040%
FOKN1_2960	molecular chaperon	17.2	5.21	143	41.2	7	7	3.08	0.148%
FOKN1_2963	heat shock protein Hsp20	19.2	6.99	115	18.6	4	4	1.40	0.067%
FOKN1_2964	histone acetyltransferase HPA2	16.8	4.55	131	21.3	6	3	1.56	0.075%
FOKN1_2971	ABC-type molybdate transporter, periplasmic component	27.6	9.34	111	4.8	2	2	0.30	0.014%
FOKN1_2979	fructose-bisphosphate aldolase	38.7	5.19	1309	42.6	54	21	9.54	0.458%
FOKN1_2980	pyruvate kinase	52.9	5.82	360	15.4	13	9	2.20	0.106%
FOKN1_2981	phosphoglycerate kinase	41.8	5.17	1330	44.7	49	21	9.17	0.441%
FOKN1_2982	glyceraldehyde-3-phosphate dehydrogenase/erythrose-4-phosphate dehydrogenase	36.0	5.53	2234	52.5	102	22	18.8	0.903%
FOKN1_2983	transketolase	72.6	5.35	3001	41.3	119	31	8.97	0.431%
FOKN1_2986	S-adenosylmethionine synthetase	42.8	5.33	114	8.2	4	3	0.32	0.015%
FOKN1_2987	S-adenosyl-L-homocysteine hydrolase	52.3	4.96	2699	47.5	94	34	12.8	0.615%
FOKN1_2988	5,10-methylenetetrahydrofolate reductase	32.3	6.05	341	24.6	11	7	1.73	0.083%
FOKN1_2998	chemotaxis protein histidine kinase	204.5	4.38	39	1.1	2	2	0.04	0.002%
FOKN1_3000	methyl-accepting chemotaxis protein	71.0	4.54	55	2.7	3	2	0.18	0.009%
FOKN1_3005	glutathione synthetase	36.0	4.91	57	7.9	2	2	0.24	0.012%

Table S4. *Thiohalobacter sp.* strain FOKN1 proteins identified on the basis of two or more peptides. emPAI; exponentially modified protein abundance index, PCI; protein content index.

Locus_tag	Product	Molecular weight (kDa)	pI	Sum of mascot score	% of sequence coverage	Total no. of accepted peptides	No. of unique peptides	Sum of emPAI	PCI (%)
FOKN1_3006	lipoprotein	38.0	5.47	311	10.6	8	5	0.89	0.043%
FOKN1_3013	uracil phosphoribosyltransferase	18.3	5.15	92	12	3	2	0.75	0.036%
FOKN1_3014	aspartate carbamoyltransferase catalytic subunit	36.1	6.49	521	24.1	16	8	2.25	0.108%
FOKN1_3015	dihydroorotase	46.6	5.82	264	14.8	8	5	0.81	0.039%
FOKN1_3018	TfP pilus assembly protein, ATPase PilU	43.7	5.93	142	13.9	7	6	0.80	0.038%
FOKN1_3019	TfP pilus assembly protein, pilus retraction ATPase PilT	38.3	6.28	523	22.6	20	10	2.66	0.128%
FOKN1_3021	pyrroline-5-carboxylate reductase	28.9	4.8	141	11.2	6	4	0.78	0.037%
FOKN1_3025	uncharacterized protein	16.8	6.62	425	29.5	15	7	6.47	0.311%
FOKN1_3028	orotate phosphoribosyltransferase	23.1	5.02	172	11.7	3	2	0.58	0.028%
FOKN1_3031	acetylglutamate kinase	31.8	5.79	314	17.9	8	6	1.55	0.074%
FOKN1_3032	phosphomannomutase	92.6	5.24	384	11	18	11	1.44	0.069%
FOKN1_3033	deoxyuridine 5'-triphosphatenucleotidohydrolase	16.4	5.26	226	13.2	6	4	1.71	0.082%
FOKN1_3034	phosphopantothenoylcysteine decarboxylase/phosphopantothenate-cysteine ligase	48.0	7.75	102	4.1	4	4	0.78	0.038%
FOKN1_3036	50S ribosomal protein L28	9.1	11.46	60	34.6	3	3	5.22	0.251%
FOKN1_3040	diguanylate phosphodiesterase	45.6	4.81	36	4.5	2	2	0.19	0.009%
FOKN1_3042	ABC-type multidrug transporter, ATPase component	34.9	6.47	71	7	2	2	0.25	0.012%
FOKN1_3044	ABC-type uncharacterized transporte, auxiliary component	50.4	5.12	374	11.4	10	5	1.78	0.086%
FOKN1_3051	pantetheine-phosphate adenylyltransferase	17.4	5.94	98	15.1	3	3	0.81	0.039%
FOKN1_3054	Zn-dependent peptidases	47.7	5.55	672	40.6	22	18	4.04	0.194%

Table S4. *Thiohalobacter sp.* strain FOKN1 proteins identified on the basis of two or more peptides. emPAI; exponentially modified protein abundance index, PCI; protein content index.

Locus_tag	Product	Molecular weight (kDa)	pI	Sum of mascot score	% of sequence coverage	Total no. of accepted peptides	No. of unique peptides	Sum of emPAI	PCI (%)
FOKN1_3055	Zn-dependent peptidases	49.3	5.43	478	24.5	16	12	1.87	0.090%
FOKN1_3056	GTPase	37.4	5.34	79	2.6	2	2	0.22	0.011%
FOKN1_3071	uncharacterized protein	29.5	5.03	48	6	2	2	0.28	0.013%
FOKN1_3072	transcriptional regulator	52.8	5.33	108	5.3	4	3	0.33	0.016%
